# Rational Design, Synthesis and Biological Evaluation of Pyrimidine-4,6-diamine derivatives as Type-II inhibitors of FLT3 Selective Against c-KIT

**DOI:** 10.1038/s41598-018-21839-3

**Published:** 2018-02-27

**Authors:** Jaideep B. Bharate, Nicholas McConnell, Gunaganti Naresh, Lingtian Zhang, Naga Rajiv Lakkaniga, Lucky Ding, Neil P. Shah, Brendan Frett, Hong-yu Li

**Affiliations:** 1 Department of Pharmaceutical Sciences, University of Arkansas for Medical Sciences, Little Rock, 72205 USA; 2 Department of Pharmacology and Toxicology, The University of Arizona, Tucson, AZ 85721 USA; 3 Division of Hematology/Oncology, University of California, San Francisco, CA 94143 USA; 4 Helen Diller Family Comprehensive Cancer Center, University of California, San Francisco, CA 94115 USA

## Abstract

FMS-like Tyrosine Kinase 3 (FLT3) is a clinically validated target for acute myeloid leukemia (AML). Inhibitors targeting FLT3 have been evaluated in clinical studies and have exhibited potential to treat FLT3-driven AML. A frequent, clinical limitation is FLT3 selectivity, as concomitant inhibition of FLT3 and c-KIT is thought to cause dose-limiting myelosuppression. Through a rational design approach, novel FLT3 inhibitors were synthesized employing a pyridine/pyrimidine warhead. The most potent compound identified from the studies is compound **13a**, which exhibited an IC_50_ value of 13.9 ± 6.5 nM against the FLT3 kinase with high selectivity over c-KIT. Mechanism of action studies suggested that **13a** is a Type-II kinase inhibitor, which was also supported through computer aided drug discovery (CADD) efforts. Cell-based assays identified that **13a** was potent on a variety of FLT3-driven cell lines with clinical relevance. We report herein the discovery and therapeutic evaluation of 4,6-diamino pyrimidine-based Type-II FLT3 inhibitors, which can serve as a FLT3-selective scaffold for further clinical development.

## Introduction

**Figure 1:**
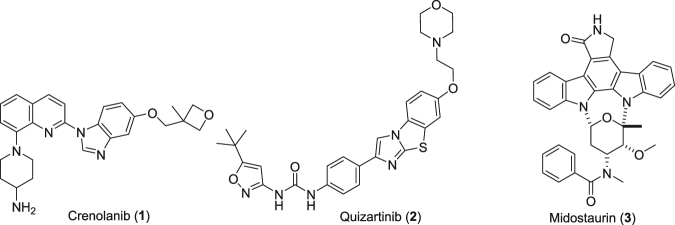
Representative examples of FLT3 kinase inhibitors **1–3** in clinical trials.

FMS-like tyrosine kinase-3 (FLT3) is a member of the receptor tyrosine kinase family. It is predominantly expressed on hematopoietic progenitor cells but is also found in other tissues such as placenta, gonads, and brain. This kinase is important for hematopoiesis and the immune system. The activation of FLT3 through a mutation is recognized as the most common molecular abnormality in acute myeloid leukemia (AML), and these mutations also play a role in other hematologic malignancies^1,2^. The majority of AMLs and acute lymphoblastic leukemias (ALL) have overexpression of FLT3. Therefore, this kinase has been an attractive target for AML. The observation that a majority of patients treated with a potent FLT3 inhibitor who developed acquired resistance harbored newly detected secondary kinase domain mutations in FLT3-ITD^3^ definitively validating FLT3-ITD as a therapeutic target in human AML. In recent years, several research groups have worked on the discovery and development of potent FLT3 inhibitors^4–12^. A large number of FLT3 small-molecule kinase inhibitors are under clinical investigation such as crenolanib (**1**)^13–16^, AC220 (quizartinib, **2**)^17–19^ and midostaurin (**3**) (Fig. 1)^20^. On April 28, 2017, Novartis’s midostaurin (PKC412) received FDA approval for the treatment of FLT3-ITD^+^ AML^21–23^.

**Figure 2:**
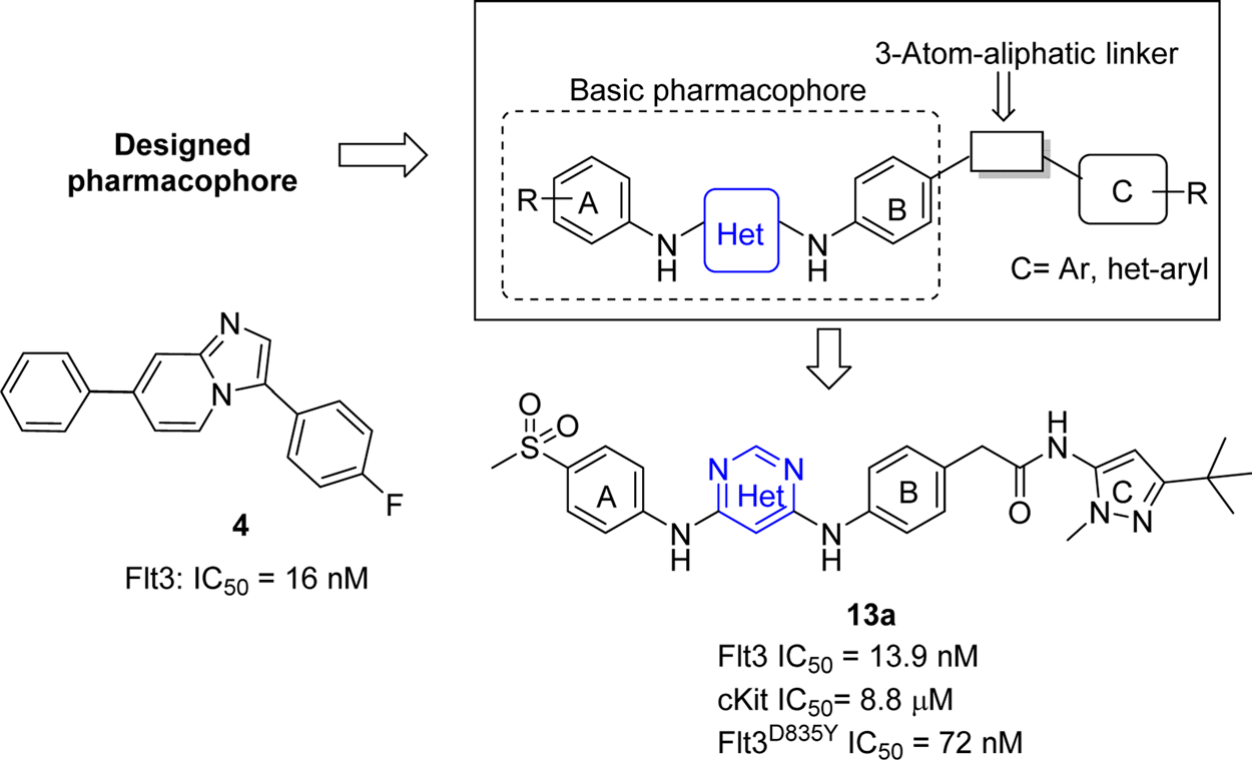
Design strategy for novel FLT3 inhibitors. The basic heterocycle in blue was designed to exploit the hinge region, while the C-region heterocycle was designed to access the allosteric pocket. At the basic pharmacophore region, free rotation was engineered into the scaffold to help improve FLT3 selectivity. Design strategies were implemented through CADD and rational design efforts in the SAR discussion.

Recently, through computer aided drug discovery (CADD) followed by *in-vitro* validation, our group has discovered an imidazopyridine core (example **4**) as a unique heterocycle inhibiting the FLT3 kinase^24^. The imidazopyridine **4** showed FLT3 inhibition with an IC_50_ value of 16 nM. Similarly, other heterocycles, such as those containing pyrimidine, have been reported as FLT3 kinase inhibitors. Han’s group^25–27^ reported thieno[2,3-d]pyrimidines as potent FLT3 inhibitors. Literature precedence also indicated that molecules with longer structures [3–4 aromatic rings connected via small linkers, e.g. quizartinib (**2**)] possess potent activity against the FLT3 kinase. Therefore, we designed a series of longer compounds comprising pyrimidine as the kinase-hinge warhead (central heterocycle shown in Fig. 2) connected to two aromatic rings (A and B) via an amine bond. We hypothesized these compounds to be potent inhibitors of the FLT3 kinase through CADD studies. The most potent pyrimidine compound identified from our study is compound **13a**, which displayed inhibition of the FLT3 kinase at both the enzymatic and cellular levels (Fig. 2). Further, compound **13a** was found to be highly selective for FLT3 over c-KIT. This is an important discovery as dual inhibition of c-KIT and FLT3 causes a ‘synthetic lethal toxicity’ leading to myelosuppression^28^. Therefore, **13a** represents a significant finding to produce second-generation FLT3 inhibitors with attenuated myelosuppression potential. In the following report, we discuss the discovery and therapeutic evaluation of 4,6-diamino pyrimidines as selective, FLT3 inhibitors.

## Results

### Chemistry

**Figure 3:**
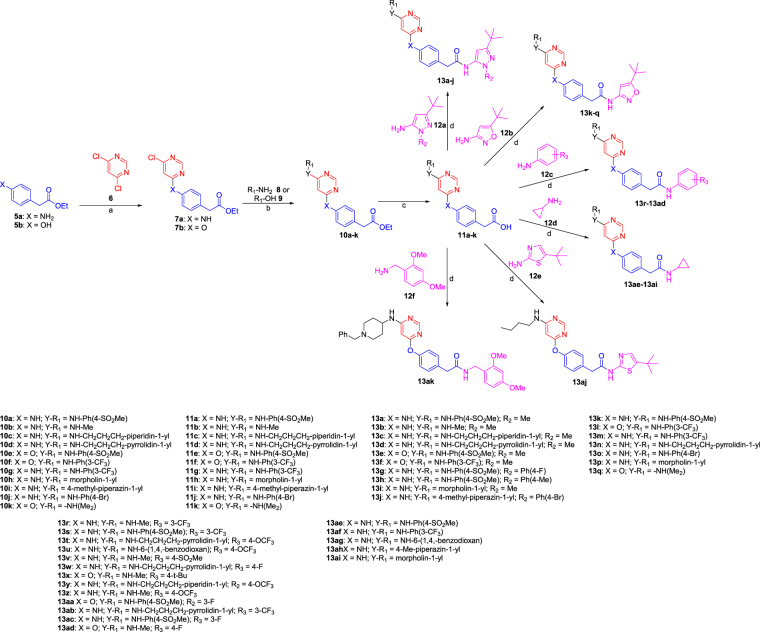
Synthesis of the 4,6-diaminopyrimidine series **13a-13ak**. Reagents and conditions: (**a**) Et_3_N (1.25 equiv.), ethanol, 80 °C, 12 h, 80%; (**b**) Pd(PPh_3_)_4_, (0.03 equiv.), Cs_2_CO_3_ (2.5 equiv.), dioxane, 110 °C 12 h, 76%; (**c**) LiOH (2.5 equiv.), 1:1 THF/Water, 100 °C, 15 min, MWI, 95%; (**d**) EDC (2.5 equiv.), HOAt (1.0 equiv.), DIPEA (1.2 equiv), DMF, RT, 12 h, 40%.

**Figure 4:**
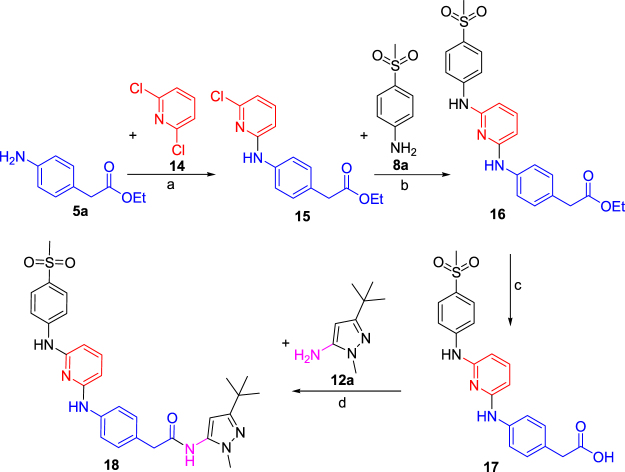
Synthesis of the 2,6-diaminopyridine series of compound **18**. Reagents and conditions: (**a**) Et_3_N (1.25 equiv.), ethanol, 80 °C, 12 h, 80%; (**b**) Pd(PPh_3_)_4_, (0.03 equiv.), Cs_2_CO_3_ (2.5 equiv.), dioxane, 110 °C, 12 h, 76%; (**c**) LiOH (2.5 equiv.), 1:1 THF/water, 100 °C, 15 min, MWI, 95%; (**d**) EDC (2.5 equiv.), HOAt (1.0 equiv.), DIPEA (1.2 equiv), DMF, RT, 12 h, 40%

**Figure 5:**
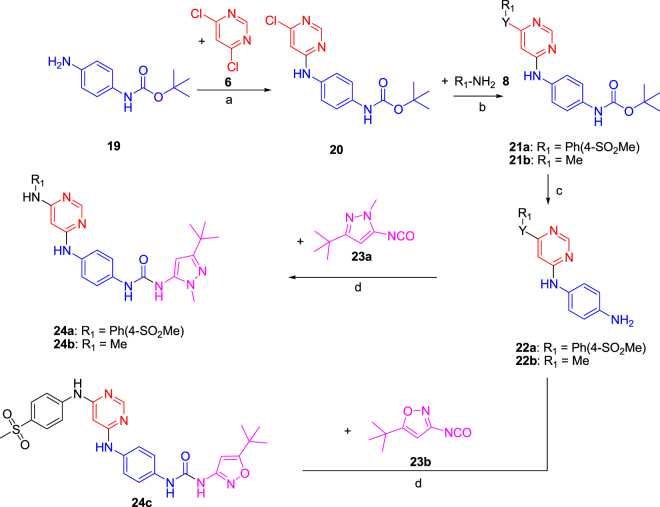
Synthesis of the 4,6-diaminopyrimidine urea series **24a-c**. Reagents and conditions: (**a**) Et_3_N (1.25 equiv.), ethanol 80 °C, 12 h, 80%; (**b**) Pd(PPh_3_)_4_, (0.03 equiv.), Cs_2_CO_3_ (2.5 equiv.), dioxane, 110 °C, 12 h, 76%; (**c**) TFA, DCM, RT, 2 h, 95%; (**d**) Et_3_N (1.5 equiv.), DCM, 45 °C, 5 h, 30%.

Two scaffolds were investigated for FLT3 inhibition, one comprising 4,6-diamino pyrimidine and the other containing 2,6-diamino pyridine. Synthesis of the pyrimidine series involved a direct nucleophilic substitution on commercially available 4,6-dichloropyrimidine (**6**) with ethyl 2-(4-aminophenyl)acetate (**5a**) or ethyl 2-(4-hydroxy phenyl)acetate (**5b**) leading to the formation of intermediate **7**. Buchwald coupling of intermediate **7** with aromatic or aliphatic amines **8** or alcohols **9** afforded intermediates **10**. LiOH-mediated saponification of the ester, followed by amide-bond formation with various aromatic and heteroaromatic amines **12**, resulted in the formation of the final products **13a-aj**. The overall yield for the four step synthetic scheme was ~40%. The synthesis of analogs **13a-ak** is depicted in Fig. 3. The second scaffold, utilizing 2,6-diaminopyridine as the warhead (**18**) was also synthesized using a similar synthetic route, as depicted in Fig. 4. 4,6-Diaminopyrimidine urea derivatives **24a-c**, wherein the benzylic -CH_2_- was replaced with –NH-, were also prepared to see the effect of limiting rotatable bonds at the kinase bridge region. Three compounds were prepared in this series, two with pyrazole (compounds **24a-b**) and one with isoxazole (compound **24c**) Fig. 5.

### FLT3 inhibition

All synthesized compounds were screened for inhibition of the FLT3 kinase. The initial screening was performed at a single point concentration of 20 µM to determine preliminary activity. After, IC_50_ values were determined for all active compounds. FLT3 inhibition and LE (ligand efficiency) values for the pyrimidine series are provided in Tables 1–3. Table 1 contains the data for the pyrimidine series of compounds **13a**-**13j**, wherein the other terminal of the scaffold comprises a pyrazole ring. Table 2 contains the data for pyrimidine series of compounds **13k**-**13q**, wherein the other terminal of the scaffold comprises an isoxazole ring. Table 3 contains the data for pyrimidine series of compounds **13r**-**13ak**, wherein the other terminal of the scaffold comprises a phenyl ring. Pyridine-based compound **18** showed inhibition of the FLT3 kinase with an IC_50_ value of 5719 nM.

**Table 1:** *In-vitro* inhibition of FLT3 kinase by pyrimidine series of compounds **13a-13j** and urea derivative **24a-b**.


Entry	R_1_	R_2_	X	FLT3^a^ IC_50_ (nM) ± SD	LE
**13a**	-Ph(4-SO_2_Me)	-Me	NH	13.9 ± 6.5	0.28
**13b**	-Me	-Me	NH	37.5 ± 13	0.35
**13c**		-Me	NH	129.3 ± 0.9	0.26
**13d**		-Me	NH	377 ± 20	0.24
**13e**	-Ph(4-SO_2_Me)	-Me	O	267.6 ± 25	0.24
**13f**	-Ph(3-CF_3_)	-Me	O	961 ± 33.7	0.22
**13g**	-Ph(4-SO_2_Me)	-Ph(4-F)	NH	3225 ± 556	0.17
**13h**	-Ph(4-SO_2_Me)	-Ph(4-Me)	NH	4842 ± 170	0.16
**13i**	Morpholine	-Me	NH	428 ± 99	0.27
**13j**	1-methylpiperazine	-Ph(4-Br)	NH	6024 ± 122	0.18
**24a**	-Ph(4-SO_2_Me)	-Me	NH	41 ± 6.8	0.27
**24b**	-Me	-Me	NH	38.2 ± 1.5	0.37

^a^IC_50_ values are expressed in nM units and are the results of three independent experiments.

**Table 2:** *In-vitro* inhibition of FLT3 kinase by pyrimidine series of compounds 13k-q and 24c.


Entry	R_1_	X	FLT3^a^ IC_50_ (nM) ± SD	LE
**13k**	-Ph(4-SO_2_Me)	NH	63.0 ± 43	0.27
**13l**	-Ph(3-CF_3_)	O	567.5 ± 62	0.23
**13m**	-Ph(3-CF_3_)	NH	1181 ± 527	0.22
**13n**		NH	1033 ± 121	0.23
**13o**	-Ph(4-Br)	NH	14800 ± 248	0.19
**13p**	Morpholine	NH	395 ± 78	0.28
**13q**	Dimethylamine	O	8763 ± 127	0.24
**24c**	-Ph(4-SO_2_Me)	NH	29 ± 1.9	0.28

^a^IC_50_ values are expressed in nM units and are the results of three independent experiments.

**Table 3:** *In-vitro* inhibition of FLT3 kinase by pyrimidine series of compounds 13r-13ak.


Entry	R_1_	R_2_	X	FLT3^a^ IC_50_ (nM) ± SD	LE
**13r**	-Me	-Ph(3-CF_3_)	NH	411 ± 236	0.30
**13s**	-Ph(4-SO_2_Me)	-Ph(3-CF_3_)	NH	860 ± 30	0.22
**13t**		-Ph(4-OCF_3_)	NH	909 ± 95.9	0.22
**13u**	6–1,4-benzodioxan	-Ph(4-OCF_3_)	NH	1319 ± 257	0.21
**13v**	-Me	-Ph(4-SO_2_Me)	NH	2119 ± 401	0.27
**13w**		-CH_2_Ph(4-F)	NH	4555 ± 330	0.21
**13x**	-Me	-Ph(4-tert-butyl)	O	5085 ± 75.5	0.25
**13y**		-Ph(4-OCF_3_)	NH	6460 ± 251	0.19
**13z**	-Me	-Ph(4-OCF_3_)	NH	7373 ± 333	0.23
**13aa**	-Ph(4-SO_2_Me)	-Ph(3-F)	O	7878 ± 29	0.20
**13ab**		-Ph(3-CF_3_)	NH	9167 ± 236	0.19
**13ac**	-Ph(4-SO_2_Me)	-Ph(3-F)	NH	11707 ± 106	0.19
**13ad**	-Me	-Ph(4-F)	O	12829 ± 834	0.26
**13ae**	-Ph(4-SO_2_Me)	-Cyclopropyl	NH	5553 ± 169	0.23
**13af**	-Ph-3-CF_3_	-Cyclopropyl	NH	8065 ± 274	0.23
**13ag**	6–1,4-benzodioxan	-Cyclopropyl	NH	10627 ± 320	0.22
**13ah**	1-methyl piperazine	-Cyclopropyl	NH	13556 ± 102	0.25
**13ai**	Morpholine	-Cyclopropyl	NH	15111 ± 168	0.25
**13aj**	-(CH_2_)_3_CH_3_	4-tert-butyl-thiazole	O	7577 ± 132	0.23
**13ak**	1-benzyl piperidine	-CH_2_Ph(2,4-Di-OMe)	O	13641 ± 494	0.16

^a^IC_50_ values are expressed in nM units and are the results of three independent experiments.

Amongst all compounds tested, the pyrimidine series of compounds, wherein the other terminal comprises a pyrazole ring, exhibited promising FLT3 inhibition with IC_50_ values in the lower nanomolar range (Table 1). The most potent compound, **13a**, displayed inhibition of the FLT3 kinase with an IC_50_ value of 13.9 nM. Interestingly, the urea analog (compound **24a**) of compound **13a**, exhibited an IC_50_ value of 41 nM against the FLT3 kinase. This suggests that free rotation at the kinase bridge region could be an important factor for potent inhibition.

### Kinase selectivity

**Figure 6:**
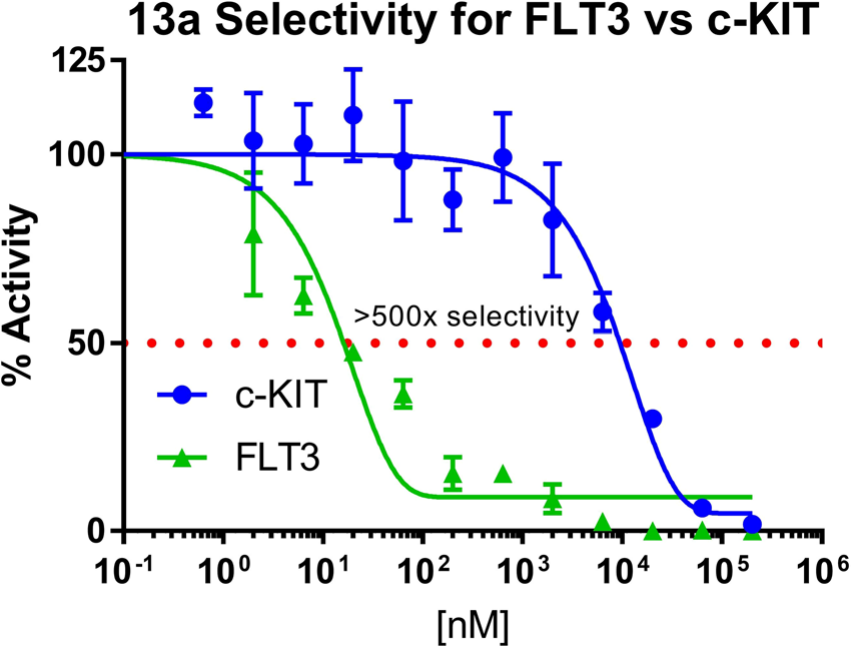
Compound **13a** screened against FLT3 and c-KIT with corresponding dose-response curves. **13a** was found >500x selective for FLT3 over c-KIT.

FLT3 inhibitors **13a** and **13k** were further studied for effects on other kinases in the greater kinome (Table 4). Compound **13a** exhibited weak inhibition of CSF-1R, RET, and Aurora B with IC_50_ values of 13 µM, 4.17 µM, and 79.83% inhibition at 20.0 µM, respectively. Compound **13a** displayed excellent activity for the activation loop mutation, FLT3^D835Y^, with an IC_50_ value of 72.5 nM. At the enzymatic level, compound **13a** is very selective for the FLT3 kinase with >100 fold less inhibition on other closely related kinases. Importantly, compound **13a** has >500 fold selectivity for FLT3 over c-KIT (Fig. 6). This selectivity could help reduce the incidence of toxicity as concomitant blockade of FLT3 and c-KIT has been shown to cause myelosuppression^28^. Interestingly, the only difference between **13a** and **13k** is ring C (the basic pharmacophore is depicted in Fig. 2). In the case of **13a**, ring C is a pyrazole while in **13k** it is an isoxazole. Therefore, it appears that FLT3 prefers pyrazole functionality at the R_2_ position. In another pair **13** **f (**IC_50_ = 961 nM) *versus* **13** **l** (IC_50_ = 567 nM), where the R_1_ substituent is 3-CF_3_ phenyl and ring C is either pyrazole or isoxazole, the trend of FLT3 inhibition was found to be opposite.

**Table 4:** *In-vitro* selectivity of compound **13a** and **13k**.

Kinase	^a^IC_50_ (nM) ± SD	
	13a	13k
FLT3	13.93 ± 6.5	63.02 ± 12
FLT3D835Y	72.52 ± 14.4	13713 ± 234
c-KIT	8,799 ± 2,083	/
CSF-1R	13,040 ± 231	60.7% at 20 µM
RET	4710 ± 342	1,664
RETV804L	62.3% at 20 µM	40.8% at 20 µM
Aurora B	79.8% at 20 µM	71.9% at 20 µM

^a^IC_50_ values are expressed in nM units and are the results of three independent experiments.

### Cell Based Studies

Compounds were progressed to cell-based studies to further evaluate their therapeutic utility. Three FLT3-driven cell line models were utilized for the studies: FLT3-driven Ba/F3 cells, Molm 14 cells with various FLT3 mutations, and MV4–11 AML cells. As can be seen from the data in Table 5, compound **13a** exhibited potent inhibitory activity against Ba/F3 FLT3-ITD, Molm14 par, and MV4-11 cells. However, despite having *in vitro* inhibitory activity against the FLT3^D835Y^ mutation, compound **13a** displayed a ~10-fold reduction in cellular activity against the Molm14^D835Y^ cell line. Compound **13a** also displayed a ~10-fold reduction against the Molm14^F691L^ cell line, which harbors a “gatekeeper” mutation. Therefore, **13a** is a potent and selective inhibitor of FLT3-ITD but exhibits a moderate reduction in activity against FLT3-ITD harboring secondary kinase domain mutations in cell-based studies. Other inhibitors screened, such as compound **13d**, exhibited good FLT3 inhibition but failed to effectively inhibit FLT3-driven cellular growth. This can be attributed to the high polarity of **13d** in which the compound has difficulty diffusing through the cellular membrane. Compound **13k** exhibited the greatest overall potency against FLT3-driven Ba/F3 and MV4-11 cells. Based on *in vitro* enzymatic screening, this result was expected as compound **13k** had very weak inhibition against the FLT3^D835Y^ mutant kinase.

**Table 5:** Cell-based inhibitory activity of compounds **13a**, **13b**, **13d**, **13k**, **13r**, **13v**, and **13ag** in FLT3-driven cell lines.

Cell Line	GI_50_ (nM)						
	13a	13b	13d	13k	13r	13v	13ag
Ba/F3 FLT3-ITD	131.3	158.5	/	40.5	266.4	>10000	4013
Molm14 par	24.4	186.7	/	19.8	186.7	>10000	1826
Molm14^D835Y^	1842	2226	/	2609	2226	>10000	/
Molm14^F691L^	1345	1346	/	2142	1346	>10000	/
MV4–11	9.9	80.6	2251	7.2	80.6	>10000	527

### Computational Studies

**Figure 7:**
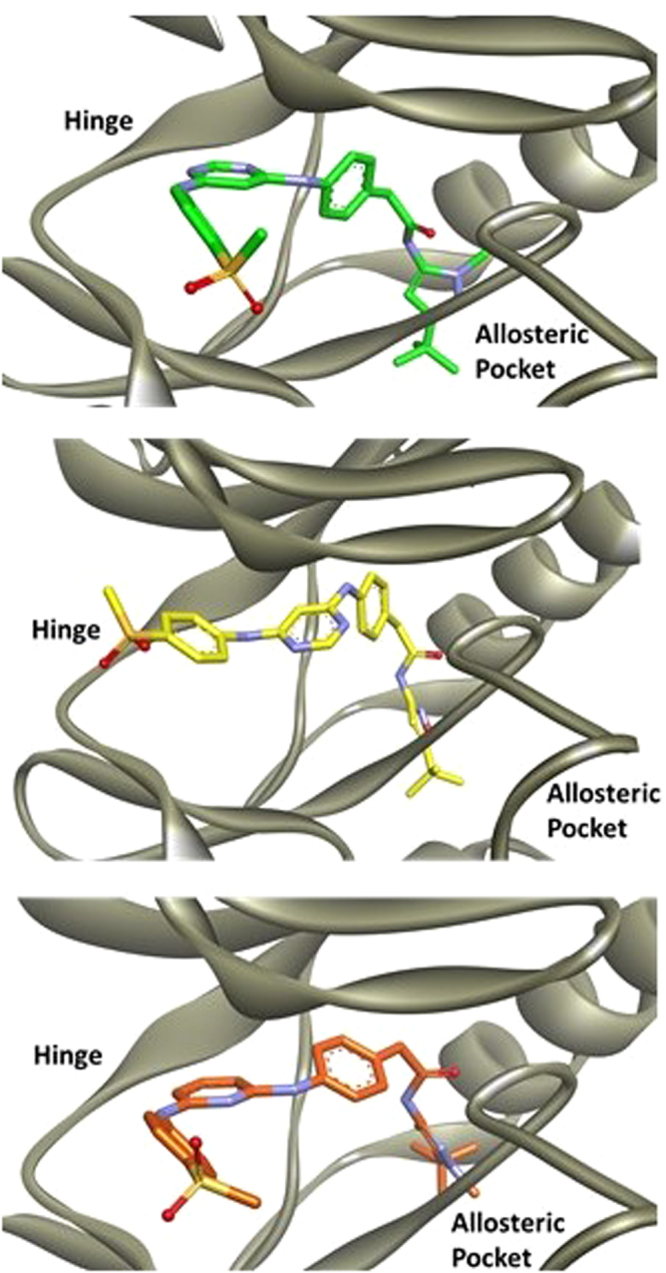
Compound **13a** (green), **13k** (yellow), and **18** (orange) computationally modeled in the FLT3 kinase.

To further understand ligand/receptor binding interactions, **13a**, **13k**, and **18** were computationally modeled in the FLT3 tyrosine kinase (Fig. 7). In all instances, each compound is predicted to bind to the FLT3 kinase in a Type-II DFG-out fashion. The R2 substituent accesses an allosteric pocket while the R1 substituent is oriented towards the solvent. The pyrimidine heterocycle remains in close proximity to the kinase hinge, forming a hydrogen-bond network. From the enzymatic screening results, compound **13a** drastically out-performed compound **18**. The difference in activity can be attributed to the ability to form hydrogen bonds at the hinge. As can be seen with compound **13a**, the pyrimidine is oriented towards the hinge and forms a hydrogen bond network. However, compound **18** is based on a pyridine scaffold, which is predicted to face away from the hinge. Therefore, **18** is unable to engage in hydrogen-bond interactions at the FLT3 kinase hinge despite having a similar structure to that of **13a**.

**Figure 8:**
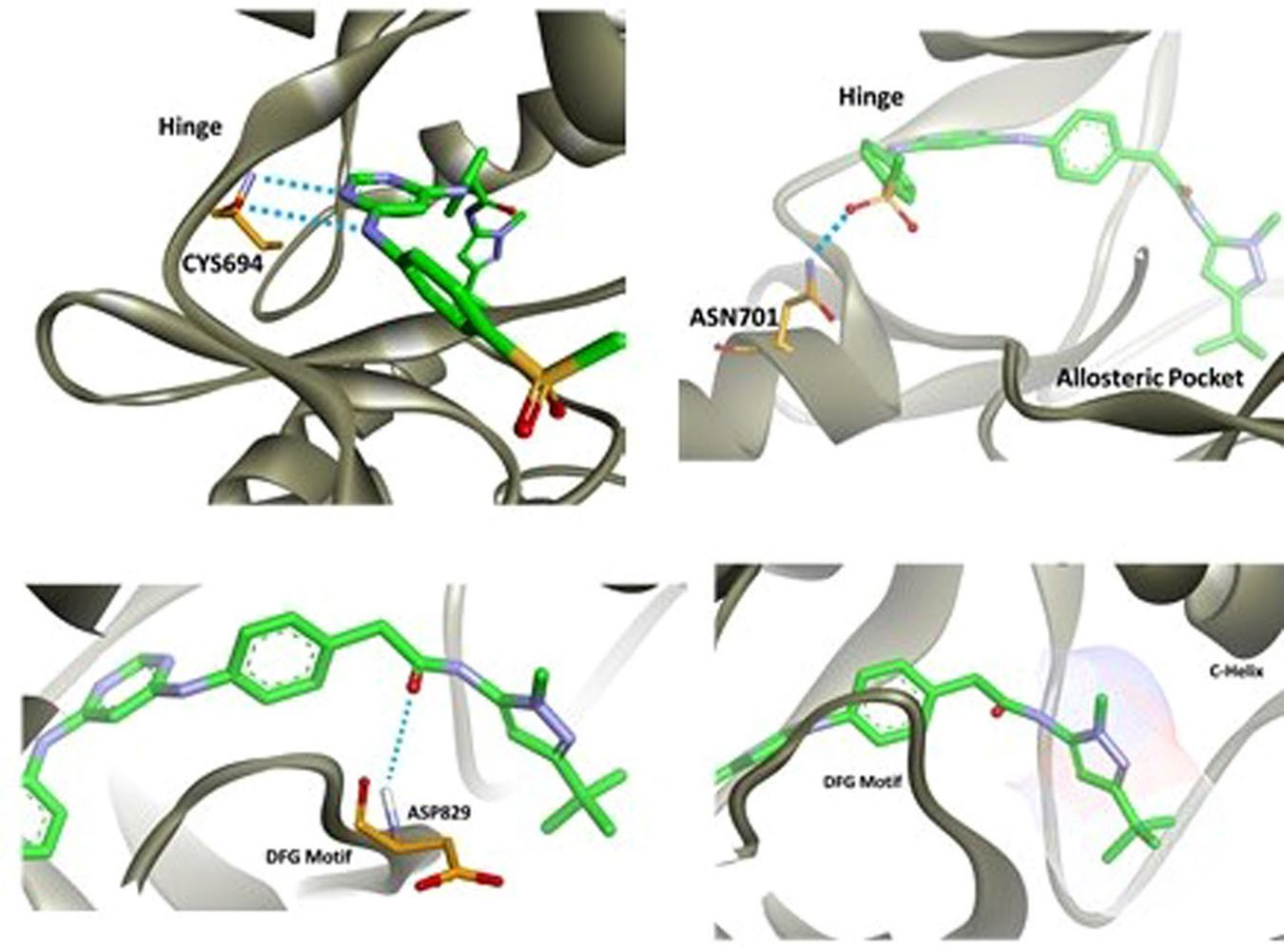
Compound **13a** computationally modeled in the FLT3 kinase.

Employing computational modeling, FLT3 binding interactions were further studied with compound **13a** (Fig. 8). At the hinge of FLT3, compound **13a** is predicted to engage in two hydrogen bonds with CYS694. Both hydrogen bonds occur with the amide backbone of FLT3. Other kinase inhibitors, such as sunitinib (SUTENT®), also engage in two hydrogen bonds at the hinge region. These hydrogen bond networks are essential for activity since compound **18**, a compound that cannot readily form hydrogen bonds at the hinge region, is >100 fold less active than **13a**. At the solvent region, the methyl sulfone substituent of **13a** is predicted to interact with ASN701. It is predicted that this interaction limits the free rotation of 4-(methylsulfonyl)phenyl and helps hold the ring system in place. This stabilization ensures proper hydrogen bonding at the hinge. Compound **13a** is able to access the FLT3 allosteric pocket by forming a hydrogen bond with ASP829 from the DFG motif at the bridge region. This interaction stabilizes the DFG motif in the ‘out’ position and permits the pyrazole moiety of **13a** to enter the allosteric pocket. As can be observed from the docking pose of **13a**, the methylene linker is rotated relative to the amide bond. It is predicted that this free rotation permits tighter binding since compound **24a**, the non-rotatable, urea analog of **13a**, exhibited ~3 fold lower activity. Within the allosteric pocket, the pyrazole substituent of **13a** efficiently fills the region. Because the isoxazole moiety at R_2_, as observed with compound **13k**, is ~5 fold less active than **13a** the isoxazole is not supreme in the allosteric pocket. On the pyrazole of **13a** there is a methyl, as well as a t-butyl, and it is hypothesized that both of these aliphatic groups are important to efficiently fill the FLT3 allosteric pocket.

### Mechanism of Inhibition

**Figure 9:**
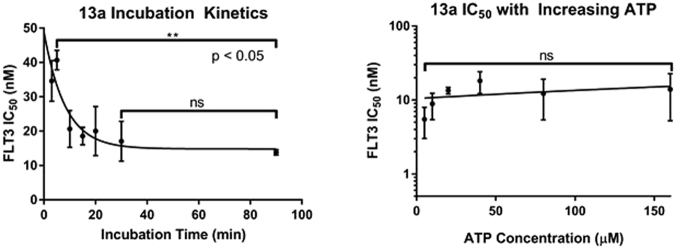
FLT3 inhibition kinetics with compound **13a**. Pre-incubation with **13a** causes a statistically significant increase in FLT3 inhibition (p < 0.05). An increase in ATP concentration does not cause a statistical significant increase in the FLT3 IC_50_ value (p > 0.05). Taken together, incubation kinetics suggest **13a** is non-competitive, Type-II kinase inhibitor.

Computational modeling studies suggested that compound **13a** bound to the DFG-out conformation of the FLT3 kinase and mechanistic inhibition studies were completed to confirm binding. First, compound **13a** was pre-incubated with the FLT3 kinase at various time intervals. Type-II kinase inhibitors display a time-dependent binding interaction, where an increase in incubation causes an increase in compound activity. This is because the compound has to push into the allosteric pocket of the kinase, which causes the inhibitor to have a time dependent k_on_. As can be seen from the incubation studies, compound **13a** had an increase in IC_50_ with an increase in pre-incubation (Fig. 9). From no incubation to a 90-minute pre-incubation, the IC_50_ value exhibited a statistically significant decrease (p < 0.05). In order for **13a** to reach maximal inhibition of the FLT3 kinase, the compound required approximately 60 minutes of pre-incubation. This is highly suggestive that compound **13a** is accessing the FLT3 allosteric pocket and is a Type-II kinase inhibitor because the compound exhibits statistically significant, time-dependent activity. In a separate experiment, the IC_50_ value of **13a** was determined at various concentrations of ATP (Fig. 9). Because **13a** is hypothesized to access an allosteric pocket on the FLT3 kinase, the compound was expected to display non-competitive inhibition characteristics and should not be directly competitive with ATP. It was determined that an increase in ATP concentration did not cause a statistically significant increase in FLT3 IC_50_. Therefore, ATP is unable to relieve the FLT3 inhibition induced by **13a** because **13a** binds to the DFG-out form of the kinase, and the DFG-out conformation does not have affinity for ATP. Therefore, these mechanistic studies are highly suggestive that compound **13a** is a Type-II, non-competitive inhibitor that accesses the FLT3 allosteric pocket.

### FLT3 vs c-KIT selectivity

**Figure 10:**
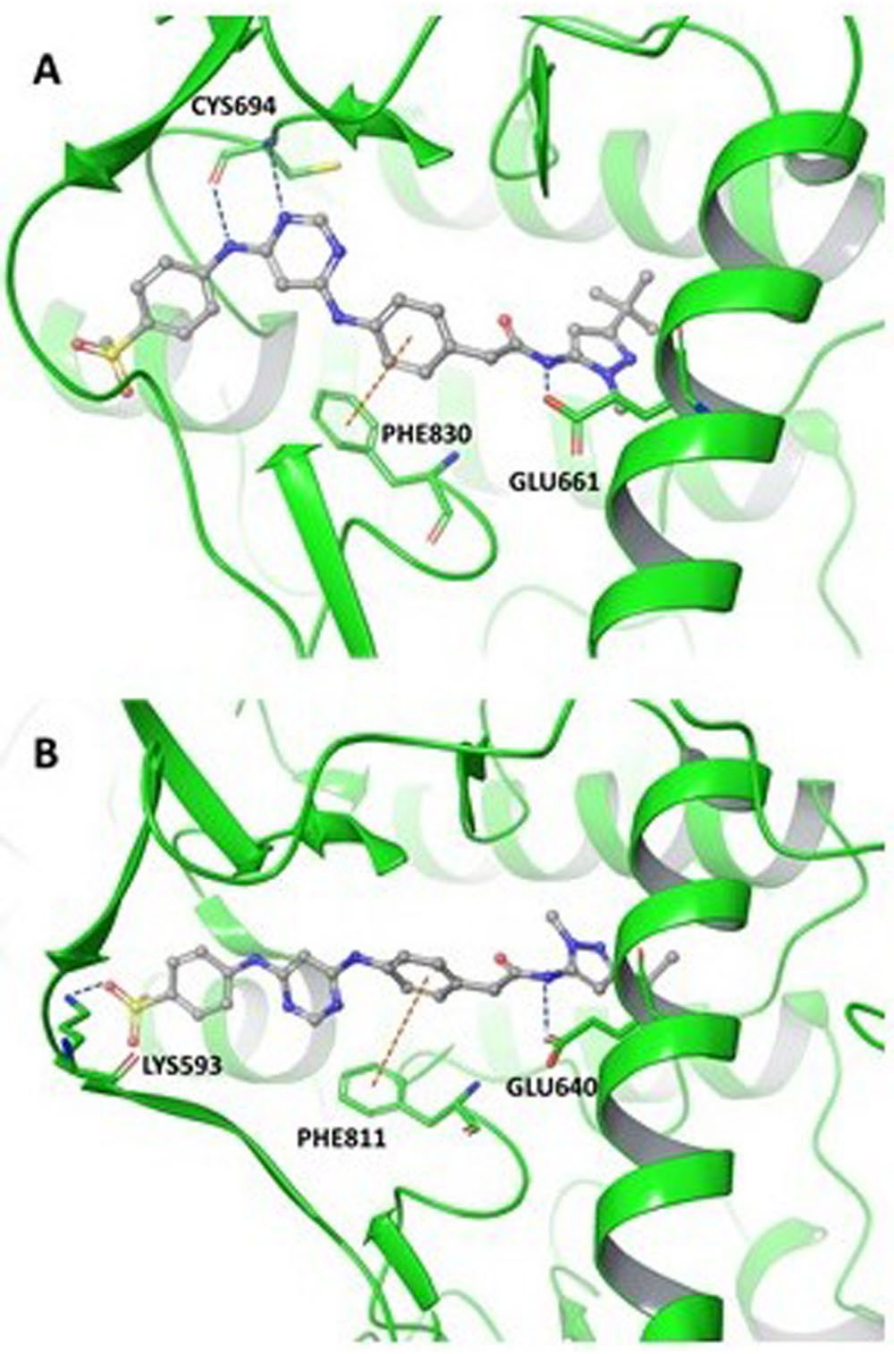
Compound **13a** computationally modeled in FLT3 (**A**) and c-KIT (**B**).

To understand the selectivity of **13a** for FLT3 over c-KIT, molecular modeling studies were performed. It was identified that **13a** does not form the correct hydrogen bonding network at the hinge of c-KIT. As can be seen in the FLT3 docking structure, **13a** forms two hydrogen bonds at CYS694, which is the hinge region. However, in c-KIT, **13a** is oriented away from the hinge, and is unable to form essential hydrogen bonds (Fig. 10). This is likely due to how **13a** interacts in the allosteric region as the pyrazole moiety is flipped between FLT3 and c-KIT. In fact, the computational binding affinity of **13a** for FLT3 was determined to be −14.479 kcal/mol while c-KIT was −8.895 kcal/mol. Therefore, computationally **13a** is very selective for FLT3 over c-KIT, which was also confirmed experimentally.

## Discussion

Using computational studies, a series of extended compounds comprising pyrimidine as the kinase-hinge warhead connected to two aromatic rings (A and B) via an amine bond were synthesized. Amongst synthesized compounds, compound **13a**, displayed inhibition of the FLT3 kinase with an IC_50_ value of 13.9 nM. The FLT3 inhibition results presented in Tables 1–4 are indicative of the fact that there are several key structure-activity relationship features. These features include: (1) The central heterocycle as pyrimidine (Tables 1–3) is favored over pyridine. (2) Heterocycles such as pyrazole or isoxazoles as “ring C” (Tables 1–2) are preferred over a simple phenyl ring (Table 3) and amongst pyrazole and oxazole, pyrazole is preferred. (3) *N*-methyl substitution on pyrazole (ring C) is preferred over other bulkier groups (Table 1). (4) The linkage of the central heterocycle with ring B via –NH– is preferred over –*O*– linkage (Tables 1–3). (5) Replacement of ring A with a single methyl does not have significant impact on FLT3 inhibitory activity (**13a** *versus* **13b**) (Table 1). (6) The 3-atom linker between ring B and C can be –CH_2_-CO-NH– or –NH-CO-NH–, which both exhibit a similar level of activity (Tables 1–2). (7) It appears that the *t-butyl* substitution on ring C is essential for efficient interaction of the inhibitor with the allosteric pocket (Tables 1–3 and Figs 7–8). (8) The carbonyl group of the 3-atom linker exhibits critical hydrogen bonding interactions with ASP829 of the DFG motif (Fig. 8). Based on these key SAR features, compound **13a** was determined to be the best and most potent inhibitor of FLT3.

In a cellular growth inhibition assay, compound **13a** displayed excellent activity for the activation loop mutation, FLT3^D835Y^, with an IC_50_ value of 72.5 nM and was found to possess >500 fold selectivity for FLT3 over c-KIT in *in vitro* kinase assays. The discrepancy between the enzymatic inhibition of FLT3^D835Y^ and cellular inhibition of Molm14^D835Y^ is likely because Molm14^D835Y^ cells express a FLT3 double mutant^29^. Molm14^D835Y^ cells contain an internal tandem duplication (ITD) and the D835Y point mutation in the FLT3 kinase. Kinase double mutations are notoriously more difficult to inhibit^29,30^, while the biochemical assay only contains the kinase domain with a single, D835Y point mutation.

In computational studies, compound **13a** was found to enter the allosteric pocket, which was further confirmed by time-dependent enzyme kinetic studies. Compound **13a** had an increase in IC_50_ with an increase in pre-incubation, which is suggesting that the compound is accessing the FLT3 allosteric pocket and is a Type-II kinase inhibitor. Furthermore, the selectivity of compound **13a** towards FLT3 over c-KIT was studied using molecular modeling and was then experimentally demonstrated.

In conclusion, the rational design of a series of 4,6-diamino pyrimidnyl compounds led to the discovery of a potent and selective FLT3 kinase inhibitor **13a**. Compound **13a** achieved a FLT3 IC_50_ value of 13.9 nM and was active against FLT3-driven cell lines. **13a** was also active on the FLT3^D835Y^ mutant with an IC_50_ of 72.5 nM and displayed activity in FLT3 cell lines with GI_50_ between 0.009–2.0 µM. From these studies, it was determined that the allosteric pocket of FLT3 is more responsive to pyrazole when compared to isoxazole. Further, from computational modeling, it was identified that amino acid ASN701 can interact with **13a**, which can aid in the design of future FLT3 inhibitors. Mechanistic inhibition studies suggested that **13a** is a Type-II, non-competitive inhibitor that accesses the FLT3 allosteric pocket. **13a** was also found to be highly selective for FLT3 over c-KIT, which can aid in the discovery of second-generation FLT3 inhibitors with attenuated myelosuppressive profiles. Currently, further modification of lead **13a** is underway to enhance activity on activation loop and gatekeeper mutations.

## Methods

### General

All solvents were reagent grade or HPLC grade and all starting materials were obtained from commercial sources and used without further purification. Purity of final compounds was assessed using a Thermo Finnigan LCQ Deca with Thermo Surveyor LCMS System at variable wavelengths of 254 nm and 214 nm and final compound purity was >95%. The HPLC mobile phase consisted of a water-methanol gradient buffered with 0.1% formic acid. ^1^H NMR spectra were recorded at 400 MHz and ^13^C spectra were recorded at 100 MHz, both completed on a Varian 400 MHz instrument (Model# 4001S41ASP). High-resolution mass spectrometry was completed using a Bruker 9.4 T Apex-Qh hybrid Fourier transfer ion-cyclotron resonance (FT-ICR) mass spectrometer. Compound activity was determined with the EZ Reader II plate reader (PerkinElmer®, Walthman, USA). All compounds were purified using Silica gel (0.035–0.070 mm, 60 Å) flash chromatography, unless otherwise noted. Microwave assisted reactions were completed in sealed vessels using a Biotage Initiator microwave synthesizer.

### Synthesis of ethyl 2-(4-(6-chloropyrimidin-4-yl)amino)phenyl)acetate (7)

To the mixture of ethyl 4-aminophenyl acetate **5** (20 g, 111.60 mmol) and 4,6-dichloropyrimidine **6** (13.79 g, 92.56 mmol) in EtOH (200 mL) was added triethyl amine (17.80 g, 176.23 mmol) The reaction mixture was stirred at 80 °C for 12 h. After completion of reaction as indicated by TLC, the solvent was removed under reduced pressure. The crude product thus obtained was purified by silica gel (100–200 mesh) flash chromatography with hexanes/EtOAc (1:3) to afford **7** as white solid (26.50 g, 81.53%); m.p. 132–134 °C; ^1^H NMR (400 MHz, DMSO-d_6_): δ 9.82 (s, 1H), 8.42 (s, 1H), 7.51 (d, *J* = 8.0 Hz, 2 H), 7.20 (d, *J* = 8.0 Hz, 2H), 6.74 (s, 1H), 4.04 (q, *J* = 8.0, 16.0 Hz, 2H), 3.58 (s, 2H), 1.14 (t, *J* = 8.0 Hz, 3H); ^13^C NMR (100 MHz, DMSO-d_6_): δ 171.62, 161.63, 158.89, 158.35, 137.97, 130.20, 129.80, 120.92, 105.26, 60.66, 14.50; LC-MS (ESI): *m/z* 292.1309 [M + H]^+^.

### Synthesis of ethyl 2-(4-(6-(4-methylsulfonyl)phenylamino)pyrimidin-4-ylamino)phenyl)acetate (10)

The 4-(methylsulfonyl)benzenamine **8a** (0.882 g, 5.5 mmol) and Cs_2_CO_3_ (2.79 g, 8.58 mmol) were added to the solution of compound **7** (1 g, 3.4 mmol) in dioxane (4 ml). The reaction mixture was then degassed with argon. After 5 minutes, Pd(PPh_3_)_4_ (0.118 g, 0.102 mmol) was added and the reaction mixture was allowed to stir at 110 °C for 12 h. After completion of the reaction as indicated by TLC, the solvent was removed under reduced pressure. The reaction was slowly basified with aqueous NaHCO_3_ and the obtained aqueous layer was extracted with ethyl acetate (100 mL × 3). The organic phase was washed with aqueous NaHCO_3_ (100 mL × 3) followed by brine (100 mL × 3) solution. The obtained organic layer was then dried over MgSO_4_, and solvent was evaporated. The crude product thus obtained was purified by silica gel (100–200 mesh) flash chromatography with hexanes/EtOAc (1:7) to afford **10** as a yellow solid (0.920 g, 63.01%); m.p. 188–190 °C; ^1^H NMR (400 MHz, DMSO-d_6_): δ 9.68 (s, 1H), 9.23 (s, 1H), 8.31 (s, 1H), 7.82 (d, *J* = 12.0 Hz, 2H), 7.76 (d, *J* = 12.0 Hz, 2H), 7.44 (d, *J* = 8.0 Hz, 2H), 7.18 (d, *J* = 8.0 Hz, 2H), 6.22 (s, 1H), 4.05 (q, *J* = 8.0, 16.0 Hz, 2H), 3.56 (s, 2H), 3.10 (s, 3H), 1.15 (t, *J* = 8.0 Hz, 3H); ^13^C NMR (100 MHz, DMSO-d6): δ 171.98, 161.09, 160.26, 158.08, 145.84, 138.96, 132.33, 130.13, 128.81, 128.54, 120.82, 118.80, 88.25, 60.80, 44.35, 40.15, 14.44; LC-MS (ESI): *m/z* 427.1897 [M + H]^+^.

### Synthesis of 2-(4-(6-(4-(methylsulfonyl)phenylamino)pyrimidin-4-ylamino)phenyl)acetic acid (11)

Compound **10** (0.500 g, 1.17 mmol) was added to THF/water (1:1, 8 mL) in a pressure reaction vessel. LiOH (0.084 g, 3.5 mmol) was then added and the reaction was heated to 100 °C for 5 h (or 15 min in microwave). TLC confirmed the complete consumption of compound **10**. Organic solvent was evaporated and the water solution was extracted with DCM (5 × 100 ml) and all DCM layers were discarded. Then, the reaction was acidified with 3 M HCl to pH ~4.0. The acidified aqueous solution was extracted (10 ml × 3) with 4:1 DCM/IPA. All extracts were combined, dried, and condensed to yield compound **11** as a white solid (0.400 g, 85.65%); m.p. 124–126 °C; ^1^H NMR (400 MHz, DMSO-d_6_): δ 12.24 (s, 1H), 9.73 (s, 1H), 9.27 (s, 1H), 8.32 (s, 1H), 7.86 (d, *J* = 8.0 Hz, 2H), 7.76 (d, *J* = 8.0 Hz, 2H), 7.45 (d, *J* = 8.0 Hz, 2H), 7.17 (d, *J* = 8.0 Hz, 2H), 6.24 (s, 1H), 3.49 (s, 2H), 3.11 (s, 3H); ^13^C NMR (100 MHz, DMSO-d_6_): δ 173.30, 161.18, 160.33, 158.08, 145.99, 139.00, 132.43, 130.16, 129.32, 128.55, 120.74, 118.65, 88.34, 44.47, 25.91; LC-MS (ESI): *m/z* 399.1773 [M + H]^+^.

### General procedure for synthesis of 4,6-diaminopyrimidine series of compounds 13a-13ak

The reaction of compound **11** (0.100 g, 0.251 mmol) or its structural analogs with 5-(tert-butyl-1-methyl-1H-pyrazol-5-amine (**12a**) (0.057 g, 0.376 mmol), in presence of EDC (0.120 g, 0.625 mmol), HOAt (0.034 g, 0.249 mmol), and DIPEA (0.053 mL, 0.410 mmol) in DMF (2 mL) was stirred at room temperature overnight. The completion of the reaction was monitored by TLC. After completion of the reaction, the organic layer was evaporated. The crude product was purified on silica gel column (mesh 100–200) using DCM: MeOH gradient (100: 0 to 70: 30 ratio of DCM: MeOH) mobile phase. The desired products **13a-13ak** were isolated in moderate to good yields.

#### 2-(4-(6-(4-(Methylsulfonyl)phenylamino)pyrimidin-4-ylamino)phenyl)-N-(3-tert-butyl-1-methyl-1H-pyrazol-5-yl)acetamide **(13a)**

White solid (0.054 g, 40.60%); m.p 134–136 °C; ^1^H NMR (400 MHz, DMSO-d_6_): δ 10.02 (s, 1H), 9.70 (s, 1H), 9.28 (s, 1H), 8.36 (s, 1H), 7.87 (d, *J* = 8.0 Hz, 2H), 7.79 (d, *J* = 8.0 Hz, 2H), 7.50 (d, *J* = 8.0 Hz, 2H), 7.28 (d, *J* = 8.0 Hz, 2H), 6.25 (s, 1H), 6.05 (s, 1H), 3.62 (s, 2H), 3.57 (s, 3H), 3.14 (s, 3H), 1.18 (s, 9H); ^13^C NMR (100 MHz, DMSO-d_6_): δ 169.58, 161.18, 160.31, 159.02, 158.09, 145.94, 139.03, 138.03, 136.71, 132.47, 129.89, 128.56, 120.80, 118.65, 95.38, 88.33, 44.46, 41.98, 35.70, 32.21, 30.74; LC-MS (ESI): *m/z* 534.2037 [M + H]^+^.

#### 2-(4-(6-(Methylamino)pyrimidin-4-ylamino)phenyl)-N-(3-tert-butyl-1-methyl-1H-pyrazol-5-yl)acetamide **(13b)**

Yellow solid (0.045 g, 29.60%); 194–196 °C; ^1^H NMR (400 MHz, DMSO-d_6_): δ 9.98 (s, 1H), 8.88 (s, 1H), 8.06 (s, 1H), 7.46 (d, *J* = 12.0 Hz, 2H), 7.20 (d, *J* = 12.0 Hz, 2H), 6.77 (d, *J* = 4.0 Hz, 1H), 6.03 (s, 1H), 5.69 (s, 1H), 3.58 (s, 2H), 3.55 (s, 3H), 2.71 (d, *J* = 8.0 Hz, 3H), 1.17 (s, 9H); ^13^C NMR (100 MHz, DMSO-d_6_): δ 169.71, 163.68, 160.56, 159.02, 157.96, 139.85, 136.72, 129.71, 128.86, 120.01, 95.43, 83.68, 41.99, 35.68, 32.21, 30.75, 27.84; LC-MS (ESI): *m/z* 394.2388 [M + H]^+^.

#### 2-(4-(6-(3-(Piperidin-1-yl)propylamino)pyrimidin-4-ylamino)phenyl)-N-(3-tert-butyl-1-methyl-1H-pyrazol-5-yl)acetamide **(13c)**

Sticky yellow solid (0.041 g, 30.14%); ^1^H NMR (400 MHz, acetone-d_6_): δ 9.19 (s, 1H), 8.08 (s, 2H), 7.52 (q, *J* = 4.0, 8.0 Hz, 2H), 7.29 (d, *J* = 8.0 Hz, 2H), 6.51 (s, 1H), 6.06 (s, 1H), 5.84 (s, 1H), 3.67 (s, 2H), 3.58 (s, 3H), 3.33 (t, *J* = 8.0 Hz, 2H), 2.58 (s, 6H), 1.84 (t, *J* = 8.0 Hz, 2H), 1.66 (t, *J* = 4.0Hz, 4H), 1.47 (t, *J* = 8.0 Hz, 2H), 1.20 (s, 9H); ^13^C NMR (100 MHz, DMSO-d_6_): δ 170.70, 169.70, 163.10, 159.02, 158.02, 139.82, 136.72, 129.71, 128.90, 120.05, 95.41, 88.03, 56.63, 54.38, 42.32, 41.97, 37.55, 35.68, 32.20, 30.74, 25.81, 24.37; LC-MS (ESI): *m/z* 505.2822 [M + H]^+^.

#### 2-(4-(6-(3-(Pyrrolidin-1-yl)propylamino)pyrimidin-4-ylamino)phenyl)-N-(3-tert-butyl-1-methyl-1H-pyrazol-5-yl)acetamide **(13d)**

Sticky pale yellow solid (0.039 g, 28.26%); ^1^H NMR (400 MHz, DMSO-d_6_): δ 9.96 (s, 1H), 8.82 (s, 1H), 8.03 (s, 1H), 7.42 (d, *J* = 8.0 Hz, 2H), 7.18 (d, *J* = 8.0 Hz, 2H), 6.83 (s, 1H), 6.00 (s, 1H), 5.70 (s, 1H), 3.55 (s, 2H), 3.53 (s, 3H), 3.17 (s, 2H), 2.41 (s, 6H), 1.64 (s, 6H), 1.15 (s, 9H); ^13^C NMR (100 MHz, DMSO-d_6_): δ 169.68, 163.13, 159.01, 158.02, 139.84, 136.72, 130.40, 129.71, 128.88, 120.04, 95.40, 90.18,54.02, 53.75, 45.58, 41.98, 35.69, 32.21, 30.75, 28.62, 23.50; LC-MS (ESI): *m/z* 491.2592 [M + H]^+^.

#### 2-(4-(6-(4-(Methylsulfonyl)phenylamino)pyrimidin-4-yloxy)phenyl)-N-(3-tert-butyl-1-methyl-1H-pyrazol-5-yl)acetamide **(13e)**

Sticky yellow solid (0.042 g, 31.57%); ^1^H NMR (400 MHz, DMSO-d_6_): δ 10.07 (d, *J* = 4.0 Hz, 2H), 8.43 (s, 1H), 7.87 (d, *J* = 8.0Hz, 2H), 7.80 (d, *J* = 8.0 Hz, 2H), 7.39 (d, *J* = 8.0 Hz, 2H), 7.16 (d, *J* = 8.0 Hz, 2H), 6.19 (s, 1H), 6.04 (s, 1H), 3.69 (s, 2H), 3.57 (s, 3H), 3.12 (s, 3H), 1.16 (s, 9H); ^13^C NMR (100 MHz, DMSO-d_6_): δ 169.97, 169.23, 162.40, 159.07, 158.57, 151.59, 145.05, 136.66, 133.53, 133.41, 131.11, 128.65, 121.94, 119.17, 95.30, 90.73, 44.37, 40.55, 35.74, 32.22, 30.75; LC-MS (ESI): *m/z* 535.2312 [M + H]^+^.

#### 2-(4-(6-(3-(Trifluoromethyl)phenylamino)pyrimidin-4-yloxy)phenyl)-N-(3-tert-butyl-1-methyl-1H-pyrazol-5-yl)acetamide **(13f)**

Yellow solid (0.046 g, 34.32%); m.p. 140–142 °C; ^1^H NMR (400 MHz, acetone-d_6_): δ 9.27 (s, 1H), 9.07 (s, 1H), 8.38 (s, 1H), 8.21 (s, 1H), 7.88 (d, *J* = 8.0 Hz, 1H), 7.51 (t, *J* = 8.0 Hz, 1H), 7.44 (d, *J* = 8.0 Hz, 2H), 7.32 (d, *J* = 8.0 Hz, 1H), 7.14 (d, *J* = 8.0 Hz, 2H), 6.17 (s, 1H), 6.11 (s, 1H), 3.77 (s, 2H), 3.60 (s, 3H), 1.21 (s, 9H); ^13^C NMR (100 MHz, acetone-d_6_): δ 170.92, 169.63, 163.50, 160.02, 159.03, 152.82, 141.87, 137.03, 133.64, 131.47, 131.19, 130.55, 126.65, 123.89, 122.40, 119.45, 116.64, 95.96, 90.64, 42.76, 35.65, 32.69, 30.79; LC-MS (ESI): *m/z* 525.2342 [M + H]^+^.

#### 2-(4-(6-(4-(Methylsulfonyl)phenylamino)pyrimidin-4-ylamino)phenyl)-N-(3-tert-butyl-1-(4-fluorophenyl)-1H-pyrazol-5-yl)acetamide **(13g)**

Pale yellow solid (0.043 g, 34.95%); m.p. 168–170 °C; ^1^H NMR (400 MHz, acetone-d_6_): δ 8.98 (s, 1H), 8.89 (s, 1H), 8.47 (s, 1H), 8.40 (s, 1H), 7.95 (d, *J* = 8.0 Hz, 2H), 7.83 (d, *J* = 8.0 Hz, 2H), 7.54 (d, *J* = 8.0 Hz, 2H), 7.44–7.41 (m, 2H), 7.29 (d, *J* = 8.0 Hz, 2H), 7.18 (t, *J* = 8.0 Hz, 2H), 6.41 (s, 1H), 6.31 (s, 1H), 3.65 (s, 2H), 3.07 (s, 3H), 1.29 (s, 9H); ^13^C NMR (100 MHz, acetone-d_6_): δ 168.81, 162.48,(d, ^1^*J*_CF_ = 244 Hz), 161.37, 161.27, 157.89, 145.65, 145.57, 138.97, 133.16, 129.80, 128.36, 125.88, 125.89, 120.97, 120.82, 118.53, 118.44, 115.84, (d, ^2^*J*_CF_ = 23 Hz), 97.33, 87.68, 43.79, 42.29, 32.06, 28.36; LC-MS (ESI): *m/z* 614.2142 [M + H]^+^.

#### 2-(4-(6-(4-(Methylsulfonyl)phenylamino)pyrimidin-4-ylamino)phenyl)-N-(3-tert-butyl-1-p-tolyl-1H-pyrazol-5-yl)acetamide **(13h)**

Yellow solid (0.049 g, 32.02%); m.p. 155–157 °C; ^1^H NMR (400 MHz, acetone-d_6_): δ 8.91 (s, 1H), 8.83 (s, 1H), 8.48 (s, 1H), 8.41 (s, 1H), 7.95 (d, *J* = 8.0 Hz, 2H), 7.83 (d, *J* = 8.0 Hz, 2H), 7.54 (d, *J* = 8.0 Hz, 2H), 7.29–7.19 (m, 6H), 6.44 (s, 1H), 6.32 (s, 1H), 3.65 (s, 2H), 3.06 (s, 3H), 2.31 (s, 3H), 1.29 (s, 9H); ^13^C NMR (100 MHz, acetone-d_6_): δ 168.47, 161.36, 160.97, 160.49, 157.90, 145.65, 139.03, 136.79, 136.45, 135.99, 133.16, 129.85, 129.59, 128.36, 123.74, 120.87, 120.72, 118.53, 118.44, 96.43, 87.68, 43.79, 42.32, 32.04, 20.06; LC-MS (ESI): *m/z* 610.2609 [M + H]^+^.

#### 2-(4-(6-Morpholinopyrimidin-4-ylamino)phenyl)-N-(3-tert-butyl-1-methyl-1H-pyrazol-5-yl)acetamide (**13i**)

Sticky white solid (0.040 g, 28.16%); ^1^H NMR (400 MHz, DMSO-d_6_): δ 9.99 (s, 1H), 9.07 (s, 1H), 8.20 (s, 1H), 7.52 (d, *J* = 8.0 Hz, 2H), 7.23 (d, *J* = 8.0 Hz, 2H), 6.04 (s, 1H), 5.94 (s, 1H), 3.66 (t, *J* = 4.0 Hz, 4H), 3.59 (s, 2H), 3.56 (s, 3H), 3.43 (t, *J* = 4.0 Hz, 4H), 1.18 (s, 9H); ^13^C NMR (100 MHz, DMSO-d_6_): δ 169.67, 162.89, 161.43, 159.03, 157.75, 139.58, 136.72, 129.76, 129.13, 120.03, 95.42, 84.44, 66.21, 44.36, 41.99, 35.69, 32.21, 30.74; LC-MS (ESI): *m/z* 450.3030 [M + H]^+^.

#### 2-(4-(6-(4-Methylpiperazin-1-yl)pyrimidin-4-ylamino)phenyl)-N-(3-tert-butyl-1-(4-bromophenyl)-1H-pyrazol-5-yl)acetamide **(13j)**

Colourless solid (0.056 g, 30.43%); m.p. 110–112 °C; ^1^H NMR (400 MHz, acetone-d_6_): δ 8.95 (s, 1H), 8.21 (s, 2H), 7.59 (d, *J* = 8.0 Hz, 2H), 7.54 (d, *J* = 12.0 Hz, 2H), 7.33 (d, *J* = 12.0 Hz, 2H), 7.23 (d, *J* = 12.0 Hz, 2H), 6.39 (s, 1H), 6.01 (s, 1H), 3.62 (s, 2H), 3.57–3.52 (m, 4H), 2.38 (t, *J* = 8.0 Hz, 4H), 2.23 (s, 3H), 1.27 (s, 9H); ^13^C NMR (100 MHz, acetone-d_6_): δ 168.83, 162.84, 161.60, 161.47, 157.55, 139.82, 138.34, 136.20, 132.06, 129.64, 128.38, 125.20, 120.09, 119.72, 97.80, 83.91, 54.40, 45.38, 43.65, 42.40, 32.08, 27.70; LC-MS (ESI): *m/z* 605.1965 [M + H]^+^.

#### 2-(4-(6-(4-(Methylsulfonyl)phenylamino)pyrimidin-4-ylamino)phenyl)-N-(5-tert-butylisoxazol-3-yl)acetamide (**13k**)

Yellow solid (0.055 g, 42.30%); m.p. 168–170 °C; ^1^H NMR (400 MHz, DMSO-d_6_): δ 11.15 (s, 1H), 9.68 (s, 1H), 9.26 (s, 1H), 8.34 (s, 1H), 7.86 (d, *J* = 8.0 Hz, 2H), 7.78 (d, *J* = 8.0 Hz, 2H), 7.46 (d, *J* = 8.0 Hz, 2H), 7.25 (d, *J* = 8.0 Hz, 2H), 6.55 (s, 1H), 6.23 (s, 1H), 3.60 (s, 2H), 3.12 (s, 3H), 1.26 (s, 9H); ^13^C NMR (100 MHz, DMSO-d_6_): δ 180.82, 170.02, 161.14, 160.30, 158.35, 158.06, 145.91, 139.06, 132.45, 129.90, 129.58, 128.53, 120.85, 118.82, 93.47, 88.26, 44.43, 42.32, 32.89, 28.71; LC-MS (ESI): *m/z* 521.1169 [M + H]^+^.

#### 2-(4-(6-(3-(Trifluoromethyl)phenylamino)pyrimidin-4-yloxy)phenyl)-N-(5-tert-butylisoxazol-3-yl)acetamide **(13l)**

Colorless solid (0.050 g, 38.16%); m.p. 120–122 °C; ^1^H NMR (400 MHz, acetone-d_6_): δ 10.16 (s, 1H), 9.06 (s, 1H), 8.37 (s, 1H), 8.20 (s, 1H), 7.89 (d, *J* = 8.0 Hz, 1H), 7.52 (t, *J* = 8.0 Hz, 1H), 7.46 (d, *J* = 8.0 Hz, 2H), 7.32 (d, *J* = 8.0 Hz, 1H), 7.14 (d, *J* = 8.0 Hz, 2H), 6.65 (s, 1H), 6.18 (s, 1H), 3.82 (s, 2H), 1.31 (s, 9H); ^13^C NMR (100 MHz, acetone-d_6_): δ 180.77, 170.03, 162.61, 158.13, 158.04, 152.00, 140.98, 132.44, 130.62, 129.66, 123.01, 121.52, 118.60, 118.56, 118.52, 116.00, 115.96, 92.95, 89.73, 42.25, 32.55, 27.93; LC-MS (ESI): *m/z* 512.2689 [M + H]^+^.

#### 2-(4-(6-(3-(Trifluoromethyl)phenylamino)pyrimidin-4-ylamino)phenyl)-N-(5-tert-butylisoxazol-3-yl)acetamide **(13m)**

White solid (0.051 g, 38.93%); m.p 254–256 °C; ^1^H NMR (400 MHz, acetone-d_6_): δ 10.04 (s, 1H), 8.68 (s, 1H), 8.33 (d, *J* = 8.0 Hz, 2H), 8.18 (s, 1H), 7.86 (d, *J* = 8.0 Hz, 1H), 7.52–7.46 (m, 3H), 7.34 (d, *J* = 8.0 Hz, 2H), 7.26 (d, *J* = 8.0 Hz, 1H), 6.64 (s, 1H), 6.24 (s, 1H), 3.75 (s, 2H), 1.30 (s, 9H); ^13^C NMR (100 MHz, acetone-d6): δ 180.69, 169.22, 161.29, 160.84, 158.07, 157.92, 141.71, 138.95, 129.73, 129.51, 122.63, 120.93, 120.78, 117.75, 117.71, 117.67, 115.62, 92.91, 86.58, 42.41, 32.53, 27.92; LC-MS (ESI): *m/z* 511.1696 [M + H]^+^.

#### 2-(4-(6-(3-(Pyrrolidin-1-yl)propylamino)pyrimidin-4-ylamino)phenyl)-N-(5-tert-butylisoxazol-3-yl)acetamide **(13n)**

White solid (0.049 g, 36.56%); m.p. 154–156 °C; ^1^H NMR (400 MHz, acetone-d_6_): δ 10.02 (s, 1H), 8.09 (s, 1H), 8.04 (s, 1H), 7.54–7.51 (m, 2H), 7.31 (d, *J* = 8.0 Hz, 2H), 6.64 (s, 1H), 6.28 (s, 1H), 5.83 (s, 1H), 3.73 (s, 2H), 3.33 (s, 2H), 2.53 (t, *J* = 8.0 Hz, 2H), 2.47 (s, 4H), 1.77 (t, *J* = 8.0 Hz, 2H), 1.73–1.70 (m, 4H), 1.31 (s, 9H); ^13^C NMR (100 MHz, acetone-d_6_): δ169.83, 169.26, 163.42, 157.88, 139.71, 130.29, 129.55, 128.69, 120.18, 120.04, 92.91, 89.97, 53.74, 53.68, 42.42, 39.56, 37.71, 32.52, 28.92, 23.21; LC-MS (ESI): *m/z* 478.1989 [M + H]^+^.

#### 2-(4-(6-(4-Bromophenoxy)pyrimidin-4-ylamino)phenyl)-N-(5-tert-butylisoxazol-3-yl)acetamide **(13o)**

White solid (0.046 g, 35.38%); m.p. 188–190 °C; ^1^H NMR (400 MHz, acetone-d_6_): δ 10.06 (s, 1H), 8.77 (s, 1H), 8.29 (s, 1H), 7.60 (d, *J* = 8.0 Hz, 4H), 7.37 (d, *J* = 8.0 Hz, 2 H), 7.16 (d, *J* = 8.0 Hz, 2H), 6.65 (s, 1H), 6.22 (s, 1H), 3.77 (s, 2H), 1.31 (s, 9H); ^13^C NMR (100 MHz, acetone-d_6_): δ; 180.68, 169.42, 163.08, 158.07, 152.52, 138.54, 132.48, 129.98, 129.73, 129.45, 123.77, 120.63, 120.49, 117.38, 92.93, 88.88, 42.40, 32.53, 27.93; LC-MS (ESI): *m/z* 522.1923 [M + H]^+^.

#### 2-(4-(6-Morpholinopyrimidin-4-ylamino)phenyl)-N-(5-tert-butylisoxazol-3-yl)acetamide **(13p)**

White solid (0.054 g, 39.13%); m.p. 218–220 °C; ^1^H NMR (400 MHz, DMSO-d_6_): δ 11.13 (s, 1H), 9.07 (s, 1H), 8.19 (s, 1H), 7.49 (d, *J* = 12.0Hz, 2H), 7.21 (d, *J* = 8.0 Hz, 2H), 6.56 (s, 1H), 5.94 (s, 1H), 3.66 (t, *J* = 4.0Hz, 4H), 3.58 (s, 2H), 3.43 (t, *J* = 4.0 Hz, 4H), 1.26 (s, 9H); ^13^C NMR (100 MHz, DMSO-d_6_): δ 180.82, 170.11, 162.89, 161.39, 158.38, 157.74, 139.63, 129.80, 128.79, 120.04, 93.51, 84.41, 66.20, 44.35, 42.33, 32.90, 28.74; LC-MS (ESI): *m/z* 437.1940 [M + H]^+^.

#### 2-(4-(6-(Dimethylamino)pyrimidin-4-yloxy)phenyl)-N-(5-tert-butylisoxazol-3-yl)acetamide **(13q)**

Colourless solid (0.045 g, 31.25%); m.p. 170–172 °C; ^1^H NMR (400 MHz, acetone-d_6_): δ 10.14 (s, 1H), 8.11 (s, 1H), 7.40 (d, *J* = 8.0 Hz, 2H), 7.07 (d, *J* = 8.0 Hz, 2H), 6.65 (s, 1H), 5.98 (s, 1H), 3.79 (s, 2H), 3.07 (s, 6H), 1.30 (s, 9H); ^13^C NMR (100 MHz, acetone-d_6_): δ 180.72, 169.80, 169.09, 164.53, 158.06, 157.26, 152.50, 131.68, 130.23, 121.42, 92.95, 85.52, 42.32, 36.36, 32.55, 27.95; LC-MS (ESI): *m/z* 396.2055 [M + H]^+^.

#### 2-(4-(6-(Methylamino)pyrimidin-4-ylamino)phenyl)-N-(3-(trifluoromethyl)phenyl) acetamide **(13r)**

White solid (0.050 g, 32.25%); m.p. 216–218 °C; ^1^H NMR (400 MHz, acetone-d_6_): δ 9.60 (s, 1H), 8.17 (s, 1H), 8.08 (s, 1H), 7.82 (d, *J* = 8.0 Hz, 1H), 7.53–7.48 (m, 4H), 7.36 (d, *J* = 8.0Hz, 1 H), 7.29 (d, *J* = 8.0 Hz, 2H), 5.98 (s, 1H), 5.78 (s, 1H), 3.67 (s, 2H), 2.82 (d, *J* = 4.0Hz, 3H); ^13^C NMR (100 MHz, acetone-d_6_): δ 169.78, 164.00, 157.78, 140.25, 139.62, 129.63, 129.52, 128.97, 122.47, 122.38, 120.17, 120.03, 119.56, 119.52, 115.48, 82.71, 43.19, 27.19; LC-MS (ESI): *m/z* 402.2207 [M +H]^+^.

#### 2-(4-(6-(4-(Methylsulfonyl)phenylamino)pyrimidin-4-ylamino)phenyl)-N-(3-(trifluoromethyl)phenyl)acetamide **(13 s)**

White solid (0.045 g, 33.33%); m.p. 225–227 °C; ^1^H NMR (400 MHz, DMSO-d_6_): δ 10.49 (s, 1H), 9.69 (s, 1H), 9.27 (s, 1H), 8.34 (s, 1H), 8.10 (s, 1H), 7.86 (d, *J* = 8.0Hz, 2H), 7.78 (d, *J* = 8.0Hz, 3H), 7.53 (t, *J* = 8.0Hz, 1H), 7.48 (d, *J* = 8.0Hz, 2H), 7.38 (d, *J* = 8.0Hz, 1H), 7.28 (d, *J* = 8.0Hz, 2H), 6.23 (s, 1H), 3.62 (s, 2H), 3.12 (s, 3H); ^13^C NMR (100 MHz, DMSO-d_6_): δ 170.38, 161.19, 160.33, 158.10, 145.95, 140.40, 139.07, 132.47, 130.43, 129.96, 129.88, 129.70, 128.56, 123.00, 120.87, 119.93, 118.65, 115.50, 115.46, 88.30, 44.46, 43.16; LC-MS (ESI): *m/z* 542.1709 [M + H]^+^.

#### 2-(4-(6-(3-(Pyrrolidin-1-yl)propylamino)pyrimidin-4-ylamino)phenyl)-N-(4-(trifluoromethoxy)phenyl)acetamide **(13t)**

White solid (0.044 g, 30.55%); m.p. 212–214 °C; ^1^H NMR (400 MHz, acetone-d_6_): δ 9.52 (s, 1H), 8.06 (d, *J* = 8.0Hz, 2H), 7.77 (d, *J* = 8.0Hz, 2H), 7.51–7.48 (m, 2H), 7.28 (d, *J* = 8.0Hz, 2H), 7.23 (d, *J* = 12.0Hz, 2H), 6.27 (d, *J* = 4.0Hz, 1H), 5.80 (s, 1H), 3.64 (s, 2H), 3.31 (s, 2H), 2.50 (t, *J* = 8.0Hz, 2H), 2.44 (t, *J* = 8.0Hz, 4H), 1.74 (t, *J* = 4.0Hz, 2H), 1.70–1.67 (m, 4H); ^13^C NMR (100 MHz, acetone-d_6_): δ 169.36, 163.38, 157.86, 144.17, 139.50, 138.58, 129.50, 121.46, 120.39, 120.30, 120.23, 120.09, 119.29, 82.98, 53.70, 53.76, 43.16, 43.11, 39.48,23.20; LC-MS (ESI): *m/z* 515.1573 [M + H]^+^.

#### 2-(4-(6-(2,3-Dihydrobenzo[b][1,4]dioxin-5-ylamino)pyrimidin-4-ylamino)phenyl)-N-(4-(trifluoromethoxy)phenyl)acetamide **(13 u)**

White solid (0.047 g, 33.09%); m.p 275–277 °C; ^1^H NMR (400 MHz, acetone-d_6_): δ 9.69 (s, 1H), 8.26 (s, 1H), 8.21 (s, 1H), 8.08 (s, 1H), 7.81 (d, *J* = 8.0Hz, 2H), 7.54–7.47 (m, 2H), 7.32 (d, *J* = 8.0Hz, 2H), 7.25 (d, *J* = 8.0Hz, 2H), 7.18–7.16 (m, 1H), 6.93–6.89 (m, 1H), 6.76 (d, *J* = 8.0Hz, 1H), 6.13 (s, 1H), 4.26–4.22 (m, 4H), 3.68 (s, 2H); ^13^C NMR (100 MHz, acetone-d_6_): δ 169.47, 161.49, 161.10, 157.95, 143.55, 139.57, 139.24, 138.74, 133.86, 129.55, 122.23, 121.44, 120.40, 120.37, 120.31, 120.22, 116.90, 114.34, 110.32, 84.88, 64.39, 64.13, 43.13; LC-MS (ESI): *m/z* 538.2409 [M +H]^+^.

#### 2-(4-(6-(methylamino)pyrimidin-4-ylamino)phenyl)-N-(4-(methylsulfonyl)phenyl) acetamide **(13 v)**

Yellow solid (0.044 g, 27.67%); m.p. 220–222 °C; ^1^H NMR (400 MHz, acetone-d_6_): δ 9.79 (s, 1H), 8.16 (s, 1H), 8.08 (s, 1H), 7.89 (d, *J* = 8.0Hz, 2H), 7.84 (d, *J* = 8.0Hz, 2H), 7.51 (d, *J* = 8.0Hz, 2H), 7.29 (d, *J* = 8.0Hz, 2H), 6.05 (d, *J* = 8.0Hz, 1H), 5.78 (s, 1H), 3.69 (s, 2H), 3.05 (s, 3H), 2.82 (d, *J* = 4.0Hz, 3H); ^13^C NMR (100 MHz, acetone-d_6_): δ 170.04, 163.99, 157.77, 144.00, 139.61, 135.33, 129.57, 128.89, 128.35, 120.25, 118.96, 118.88, 82.63, 43.65, 43.18, 27.23; LC-MS (ESI): *m/z* 412.1931 [M +H]^+^.

#### N-(4-Fluorobenzyl)-2-(4-(6-(3-(pyrrolidin-1-yl)propylamino)pyrimidin-4-yloxy)phenyl)acetamide **(13w)**

Sticky colourless solid (0.042 g, 32.30%); ^1^H NMR (400 MHz, DMSO-d_6_): δ 8.80 (s, 1H), 8.47 (t, *J* = 8.0Hz, 1H), 8.03 (s, 1H), 7.38 (d, *J* = 8.0Hz, 1H), 7.24 (dd, *J* = 4.0, 8.0Hz, 2H), 7.13–7.07 (m, 4H), 6.85 (t, *J* = 8.0Hz, 1H), 5.71 (s, 1H), 4.20 (d, *J* = 4.0Hz, 2H), 3.36 (s, 2H), 3.18 (s, 3H), 2.54 (s, 5H), 1.68–1.64 (m, 6H); ^13^C NMR (100 MHz, DMSO-d_6_): δ 170.40, 162.66, (d, ^1^*J*_CF_ = 241Hz), 159.90, 157.59, 139.12, 135.72, 129.28, 129.20,129.19, 129.10, 119.64, 115.06, (d, ^2^*J*_CF_ = 21Hz), 83.66, 53.50, 53.06, 41.71, 41.46, 38.53, 27.69, 22.98; LC-MS (ESI): *m/z* 464.1752 [M + H]^+^.

#### 2-(4-(6-(Methylamino)pyrimidin-4-yloxy)phenyl)-N-(4-tert-butylphenyl)acetamide **(13x)**

White solid (0.046 g, 30.66%); m.p. 208–210 °C; ^1^H NMR (400 MHz, DMSO-d_6_): δ 10.12 (s, 1H), 7.62 (t, *J* = 8.0Hz, 2H), 7.56–7.51 (m, 3H), 7.37 (d, *J* = 8.0Hz, 2H), 7.31 (d, *J* = 12.0Hz, 3H), 7.08 (d, *J* = 8.0Hz, 2H), 3.63 (s, 2H), 2.76 (s, 3H), 1.24 (s, 9H); ^13^C NMR (100 MHz, DMSO-d_6_): δ 169.25, 151.95, 145.97, 137.04, 132.47, 131.95, 130.73, 129.24, 129.12, 125.73, 121.77, 119.30, 85.70, 43.01, 42.12, 34.41, 31.61; LC-MS (ESI): *m/z* 391.2869 [M +H]^+^.

#### 2-(4-(6-(3-(Piperidin-1-yl)propylamino)pyrimidin-4-ylamino)phenyl)-N-(4-(trifluoromethoxy)phenyl)acetamide **(13y)**

Sticky pale yellow solid (0.041 g, 28.67%); ^1^H NMR (400 MHz, DMSO-d_6_): δ 10.40 (s, 1H), 8.89 (s, 1H), 8.06 (s, 1H), 7.69 (d, *J* = 8.0Hz, 2H), 7.40 (d, *J* = 8.0Hz, 2H), 7.27 (d, *J* = 8.0Hz, 2H), 7.20 (d, *J* = 8.0Hz, 2H), 6.97 (s, 1H), 5.75 (s, 1H), 3.55 (s, 2H), 2.95 (s, 5H), 1.85 (s, 3H), 1.69 (s, 5H), 1.49 (s, 3H); ^13^C NMR (100 MHz, DMSO-d_6_): δ 170.09, 162.99, 160.48, 158.03, 143.86, 139.62, 138.89, 129.78, 129.32, 122.03, 120.80, 120.30, 119.27, 83.97, 54.45, 52.59, 43.05, 31.12, 24.26, 23.13, 21.92; LC-MS (ESI): *m/z* 529.1870 [M + H]^+^.

#### 2-(4-(6-(Methylamino)pyrimidin-4-ylamino)phenyl)-N-(4-(trifluoromethoxy)phenyl) acetamide **(13z)**

White solid (0.052 g, 32.29%); m.p 220–222 °C; ^1^H NMR (400 MHz, acetone-d_6_): δ 9.47 (s, 1H), 8.08 (s, 2H), 7.76 (d, *J* = 8.0Hz, 2H), 7.51 (d, *J* = 8.0Hz, 2H), 7.25 (q, *J* = 8.0Hz, 4H), 5.99 (s, 1H), 5.78 (s, 1H), 3.64 (s, 2H), 2.82 (d, *J* = 4.0Hz, 3H); ^13^C NMR (100 MHz, acetone-d_6_): δ 169.41, 164.01, 157.79, 144.18, 139.58, 138.65, 129.49, 129.14, 121.47, 120.38, 120.29, 120.18, 120.04, 82.71, 43.16, 27.19; LC-MS (ESI): *m/z* 418.2319 [M + H]^+^.

#### 2-(4-(6-(4-(Methylsulfonyl)phenylamino)pyrimidin-4-yloxy)phenyl)-N-(3-fluorophenyl)acetamide **(13aa)**

Brown solid (0.038 g, 30.89%); m.p. 228–230 °C; ^1^H NMR (400 MHz, acetone-d_6_): δ 9.67 (s, 1H), 9.29 (s, 1H), 8.40 (s, 1H), 7.97 (d, *J* = 8.0Hz, 2H), 7.84 (d, *J* = 8.0Hz, 2H), 7.70 (d, *J* = 12.0Hz, 1H), 7.45 (d, *J* = 8.0Hz, 2H), 7.34–7.27 (m, 2H), 7.13 (d, *J* = 8.0Hz, 2H), 6.80 (t, *J* = 8.0Hz, 1H), 6.24 (s, 1H), 3.75 (s, 2H), 3.06 (s, 3H), ^13^C NMR (100 MHz, acetone-d_6_): δ 170.13, 169.25, 169.16, 163.99 (d, ^1^*J*_CF_ = 240Hz), 162.35, 158.09, 151.84, 144.93, 144.84, 132.91, 130.61, 130.16 (d, ^3^*J*_CF_ = 9.0Hz), 128.39, 121.47, 118.95, 114.72, 109.78, (d, ^2^*J*_CF_ = 22Hz), 106.28, (d, ^2^*J*_CF_ = 26Hz), 90.52, 43.73, 42.98; LC-MS (ESI): *m/z* 493.1870 [M +H]^+^.

#### 2-(4-(6-(3-(Pyrrolidin-1-yl)propylamino)pyrimidin-4-ylamino)phenyl)-N-(3-(trifluoromethyl)phenyl)acetamide **(13ab)**

Sticky pale yellow solid (0.045 g, 32.14%); ^1^H NMR (400 MHz, DMSO-d_6_): δ 10.51 (s, 1H), 8.84 (s, 1H), 8.08 (s, 1H), 8.03 (s, 1H), 7.76 (d, *J* = 8.0Hz, 1H), 7.51 (t, *J* = 8.0Hz, 1H), 7.41 (d, *J* = 8.0Hz, 2H), 7.35 (d, *J* = 8.0Hz, 1H), 7.20 (d, *J* = 8.0Hz, 2H), 6.85 (s, 1H), 5.72 (s, 1H), 3.57 (s, 2H), 3.18 (s, 2H), 2.54 (s, 6H), 1.64–1.59 (m, 6H); ^13^C NMR (100 MHz, DMSO-d_6_): δ 167.16, 159.79, 154.71, 137.13, 136.51, 127.08, 126.67, 126.47, 125.60, 122.56, 119.85, 119.67, 116.84, 116.56, 112.18, 89.99, 50.59, 50.14, 39.82, 35.64, 24.75, 20.09; LC-MS (ESI): *m/z* 499.1751 [M +H]^+^.

#### 2-(4-(6-(4-(Methylsulfonyl)phenylamino)pyrimidin-4-ylamino)phenyl)-N-(3-fluorophenyl)acetamide **(13ac)**

Sticky white solid (0.047 g, 38.21%); ^1^H NMR (400 MHz, DMSO-d_6_): δ 10.38 (s, 1H), 9.70 (s, 1H), 9.27 (s, 1H), 8.34 (s, 1H), 7.87 (d, *J* = 8.0Hz, 2H), 7.78 (d, *J* = 12.0Hz, 2H), 7.60 (d, *J* = 12.0Hz, 1H), 7.48 (d, *J* = 8.0Hz, 2H), 7.34–7.27 (m, 4H), 6.85 (t, *J* = 8.0Hz, 1H), 6.25 (s, 1H), 3.60 (s, 2H), 3.13 (s, 3H); ^13^C NMR (100 MHz, DMSO-d_6_): δ 170.13, 163.72, 161.32, 161.17, 160.31 (d, ^1^*J*_CF_ = 223Hz), 145.94, 141.42, 139.00, 132.43, 130.81, 130.00, 129.92, 128.54, 120.87, 115.21, 115.19, 110.14 (d, ^2^*J*_CF_ = 21Hz), 106.36 (d, ^2^*J*_CF_ = 26Hz), 88.26, 44.44, 43.13; LC-MS (ESI): *m/z* 492.1703 [M +H]^+^.

#### 2-(4-(6-(Methylamino)pyrimidin-4-yloxy)phenyl)-N-(4-fluorophenyl)acetamide **(13ad)**

White solid (0.048 g, 35.55%); m.p. 124–126 °C; ^1^H NMR (400 MHz, acetone-d_6_): δ 9.43 (s, 1H), 8.09 (s, 1H), 7.72–7.66 (m, 2H), 7.41 (d, *J* = 8.0Hz, 2H), 7.10–7.04 (m, 4H), 6.47 (s, 1H), 5.83 (s, 1H), 3.71 (s, 2H), 2.90 (d, *J* = 8.0Hz, 3H); ^13^C NMR (100 MHz, acetone-d_6_): δ 168.78, 159.82 (d, ^1^*J*_CF_ = 241Hz) 157.94, 152.27, 135.79, 132.45, 130.22, 121.37, 120.94, 120.86, 120.77, 115.12 (d, ^2^*J*_CF_ = 23Hz), 83.01, 42.97, 27.22; LC-MS (ESI): *m/z* 353.1776 [M +H]^+^.

#### 2-(4-(6-(4-(Methylsulfonyl)phenylamino)pyrimidin-4-ylamino)phenyl)-N-cyclopropylacetamide **(13ae)**

Yellow solid (0.036 g, 33.02%); m.p. 110–112 °C; ^1^H NMR (400 MHz, DMSO-d_6_): δ 9.69 (s, 1H), 9.22 (s, 1H), 8.34 (s, 1H), 8.08 (s, 1H), 7.86 (d, *J* = 12.0Hz, 2H), 7.78 (d, *J* = 12.0Hz, 2H), 7.43 (d, *J* = 8.0Hz, 2H), 7.17 (d, *J* = 8.0Hz, 2H), 6.23 (s, 1H), 3.29 (s, 2H), 3.13 (s, 3H), 2.62–2.58 (m, 1H), 0.62–0.57 (m, 2H), 0.40–0.36 (m, 2H); ^13^C NMR (100 MHz, DMSO-d_6_): δ 171.92, 161.41, 160.50, 158.28, 146.14, 138.85, 132.62, 131.07, 129.90, 128.73, 121.07, 118.81, 88.32, 44.64, 42.22, 23.00, 6.28; LC-MS (ESI): *m/z* 438.1555 [M + H]^+^.

#### 2-(4-(6-(3-(Trifluoromethyl)phenylamino)pyrimidin-4-ylamino)phenyl)-N-cyclopropylacetamide **(13af)**

Sticky pale yellow solid (0.040 g, 36.36%); ^1^H NMR (400 MHz, DMSO-d_6_): δ 9.46 (s, 1H), 9.14 (s, 1H), 8.29 (s, 1H), 8.09–8.06 (m, 2H), 7.80 (d, *J* = 8.0Hz, 1H), 7.46 (t, *J* = 8.0Hz, 1H), 7.40 (d, *J* = 8.0Hz, 2H), 7.22 (d, *J* = 8.0Hz, 1H), 7.14 (d, *J* = 8.0Hz, 2H), 6.13 (s, 1H), 3.26 (s, 2H), 2.61–2.54 (m, 1H), 0.59–0.55 (m, 2H), 0.37–0.33 (m, 2H); ^13^C NMR (100 MHz, DMSO-d_6_): δ 171.76, 161.10, 160.60, 158.12, 142.03, 138.79, 130.75, 130.18, 129.99, 129.69, 123.36, 122.87, 120.79, 117.75, 115.31, 87.30, 42.05, 22.83, 6.11; LC-MS (ESI): *m/z* 428.1801 [M +H]^+^.

#### 2-(4-(6-(2,3-Dihydrobenzo[b][1,4]dioxin-5-ylamino)pyrimidin-4-ylamino)phenyl)-N-cyclopropylacetamide **(13ag)**

White solid (0.041 g, 37.27%); m.p. 234–226 °C; ^1^H NMR (400 MHz, acetone-d_6_): δ 8.21 (s, 1H), 8.19 (s, 1H), 8.03 (s, 1H), 7.48 (dd, *J* = 4.0, 8.0Hz, 2H), 7.22 (d, *J* = 8.0Hz, 3H), 7.17–7.15 (m, 1H), 6.92–6.89 (m, 1H), 6.77 (d, *J* = 8.0Hz, 1H), 6.10 (s, 1H), 4.27–4.23 (m, 4H), 3.37 (s, 2H), 2.73–2.66 (m, 1H), 0.65–0.60 (m, 2H), 0.44–0.40 (m, 2H); ^13^C NMR (100 MHz, acetone-d_6_ + MeOD): δ 172.45, 161.38, 161.01, 157.88, 143.57, 139.71, 138.81, 133.53, 130.12, 129.34, 120.39, 116.92, 114.47, 110.45, 84.60, 64.37, 64.13, 41.96, 22.27, 5.37; LC-MS (ESI): *m/z* 418.1931 [M +H]^+^.

#### 2-(4-(6-(4-Methylpiperazin-1-yl)pyrimidin-4-ylamino)phenyl)-N-cyclopropyl acetamide **(13ah)**

Sticky colourless solid (0.042 g, 38.18%); ^1^H NMR (400 MHz, DMSO-d_6_): δ 8.95 (s, 1H), 8.13 (s, 1H), 8.04 (s, 1H), 7.42 (d, *J* = 8.0 Hz, 2H), 7.09 (d, *J* = 8.0HHz, 2H), 5.90 (s, 1H), 3.43 (t, *J* = 4.0Hz, 4H), 3.32 (s, 2H), 2.60–2.53 (m, 1H), 2.32 (t, *J* = 4.0Hz, 4H), 2.17 (s, 3H), 0.58–0.54 (m, 2H), 036–0.34 (m, 2H); ^13^C NMR (100 MHz, DMSO-d_6_): δ 171.82, 162.61, 161.42, 157.76, 139.31, 129.93, 129.56, 119.94, 84.26, 54.58, 46.20, 43.89, 42.03, 22.81, 6.10; LC-MS (ESI): *m/z* 367.2036 [M + H]^+^.

#### 2-(4-(6-Morpholinopyrimidin-4-ylamino)phenyl)-N-cyclopropylacetamide **(13ai)**

Yellow solid (0.038 g, 33.92%); m.p. 110–112 °C; ^1^H NMR (400 MHz, DMSO-d_6_): δ 9.00 (s, 1H), 8.15 (s, 1H), 8.04 (s, 1H), 7.43 (d, *J* = 12.0Hz, 2H), 7.10 (d, *J* = 8.0Hz, 2H), 5.90 (s, 1H), 3.63 (t, *J* = 4.0Hz, 4H), 3.40 (t, *J* = 4.0Hz, 4H), 3.23 (s, 2H), 2.60–2.53 (m, 1H), 0.59–0.59 (m, 2H), 0.36–0.33 (m, 2H); ^13^C NMR (100 MHz, DMSO-d_6_): δ 171.82, 162.88, 161.43, 157.74, 139.23, 130.03, 129.57, 120.01, 84.30, 66.20, 44.36, 42.02, 22.81, 6.10; LC-MS (ESI): *m/z* 354.3063 [M +H]^+^.

#### 2-(4-(6-(Butylamino)pyrimidin-4-yloxy)phenyl)-N-(4-tert-butylthiazol-2-yl)acetamide **(13aj)**

Colourless solid (0.055 g, 37.93%); m.p. 102–104 °C; ^1^H NMR (400 MHz, acetone-d_6_): δ 11.09 (s, 1H), 8.07 (s, 1H), 7.44 (d, *J* = 8.0Hz, 2H), 7.08 (d, *J* = 8.0Hz, 2H), 6.64 (s, 1H), 6.51 (s, 1H), 5.80 (s, 1H), 3.90 (s, 2H), 3.34 (s, 2H), 1.56 (t, *J* = 8.0Hz, 2H), 1.40–1.35 (m, 2H), 1.23 (s, 9H), 0.90 (t, *J* = 4.0Hz, 3H); ^13^C NMR (100 MHz, acetone-d_6_): δ 168.72, 165.14, 160.68, 158.03, 157.10, 152.48, 131.64, 130.41, 130.21, 121.51, 104.37, 85.55, 41.53, 40.50, 34.07, 31.27, 27.92, 19.78, 13.15; LC-MS (ESI): *m/z* 440.2009 [M +H]^+^.

#### N-(2,4-Dimethoxybenzyl)-2-(4-(6-(1-benzylpiperidin-4-ylamino)pyrimidin-4-yloxy)phenyl)acetamide **(13ak)**

Yellow solid (0.048 g, 35.55%); m.p. 102–104 °C; ^1^H NMR (400 MHz, acetone-d_6_): δ 8.80 (s, 1H), 7.35–7.27 (m, 6H), 7.23–7.20 (m, 1H), 7.10 (d, *J* = 8.0Hz, 1H), 7.02 (d, *J* = 8.0Hz, 2H), 6.50 (d, *J* = 4.0Hz, 1H), 6.46 (d, *J* = 8.0Hz, 1H), 6.43 (d, *J* = 4.0Hz, 1H), 6.41 (d, *J* = 4.0Hz, 1H), 5.77 (s, 1H), 4.28 (d, *J* = 4.0Hz, 2H), 3.77 (s, 3H), 3.75 (s, 3H), 3.52 (s, 2H), 3.47 (s, 2H), 3.41–3.36 (m, 1H), 2.81 (s, 2H), 2.11 (d, *J* = 8.0Hz, 2H), 1.94 (d, *J* = 12.0Hz, 2H), 1.57–1.49 (m, 2H); ^13^C NMR (100 MHz, acetone-d_6_): δ 169.71, 164.37, 160.40, 158.35, 158.13, 152.06, 139.09, 133.22, 130.27, 129.46, 129.44, 128.71, 128.04, 126.75, 121.30, 119.18, 104.04, 98.06, 62.62, 54.81, 54.67, 52.17, 42.11, 42.06, 37.90, 37.77, 31.98; LC-MS (ESI): *m/z* 568.1732 [M + H]^+^.

#### 2-(4-(6-(4-(Methylsulfonyl)phenylamino)pyridin-2-ylamino)phenyl)-N-(3-tert-butyl-1-methyl-1H-pyrazol-5-yl)acetamide **(18)**

Sticky white solid (0.040 g, 29.85%); ^1^H NMR (400 MHz, DMSO-d_6_): δ 9.98 (s, 1H), 9.39 (s, 1H), 8.85 (s, 1H), 7.80 (d, *J* = 8.0 Hz, 2H), 7.67 (d, *J* = 8.0 Hz, 2H), 7.46–7.39 (m, 3H), 7.20 (d, *J* = 12.0Hz, 2H), 6.31 (dd, *J* = 8.0, 12.0 Hz, 2H), 6.02 (s, 1H), 3.59 (s, 2H), 3.54 (s, 3H), 3.08 (s, 3H), 1.15 (s, 9H); ^13^C NMR (100 MHz, DMSO-d_6_): δ 169.78, 159.01, 154.97, 153.59, 146.88, 140.39, 139.28, 136.74, 130.86, 129.69, 128.41, 128.30, 119.60, 117.40, 101.70, 101.38, 95.42, 44.50, 41.97, 35.69, 32.21, 30.75; LC-MS (ESI): *m/z* 533.2224 [M +H]^+^.

### Synthesis of tert-butyl 4-(6-chloropyrimidin-4-ylamino)phenylcarbamate (20)

To the mixture of tert-butyl 4-aminophenylcarbamate **19** (2.09 g, 10.06 mmol) and 4,6-dichloropyrimidine **6** (1 g, 6.71 mmol) in EtOH (20 mL) was added triethyl amine (1.17 g, 11.58 mmol) The reaction mixture was stirred at 80 °C for 12 h. After completion of reaction as indicate by TLC, the solvent was removed under reduced pressure. The crude product thus obtained was purified by silica gel (mesh 100–200) flash chromatography with hexanes/EtOAc (1:3) to afford **20** as a white solid (2.85 g, 89.06%); m.p. 155–157 °C; ^1^H NMR (400 MHz, CDCl_3_): δ 8.41 (s, 1H), 7.43 (d, *J* = 8.0Hz, 3H), 7.22 (d, *J* = 8.0Hz, 2H), 6.66 (s, 1H), 6.58 (s, 1H), 1.53 (s, 9H); ^13^C NMR (100 MHz, CDCl_3_): δ 162.52, 160.45, 158.56, 152.71, 136.62, 131.71, 124.71, 119.73, 102.29, 80.91, 28.29; LC-MS (ESI): *m/z* 320.9424 [M +H]^+^.

### Synthesis of *tert*-butyl 4-(6-(4-(methylsulfonyl)phenylamino)pyrimidin-4-ylamino)phenylcarbamate (21)

The 4-(methylsulfonyl)benzenamine **8a** (0.802 g, 4.68 mmol) and Cs_2_CO_3_ (2.53 g, 7.78 mmol) were added to the solution of compound **20** (1 g, 3.12 mmol) in dioxane (4 ml). The reaction mixture was then degassed with argon. After completion of 5 minutes, Pd(PPh_3_)_4_ (0.108 g, 0.093 mmol) was added and the reaction mixture was allowed to stirr at 110 °C for 12 h. After completion of reaction as indicated by TLC, the solvent was removed under reduced pressure. The obtained residue was slowly basified with aqueous NaHCO_3_ and extracted with ethyl acetate (100 mL × 3). The organic layer was washed with aqueous NaHCO_3_ (100 mL × 3) followed by brine (100 mL × 3) solution. The obtained organic layer was dried over MgSO_4_, and solvent was evaporated to yield **21** as a yellow solid (0.950 g, 66.90%). m.p. 178–180 °C; ^1^H NMR (400 MHz, DMSO-d_6_): δ 9.61 (s, 1H), 9.21 (s, 1H), 9.08 (s, 1H), 8.28 (s, 1H), 7.83 (d, *J* = 8.0Hz, 2H), 7.74 (d, *J* = 12.0Hz, 2H), 7.38–7.32 (m, 4H), 6.12 (s, 1H), 3.10 (s, 3H), 1.43 (s, 9H); ^13^C NMR (100 MHz,DMSO-d_6_): δ 161.42, 160.27, 158.10, 153.27, 146.01, 135.08, 134.62, 132.36, 128.55, 121.92, 119.21, 118.56, 87.72, 79.25, 44.46, 28.58; LC-MS (ESI): *m/z* 455.9876 [M + H]^+^.

### Synthesis of N4-(4-aminophenyl)-N6-(4-(methylsulfonyl)phenyl)pyrimidine-4,6-diamine (22)

Compound **21** (0.950 g, 1.0 mmol) was treated with 25% TFA/DCM at RT for 2 h, after which time the volatiles were removed in vacuo. The crude was diluted with EtOAc (100 mL), washed with saturated aq NaHCO_3_ (100 mL × 3), and then with brine solution (100 mL × 3). The organic layer was dried over MgSO_4_ and concentrated in vacuo to provide the product **22** as yellow solid (0.736 g, 99.72%). m.p. 149–151 °C; ^1^H NMR (400 MHz, DMSO-d_6_): δ 9.51 (s, 1H), 8.66 (s, 1H), 8.20 (s, 1H), 7.81 (d, *J* = 8.0Hz, 2H), 7.72 (d, *J* = 8.0Hz, 2H), 6.99 (d, *J* = 8.0Hz, 2H), 6.54 (d, *J* = 8.0Hz, 2H), 5.93 (s, 1H), 4.92 (s, 2H), 3.08 (s, 3H); ^13^C NMR (100 MHz, DMSO-d_6_): δ 162.45, 160.25, 158.08, 146.18, 145.99, 132.11, 128.51, 128.21, 124.82, 118.38, 114.65, 86.34, 44.47; LC-MS (ESI): *m/z* 356.1468 [M +H]^+^.

### General procedure for synthesis of 4,6-diaminopyrimidine series of compounds 24a-c

The reaction of compound **22** (0.100 g, 0.281 mmol) or its structural analogs with 3-tert-butyl-5-isocyanato-1-methyl-*1H*-pyrazole (**23**) (0.057 g, 0.422 mmol), in presence of triethyl amine (0.098 g, 0.970 mmol), in DCM (2 mL) was stirred at 45 °C for 5 h. The completion of the reaction was monitored by TLC. After completion of the reaction, the organic layer was evaporated. The crude product was purified on silica gel column (mesh 100–200) using DCM: MeOH gradient (100: 0 to 70: 30 ratio of DCM: MeOH). The desired products **24a-c** were isolated in moderate to good yields.

#### 1-(4-(6-(4-(Methylsulfonyl)phenylamino)pyrimidin-4-ylamino)phenyl)-3-(3-tert-butyl-1-methyl-1H-pyrazol-5-yl)urea **(24a)**

Yellow solid (0.030 g, 20.0%); m.p. 238–240 °C; ^1^H NMR (400 MHz, DMSO-d_6_): δ 9.66 (s, 1H), 9.15 (s, 1H), 8.81 (s, 1H), 8.45 (s, 1H), 8.32 (s, 1H), 8.86 (d, *J* = 8.0Hz, 2H), 7.78 (d, *J* = 8.0Hz, 2H), 7.41 (s, 4H), 6.18 (s, 1H), 6.03 (s, 1H), 3.59 (s, 3H), 3.13 (s, 3H), 1.20 (s, 9H); ^13^C NMR (100 MHz, DMSO-d_6_): δ 161.38, 160.27, 158.92, 158.09, 152.29, 146.02, 137.62, 134.74, 132.38, 130.93, 128.55, 121.94, 119.33, 118.58, 93.87, 87.83, 44.47, 35.36, 32.23, 30.81; LC-MS (ESI): *m/z* 535.1774 [M +H]^+^.

#### 1-(4-(6-(Methylamino)pyrimidin-4-ylamino)phenyl)-3-(3-tert-butyl-1-methyl-1H-pyrazol-5-yl)urea **(24b)**

Yellow solid (0.020 g, 18.18%); m.p. 242–244 °C; ^1^H NMR (400 MHz, DMSO-d_6_): δ 8.74 (s, 2H), 8.44 (s, 1H), 7.99 (s, 1H), 7.35 (d, *J* = 8.0Hz, 2H), 7.30 (d, *J* = 8.0Hz, 2H), 6.67 (d, *J* = 4.0Hz, 1H), 5.99 (s, 1H), 5.60 (s, 1H), 3.54 (s, 3H), 2.67 (d, *J* = 4.0Hz, 3H), 1.16 (s, 9H); ^13^C NMR (100 MHz, DMSO-d_6_): δ 163.54, 160.70, 159.02, 157.85, 152.35, 137.64, 135.61, 134.11, 121,17, 119.40, 93.96, 85.39, 35.29, 32.20, 30.77, 27.86; LC-MS (ESI): *m/z* 395.0813[M +H]^+^.

#### 1-(4-(6-(4-(Methylsulfonyl)phenylamino)pyrimidin-4-ylamino)phenyl)-3-(5-tert-butylisoxazol-3-yl)urea **(24c)**

Yellow solid (0.031 g, 21.23%); m.p. 245–247 °C; ^1^H NMR (400 MHz, DMSO-d_6_): δ 9.67 (s, 1H), 9.44 (s, 1H), 9.19 (s, 1H), 8.76 (s, 1H), 8.33 (s, 1H), 7.86 (d, *J* = 8.0Hz, 2H), 7.78 (d, *J* = 8.0Hz, 2H), 7.45 (d, *J* = 8.0Hz, 2H), 7.39 (d, *J* = 12.0Hz, 2H), 6.48 (s, 1H), 6.18 (s, 1H), 3.13 (s, 3H), 1.28 (s, 9H); ^13^C NMR (100 MHz, DMSO-d_6_): δ 180.53, 161.31, 160.26, 158.85, 158.09, 151.79, 145.99, 135.29, 134.21, 132.41, 128.56, 121.76, 119.72, 118.60, 92.83, 87.98, 44.46, 32.89, 28.78; LC-MS (ESI): *m/z* 522.1923 [M +H]^+^.

### Biochemical Enzyme Inhibition Assay

Kinase activity was measured in a microfluidics assay that monitors the separation of a phosphorylated product from substrate^31,32^. The assay was run using a 12-sipper chip on a Caliper EZ Reader II (PerkinElmer®, Walthman, USA) with separation buffer (100 mM HEPES, 10 mM EDTA, 0.015% Brij-35, 0.1% CR-3 [PerkinElmer®, Walthman, USA]). In 96-well polypropylene plates (Greiner, Frickenhausen, Germany) compound stocks (20 mM in DMSO) were diluted into kinase buffer (50 mM HEPES, 0.075% Brij-35, 0.1% Tween 20, 2 mM DTT, 10 mM MgCl_2_, and 0.02% NaN_3_) in 12-point ½log dilutions (2 mM–6.32 nM). After, 1 μL was transferred into a 384-well polypropylene assay plate (Greiner, Frickenhausen, Germany). The FLT3 enzyme (Invitrogen™, Grand Island, USA) was diluted in kinase buffer to a concentration of 2 nM and 5 µL of the enzyme mixture was transferred to the assay plate. The inhibitors/FLT3 enzyme were incubated for 60 minutes with minor shaking. A substrate mix was prepared containing ATP (Ambresco®, Solon, USA) and 5FAM tagged FLT3 peptide (peptide #22, 5′ FAM-EPLYWSFPA, PerkinElmer®, Walthman, USA) dissolved in kinase buffer, and 5 µL of the substrate mix was added to the assay plate. Running concentrations were as follows: ATP (190 µM), peptide (1.5 µM), compound 12-point ½log dilutions (0.2 mM–0.632 nM). For positive control, no inhibitor was added. For negative control, no enzyme was added. For running control, quizartinib was utilized. The plate was run until 10–20% conversion based on the positive control wells. The following separation conditions were utilized: upstream voltage −500V; downstream voltage, −1900V; chip pressure −0.8. Percent inhibition was measured for each well comparing starting peptide to phosphorylated product peaks relative to the baseline. Dose response curves, spanning the IC_50_ dose, were generated in GraphPad Prisim 6 and fit to an exponential one-phase decay line and IC_50_ values were obtained from the half-life value of the curve. IC_50_ values were generated in duplicate and error was calculated from the standard deviation between values.

### Mechanism of Inhibition Procedure

Compound **13a** was pre-incubated with the FLT3 kinase at 1, 3, 5, 10, 20, 30, 50, and 90 minutes. After the pre-incubation, IC_50_ values at each incubation interval was determined according to the procedure outlined in section 4.3. In a separate experiment, compound **13a** was pre-incubated with the FLT3 kinase for 60 minutes. After, IC_50_ values were determined at 5, 10, 20, 40, 80, and 160 µM ATP. To determine IC_50_ values at each concentration of ATP, the same procedure was followed as outlined in section S10.

### Computational Modeling

Computational modeling^31–33^ studies were completed using AutoDock Vina^33^, AutoDock Tools, and Discovery Studio 3.5. Using AutoDock Tools, kinase crystal structures were prepared as follows: 1) All hydrogens were added as ‘Polar Only’ 2) A grid box for the ATP binding site was created. Compounds to be computationally modeled were assigned appropriate rotatable bonds using AutoDock Tools. To computationally model the compounds, AutoDock Vina was employed. AutoDock Vina provides docking scores in terms of ΔG values. After the modeling study, kinase inhibitors docked in FLT3 were visualized and analyzed with Discovery Studio 3.5.

### Cell Cultures

Stable BaF3 populations expressing activated FLT3 were generated by retroviral spinfection with the appropriate mutated plasmid followed by selection and growth factor withdrawal as previously described^34^. The BaF3 cell line was originally obtained from the laboratory of Charles Sawyers and has not been authenticated. MV411 and Molm14 cells were obtained from the laboratory of Scott Kogan and authenticated by Promega STR analysis in June 2013. All cell lines were mycoplasma-free. Cells were incubated with compounds for 48 hours and proliferation was assessed using CellTiter-Glo (Promega; Madison, WI) according to the manufacturer’s recommendation on a SpectraMax M3 microplate reader using SpectraMax Software (Molecular Devices; Sunnyvale, CA). All cell viability data shown is reflective of experiments performed a minimum of three times.

## Electronic supplementary material

supporting information

## References

[1] Levis M, Small D (2003). Novel FLT3 tyrosine kinase inhibitors. Expert Opinion on Investigational Drugs.

[2] Levis M, Small D (2003). FLT3: ITDoes matter in leukemia. Leukemia.

[3] Smith CC (2012). Validation of ITD mutations in FLT3 as a therapeutic target in human acute myeloid leukaemia. Nature.

[4] Wu H (2016). Discovery of a highly potent FLT3 kinase inhibitor for FLT3-ITD-positive AML. Leukemia.

[5] Sun D (2016). Discovery and Rational Design of Pteridin-7(8H)-one-Based Inhibitors Targeting FMS-like Tyrosine Kinase 3 (FLT3) and Its Mutants. Journal of Medicinal Chemistry.

[6] Saleh AM (2016). Novel anticancer compound [trifluoromethyl-substituted pyrazole N-nucleoside] inhibits FLT3 activity to induce differentiation in acute myeloid leukemia cells. Cancer Letters.

[7] Mashkani B, Tanipour MH, Saadatmandzadeh M, Ashman LK, Griffith R (2016). FMS-like tyrosine kinase 3 (FLT3) inhibitors: Molecular docking and experimental studies. European journal of pharmacology.

[8] Hatcher JM (2016). Discovery of a Highly Potent and Selective Indenoindolone Type 1 Pan-FLT3 Inhibitor. ACS Medicinal Chemistry Letters.

[9] Hassanein M, Almahayni MH, Ahmed SO, Gaballa S, El Fakih R (2016). FLT3 Inhibitors for Treating Acute Myeloid Leukemia. Clin Lymphoma Myeloma Leuk.

[10] Chen Y (2016). Identification of an orally available compound with potent and broad FLT3 inhibition activity. Oncogene.

[11] Xu Y (2015). *Discovery of novel N-(5-(tert-butyl*)isoxazol-3-yl)-N′-phenylurea analogs as potent FLT3 inhibitors and evaluation of their activity against acute myeloid leukemia *in vitro* and *in vivo*. Bioorganic & Medicinal Chemistry.

[12] Simon T (2015). Design of FLT3 Inhibitor - Gold Nanoparticle Conjugates as Potential Therapeutic Agents for the Treatment of Acute Myeloid Leukemia. Nanoscale Research Letters.

[13] Galanis A (2014). Crenolanib is a potent inhibitor of FLT3 with activity against resistance-conferring point mutants. Blood.

[14] Smith CC (2014). Crenolanib is a selective type I pan-FLT3 inhibitor. Proceedings of the National Academy of Sciences of the United States of America.

[15] Zimmerman EI (2013). Crenolanib is active against models of drug-resistant FLT3-ITD-positive acute myeloid leukemia. Blood.

[16] Fathi AT (2013). Emergence of crenolanib for FLT3-mutant AML. Blood.

[17] Sanga, M. *et al*. An Open-Label, Single-Dose, Phase I Study of the Absorption, Metabolism, and Excretion of Quizartinib, a Highly Selective and Potent FLT3 Tyrosine Kinase Inhibitor, in Healthy Male Subjects, for the Treatment of Acute Myeloid Leukemia. *Xenobiotica* 1–43 (2016). 10.1080/00498254.2016.1217100 27460866

[18] Cooper TM (2016). A Phase I Study of Quizartinib Combined with Chemotherapy in Relapsed Childhood Leukemia: A Therapeutic Advances in Childhood Leukemia & Lymphoma (TACL) Study. Clinical cancer research: an official journal of the American Association for Cancer Research.

[19] Levis M (2013). Quizartinib in acute myeloid leukemia. Clinical Advances in Hematology & Oncology: H&O.

[20] Gallogly MM, Lazarus HM (2016). Midostaurin: an emerging treatment for acute myeloid leukemia patients. Journal of Blood Medicine.

[21] Wolleschak D (2014). Clinically relevant doses of FLT3-kinase inhibitors quizartinib and midostaurin do not impair T-cell reactivity and function. Haematologica.

[22] Stone RM (2012). Phase IB study of the FLT3 kinase inhibitor midostaurin with chemotherapy in younger newly diagnosed adult patients with acute myeloid leukemia. Leukemia.

[23] Fischer T (2010). Phase IIB trial of oral Midostaurin (PKC412), the FMS-like tyrosine kinase 3 receptor (FLT3) and multi-targeted kinase inhibitor, in patients with acute myeloid leukemia and high-risk myelodysplastic syndrome with either wild-type or mutated FLT3. Journal of clinical oncology: official journal of the American Society of Clinical Oncology.

[24] Frett B (2015). Computer aided drug discovery of highly ligand efficient, low molecular weight imidazopyridine analogs as FLT3 inhibitors. European Journal of Medicinal Chemistry.

[25] Park CH (2014). *Discovery of thienopyrimidine-based FLT3 in*hibitors from the structural modification of known IKKbeta inhibitors. Bioorganic & Medicinal Chemistry Letters.

[26] Oh C (2017). Synthetic strategy for increasing solubility of potential FLT3 inhibitor thieno[2,3-d]pyrimidine derivatives through structural modifications at the C2 and C6 positions. Bioorganic and Medicinal Chemistry Letters.

[27] Kim H (2016). Structural modifications at the 6-position of thieno[2,3-d]pyrimidines and their effects on potency at FLT3 for treatment of acute myeloid leukemia. European Journal of Medicinal Chemistry.

[28] Warkentin AA (2014). Overcoming myelosuppression due to synthetic lethal toxicity for FLT3-targeted acute myeloid leukemia therapy. eLife.

[29] Doss GP (2014). Structural signature of the G719S-T790M double mutation in the EGFR kinase domain and its response to inhibitors. Scientific Reports.

[30] Smith CC (2017). Heterogeneous resistance to quizartinib in acute myeloid leukemia (AML) revealed by single cell analysis. Blood.

[31] Frett B (2014). Identification of pyrazine-based TrkA inhibitors: design, synthesis, evaluation, and computational modeling studies. MedChemComm.

[32] Frett B, Moccia M, Carlomagno F, Santoro M, Li HY (2014). Identification of two novel RET kinase inhibitors through MCR-based drug discovery: design, synthesis and evaluation. European Journal of Medicinal Chemistry.

[33] Trott O, Olson AJ (2010). AutoDock Vina: improving the speed and accuracy of docking with a new scoring function, efficient optimization and multithreading. J. Comput. Chem..

[34] Smith CC (2012). Validation of ITD mutations in FLT3 as a therapeutic target in human acute myeloid leukaemia. Nature.

