# Supplementary material for: Rational Design, Synthesis and Biological Evaluation of Pyrimidine-4,6-diamine derivatives as Type-II inhibitors of FLT3 Selective Against c-KIT
Source: Sci Rep. 2018 Feb 27;8:3722. doi: 10.1038/s41598-018-21839-3 (PMC5829162; doi:10.1038/s41598-018-21839-3)
Supplement: Supplementary file 1 — supporting information [file 41598_2018_21839_MOESM1_ESM.pdf]

Supporting Information

Rational Design, Synthesis and Biological Evaluation of Pyrimidine-4,6-diamine derivatives as Type-II inhibitors of FLT3 Selective Against c-KIT

Jaideep B. Bharate[1#], Nicholas McConnell[1,2#], Naresh Gunaganti[1], Lingtian Zhang[1], Naga Rajiv Lakkaniga,[1] Lucky Ding,[3] Neil P. Shah[3,4], Brendan Frett[1], and Hong-yu Li[1]*

[1]Department of Pharmaceutical Sciences, University of Arkansas for Medical Sciences, Little Rock, USA, 72205

[2]Department of Pharmacology and Toxicology, The University of Arizona, Tucson, AZ, 85721
[3]Division of Hematology/Oncology, University of California, San Francisco, CA, 94143

[4]Helen Diller Family Comprehensive Cancer Center, University of California, San Francisco, CA, 94115

*****Corresponding author

#Equal contributors

Prof. Hong-Yu Li

Department of Pharmaceutical Sciences,

University of Arkansas for Medical Sciences, Little Rock, USA, 72205

Email: HLi2@uams.edu

TABLE OF CONTENTS

Sr. No.	CONTENTS	Page nos
**S1-S9**	A. Experimental procedures & experimental data of Pyrimidine-4,6-diamine derivatives.	**S2-S23**
**S10-S13**	Biological methods for the determination of In-vitro activities	**S24-S25**
**S14-S60**	B. NMR Spectras Scans (^{1}H NMR, ^{13}C NMR) of Pyrimidine-4,6-diamine derivatives.	**S26-S119**
**S61**	References	**S120**

A. EXPERIMENTAL PROCEDURES:

S1. General

All solvents were reagent grade or HPLC grade and all starting materials were obtained from commercial sources and used without further purification. Purity of final compounds was assessed using a Thermo Finnigan LCQ Deca with Thermo Surveyor LCMS System at variable wavelengths of 254 nm and 214 nm and final compound purity was >95%. The HPLC mobile phase consisted of a water-methanol gradient buffered with 0.1% formic acid. ^{1}H NMR spectra were recorded at 400 MHz and ^{13}C spectra were recorded at 100 MHz, both completed on a Varian 400 MHz instrument (Model# 4001S41ASP). High-resolution mass spectrometry was completed using a Bruker 9.4 T Apex-Qh hybrid Fourier transfer ion-cyclotron resonance (FT-ICR) mass spectrometer. Compound activity was determined with the EZ Reader II plate reader (PerkinElmer®, Walthman, USA). All compounds were purified using Silica gel (0.035-0.070 mm, 60 Å) flash chromatography, unless otherwise noted. Microwave assisted reactions were completed in sealed vessels using a Biotage Initiator microwave synthesizer.

S2. Synthesis of ethyl 2-(4-(6-chloropyrimidin-4-yl)amino)phenyl)acetate (7)

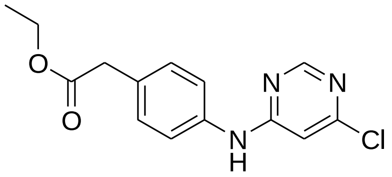

To the mixture of ethyl 4-aminophenyl acetate **5** (20 g, 111.60 mmol) and 4,6-dichloropyrimidine **6** (13.79 g, 92.56 mmol) in EtOH (200 mL) was added triethyl amine

(17.80g, 176.23 mmol) The reaction mixture was stirred at 80 °C for 12 h. After completion of reaction as indicate by TLC, the solvent was removed under reduced pressure. The crude product thus obtained was purified by silica gel (100-200 mesh) flash chromatography with hexanes/EtOAc (1:3) to afford **7** as white solid (26.50 g, 81.53%); m.p. 132-134 °C; ^{1}H NMR (400 MHz, DMSO-d_6): δ 9.82 (s, 1H), 8.42 (s, 1H), 7.51 (d, *J* = 8.0 Hz, 2H), 7.20 (d, *J* = 8.0 Hz, 2H), 6.74 (s, 1H), 4.04 (q, *J* = 8.0, 16.0 Hz, 2H), 3.58 (s, 2H), 1.14 (t, *J* = 8.0 Hz, 3H); ^{13}C NMR (100 MHz, DMSO-d_6): δ 171.62, 161.63, 158.89, 158.35, 137.97, 130.20, 129.80, 120.92, 105.26, 60.66, 14.50; LC-MS (ESI): *m/z* 292.1309 $[M+H]^+$.

S3. Synthesis of ethyl 2-(4-(6-(4-methylsulfonyl)phenylamino)pyrimidin-4-ylamino)phenyl)acetate (10)

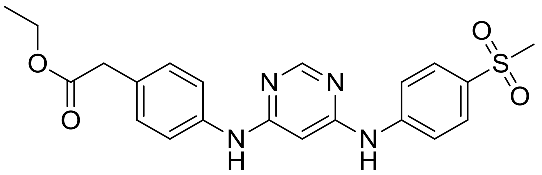

The 4-(methylsulfonyl)benzenamine **8a** (0.882 g, 5.5 mmol) and Cs_2CO_3 (2.79 g, 8.58 mmol) were added to the solution of compound **7** (1 g, 3.4 mmol) in dioxane (4 ml). The reaction mixture was then degassed with argon. After 5 minutes, $Pd(PPh_3)_4$ (0.118g, 0.102 mmol) was added and the reaction mixture was allowed to stir at 110 °C for 12 h. After completion of the reaction as indicated by TLC, the solvent was removed under reduced pressure. The reaction was slowly basified with aqueous $NaHCO_3$ and the obtained aqueous layer was extracted with ethyl acetate (100 mL X 3). The organic phase was washed 3 times with aqueous $NaHCO_3$ followed by brine solution. The obtained organic layer was then dried over $MgSO_4$, and solvent was evaporated Then crude product thus obtained was purified by silica gel (100-200 mesh) flash chromatography with hexanes/EtOAc (1:7) to afford **10** as a yellow solid (0.920 g, 63.01%); m.p. 188-190 °C; ^{1}H NMR (400 MHz, DMSO-d6): δ 9.68 (s, 1H), 9.23 (s, 1H), 8.31 (s, 1H), 7.82 (d, *J* = 12.0 Hz, 2H), 7.76 (d, *J* = 12.0 Hz, 2H), 7.44 (d, *J* = 8.0 Hz, 2H), 7.18 (d, *J* = 8.0 Hz, 2H), 6.22 (s, 1H), 4.05 (q, *J* = 8.0, 16.0 Hz, 2H), 3.56 (s, 2H), 3.10 (s, 3H), 1.15 (t, *J* = 8.0 Hz, 3H); ^{13}C NMR (100 MHz, DMSO-d6): δ 171.98, 161.09, 160.26, 158.08, 145.84, 138.96, 132.33, 130.13, 128.81, 128.54, 120.82, 118.80, 88.25, 60.80, 44.35, 40.15, 14.44; LC-MS (ESI): *m/z* 427.1897 $[M+H]^+$.

S4. Synthesis of 2-(4-(6-(4-(methylsulfonyl)phenylamino)pyrimidin-4-ylamino)phenyl)acetic acid (11)

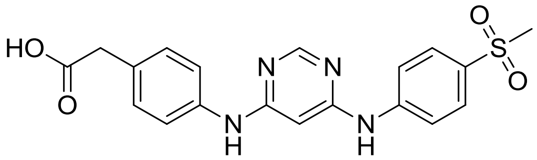

Compound **10** (0.500 g, 1.17 mmol) was added to THF/water (1:1, 8 mL) in a pressure reaction vessel. LiOH (0.084 g, 3.5 mmol) was then added and the reaction was heated to 100 °C for 5 h (or 15 min in microwave). TLC confirmed the complete consumption of compound **10**. Organic solvent was evaporated and the water solution was extracted with DCM (5 x 100 ml) and all DCM layers were discarded. Then, the reaction was acidified with 3M HCl to pH ~4.0. The acidified aqueous solution was extracted (10 ml x 3) with 4:1 DCM/IPA. All extracts were combined, dried, and condensed to yield compound **11** as a white solid (0.400 g, 85.65%); m.p. 124-126 °C; ^{1}H NMR (400 MHz, DMSO-d_6): δ 12.24 (s, 1H), 9.73 (s, 1H), 9.27 (s, 1H), 8.32 (s, 1H), 7.86 (d, *J* = 8.0 Hz, 2H), 7.76 (d, *J* = 8.0 Hz, 2H), 7.45 (d, *J* = 8.0 Hz, 2H), 7.17 (d, *J* = 8.0 Hz, 2H), 6.24 (s, 1H), 3.49 (s, 2H), 3.11 (s, 3H); ^{13}C NMR (100 MHz, DMSO-d_6): δ 173.30, 161.18, 160.33, 158.08, 145.99, 139.00, 132.43, 130.16, 129.32, 128.55, 120.74, 118.65, 88.34, 44.47, 25.91; LC-MS (ESI): *m/z* 399.1773 $[M+H]^+$.

S5. General procedure for synthesis of 4,6-diaminopyrimidine series of compounds 13a-13ak: The reaction of compound **11** (0.100 g, 0.251 mmol) or its structural analogs with 5-(tert-butyl-1-methyl-1H-pyrazol-5-amine (**12a**) (0.057 g, 0.376 mmol), in presence of EDC (0.120 g, 0.625 mmol), HOAt (0.034 g, 0.249 mmol), and DIPEA (0.053 mL, 0.410 mmol) in DMF (2 mL) was stirred at room temperature for overnight. The completion of the reaction was monitored by TLC. After completion of the reaction, the organic layer was evaporated. The crude product was purified on silica gel column (mesh 100-200) using DCM: MeOH gradient (100: 0 to 70: 30 ratio of DCM: MeOH) mobile phase. The desired products **13a-13ak** were isolated in moderate to good yields.

S5.a. 2-(4-(6-(4-(methylsulfonyl)phenylamino)pyrimidin-4-ylamino)phenyl)-N-(3-tert-butyl-1-methyl-1H-pyrazol-5-yl)acetamide (13a)

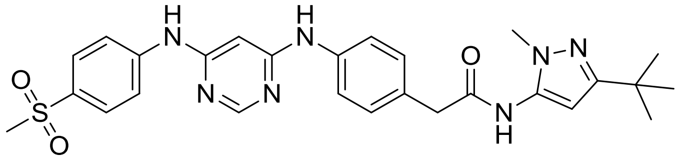

White solid (0.054 g, 40.60 %); m.p 134-136 °C; ^{1}H NMR (400 MHz, DMSO-d$_6$): δ 10.02 (s, 1H), 9.70 (s, 1H), 9.28 (s, 1H), 8.36 (s, 1H), 7.87 (d, *J* = 8.0 Hz, 2H), 7.79 (d, *J* = 8.0 Hz, 2H), 7.50 (d, *J* = 8.0 Hz, 2H), 7.28 (d, *J* = 8.0 Hz, 2H), 6.25 (s, 1H), 6.05 (s, 1H), 3.62 (s, 2H), 3.57 (s, 3H), 3.14 (s, 3H), 1.18 (s, 9H); ^{13}C NMR (100 MHz, DMSO-d$_6$): δ169.58, 161.18, 160.31, 159.02, 158.09, 145.94, 139.03, 138.03, 136.71, 132.47, 129.89, 128.56, 120.80, 118.65, 95.38, 88.33, 44.46, 41.98, 35.70, 32.21, 30.74; LC-MS (ESI): *m/z* 534.2037 [M+H]$^+$.

S5.b. 2-(4-(6-(methylamino)pyrimidin-4-ylamino)phenyl)-N-(3-tert-butyl-1-methyl-1H-pyrazol-5-yl)acetamide (13b)

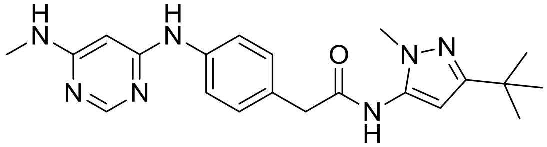

Yellow solid (0.045 g, 29.60%); m.p.194-196 °C; ^{1}H NMR (400 MHz, DMSO-d$_6$): δ 9.98 (s, 1H), 8.88 (s, 1H), 8.06 (s, 1H), 7.46 (d, *J* = 12.0 Hz, 2H), 7.20 (d, *J* = 12.0 Hz, 2H), 6.77 (d, *J* = 4.0 Hz, 1H), 6.03 (s, 1H), 5.69 (s, 1H), 3.58 (s, 2H), 3.55 (s, 3H), 2.71 (d, *J* = 8.0 Hz, 3H), 1.17 (s, 9H); ^{13}C NMR (100 MHz, DMSO-d$_6$): δ 169.71, 163.68, 160.56, 159.02, 157.96, 139.85, 136.72, 129.71, 128.86, 120.01, 95.43, 83.68, 41.99, 35.68, 32.21, 30.75, 27.84; LC-MS (ESI): *m/z* 394.2388 [M+H]$^+$.

S5.c. 2-(4-(6-(3-(piperidin-1-yl)propylamino)pyrimidin-4-ylamino)phenyl)-N-(3-tert-butyl-1-methyl-1H-pyrazol-5-yl)acetamide (13c)

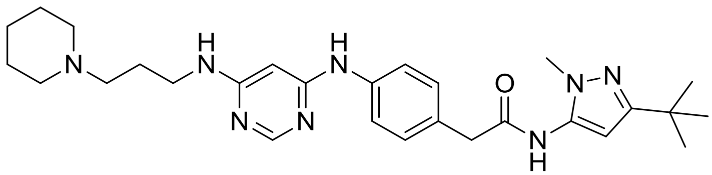

Sticky yellow solid (0.041 g, 30.14%); ^{1}H NMR (400 MHz, acetone-d_6): δ 9.19 (s, 1H), 8.08 (s, 2H), 7.52 (q, *J* = 4.0, 8.0 Hz, 2H), 7.29 (d, *J* = 8.0 Hz, 2H), 6.51 (s, 1H), 6.06 (s, 1H), 5.84 (s, 1H), 3.67 (s, 2H), 3.58 (s, 3H), 3.33 (t, *J* = 8.0 Hz, 2H), 2.58 (s, 6H), 1.84 (t, *J* = 8.0 Hz, 2H), 1.66 (t, *J* = 4.0 Hz, 4H), 1.47 (t, *J* = 8.0 Hz, 2H), 1.20 (s, 9H); ^{13}C NMR (100 MHz, DMSO-d_6): δ 170.70, 169.70, 163.10, 159.02, 158.02, 139.82, 136.72, 129.71, 128.90, 120.05, 95.41, 88.03, 56.63, 54.38, 42.32, 41.97, 37.55, 35.68, 32.20, 30.74, 25.81, 24.37; LC-MS (ESI): *m/z* 505.2822 $[M+H]^+$.

S5.d. 2-(4-(6-(3-(pyrrolidin-1-yl)propylamino)pyrimidin-4-ylamino)phenyl)-N-(3-tert-butyl-1-methyl-1H-pyrazol-5-yl)acetamide (13d)

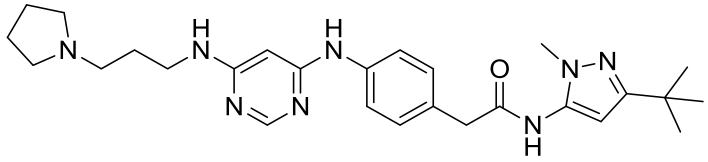

Sticky pale yellow solid (0.039 g, 28.26%); ^{1}H NMR (400 MHz, DMSO-d_6): δ 9.96 (s, 1H), 8.82 (s, 1H), 8.03 (s, 1H), 7.42 (d, *J* = 8.0 Hz, 2H), 7.18 (d, *J* = 8.0 Hz, 2H), 6.83 (s, 1H), 6.00 (s, 1H), 5.70 (s, 1H), 3.55 (s, 2H), 3.53 (s, 3H), 3.17 (s, 2H), 2.41 (s, 6H), 1.64 (s, 6H), 1.15 (s, 9H); ^{13}C NMR (100 MHz, DMSO-d_6): δ 169.68, 163.13, 159.01, 158.02, 139.84, 136.72, 130.40, 129.71, 128.88, 120.04, 95.40, 90.18, 54.02, 53.75, 45.58, 41.98, 35.69, 32.21, 30.75, 28.62, 23.50; LC-MS (ESI): *m/z* 491.2592 $[M+H]^+$.

S5.e. 2-(4-(6-(4-(methylsulfonyl)phenylamino)pyrimidin-4-yloxy)phenyl)-N-(3-tert-butyl-1-methyl-1H-pyrazol-5-yl)acetamide (13e)

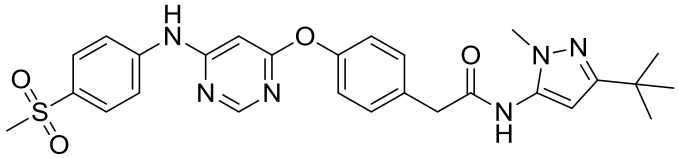

Sticky yellow solid (0.042 g, 31.57%); ^{1}H NMR (400 MHz, DMSO-d_6): δ 10.07 (d, *J* = 4.0 Hz, 2H), 8.43 (s, 1H), 7.87 (d, *J* = 8.0 Hz, 2H), 7.80 (d, *J* = 8.0 Hz, 2H), 7.39 (d, *J* = 8.0 Hz, 2H), 7.16 (d, *J* = 8.0 Hz, 2H), 6.19 (s, 1H), 6.04 (s, 1H), 3.69 (s, 2H), 3.57 (s, 3H), 3.12 (s, 3H), 1.16 (s, 9H); ^{13}C NMR (100 MHz, DMSO-d_6): δ 169.97, 169.23, 162.40, 159.07, 158.57, 151.59,

145.05, 136.66, 133.53, 133.41, 131.11, 128.65, 121.94, 119.17, 95.30, 90.73, 44.37, 40.55, 35.74, 32.22, 30.75; LC-MS (ESI): *m/z* 535.2312 $[M+H]^+$.

S5.f. 2-(4-(6-(3-(trifluoromethyl)phenylamino)pyrimidin-4-yloxy)phenyl)-N-(3-tert-butyl-1-methyl-1H-pyrazol-5-yl)acetamide (13f)

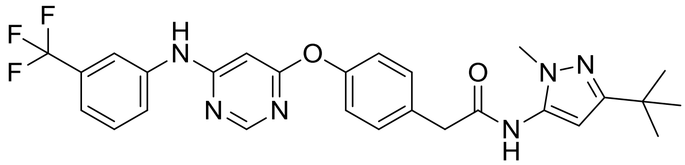

Yellow solid (0.046 g, 34.32%); m.p. 140-142 °C; ^{1}H NMR (400 MHz, acetone-d_6): δ 9.27 (s, 1H), 9.07 (s, 1H), 8.38 (s, 1H), 8.21 (s, 1H), 7.88 (d, *J* = 8.0 Hz, 1H), 7.51 (t, *J* = 8.0 Hz, 1H), 7.44 (d, *J* = 8.0 Hz, 2H), 7.32 (d, *J* = 8.0 Hz, 1H), 7.14 (d, *J* = 8.0 Hz, 2H), 6.17 (s, 1H), 6.11 (s, 1H), 3.77 (s, 2H), 3.60 (s, 3H), 1.21 (s, 9H); ^{13}C NMR (100 MHz, acetone-d_6): δ 170.92, 169.63, 163.50, 160.02, 159.03, 152.82, 141.87, 137.03, 133.64, 131.47, 131.19, 130.55, 126.65, 123.89, 122.40, 119.45, 116.64, 95.96, 90.64, 42.76, 35.65, 32.69, 30.79; LC-MS (ESI): *m/z* 525.2342 $[M+H]^+$.

S5.g. 2-(4-(6-(4-(methylsulfonyl)phenylamino)pyrimidin-4-ylamino)phenyl)-N-(3-tert-butyl-1-(4-fluorophenyl)-1H-pyrazol-5-yl)acetamide (13g)

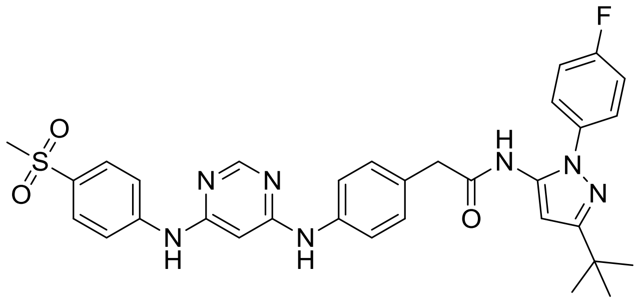

Pale yellow solid (0.043 g, 34.95%); m.p. 168-170 °C; ^{1}H NMR (400 MHz, acetone-d_6): δ 8.98 (s, 1H), 8.89 (s, 1H), 8.47 (s, 1H), 8.40 (s, 1H), 7.95 (d, *J* = 8.0 Hz, 2H), 7.83 (d, *J* = 8.0 Hz, 2H), 7.54 (d, *J* = 8.0 Hz, 2H), 7.44-7.41 (m, 2H), 7.29 (d, *J* = 8.0 Hz, 2H), 7.18 (t, *J* = 8.0 Hz, 2H), 6.41 (s, 1H), 6.31 (s, 1H), 3.65 (s, 2H), 3.07 (s, 3H), 1.29 (s, 9H); ^{13}C NMR (100 MHz, acetone-d_6): δ 168.81, 162.48, (d, $^1J_{CF}$ = 244 Hz), 161.37, 161.27, 157.89, 145.65, 145.57, 138.97, 133.16, 129.80, 128.36, 125.88, 125.89, 120.97, 120.82, 118.53, 118.44, 115.84, (d, $^2J_{CF}$ = 23 Hz), 97.33, 87.68, 43.79, 42.29, 32.06, 28.36; LC-MS (ESI): *m/z* 614.2142 $[M+H]^+$.

S5.h. 2-(4-(6-(4-(methylsulfonyl)phenylamino)pyrimidin-4-ylamino)phenyl)-N-(3-tert-butyl-1-p-tolyl-1H-pyrazol-5-yl)acetamide (13h)

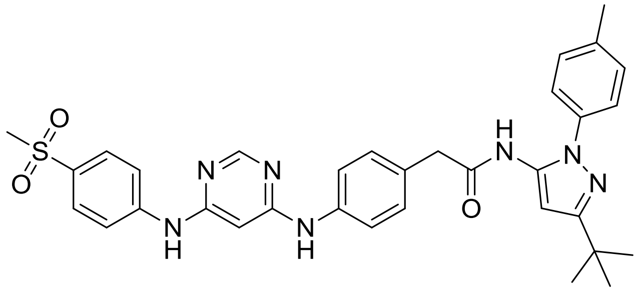

Yellow solid (0.049 g, 32.02%); m.p. 155-157 °C; ^{1}H NMR (400 MHz, acetone-d$_6$): δ 8.91 (s, 1H), 8.83 (s, 1H), 8.48 (s, 1H), 8.41 (s, 1H), 7.95 (d, *J* = 8.0 Hz, 2H), 7.83 (d, *J* = 8.0 Hz, 2H), 7.54 (d, *J* = 8.0 Hz, 2H), 7.29-7.19 (m, 6H), 6.44 (s, 1H), 6.32 (s, 1H), 3.65 (s, 2H), 3.06 (s, 3H), 2.31 (s, 3H), 1.29 (s, 9H); ^{13}C NMR (100 MHz, acetone-d$_6$): δ 168.47, 161.36, 160.97, 160.49, 157.90, 145.65, 139.03, 136.79, 136.45, 135.99, 133.16, 129.85, 129.59, 128.36, 123.74, 120.87, 120.72, 118.53, 118.44, 96.43, 87.68, 43.79, 42.32, 32.04, 20.06; LC-MS (ESI): *m/z* 610.2609 $[M+H]^+$.

S5.i. 2-(4-(6-morpholinopyrimidin-4-ylamino)phenyl)-N-(3-tert-butyl-1-methyl-1H-pyrazol-5-yl)acetamide (13i)

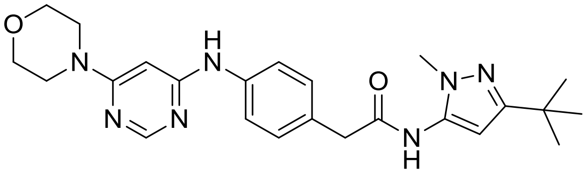

Sticky white solid (0.040 g, 28.16%); ^{1}H NMR (400 MHz, DMSO-d$_6$): δ 9.99 (s, 1H), 9.07 (s, 1H), 8.20 (s, 1H), 7.52 (d, *J* = 8.0 Hz, 2H), 7.23 (d, *J* = 8.0 Hz, 2H), 6.04 (s, 1H), 5.94 (s, 1H), 3.66 (t, *J* = 4.0 Hz, 4H), 3.59 (s, 2H), 3.56 (s, 3H), 3.43 (t, *J* = 4.0 Hz, 4H), 1.18 (s, 9H); ^{13}C NMR (100 MHz, DMSO-d$_6$): δ 169.67, 162.89, 161.43, 159.03, 157.75, 139.58, 136.72, 129.76, 129.13, 120.03, 95.42, 84.44, 66.21, 44.36, 41.99, 35.69, 32.21, 30.74; LC-MS (ESI): *m/z* 450.3030 $[M+H]^+$.

S5.j. 2-(4-(6-(4-methylpiperazin-1-yl)pyrimidin-4-ylamino)phenyl)-N-(3-tert-butyl-1-(4-bromophenyl)-1H-pyrazol-5-yl)acetamide (13j)

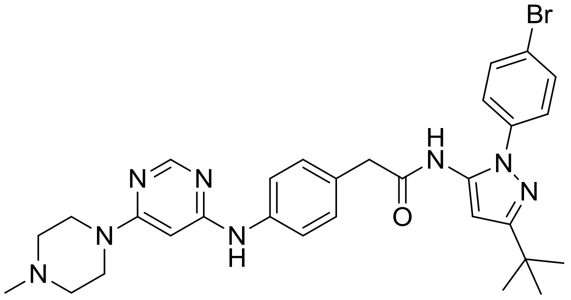

Colourless solid (0.056 g, 30.43%); m.p. 110-112 °C; ^{1}H NMR (400 MHz, acetone-d$_6$): δ 8.95 (s, 1H), 8.21 (s, 2H), 7.59 (d, *J* = 8.0 Hz, 2H), 7.54 (d, *J* = 12.0 Hz, 2H), 7.33 (d, *J* = 12.0 Hz, 2H), 7.23 (d, *J* = 12.0 Hz, 2H), 6.39 (s, 1H), 6.01 (s, 1H), 3.62 (s, 2H), 3.57-3.52 (m, 4H), 2.38 (t, *J* = 8.0 Hz, 4H), 2.23 (s, 3H), 1.27 (s, 9H); ^{13}C NMR (100 MHz, acetone-d$_6$): δ 168.83, 162.84, 161.60, 161.47, 157.55, 139.82, 138.34, 136.20, 132.06, 129.64, 128.38, 125.20, 120.09, 119.72, 97.80, 83.91, 54.40, 45.38, 43.65, 42.40, 32.08, 27.70; LC-MS (ESI): *m/z* 605.1965 [M+H]$^+$.

S5.k. 2-(4-(6-(4-(methylsulfonyl)phenylamino)pyrimidin-4-ylamino)phenyl)-N-(5-tert-butylisoxazol-3-yl)acetamide (13k)

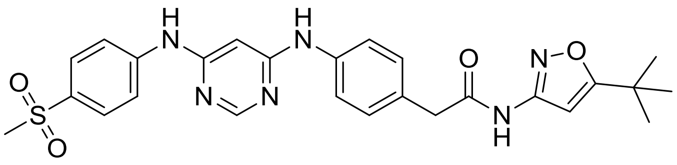

Yellow solid (0.055 g, 42.30%); m.p. 168-170 °C; ^{1}H NMR (400 MHz , DMSO-d$_6$): δ 11.15 (s, 1H), 9.68 (s, 1H), 9.26 (s, 1H), 8.34 (s, 1H), 7.86 (d, *J* = 8.0 Hz, 2H), 7.78 (d, *J* = 8.0 Hz, 2H), 7.46 (d, *J* = 8.0 Hz, 2H), 7.25 (d, *J* = 8.0 Hz, 2H), 6.55 (s, 1H), 6.23 (s, 1H), 3.60 (s, 2H), 3.12 (s, 3H), 1.26 (s, 9H); ^{13}C NMR (100 MHz, DMSO-d$_6$): δ 180.82, 170.02, 161.14, 160.30, 158.35, 158.06, 145.91, 139.06, 132.45, 129.90, 129.58, 128.53, 120.85, 118.82, 93.47, 88.26, 44.43, 42.32, 32.89, 28.71; LC-MS (ESI): *m/z* 521.1169 [M+H]$^+$.

S5.l. 2-(4-(6-(3-(trifluoromethyl)phenylamino)pyrimidin-4-yloxy)phenyl)-N-(5-tert-butylisoxazol-3-yl)acetamide (13l)

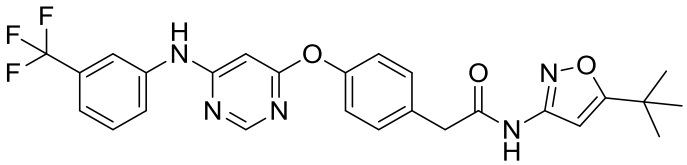

Colourless solid (0.050 g, 38.16%); m.p.120-122 °C; ^{1}H NMR (400 MHz, acetone-d_6): δ 10.16 (s, 1H), 9.06 (s, 1H), 8.37 (s, 1H), 8.20 (s, 1H), 7.89 (d, *J* = 8.0 Hz, 1H), 7.52 (t, *J* = 8.0 Hz, 1H), 7.46 (d, *J* = 8.0 Hz, 2H), 7.32 (d, *J* = 8.0 Hz, 1H), 7.14 (d, *J* = 8.0 Hz, 2H), 6.65 (s, 1H), 6.18 (s, 1H), 3.82 (s, 2H), 1.31 (s, 9H); ^{13}C NMR (100 MHz, acetone-d_6): δ 180.77, 170.03, 162.61, 158.13, 158.04, 152.00, 140.98, 132.44, 130.62, 129.66, 123.01, 121.52, 118.60, 118.56, 118.52, 116.00, 115.96, 92.95, 89.73, 42.25, 32.55, 27.93; LC-MS (ESI): *m/z* 512.2689 $[M+H]^+$.

S5.m. 2-(4-(6-(3-(trifluoromethyl)phenylamino)pyrimidin-4-ylamino)phenyl)-N-(5-tert-butylisoxazol-3-yl)acetamide (13m)

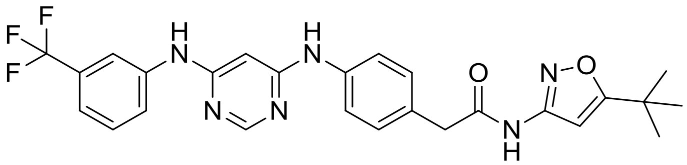

White solid (0.051 g, 38.93%); m.p 254-256 °C; ^{1}H NMR (400 MHz, acetone-d_6): δ 10.04 (s, 1H), 8.68 (s, 1H), 8.33 (d, *J* = 8.0 Hz, 2H), 8.18 (s, 1H), 7.86 (d, *J* = 8.0 Hz, 1H), 7.52-7.46 (m, 3H), 7.34 (d, *J* = 8.0 Hz, 2H), 7.26 (d, *J* = 8.0 Hz, 1H), 6.64 (s, 1H), 6.24 (s, 1H), 3.75 (s, 2H), 1.30 (s, 9H); ^{13}C NMR (100 MHz, acetone-d6): δ 180.69, 169.22, 161.29, 160.84, 158.07, 157.92, 141.71, 138.95, 129.73, 129.51, 122.63, 120.93, 120.78, 117.75, 117.71, 117.67, 115.62, 92.91, 86.58, 42.41, 32.53, 27.92; LC-MS (ESI): *m/z* 511.1696 $[M+H]^+$.

S5.n. 2-(4-(6-(3-(pyrrolidin-1-yl)propylamino)pyrimidin-4-ylamino)phenyl)-N-(5-tert-butylisoxazol-3-yl)acetamide (13n)

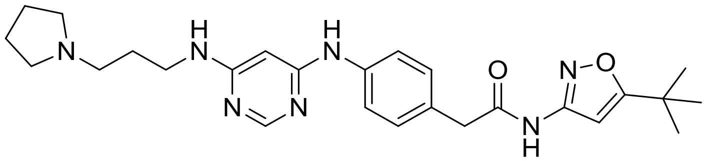

White solid (0.049 g, 36.56%); m.p. 154-156 °C; ^{1}H NMR (400 MHz, acetone-d_6): δ 10.02 (s, 1H), 8.09 (s, 1H), 8.04 (s, 1H), 7.54-7.51 (m, 2H), 7.31 (d, *J* = 8.0 Hz, 2H), 6.64 (s, 1H), 6.28 (s, 1H), 5.83 (s, 1H), 3.73 (s, 2H), 3.33 (s, 2H), 2.53 (t, *J* = 8.0 Hz, 2H), 2.47 (s, 4H), 1.77 (t, *J* = 8.0 Hz, 2H), 1.73-1.70 (m, 4H), 1.31 (s, 9H); ^{13}C NMR (100 MHz, acetone-d_6): δ 169.83, 169.26, 163.42, 157.88, 139.71, 130.29, 129.55, 128.69, 120.18, 120.04, 92.91, 89.97, 53.74, 53.68, 42.42, 39.56, 37.71, 32.52, 28.92, 23.21; LC-MS (ESI): *m/z* 478.1989 $[M+H]^+$.

S5.o. 2-(4-(6-(4-bromophenoxy)pyrimidin-4-ylamino)phenyl)-N-(5-tert-butylisoxazol-3-yl)acetamide (13o)

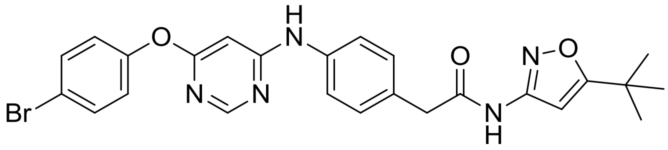

White solid (0.046 g, 35.38%); m.p. 188-190 °C; ^{1}H NMR (400 MHz, acetone-d$_6$): δ 10.06 (s, 1H), 8.77 (s, 1H), 8.29 (s, 1H), 7.60 (d, *J* = 8.0 Hz, 4H), 7.37 (d, *J* = 8.0 Hz, 2H), 7.16 (d, *J* = 8.0 Hz, 2H), 6.65 (s, 1H), 6.22 (s, 1H), 3.77 (s, 2H), 1.31 (s, 9H); ^{13}C NMR (100 MHz, acetone-d$_6$): δ; 180.68, 169.42, 163.08, 158.07, 152.52, 138.54, 132.48, 129.98, 129.73, 129.45, 123.77, 120.63, 120.49, 117.38, 92.93, 88.88, 42.40, 32.53, 27.93; LC-MS (ESI): *m/z* 522.1923 $[M+H]^+$.

S5.p. 2-(4-(6-morpholinopyrimidin-4-ylamino)phenyl)-N-(5-tert-butylisoxazol-3-yl)acetamide (13p)

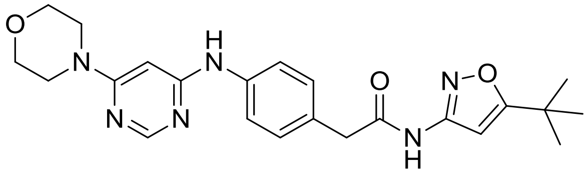

White solid (0.054 g, 39.13%); m.p. 218-220 °C; ^{1}H NMR (400 MHz, DMSO-d$_6$): δ 11.13 (s, 1H), 9.07 (s, 1H), 8.19 (s, 1H), 7.49 (d, *J* = 12.0 Hz, 2H), 7.21 (d, *J* = 8.0 Hz, 2H), 6.56 (s, 1H), 5.94 (s, 1H), 3.66 (t, *J* = 4.0 Hz, 4H), 3.58 (s, 2H), 3.43 (t, *J* = 4.0 Hz, 4H), 1.26 (s, 9H); ^{13}C NMR (100 MHz, DMSO-d$_6$): δ 180.82, 170.11, 162.89, 161.39, 158.38, 157.74, 139.63, 129.80, 128.79, 120.04, 93.51, 84.41, 66.20, 44.35, 42.33, 32.90, 28.74; LC-MS (ESI): *m/z* 437.1940 $[M+H]^+$.

S5.q. 2-(4-(6-(dimethylamino)pyrimidin-4-yloxy)phenyl)-N-(5-tert-butylisoxazol-3-yl)acetamide (13q)

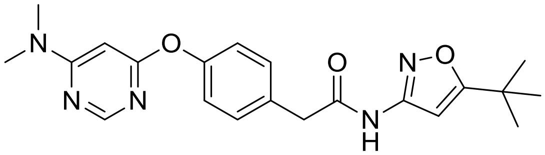

Colourless solid (0.045 g, 31.25%); m.p. 170-172 °C; ^{1}H NMR (400 MHz, acetone-d$_6$): δ 10.14 (s, 1H), 8.11 (s, 1H), 7.40 (d, *J* = 8.0 Hz, 2H), 7.07 (d, *J* = 8.0 Hz, 2H), 6.65 (s, 1H), 5.98 (s, 1H),

3.79 (s, 2H), 3.07 (s, 6H), 1.30 (s, 9H); ^{13}C NMR (100 MHz, acetone-d$_6$): δ 180.72, 169.80, 169.09, 164.53, 158.06, 157.26, 152.50, 131.68, 130.23, 121.42, 92.95, 85.52, 42.32, 36.36, 32.55, 27.95; LC-MS (ESI): *m/z* 396.2055 [M+H]$^+$.

S5.r. 2-(4-(6-(methylamino)pyrimidin-4-ylamino)phenyl)-N-(3-(trifluoromethyl)phenyl) acetamide (13r)

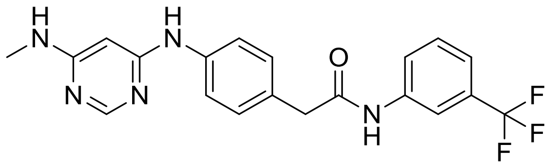

White solid (0.050 g, 32.25%); m.p. 216-218 °C; ^{1}H NMR (400 MHz, acetone-d$_6$): δ 9.60 (s, 1H), 8.17 (s, 1H), 8.08 (s, 1H), 7.82 (d, *J* = 8.0 Hz, 1H), 7.53-7.48 (m, 4H), 7.36 (d, *J* = 8.0 Hz, 1H), 7.29 (d, *J* = 8.0 Hz, 2H), 5.98 (s, 1H), 5.78 (s, 1H), 3.67 (s, 2H), 2.82 (d, *J* = 4.0 Hz, 3H); ^{13}C NMR (100 MHz, acetone-d$_6$): δ 169.78, 164.00, 157.78, 140.25, 139.62, 129.63, 129.52, 128.97, 122.47, 122.38, 120.17, 120.03, 119.56, 119.52, 115.48, 82.71, 43.19, 27.19; LC-MS (ESI): *m/z* 402.2207 [M+H]$^+$.

S5.s. 2-(4-(6-(4-(methylsulfonyl)phenylamino)pyrimidin-4-ylamino)phenyl)-N-(3-(trifluoromethyl)phenyl)acetamide (13s)

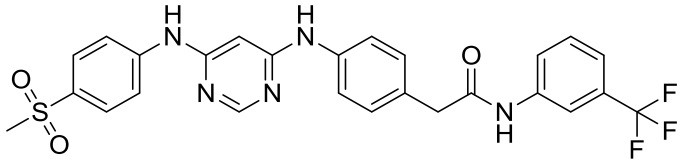

White solid (0.045 g, 33.33%); m.p. 225-227 °C; ^{1}H NMR (400 MHz, DMSO-d$_6$): δ 10.49 (s, 1H), 9.69 (s, 1H), 9.27 (s, 1H), 8.34 (s, 1H), 8.10 (s, 1H), 7.86 (d, *J* = 8.0 Hz, 2H), 7.78 (d, *J* = 8.0 Hz, 3H), 7.53 (t, *J* = 8.0 Hz, 1H), 7.48 (d, *J* = 8.0 Hz, 2H), 7.38 (d, *J* = 8.0 Hz, 1H), 7.28 (d, *J* = 8.0 Hz, 2H), 6.23 (s, 1H), 3.62 (s, 2H), 3.12 (s, 3H); ^{13}C NMR (100 MHz, DMSO-d$_6$): δ 170.38, 161.19, 160.33, 158.10, 145.95, 140.40, 139.07, 132.47, 130.43, 129.96, 129.88, 129.70, 128.56, 123.00, 120.87, 119.93, 118.65, 115.50, 115.46, 88.30, 44.46, 43.16; LC-MS (ESI): *m/z* 542.1709 [M+H]$^+$.

S5.t. 2-(4-(6-(3-(pyrrolidin-1-yl)propylamino)pyrimidin-4-ylamino)phenyl)-N-(4-(trifluoromethoxy)phenyl)acetamide (13t)

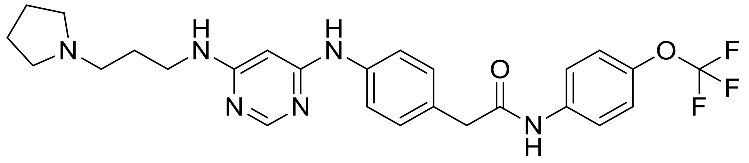

White solid (0.044 g, 30.55%); m.p. 212-214 °C; ^{1}H NMR (400 MHz, acetone-d_6): δ 9.52 (s, 1H), 8.06 (d, *J* = 8.0 Hz, 2H), 7.77 (d, *J* = 8.0 Hz, 2H), 7.51-7.48 (m, 2H), 7.28 (d, *J* = 8.0 Hz, 2H), 7.23 (d, *J* = 12.0 Hz, 2H), 6.27 (d, *J* = 4.0 Hz, 1H), 5.80 (s, 1H), 3.64 (s, 2H), 3.31 (s, 2H), 2.50 (t, *J* = 8.0 Hz, 2H), 2.44 (t, *J* = 8.0 Hz, 4H), 1.74 (t, *J* = 4.0 Hz, 2H), 1.70-1.67 (m, 4H); ^{13}C NMR (100 MHz, acetone-d_6): δ 169.36, 163.38, 157.86, 144.17, 139.50, 138.58, 129.50, 121.46, 120.39, 120.30, 120.23, 120.09, 119.29, 82.98, 53.70, 53.76, 43.16, 43.11, 39.48, 23.20; LC-MS (ESI): *m/z* 515.1573 $[M+H]^+$.

S5.u. 2-(4-(6-(2,3-dihydrobenzo[b][1,4]dioxin-5-ylamino)pyrimidin-4-ylamino)phenyl)-N-(4-(trifluoromethoxy)phenyl)acetamide (13u)

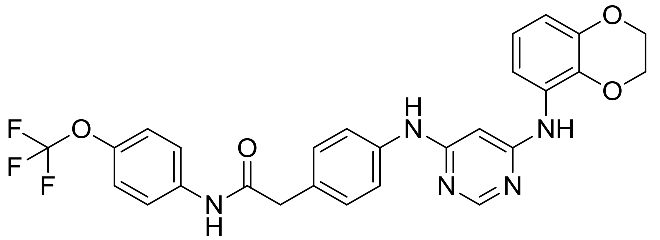

White solid (0.047 g, 33.09%); m.p 275-277 °C; ^{1}H NMR (400 MHz, acetone-d_6): δ 9.69 (s, 1H), 8.26 (s, 1H), 8.21 (s, 1H), 8.08 (s, 1H), 7.81 (d, *J* = 8.0 Hz, 2H), 7.54-7.47 (m, 2H), 7.32 (d, *J* = 8.0 Hz, 2H), 7.25 (d, *J* = 8.0 Hz, 2H), 7.18-7.16 (m, 1H), 6.93-6.89 (m, 1H), 6.76 (d, *J* = 8.0 Hz, 1H), 6.13 (s, 1H), 4.26-4.22 (m, 4H), 3.68 (s, 2H); ^{13}C NMR (100 MHz, acetone-d_6): δ 169.47, 161.49, 161.10, 157.95, 143.55, 139.57, 139.24, 138.74, 133.86, 129.55, 122.23, 121.44, 120.40, 120.37, 120.31, 120.22, 116.90, 114.34, 110.32, 84.88, 64.39, 64.13, 43.13; LC-MS (ESI): *m/z* 538.2409 $[M+H]^+$.

S5.v. 2-(4-(6-(methylamino)pyrimidin-4-ylamino)phenyl)-N-(4-(methylsulfonyl)phenyl) acetamide (13v)

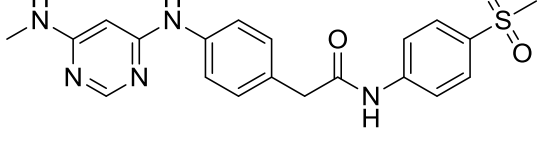

Yellow solid (0.044 g, 27.67%); m.p 220-222 °C; ^{1}H NMR (400 MHz, acetone-d$_6$): δ 9.79 (s, 1H), 8.16 (s, 1H), 8.08 (s, 1H), 7.89 (d, *J* = 8.0 Hz, 2H), 7.84 (d, *J* = 8.0 Hz, 2H), 7.51 (d, *J* = 8.0 Hz, 2H), 7.29 (d, *J* = 8.0 Hz, 2H), 6.05 (d, *J* = 8.0 Hz, 1H), 5.78 (s, 1H), 3.69 (s, 2H), 3.05 (s, 3H), 2.82 (d, *J* = 4.0 Hz, 3H); ^{13}C NMR (100 MHz, acetone-d$_6$): δ 170.04, 163.99, 157.77, 144.00, 139.61, 135.33, 129.57, 128.89, 128.35, 120.25, 118.96, 118.88, 82.63, 43.65, 43.18, 27.23; LC-MS (ESI): *m/z* 412.1931 [M+H]$^+$.

S5.w. N-(4-fluorobenzyl)-2-(4-(6-(3-(pyrrolidin-1-yl)propylamino)pyrimidin-4-yloxy)phenyl)acetamide (13w)

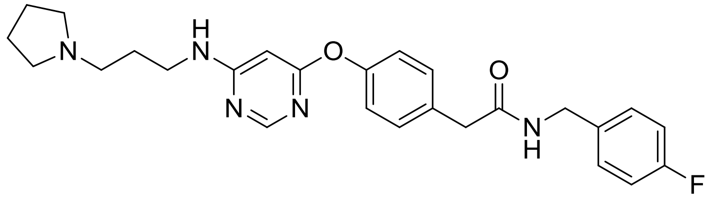

Sticky colourless solid (0.042 g, 32.30%); ^{1}H NMR (400 MHz, DMSO-d$_6$): δ 8.80 (s, 1H), 8.47 (t, *J* = 8.0 Hz, 1H), 8.03 (s, 1H), 7.38 (d, *J* = 8.0 Hz, 1H), 7.24 (dd, *J* = 4.0, 8.0 Hz, 2H), 7.13-7.07 (m, 4H), 6.85 (t, *J* = 8.0 Hz, 1H), 5.71 (s, 1H), 4.20 (d, *J* = 4.0 Hz, 2H), 3.36 (s, 2H), 3.18 (s, 3H), 2.54 (s, 5H), 1.68-1.64 (m, 6H); ^{13}C NMR (100 MHz, DMSO-d$_6$): δ 170.40, 162.66, (d, $^{1}J_{CF}$=241 Hz), 159.90, 157.59, 139.12, 135.72, 129.28, 129.20, 129.19, 129.10, 119.64, 115.06, (d, $^{2}J_{CF}$=21 Hz), 83.66, 53.50, 53.06, 41.71, 41.46, 38.53, 27.69, 22.98; LC-MS (ESI): *m/z* 464.1752 [M+H]$^+$.

S5.x. 2-(4-(6-(methylamino)pyrimidin-4-yloxy)phenyl)-N-(4-tert-butylphenyl)acetamide (13x)

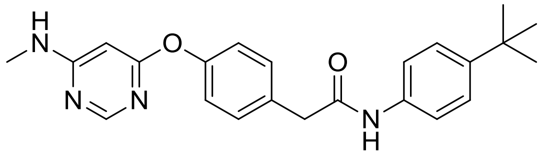

White solid (0.046 g, 30.66%); m.p. 208-210 °C; ^{1}H NMR (400 MHz, DMSO-d$_6$): δ 10.12 (s, 1H), 7.62 (t, *J* = 8.0 Hz, 2H), 7.56-7.51 (m, 3H), 7.37 (d, *J* = 8.0 Hz, 2H), 7.31 (d, *J* = 12.0 Hz, 3H), 7.08 (d, *J* = 8.0 Hz, 2H), 3.63 (s, 2H), 2.76 (s, 3H), 1.24 (s, 9H); ^{13}C NMR (100 MHz, DMSO-d$_6$): δ 169.25, 151.95, 145.97, 137.04, 132.47, 131.95, 130.73, 129.24, 129.12, 125.73, 121.77, 119.30, 85.70, 43.01, 42.12, 34.41, 31.61; LC-MS (ESI): *m/z* 391.2869 [M+H]$^+$.

S5.y. 2-(4-(6-(3-(piperidin-1-yl)propylamino)pyrimidin-4-ylamino)phenyl)-N-(4-(trifluoromethoxy)phenyl)acetamide (13y)

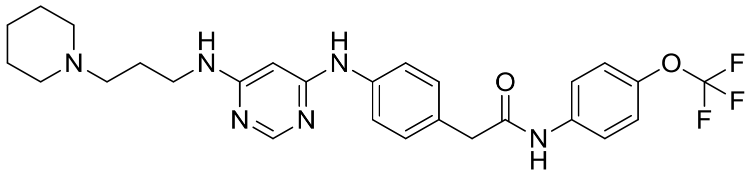

Sticky pale yellow solid (0.041 g, 28.67%); ^{1}H NMR (400 MHz, DMSO-d$_6$): δ 10.40 (s, 1H), 8.89 (s, 1H), 8.06 (s, 1H), 7.69 (d, *J* = 8.0 Hz, 2H), 7.40 (d, *J* = 8.0 Hz, 2H), 7.27 (d, *J* = 8.0 Hz, 2H), 7.20 (d, *J* = 8.0 Hz, 2H), 6.97 (s, 1H), 5.75 (s, 1H), 3.55 (s, 2H), 2.95 (s, 5H), 1.85 (s, 3H), 1.69 (s, 5H), 1.49 (s, 3H); ^{13}C NMR (100 MHz, DMSO-d$_6$): δ 170.09, 162.99, 160.48, 158.03, 143.86, 139.62, 138.89, 129.78, 129.32, 122.03, 120.80, 120.30, 119.27, 83.97, 54.45, 52.59, 43.05, 31.12, 24.26, 23.13, 21.92; LC-MS (ESI): *m/z* 529.1870 [M+H]$^+$.

S5.z. 2-(4-(6-(methylamino)pyrimidin-4-ylamino)phenyl)-N-(4-(trifluoromethoxy)phenyl) acetamide (13z)

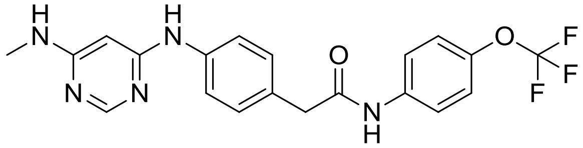

White solid (0.052 g, 32.29%); m.p 220-222 °C; ^{1}H NMR (400 MHz, acetone-d$_6$): δ 9.47 (s, 1H), 8.08 (s, 2H), 7.76 (d, *J* = 8.0 Hz, 2H), 7.51 (d, *J* = 8.0 Hz, 2H), 7.25 (q, *J* = 8.0, 8.0 Hz, 4H), 5.99 (s, 1H), 5.78 (s, 1H), 3.64 (s, 2H), 2.82 (d, *J* = 4.0 Hz, 3H); ^{13}C NMR (100 MHz, acetone-d$_6$): δ 169.41, 164.01, 157.79, 144.18, 139.58, 138.65, 129.49, 129.14, 121.47, 120.38, 120.29, 120.18, 120.04, 82.71, 43.16, 27.19; LC-MS (ESI): *m/z* 418.2319 [M+H]$^+$.

S5.aa. 2-(4-(6-(4-(methylsulfonyl)phenylamino)pyrimidin-4-yloxy)phenyl)-N-(3-fluorophenyl)acetamide (13aa)

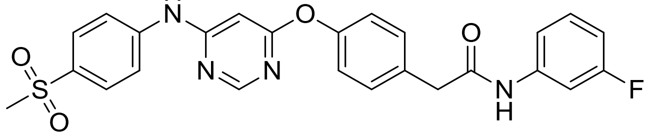

Brown solid (0.038 g, 30.89%); m.p. 228-230 °C; ^{1}H NMR (400 MHz, acetone-d$_6$): δ 9.67 (s, 1H), 9.29 (s, 1H), 8.40 (s, 1H), 7.97 (d, *J* = 8.0 Hz, 2H), 7.84 (d, *J* = 8.0 Hz, 2H), 7.70 (d, *J* =

12.0 Hz, 1H), 7.45 (d, J = 8.0 Hz, 2H), 7.34-7.27 (m, 2H), 7.13 (d, J = 8.0 Hz, 2H), 6.80 (t, J = 8.0 Hz, 1H), 6.24 (s, 1H), 3.75 (s, 2H), 3.06 (s, 3H), ^{13}C NMR (100 MHz, acetone-d$_6$): δ 170.13, 169.25, 169.16, 163.99 (d, $^1J_{CF}$ = 240 Hz), 162.35, 158.09, 151.84, 144.93, 144.84, 132.91, 130.61, 130.16 (d, $^3J_{CF}$ = 9.0 Hz), 128.39, 121.47, 118.95, 114.72, 109.78, (d, $^2J_{CF}$ = 22 Hz), 106.28, (d, $^2J_{CF}$ = 26 Hz), 90.52, 43.73, 42.98; LC-MS (ESI): *m/z* 493.1870 [M+H]$^+$.

S5.ab. 2-(4-(6-(3-(pyrrolidin-1-yl)propylamino)pyrimidin-4-ylamino)phenyl)-N-(3-(trifluoromethyl)phenyl)acetamide (13ab)

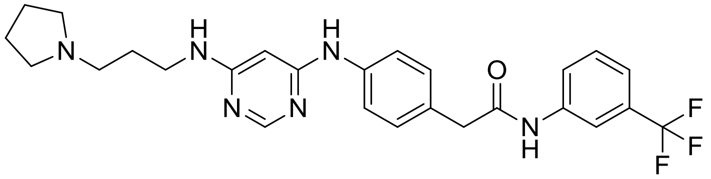

Sticky pale yellow solid (0.045 g, 32.14%); ^{1}H NMR (400 MHz , DMSO-d$_6$): δ 10.51 (s, 1H), 8.84 (s, 1H), 8.08 (s, 1H), 8.03 (s, 1H), 7.76 (d, J = 8.0 Hz, 1H), 7.51 (t, J = 8.0 Hz, 1H), 7.41 (d, J = 8.0 Hz, 2H), 7.35 (d, J = 8.0 Hz, 1H), 7.20 (d, J = 8.0 Hz, 2H), 6.85 (s, 1H), 5.72 (s, 1H), 3.57 (s, 2H), 3.18 (s, 2H), 2.54 (s, 6H), 1.64-1.59 (m, 6H); ^{13}C NMR (100 MHz, DMSO-d$_6$): δ 167.16, 159.79, 154.71, 137.13, 136.51, 127.08, 126.67, 126.47, 125.60, 122.56, 119.85, 119.67, 116.84, 116.56, 112.18, 89.99, 50.59, 50.14, 39.82, 35.64, 24.75, 20.09; LC-MS (ESI): *m/z* 499.1751 [M+H]$^+$.

S5.ac. 2-(4-(6-(4-(methylsulfonyl)phenylamino)pyrimidin-4-ylamino)phenyl)-N-(3-fluorophenyl)acetamide (13ac)

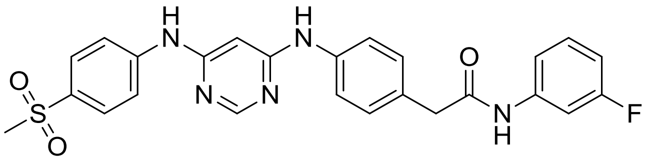

Sticky white solid (0.047 g, 38.21%); ^{1}H NMR (400 MHz, DMSO-d$_6$): δ 10.38 (s, 1H), 9.70 (s, 1H), 9.27 (s, 1H), 8.34 (s, 1H), 7.87 (d, J = 8.0 Hz, 2H), 7.78 (d, J = 12.0 Hz, 2H), 7.60 (d, J = 12.0 Hz, 1H), 7.48 (d, J = 8.0 Hz, 2H), 7.34-7.27 (m, 4H), 6.85 (t, J = 8.0 Hz, 1H), 6.25 (s, 1H), 3.60 (s, 2H), 3.13 (s, 3H); ^{13}C NMR (100 MHz, DMSO-d$_6$): δ 170.13, 163.72, 161.32, 161.17, 160.31 (d, $^1J_{CF}$ = 223 Hz), 145.94, 141.42, 139.00, 132.43, 130.81, 130.00, 129.92, 128.54,

120.87, 115.21, 115.19, 110.14 (d, $^{2}J_{CF}$ = 21 Hz), 106.36 (d, $^{2}J_{CF}$ = 26 Hz), 88.26, 44.44, 43.13; LC-MS (ESI): *m/z* 492.1703 $[M+H]^{+}$.

S5.ad. 2-(4-(6-(methylamino)pyrimidin-4-yloxy)phenyl)-N-(4-fluorophenyl)acetamide (13ad)

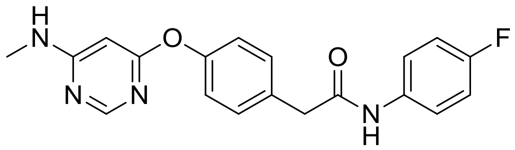

White solid (0.048 g, 35.55%); m.p. 124-126 °C; ^{1}H NMR (400 MHz, acetone-d_6): δ 9.43 (s, 1H), 8.09 (s, 1H), 7.72-7.66 (m, 2H), 7.41 (d, *J* = 8.0 Hz, 2H), 7.10-7.04 (m, 4H), 6.47 (s, 1H), 5.83 (s, 1H), 3.71 (s, 2H), 2.90 (d, *J* = 8.0 Hz, 3H); ^{13}C NMR (100 MHz, acetone-d_6): δ 168.78, 159.82 (d, $^{1}J_{CF}$ = 241 Hz) 157.94, 152.27, 135.79, 132.45, 130.22, 121.37, 120.94, 120.86, 120.77, 115.12 (d, $^{2}J_{CF}$ = 23 Hz), 83.01, 42.97, 27.22; LC-MS (ESI): *m/z* 353.1776 $[M+H]^{+}$.

S5.ae. 2-(4-(6-(4-(methylsulfonyl)phenylamino)pyrimidin-4-ylamino)phenyl)-N-cyclopropylacetamide (13ae)

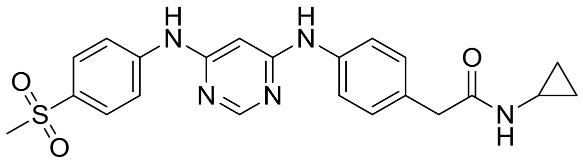

Yellow solid (0.036 g, 33.02%); m.p. 110-112 °C; ^{1}H NMR (400 MHz, DMSO-d_6): δ 9.69 (s, 1H), 9.22 (s, 1H), 8.34 (s, 1H), 8.08 (s, 1H), 7.86 (d, *J* = 12.0 Hz, 2H), 7.78 (d, *J* = 12.0 Hz, 2H), 7.43 (d, *J* = 8.0 Hz, 2H), 7.17 (d, *J* = 8.0 Hz, 2H), 6.23 (s, 1H), 3.29 (s, 2H), 3.13 (s, 3H), 2.62-2.58 (m, 1H), 0.62-0.57 (m, 2H), 0.40-0.36 (m, 2H); ^{13}C NMR (100 MHz, DMSO-d_6): δ 171.92, 161.41, 160.50, 158.28, 146.14, 138.85, 132.62, 131.07, 129.90, 128.73, 121.07, 118.81, 88.32, 44.64, 42.22, 23.00, 6.28; LC-MS (ESI): *m/z* 438.1555 $[M+H]^{+}$.

S5.af. 2-(4-(6-(3-(trifluoromethyl)phenylamino)pyrimidin-4-ylamino)phenyl)-N-cyclopropylacetamide (13af)

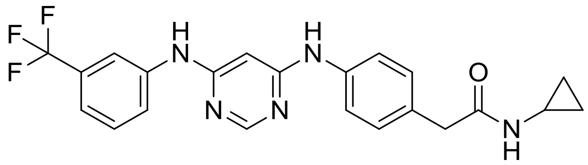

Sticky pale yellow solid (0.040 g, 36.36%); ^{1}H NMR (400 MHz, DMSO-d_6): δ 9.46 (s, 1H), 9.14 (s, 1H), 8.29 (s, 1H), 8.09-8.06 (m, 2H), 7.80 (d, *J* = 8.0 Hz, 1H), 7.46 (t, *J* = 8.0 Hz, 1H), 7.40 (d, *J* = 8.0 Hz, 2H), 7.22 (d, *J* = 8.0 Hz, 1H), 7.14 (d, *J* = 8.0 Hz, 2H), 6.13 (s, 1H), 3.26 (s, 2H), 2.61-2.54 (m, 1H), 0.59-0.55 (m, 2H), 0.37-0.33 (m, 2H); ^{13}C NMR (100 MHz, DMSO-d_6): δ 171.76, 161.10, 160.60, 158.12, 142.03, 138.79, 130.75, 130.18, 129.99, 129.69, 123.36, 122.87, 120.79, 117.75, 115.31, 87.30, 42.05, 22.83, 6.11; LC-MS (ESI): *m/z* 428.1801 $[M+H]^+$.

S5.ag. 2-(4-(6-(2,3-dihydrobenzo[b][1,4]dioxin-5-ylamino)pyrimidin-4-ylamino)phenyl)-N-cyclopropylacetamide (13ag)

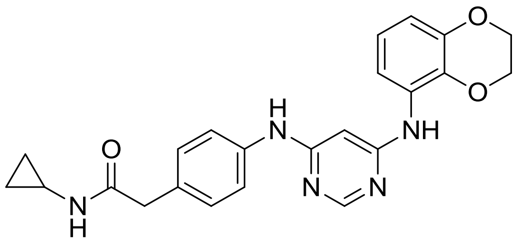

White solid (0.041 g, 37.27%); m.p. 234-226 °C; ^{1}H NMR (400 MHz, acetone-d_6): δ 8.21 (s, 1H), 8.19 (s, 1H), 8.03 (s, 1H), 7.48 (dd, *J* = 4.0, 8.0 Hz, 2H), 7.22 (d, *J* = 8.0 Hz, 3H), 7.17-7.15 (m, 1H), 6.92-6.89 (m, 1H), 6.77 (d, *J* = 8.0 Hz, 1H), 6.10 (s, 1H), 4.27-4.23 (m, 4H), 3.37 (s, 2H), 2.73-2.66 (m, 1H), 0.65-0.60 (m, 2H), 0.44-0.40 (m, 2H); ^{13}C NMR (100 MHz, acetone-d_6 + MeOD): δ 172.45, 161.38, 161.01, 157.88, 143.57, 139.71, 138.81, 133.53, 130.12, 129.34, 120.39, 116.92, 114.47, 110.45, 84.60, 64.37, 64.13, 41.96, 22.27, 5.37; LC-MS (ESI): *m/z* 418.1931 $[M+H]^+$.

S5.ah. 2-(4-(6-(4-methylpiperazin-1-yl)pyrimidin-4-ylamino)phenyl)-N-cyclopropyl acetamide (13ah)

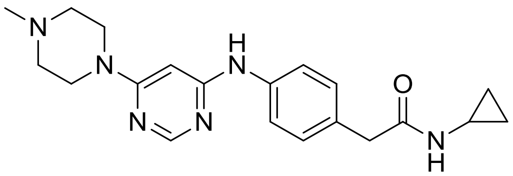

Sticky colourless solid (0.042 g, 38.18%); ^{1}H NMR (400 MHz, DMSO-d_6): δ 8.95 (s, 1H), 8.13 (s, 1H), 8.04 (s, 1H), 7.42 (d, *J* = 8.0 Hz, 2H), 7.09 (d, *J* = 8.0 Hz, 2H), 5.90 (s, 1H), 3.43 (t, *J* = 4.0 Hz, 4H), 3.32 (s, 2H), 2.60-2.53 (m, 1H), 2.32 (t, *J* = 4.0 Hz, 4H), 2.17 (s, 3H), 0.58-0.54 (m, 2H), 036-0.34 (m, 2H); ^{13}C NMR (100 MHz, DMSO-d_6): δ 171.82, 162.61, 161.42, 157.76,

139.31, 129.93, 129.56, 119.94, 84.26, 54.58, 46.20, 43.89, 42.03, 22.81, 6.10; LC-MS (ESI): *m/z* 367.2036 $[M+H]^+$.

S5.ai. 2-(4-(6-morpholinopyrimidin-4-ylamino)phenyl)-N-cyclopropylacetamide (13ai)

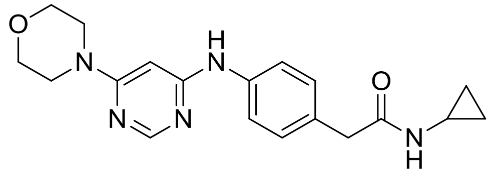

Yellow solid (0.038 g, 33.92%); m.p. 110-112 °C; ^{1}H NMR (400 MHz, DMSO-d_6): δ 9.00 (s, 1H), 8.15 (s, 1H), 8.04 (s, 1H), 7.43 (d, *J* = 12.0 Hz, 2H), 7.10 (d, *J* = 8.0 Hz, 2H), 5.90 (s, 1H), 3.63 (t, *J* = 4.0 Hz, 4H), 3.40 (t, *J* = 4.0 Hz, 4H), 3.23 (s, 2H), 2.60-2.53 (m, 1H), 0.59-0.59 (m, 2H), 0.36-0.33 (m, 2H); ^{13}C NMR (100 MHz, DMSO-d_6): δ 171.82, 162.88, 161.43, 157.74, 139.23, 130.03, 129.57, 120.01, 84.30, 66.20, 44.36, 42.02, 22.81, 6.10; LC-MS (ESI): *m/z* 354.3063 $[M+H]^+$.

S5.aj. 2-(4-(6-(butylamino)pyrimidin-4-yloxy)phenyl)-N-(4-tert-butylthiazol-2-yl) acetamide (13aj)

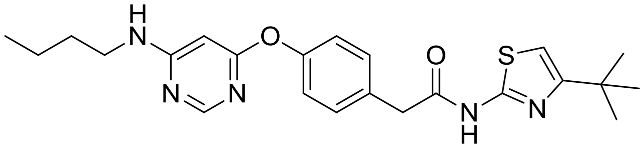

Colourless solid (0.055 g, 37.93%); m.p. 102-104 °C; ^{1}H NMR (400 MHz, acetone-d_6): δ 11.09 (s, 1H), 8.07 (s, 1H), 7.44 (d, *J* = 8.0 Hz, 2H), 7.08 (d, *J* = 8.0 Hz, 2H), 6.64 (s, 1H), 6.51 (s, 1H), 5.80 (s, 1H), 3.90 (s, 2H), 3.34 (s, 2H), 1.56 (t, *J* = 8.0 Hz, 2H), 1.40-1.35 (m, 2H), 1.23 (s, 9H), 0.90 (t, *J* = 4.0 Hz, 3H); ^{13}C NMR (100 MHz, acetone-d_6): δ 168.72, 165.14, 160.68, 158.03, 157.10, 152.48, 131.64, 130.41, 130.21, 121.51, 104.37, 85.55, 41.53, 40.50, 34.07, 31.27, 27.92, 19.78, 13.15; LC-MS (ESI): *m/z* 440.2009 $[M+H]^+$.

S5.ak. N-(2,4-dimethoxybenzyl)-2-(4-(6-(1-benzylpiperidin-4-ylamino)pyrimidin-4-yloxy)phenyl)acetamide (13ak)

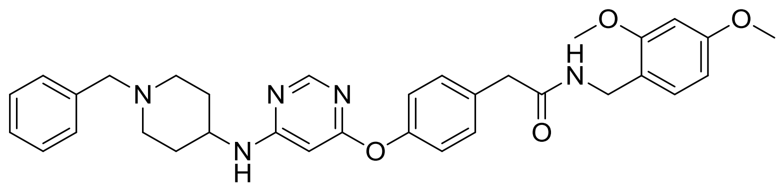

Yellow solid (0.048 g, 35.55%); m.p. 102-104 °C; ^{1}H NMR (400 MHz, acetone-d_6): δ 8.80 (s, 1H), 7.35-7.27 (m, 6H), 7.23-7.20 (m, 1H), 7.10 (d, *J* = 8.0 Hz, 1H), 7.02 (d, *J* = 8.0 Hz, 2H), 6.50 (d, *J* = 4.0 Hz, 1H), 6.46 (d, *J* = 8.0 Hz, 1H), 6.43 (d, *J* = 4.0 Hz, 1H), 6.41 (d, *J* = 4.0 Hz, 1H), 5.77 (s, 1H), 4.28 (d, *J* = 4.0 Hz, 2H), 3.77 (s, 3H), 3.75 (s, 3H), 3.52 (s, 2H), 3.47 (s, 2H), 3.41-3.36 (m, 1H), 2.81 (s, 2H), 2.11 (d, *J* = 8.0 Hz, 2H), 1.94 (d, *J* = 12.0 Hz, 2H), 1.57-1.49 (m, 2H); ^{13}C NMR (100 MHz, acetone-d_6): δ 169.71, 164.37, 160.40, 158.35, 158.13, 152.06, 139.09, 133.22, 130.27, 129.46, 129.44, 128.71, 128.04, 126.75, 121.30, 119.18, 104.04, 98.06, 62.62, 54.81, 54.67, 52.17, 42.11, 42.06, 37.90, 37.77, 31.98; LC-MS (ESI): *m/z* 568.1732 $[M+H]^+$.

S5.al. 2-(4-(6-(4-(methylsulfonyl)phenylamino)pyridin-2-ylamino)phenyl)-N- (3-tert-butyl-1-methyl-1H-pyrazol-5-yl)acetamide (18)

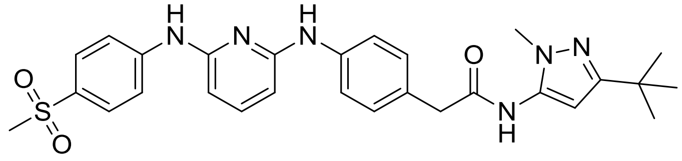

Sticky white solid (0.040 g, 29.85%); ^{1}H NMR (400 MHz, DMSO-d_6): δ 9.98 (s, 1H), 9.39 (s, 1H), 8.85 (s, 1H), 7.80 (d, *J* = 8.0 Hz, 2H), 7.67 (d, *J* = 8.0 Hz, 2H), 7.46-7.39 (m, 3H), 7.20 (d, *J* = 12.0 Hz, 2H), 6.31 (dd, *J* = 8.0, 12.0 Hz, 2H), 6.02 (s, 1H), 3.59 (s, 2H), 3.54 (s, 3H), 3.08 (s, 3H), 1.15 (s, 9H); ^{13}C NMR (100 MHz, DMSO-d_6): δ 169.78, 159.01, 154.97, 153.59, 146.88, 140.39, 139.28, 136.74, 130.86, 129.69, 128.41, 128.30, 119.60, 117.40, 101.70, 101.38, 95.42, 44.50, 41.97, 35.69, 32.21, 30.75; LC-MS (ESI): *m/z* 533.2224 $[M+H]^+$.

S6. Synthesis of tert-butyl 4-(6-chloropyrimidin-4-ylamino)phenylcarbamate (20)

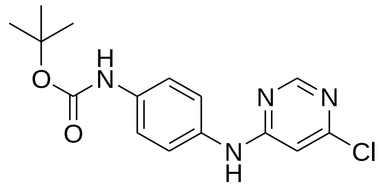

To the mixture of tert-butyl 4-aminophenylcarbamate **19** (2.09 g, 10.06 mmol) and 4,6-dichloropyrimidine **6** (1 g, 6.71 mmol) in EtOH (20 mL) was added triethyl amine (1.17g, 11.58 mmol) The reaction mixture was stirred at 80 °C for 12 h. After completion of reaction as indicate by TLC, the solvent was removed under reduced pressure. The crude product thus obtained was purified by silica gel (mesh 100-200) flash chromatography with hexanes/EtOAc (1:3) to afford **20** as white solid (2.85 g, 89.06%); m.p. 155-157 °C; ^{1}H NMR (400 MHz, $CDCl_3$): δ 8.41 (s, 1H), 7.43 (d, *J* = 8.0 Hz, 3H), 7.22 (d, *J* = 8.0 Hz, 2H), 6.66 (s, 1H), 6.58 (s, 1H), 1.53 (s, 9H); ^{13}C NMR (100 MHz, $CDCl_3$): δ 162.52, 160.45, 158.56, 152.71, 136.62, 131.71, 124.71, 119.73, 102.29, 80.91, 28.29; LC-MS (ESI): *m/z* 320.9424 $[M+H]^+$.

S7. Synthesis of tert-butyl 4-(6-(4-(methylsulfonyl)phenylamino)pyrimidin-4-ylamino)phenylcarbamate (21)

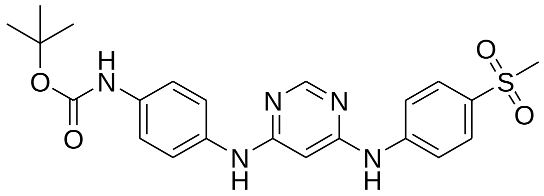

The 4-(methylsulfonyl)benzenamine **8a** (0.802 g, 4.68 mmol) and Cs_2CO_3 (2.53 g, 7.78 mmol) were added to the solution of compound **20** (1 g, 3.12 mmol) in dioxane (4 ml). The reaction mixture was then degassed with argon. After completion of 5 minutes, $Pd(PPh_3)_4$ (0.108 g, 0.093 mmol) was added and the reaction mixture was allowed to stirr at 110 °C for 12 h. After completion of reaction as indicated by TLC, the solvent was removed under reduced pressure. The obtained residue was slowly basified with aqueous $NaHCO_3$ and extracted with ethyl acetate. The organic layer was washed 3 times with aqueous $NaHCO_3$ followed by brine solution. The obtained organic layer was dried over $MgSO_4$, and solvent was evaporated to yield **21** as a yellow solid (0.950 g, 66.90%); m.p. 178-180 °C; ^{1}H NMR (400 MHz, DMSO-d_6): δ 9.61 (s, 1H), 9.21 (s, 1H), 9.08 (s, 1H), 8.28 (s, 1H), 7.83 (d, *J* = 8.0 Hz, 2H), 7.74 (d, *J* = 12.0 Hz, 2H), 7.38-7.32 (m, 4H), 6.12 (s, 1H), 3.10 (s, 3H), 1.43 (s, 9H); ^{13}C NMR (100 MHz, DMSO-d_6): δ 161.42, 160.27, 158.10, 153.27, 146.01, 135.08, 134.62, 132.36, 128.55, 121.92, 119.21, 118.56, 87.72, 79.25, 44.46, 28.58; LC-MS (ESI): *m/z* 455.9876 $[M+H]^+$.

S8. Synthesis of N4-(4-aminophenyl)-N6-(4-(methylsulfonyl)phenyl)pyrimidine-4,6-diamine (22)

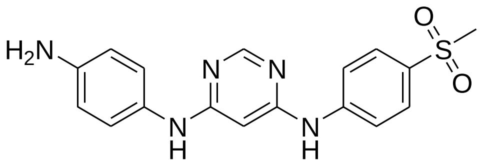

Compound **21** (0.950 g, 1.0 mmol) was treated with 25% TFA/DCM at RT for 2 h, after which time the volatiles were removed in vacuo. The crude was diluted with EtOAc (100 mL), washed with saturated aq $NaHCO_3$ (100 mL x 3), and then with brine solution (100 mL x 3). The organic layer was dried $MgSO_4$ and concentrated in vacuo to provide the product **22** as yellow solid (0.736, 99.72%). m.p. 149-151 °C; ^{1}H NMR (400 MHz, DMSO-d_6): δ 9.51 (s, 1H), 8.66 (s, 1H), 8.20 (s, 1H), 7.81 (d, *J* = 8.0 Hz, 2H), 7.72 (d, *J* = 8.0 Hz, 2H), 6.99 (d, *J* = 8.0 Hz, 2H), 6.54 (d, *J* = 8.0 Hz, 2H), 5.93 (s, 1H), 4.92 (s, 2H), 3.08 (s, 3H); ^{13}C NMR (100 MHz, DMSO-d_6): δ 162.45, 160.25, 158.08, 146.18, 145.99, 132.11, 128.51, 128.21, 124.82, 118.38, 114.65, 86.34, 44.47; LC-MS (ESI): *m/z* 356.1468 $[M+H]^+$.

S9. General procedure for synthesis of 4,6-diaminopyrimidine series of compounds 24a-c: reaction of compound **22** (0.100 g, 0.281 mmol) or its structural analogs with 3-tert-butyl-5-isocyanato-1-methyl-*1H*-pyrazole (**23**) (0.057 g, 0.422 mmol), in presence of triethyl amine (0.098 g, 0.970 mmol), in DCM (2 mL) was stirred at 45 °C for 5 h. The completion of the reaction was monitored by TLC. After completion of the reaction, the organic layer was evaporated. The crude product was purified on silica gel column (mesh 100-200) using DCM: MeOH gradient (100: 0 to 70: 30 ratio of DCM: MeOH). The desired products **24a-c** were isolated in moderate to good yields.

S9.a. 1-(4-(6-(4-(methylsulfonyl)phenylamino)pyrimidin-4-ylamino)phenyl)-3-(3-tert-butyl-1-methyl-1H-pyrazol-5-yl)urea (24a)

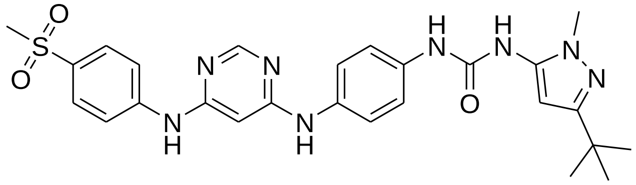

Yellow solid (0.030 g, 20.0%); m.p. 238-240 °C; ^{1}H NMR (400 MHz, DMSO-d$_6$): δ 9.66 (s, 1H), 9.15 (s, 1H), 8.81 (s, 1H), 8.45 (s, 1H), 8.32 (s, 1H), 8.86 (d, J = 8.0 Hz, 2H), 7.78 (d, J = 8.0 Hz, 2H), 7.41 (s, 4H), 6.18 (s, 1H), 6.03 (s, 1H), 3.59 (s, 3H), 3.13 (s, 3H), 1.20 (s, 9H); ^{13}C NMR (100 MHz, DMSO-d$_6$): δ 161.38, 160.27, 158.92, 158.09, 152.29, 146.02, 137.62, 134.74, 132.38, 130.93, 128.55, 121.94, 119.33, 118.58, 93.87, 87.83, 44.47, 35.36, 32.23, 30.81; LC-MS (ESI): *m/z* 535.1774 [M+H]$^+$.

S9.b. 1-(4-(6-(methylamino)pyrimidin-4-ylamino)phenyl)-3-(3-tert-butyl-1-methyl-1H-pyrazol-5-yl)urea (24b)

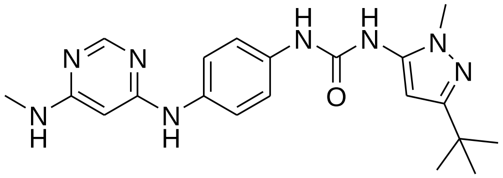

Yellow solid (0.020 g, 18.18%); m.p. 242-244 °C; ^{1}H NMR (400 MHz, DMSO-d$_6$): δ 8.74 (s, 2H), 8.44 (s, 1H), 7.99 (s, 1H), 7.35 (d, J = 8.0 Hz, 2H), 7.30 (d, J = 8.0 Hz, 2H), 6.67 (d, J = 4.0 Hz, 1H), 5.99 (s, 1H), 5.60 (s, 1H), 3.54 (s, 3H), 2.67 (d, J = 4.0 Hz, 3H), 1.16 (s, 9H); ^{13}C NMR (100 MHz, DMSO-d$_6$): δ 163.54, 160.70, 159.02, 157.85, 152.35, 137.64, 135.61, 134.11, 121,17, 119.40, 93.96, 85.39, 35.29, 32.20, 30.77, 27.86; LC-MS (ESI): *m/z* 395.0813[M+H]$^+$.

S9.c. 1-(4-(6-(4-(methylsulfonyl)phenylamino)pyrimidin-4-ylamino)phenyl)-3-(5-tert-butylisoxazol-3-yl)urea (24c)

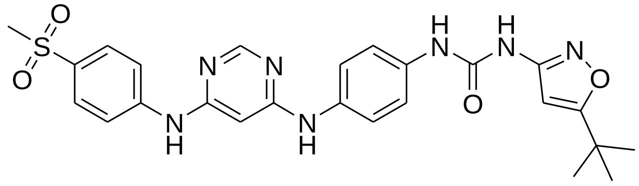

Yellow solid (0.031 g, 21.23%); m.p. 245-247 °C; ^{1}H NMR (400 MHz, DMSO-d$_6$): δ 9.67 (s, 1H), 9.44 (s, 1H), 9.19 (s, 1H), 8.76 (s, 1H), 8.33 (s, 1H), 7.86 (d, J = 8.0 Hz, 2H), 7.78 (d, J = 8.0 Hz, 2H), 7.45 (d, J = 8.0 Hz, 2H), 7.39 (d, J = 12.0 Hz, 2H), 6.48 (s, 1H), 6.18 (s, 1H), 3.13 (s, 3H), 1.28 (s, 9H); ^{13}C NMR (100 MHz, DMSO-d$_6$): δ 180.53, 161.31, 160.26, 158.85, 158.09, 151.79, 145.99, 135.29, 134.21, 132.41, 128.56, 121.76, 119.72, 118.60, 92.83, 87.98, 44.46, 32.89, 28.78; LC-MS (ESI): *m/z* 522.1923 [M+H]$^+$.

S10. Biochemical Inhibition Assay:[1,2] Kinase activity was measured in a microfluidics assay that monitors the separation of a phosphorylated product from substrate. The assay was run using a 12-sipper chip on a Caliper EZ Reader II (PerkinElmer®, Walthman, USA) with separation buffer (100 mM HEPES, 10 mM EDTA, 0.015% Brij-35, 0.1% CR-3 [PerkinElmer®, Walthman, USA]). In 96-well polypropylene plates (Greiner, Frickenhausen, Germany) compound stocks (20 mM in DMSO) were diluted into kinase buffer (50 mM HEPES, 0.075% Brij-35, 0.1% Tween 20, 2 mM DTT, 10 mM $MgCl_2$, and 0.02% NaN_3) in 12-point ½log dilutions (2 mM–6.32 nM). After, 1 µL was transferred into a 384-well polypropylene assay plate (Greiner, Frickenhausen, Germany). The FLT3 enzyme (Invitrogen™, Grand Island, USA) was diluted in kinase buffer to a concentration of 2 nM and 5 µL of the enzyme mixture was transferred to the assay plate. The inhibitors/FLT3 enzyme were incubated for 60 minutes with minor shaking. A substrate mix was prepared containing ATP (Ambresco®, Solon, USA) and 5FAM tagged FLT3 peptide (peptide #22, 5' FAM-EPLYWSFPA, PerkinElmer®, Walthman, USA) dissolved in kinase buffer, and 5 µL of the substrate mix was added to the assay plate. Running concentrations were as follows: ATP (190 µM), peptide (1.5 µM), compound 12-point ½log dilutions (0.2 mM–0.632 nM). For positive control, no inhibitor was added. For negative control, no enzyme was added. For running control, quizartinib was utilized. The plate was run until 10-20% conversion based on the positive control wells. The following separation conditions were utilized: upstream voltage -500V; downstream voltage, -1900V; chip pressure -0.8. Percent inhibition was measured for each well comparing starting peptide to phosphorylated product peaks relative to the baseline. Dose response curves, spanning the IC_{50} dose, were generated in GraphPad Prisim 6 and fit to an exponential one-phase decay line and IC_{50} values were obtained from the half-life value of the curve. IC_{50} values were generated in duplicate and error was calculated from the standard deviation between values.

S11. Mechanism of Inhibition Procedure: Compound **13a** was pre-incubated with the FLT3 kinase at 1, 3, 5, 10, 20, 30, 50, and 90 minutes. After the pre-incubation, IC_{50} values at each incubation interval was determined according to the procedure outlined in section 4.3. In a separate experiment, compound **13a** was pre-incubated with the FLT3 kinase for 60 minutes. After, IC_{50} values were determined at 5, 10, 20, 40, 80, and 160 µM ATP. To determine IC_{50} values at each concentration of ATP, same procedure was followed as outlined in section S10.

***S12. Computational Modeling**:*[1-3] Computational modeling studies were completed using AutoDock Vina,[3] AutoDock Tools, and Discovery Studio 3.5. Using AutoDock Tools, kinase crystal structures were prepared as follows: 1) All hydrogens were added as 'Polar Only' 2) A grid box for the ATP binding site was created. Compounds to be computationally modeled were assigned appropriate rotatable bonds using AutoDock Tools. To computational model the compounds, AutoDock Vina was employed. AutoDock Vina provides docking scores in terms of ΔG values. After the modeling study, kinase inhibitors docked in FLT3 were visualized and analyzed with Discovery Studio 3.5.

***S13. Cell Cultures**:* Stable BaF3 populations expressing activated FLT3 were generated by retroviral spinfection with the appropriate mutated plasmid followed by selection and growth factor withdrawal as previously described.[4] The BaF3 cell line was originally obtained from the laboratory of Charles Sawyers and has not been authenticated. MV411 and Molm14 cells were obtained from the laboratory of Scott Kogan and authenticated by Promega STR analysis in June 2013. All cell lines were mycoplasma-free. Cells were incubated with compounds for 48 hours and proliferation was assessed using CellTiter-Glo (Promega; Madison, WI) according to the manufacturer's recommendation on a SpectraMax M3 microplate reader using SpectraMax Software (Molecular Devices; Sunnyvale, CA). All cell viability data shown is reflective of experiments performed a minimum of three times.

B. <u>NMR SPECTRA SCANS</u>

S14. ^{1}H, ^{13}C spectrum of ethyl 2-(4-((6-chloropyrimidin-4-yl)amino)phenyl)acetate (7)

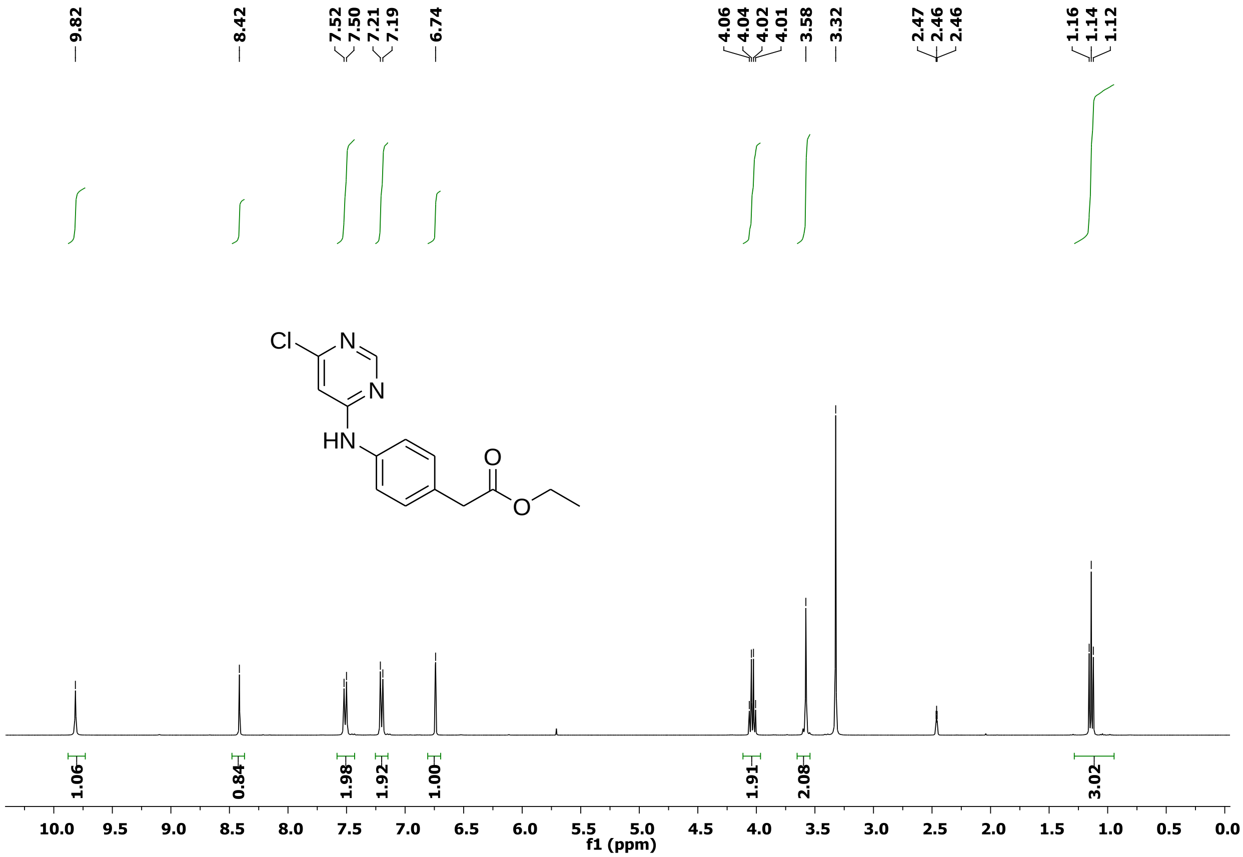

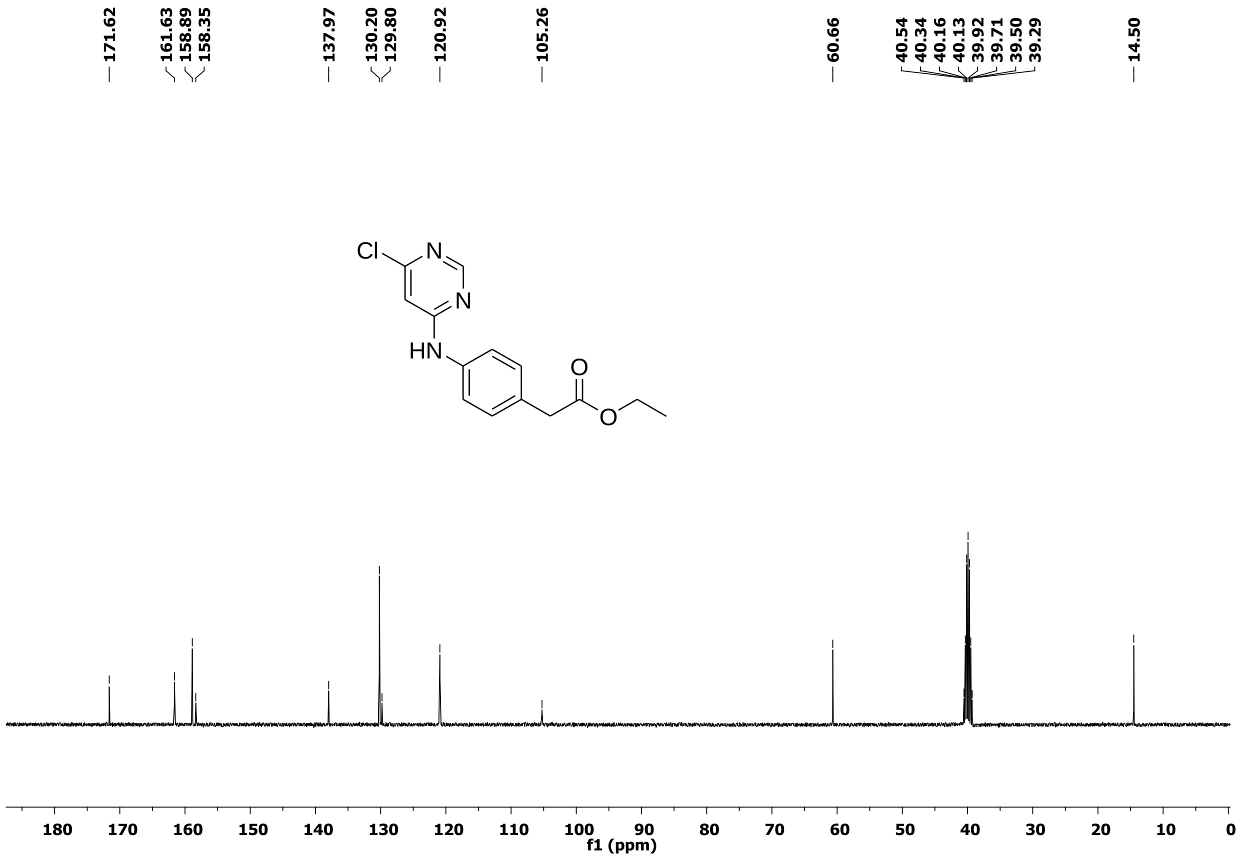
171.62
161.63
158.89
158.35
137.97
130.20
129.80
120.92
105.26
60.66
40.54
40.34
40.16
40.13
39.92
39.71
39.50
39.29
14.50
Cl
N
N
HN
O
O
180 170 160 150 140 130 120 110 100 90 80 70 60 50 40 30 20 10 0
f1 (ppm)

S15. ^{1}H, ^{13}C spectrum of ethyl 2-(4-(6-(4-methylsulfonyl)phenylamino)pyrimidin-4-ylamino)phenyl)acetate (10)

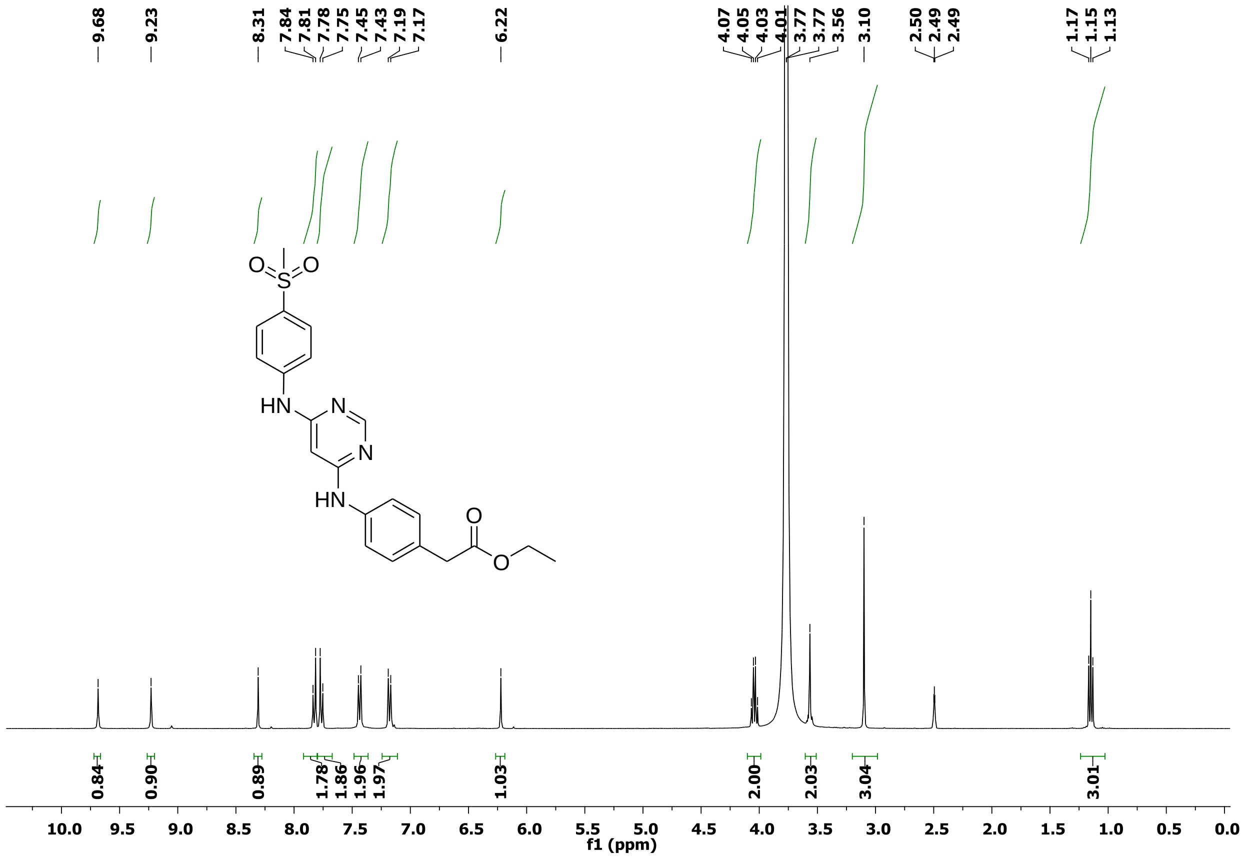

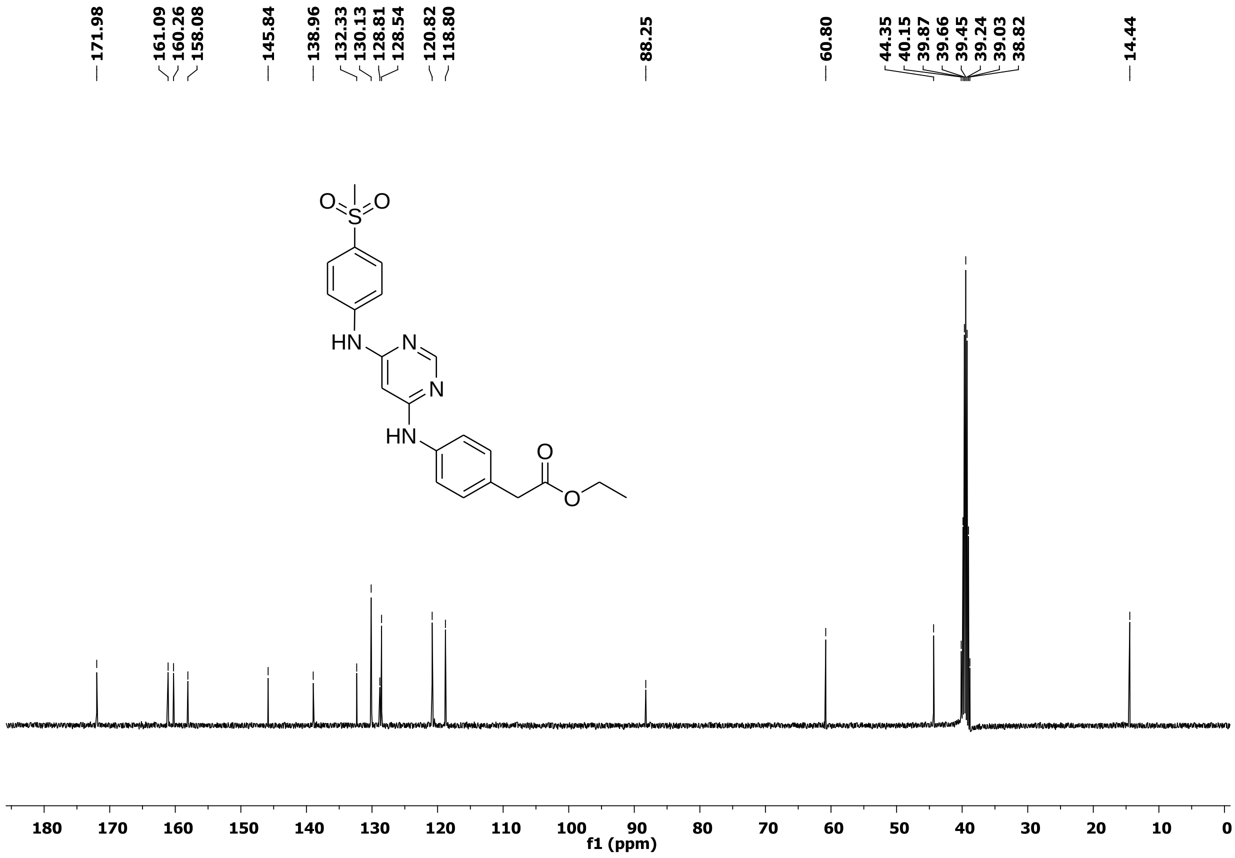

171.98
161.09
160.26
158.08
145.84
138.96
132.33
130.13
128.81
128.54
120.82
118.80
88.25
60.80
44.35
40.15
39.87
39.66
39.45
39.24
39.03
38.82
14.44
O
S
O
HN
N
N
HN
O
O
180
170
160
150
140
130
120
110
100
90
80
70
60
50
40
30
20
10
0
f1 (ppm)

S16. ^{1}H, ^{13}C spectrum of 2-(4-(6-(4-(methylsulfonyl)phenylamino)pyrimidin-4-ylamino)phenyl)acetic acid (11)

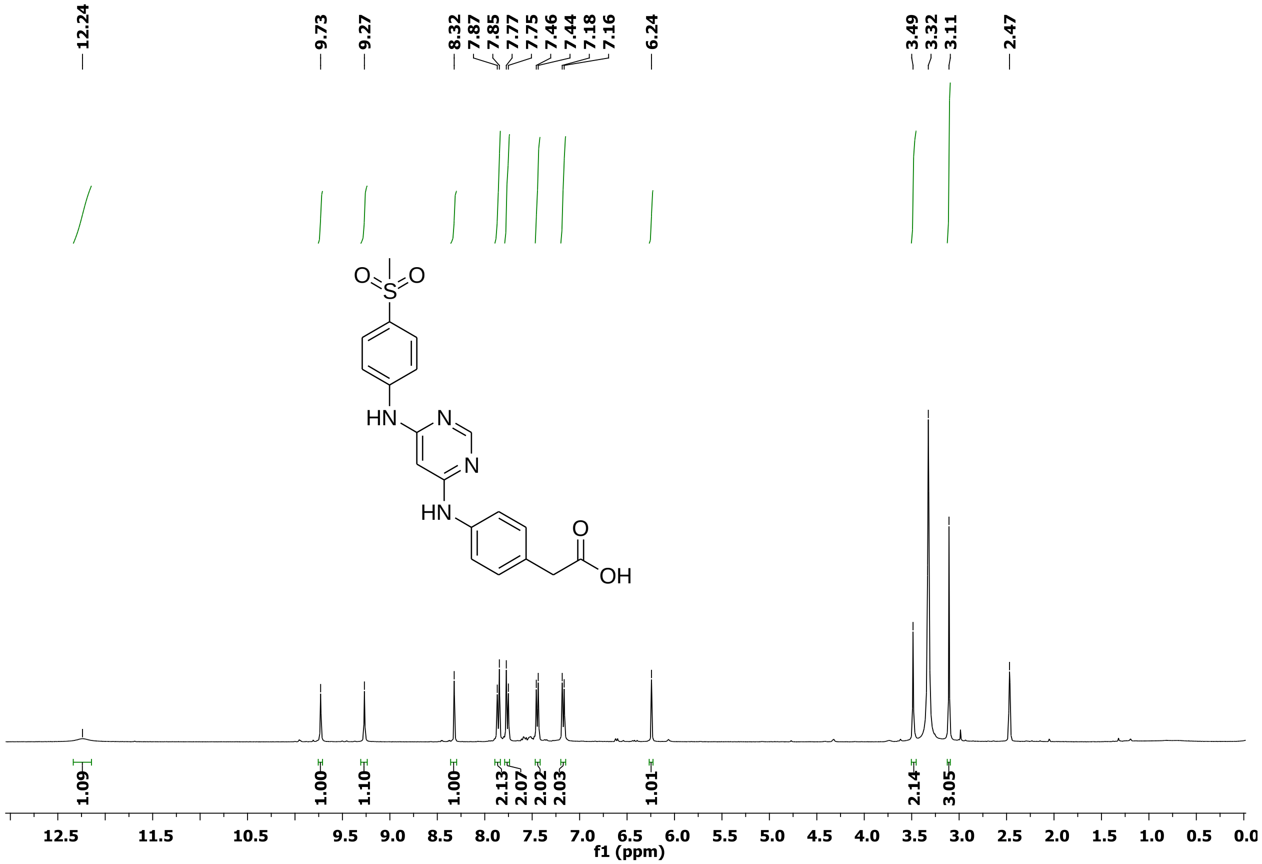

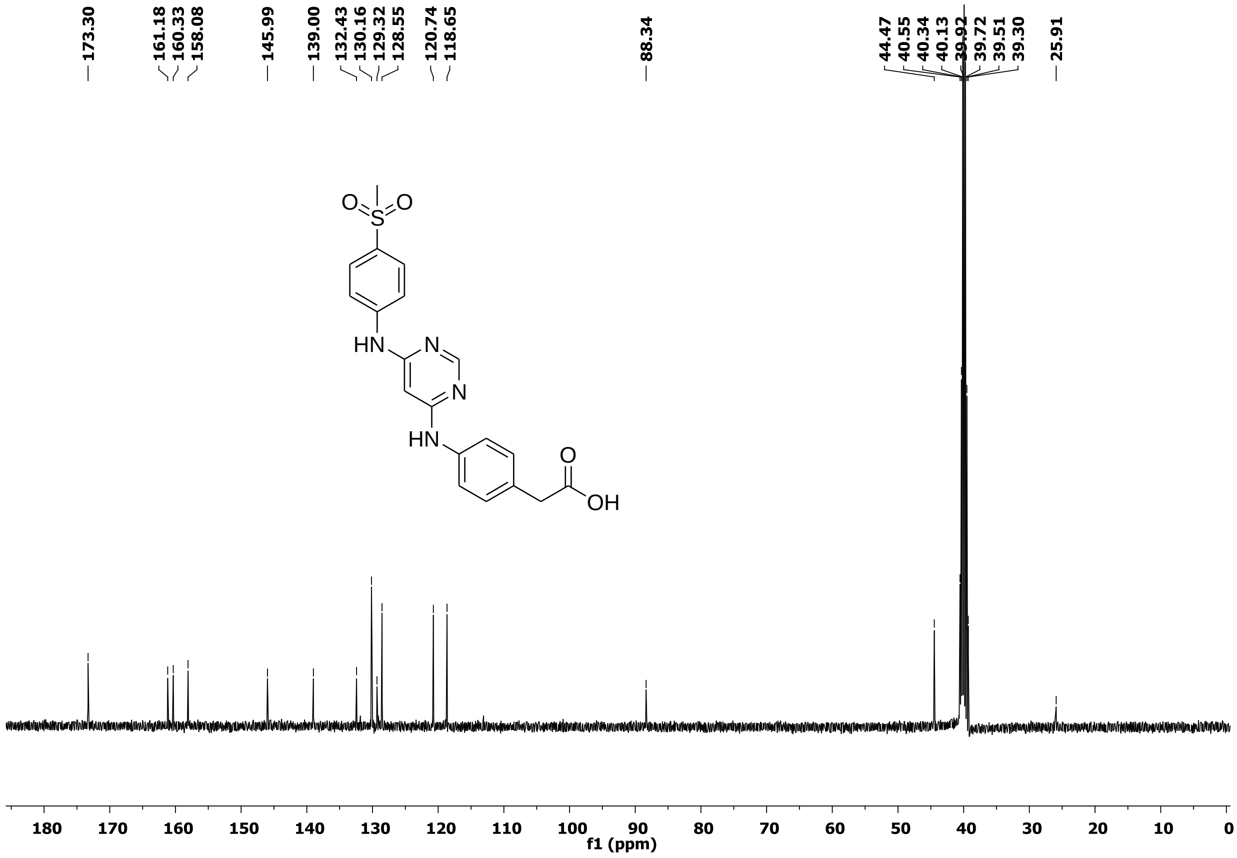
173.30
161.18
160.33
158.08
145.99
139.00
132.43
130.16
129.32
128.55
120.74
118.65
88.34
44.47
40.55
40.34
40.13
39.92
39.72
39.51
39.30
25.91
HN
N
N
HN
O
S
O
O
OH
180
170
160
150
140
130
120
110
100
90
80
70
60
50
40
30
20
10
0
f1 (ppm)

S17. ^{1}H, ^{13}C spectrum of 2-(4-(6-(4-(methylsulfonyl)phenylamino)pyrimidin-4-ylamino)phenyl)-N-(3-tert-butyl-1-methyl-1H-pyrazol-5-yl)acetamide (13a)

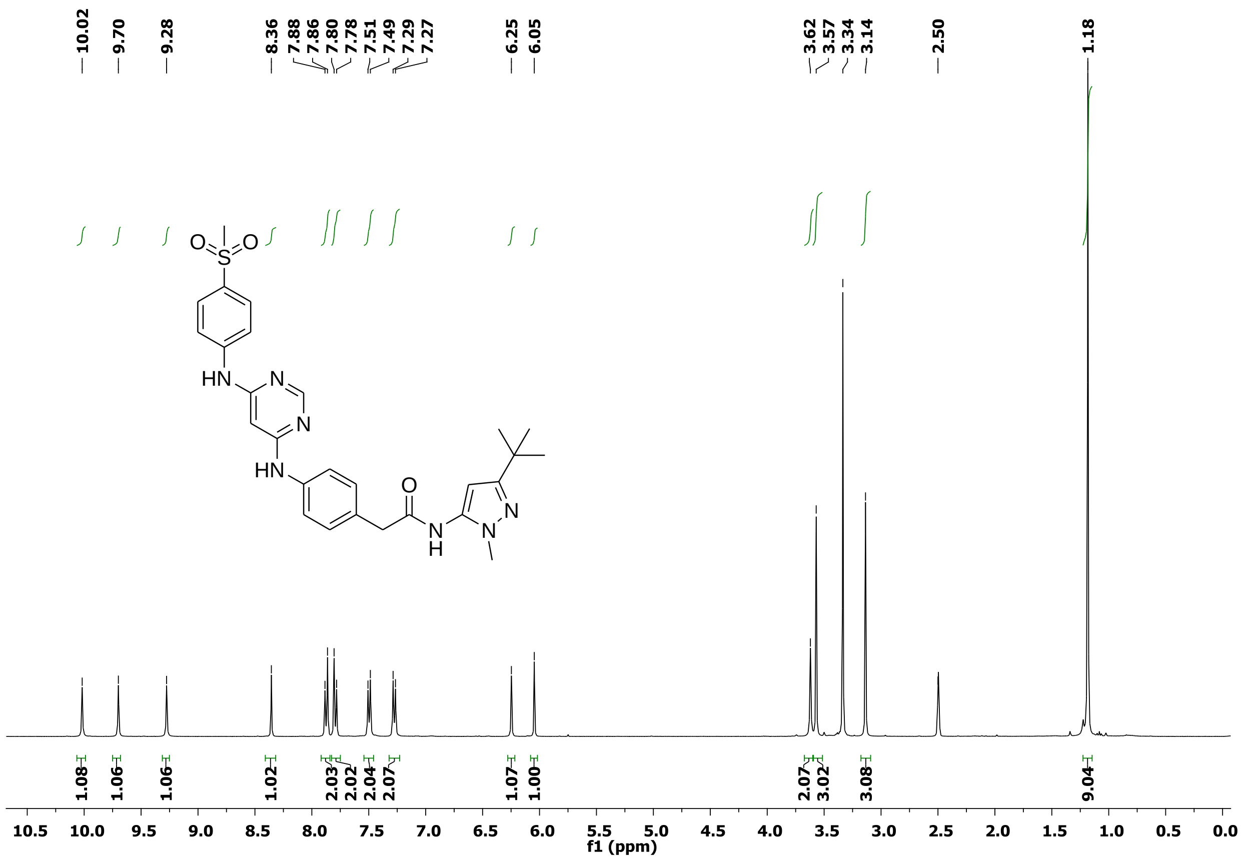

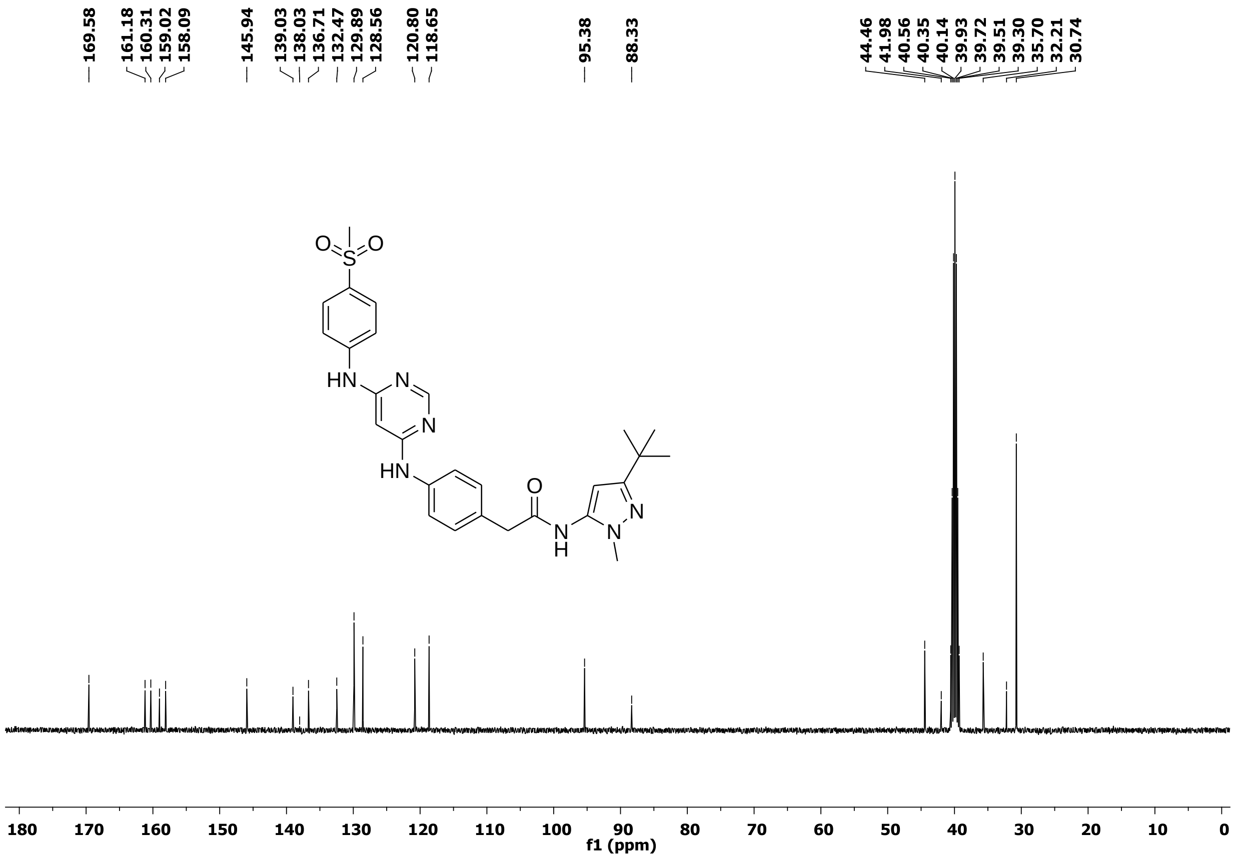
169.58
161.18
160.31
159.02
158.09
145.94
139.03
138.03
136.71
132.47
129.89
128.56
120.80
118.65
95.38
88.33
44.46
41.98
40.56
40.35
40.14
39.93
39.72
39.51
39.30
35.70
32.21
30.74
O
S
O
HN
N
N
HN
O
N
H
N
N
180
170
160
150
140
130
120
110
100
90
80
70
60
50
40
30
20
10
0
f1 (ppm)

S18. ^{1}H, ^{13}C spectrum of 2-(4-(6-(methylamino)pyrimidin-4-ylamino)phenyl)-N-(3-tert-butyl-1-methyl-1H-pyrazol-5-yl)acetamide (13b)

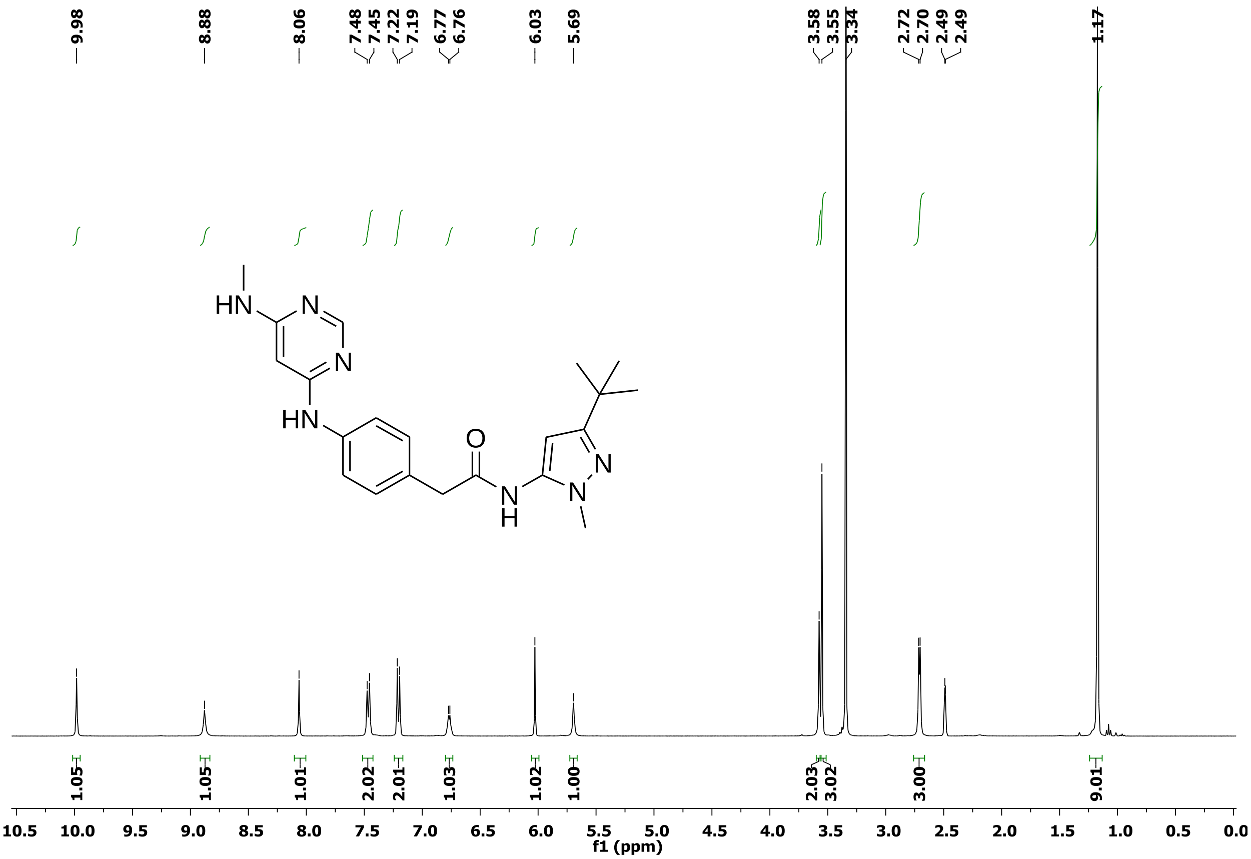

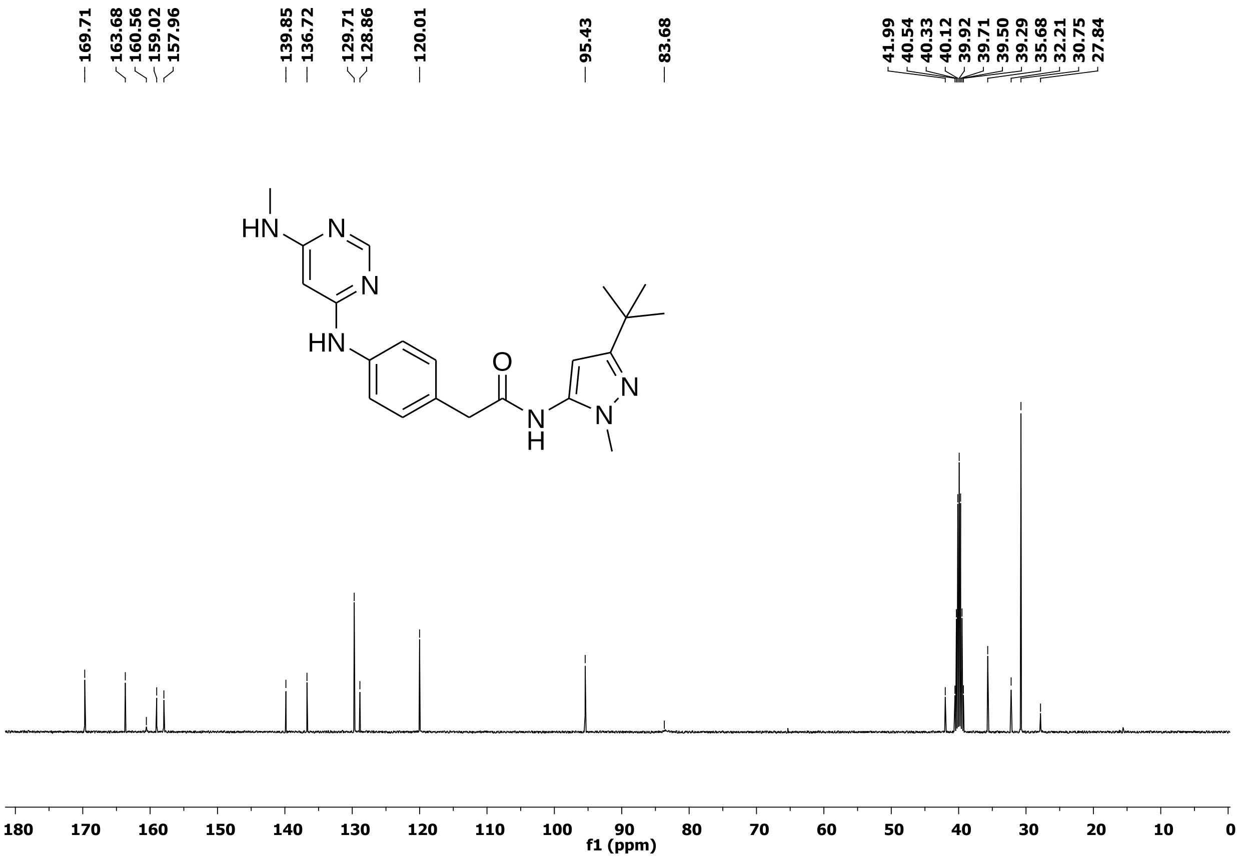
169.71
163.68
160.56
159.02
157.96
139.85
136.72
129.71
128.86
120.01
95.43
83.68
41.99
40.54
40.33
40.12
39.92
39.71
39.50
39.29
35.68
32.21
30.75
27.84
HN
N
N
HN
O
N
H
N
N
180 170 160 150 140 130 120 110 100 90 80 70 60 50 40 30 20 10 0
f1 (ppm)

S19. ^{1}**H,** ^{13}**C spectrum of 2-(4-(6-(3-(piperidin-1-yl)propylamino)pyrimidin-4-ylamino)phenyl)-N-(3-tert-butyl-1-methyl-1H-pyrazol-5-yl)acetamide (13c)**

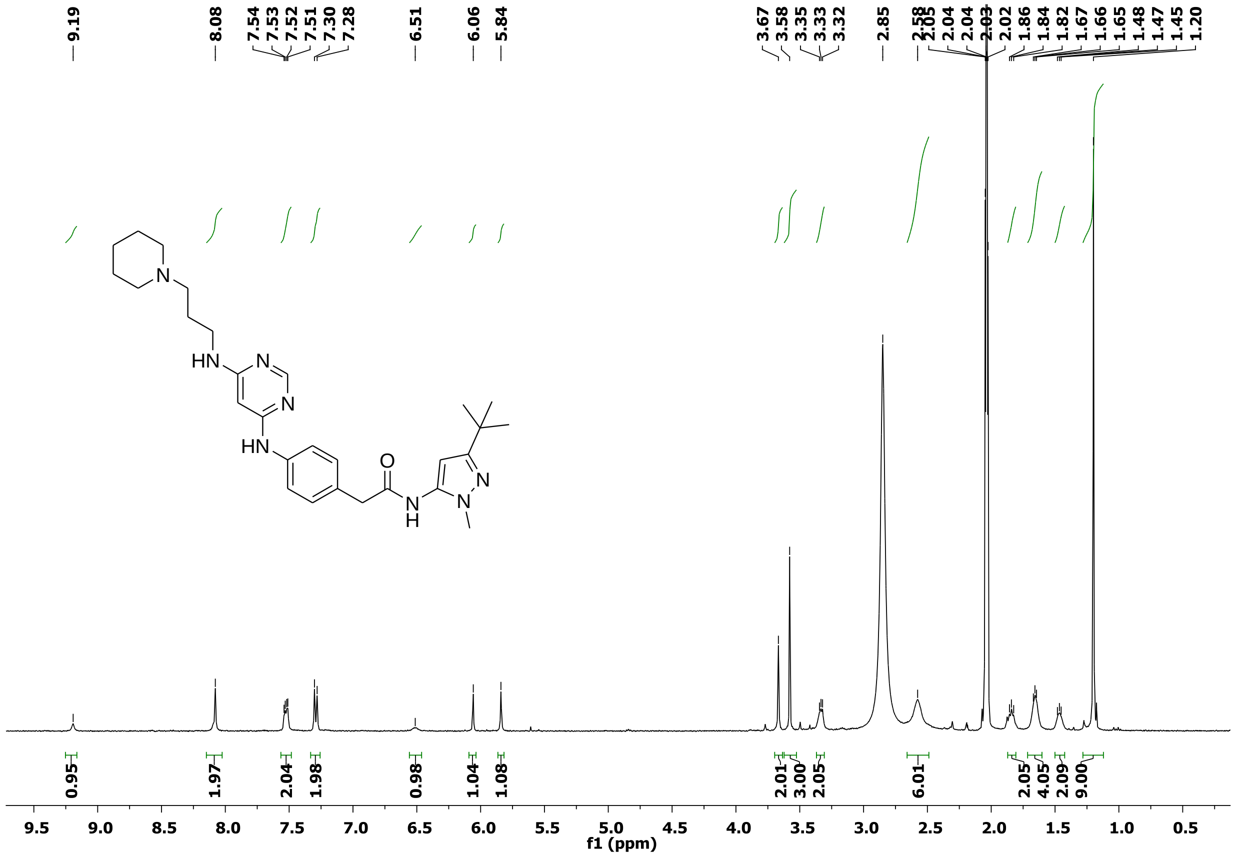

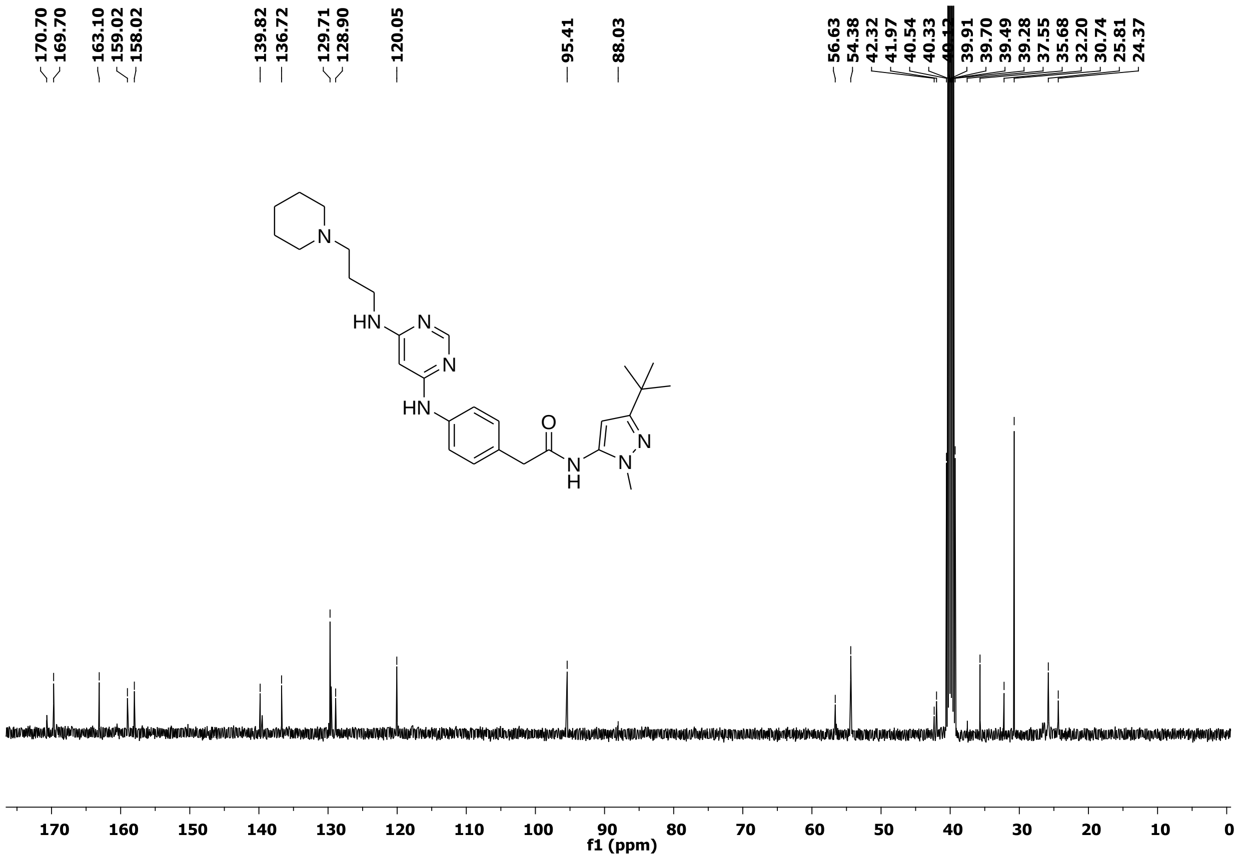
170.70
169.70
163.10
159.02
158.02
139.82
136.72
129.71
128.90
120.05
95.41
88.03
56.63
54.38
42.32
41.97
40.54
40.33
39.91
39.70
39.49
39.28
37.55
35.68
32.20
30.74
25.81
24.37
N
HN
N
N
HN
O
N
N
N
H
170
160
150
140
130
120
110
100
90
80
70
60
50
40
30
20
10
0
f1 (ppm)

S20. ^{1}H, ^{13}C **spectrum of 2-(4-(6-(3-(pyrrolidin-1-yl)propylamino)pyrimidin-4-ylamino)phenyl)-N-(3-tert-butyl-1-methyl-1H-pyrazol-5-yl)acetamide (13d)**

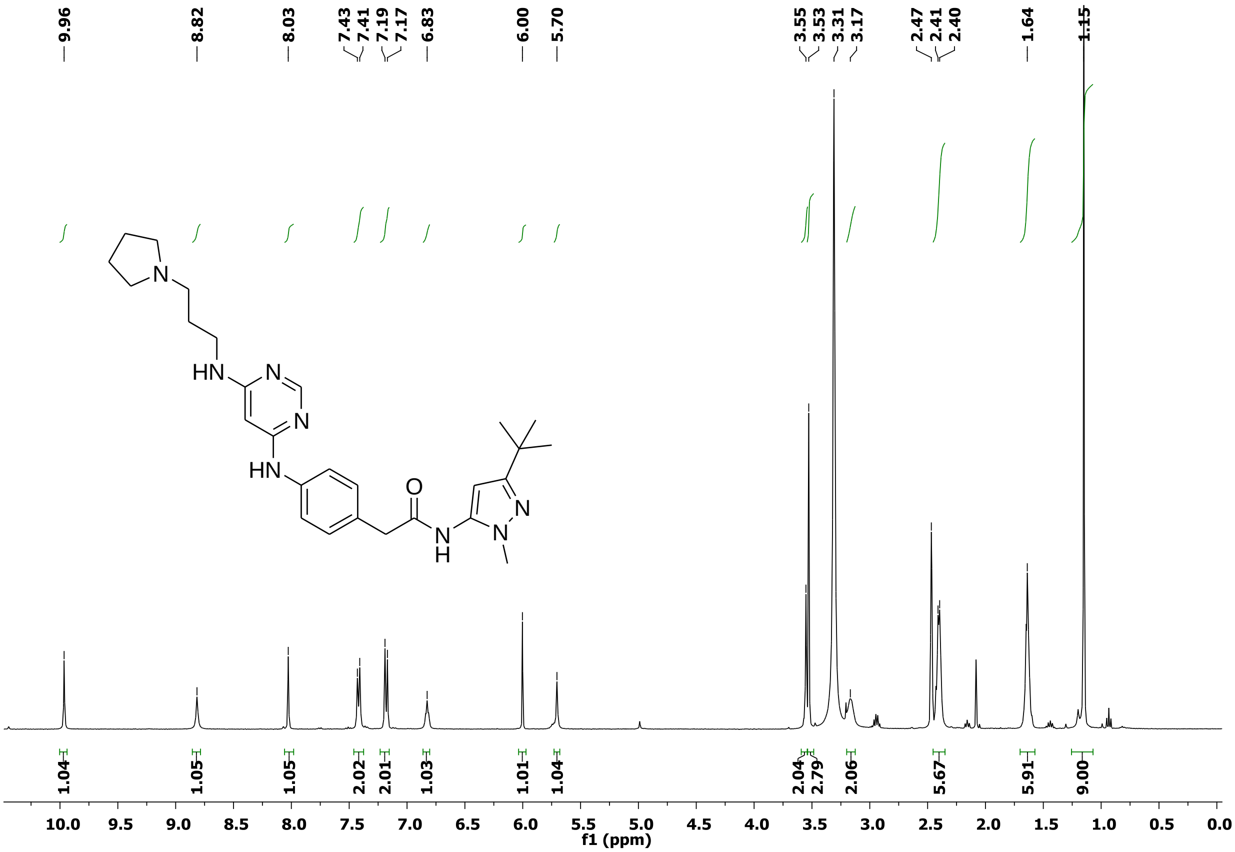

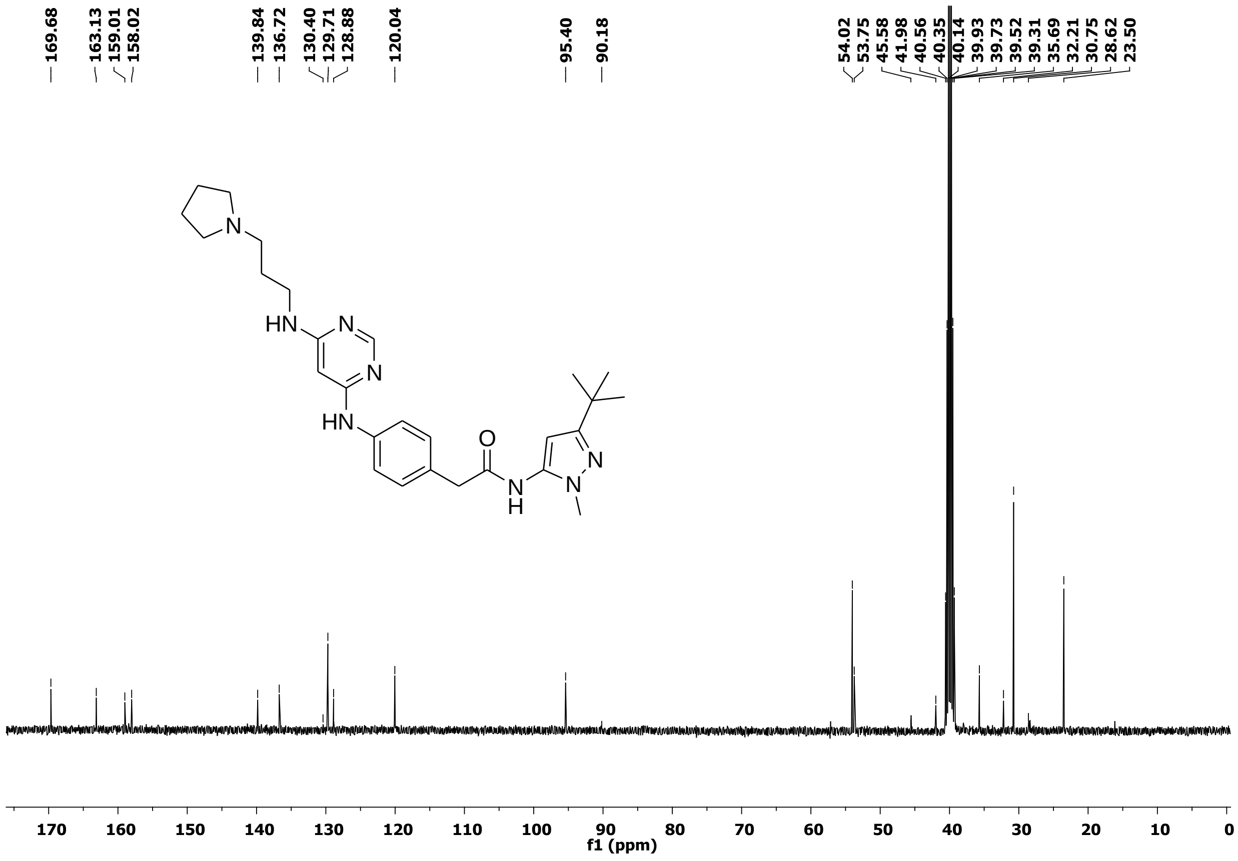
169.68
163.13
159.01
158.02
139.84
136.72
130.40
129.71
128.88
120.04
95.40
90.18
54.02
53.75
45.58
41.98
40.56
40.35
40.14
39.93
39.73
39.52
39.31
35.69
32.21
30.75
28.62
23.50
f1 (ppm)

S21. ^{1}H, ^{13}C spectrum of 2-(4-(6-(4-(methylsulfonyl)phenylamino)pyrimidin-4-yloxy)phenyl)-N-(3-tert-butyl-1-methyl-1H-pyrazol-5-yl)acetamide (13e)

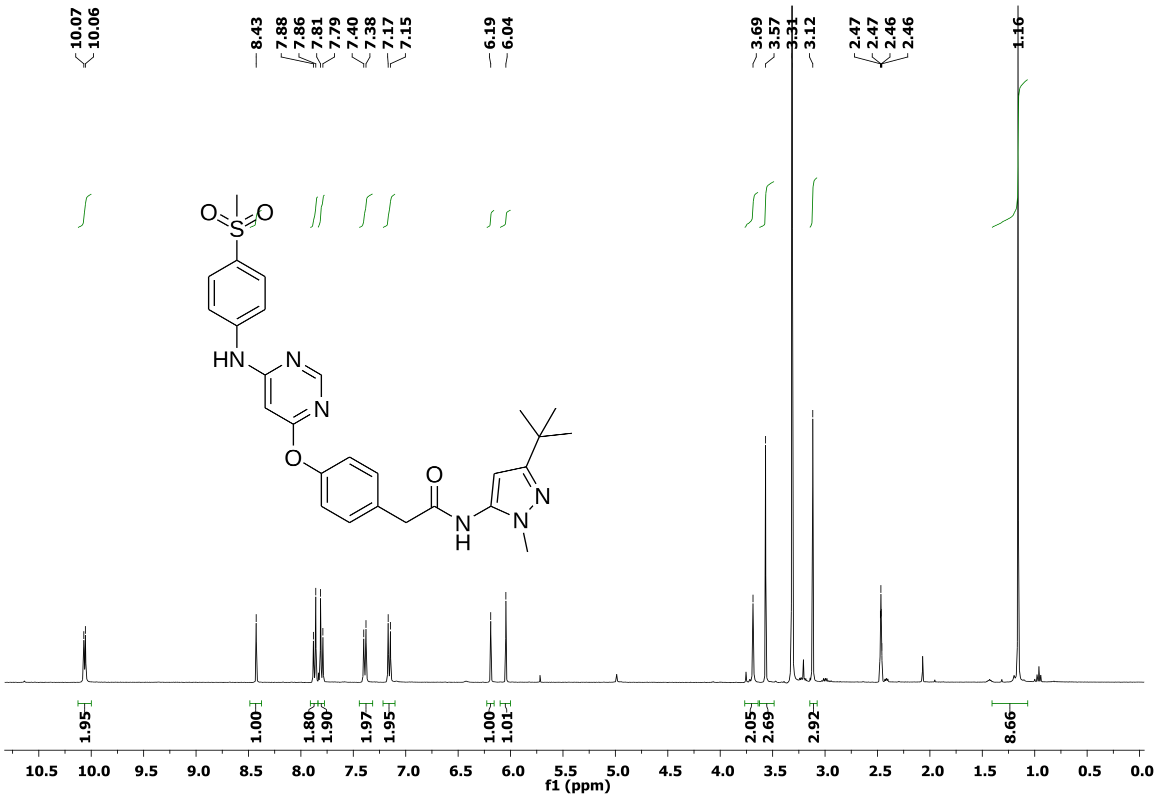

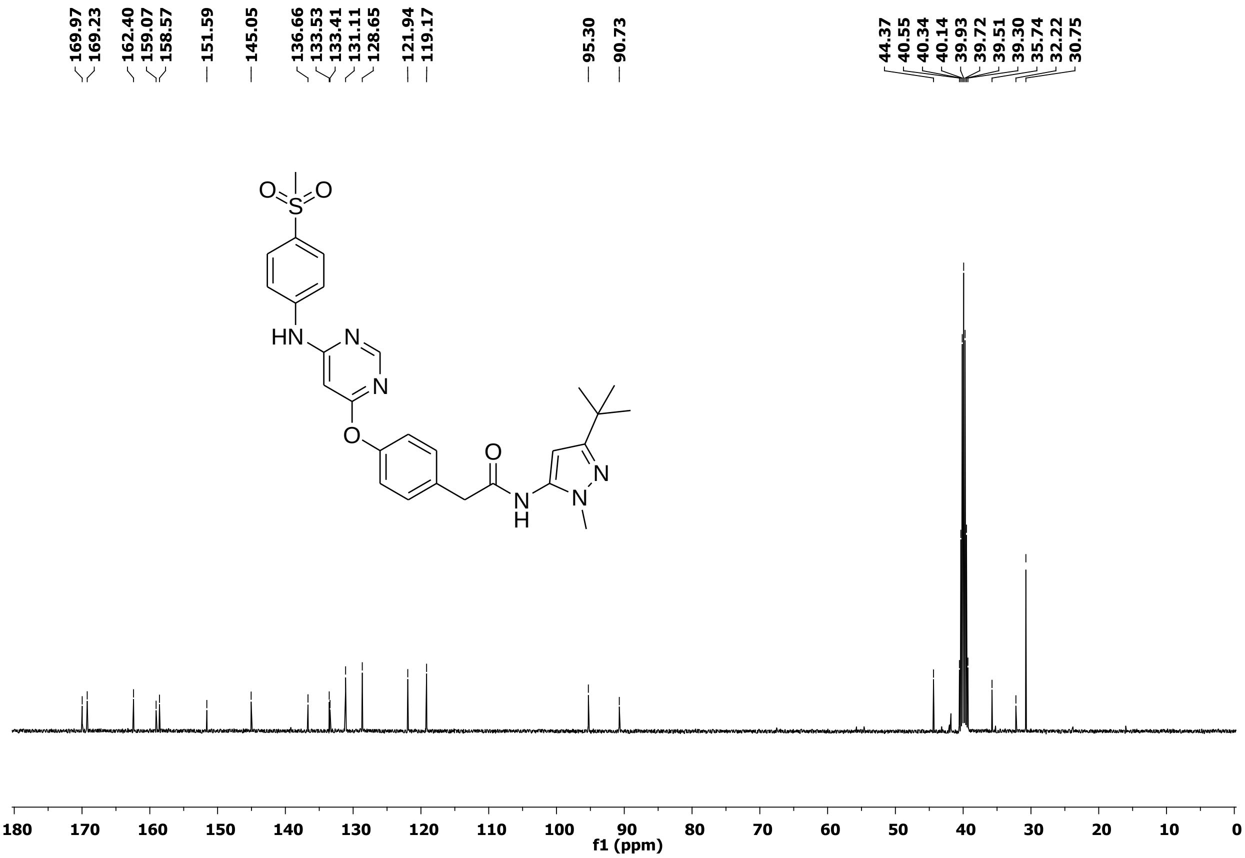
169.97
169.23
162.40
159.07
158.57
151.59
145.05
136.66
133.53
133.41
131.11
128.65
121.94
119.17
95.30
90.73
44.37
40.55
40.34
40.14
39.93
39.72
39.51
39.30
35.74
32.22
30.75
O
S
O
HN
N
N
O
O
N
H
N
N
180
170
160
150
140
130
120
110
100
90
80
70
60
50
40
30
20
10
0
f1 (ppm)

S22. ^{1}H, ^{13}C spectrum of 2-(4-(6-(3-(trifluoromethyl)phenylamino)pyrimidin-4-yloxy)phenyl)-N-(3-tert-butyl-1-methyl-1H-pyrazol-5-yl)acetamide (13f)

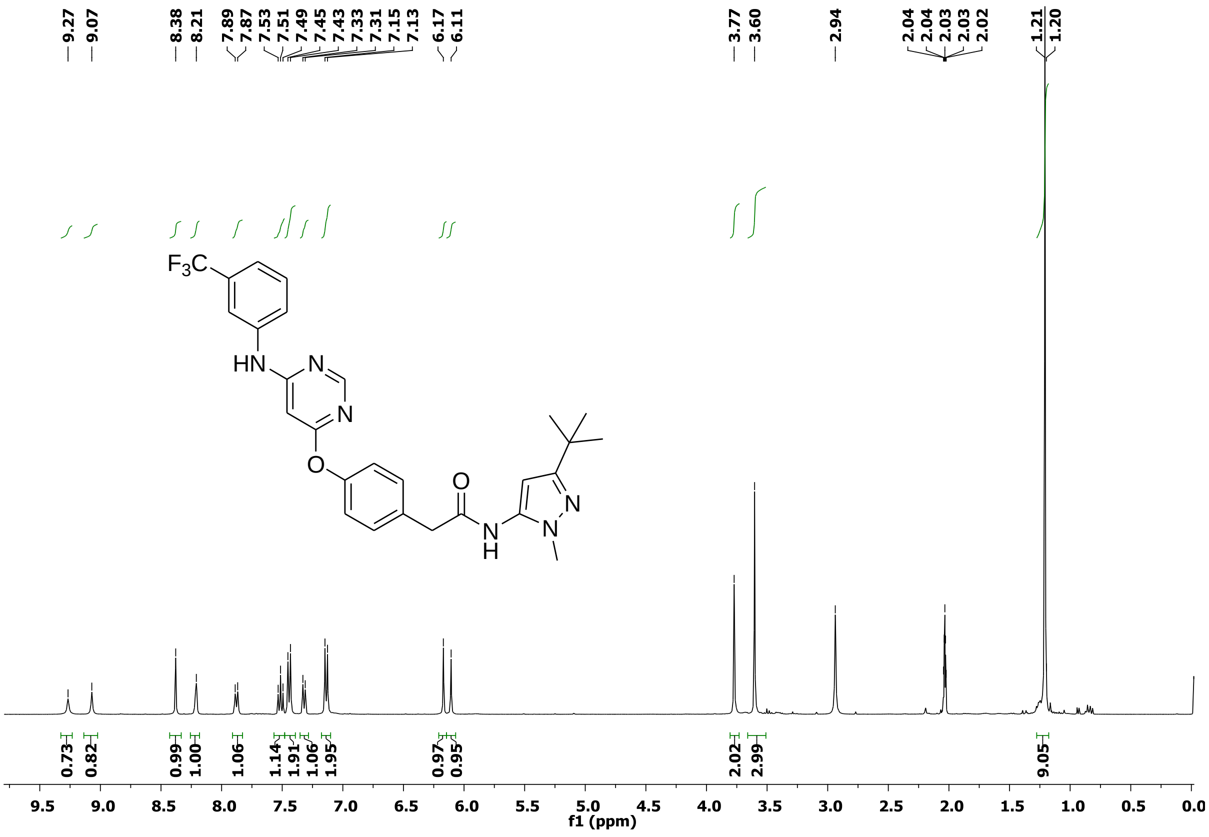

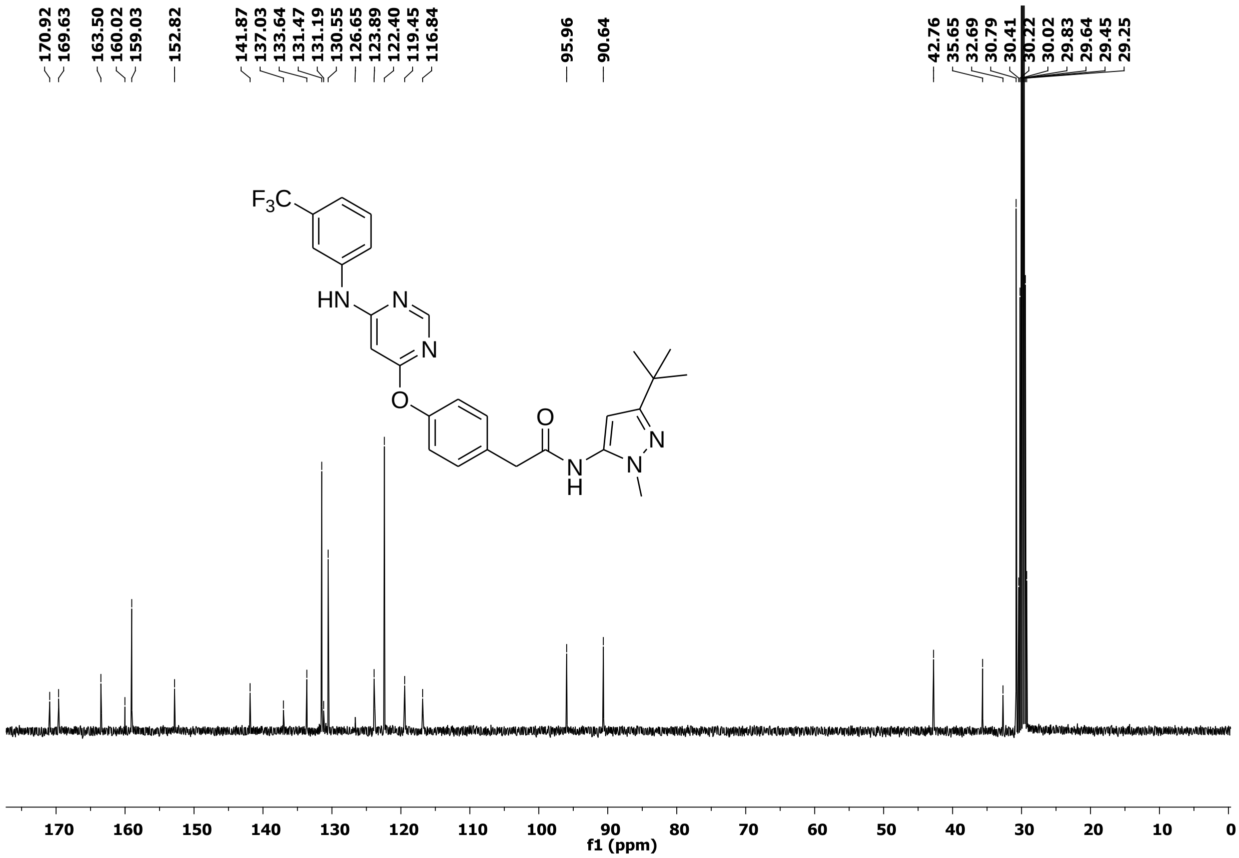
170.92
169.63
163.50
160.02
159.03
152.82
141.87
137.03
133.64
131.47
131.19
130.55
126.65
123.89
122.40
119.45
116.84
95.96
90.64
42.76
35.65
32.69
30.79
30.41
30.22
30.02
29.83
29.64
29.45
29.25
F3C
HN
N
N
O
O
N
H
N
N
170 160 150 140 130 120 110 100 90 80 70 60 50 40 30 20 10 0
f1 (ppm)

S23. ^{1}H, ^{13}C spectrum of 2-(4-(6-(4-(methylsulfonyl)phenylamino)pyrimidin-4-ylamino)phenyl)-N-(3-tert-butyl-1-(4-fluorophenyl)-1H-pyrazol-5-yl)acetamide (13g)

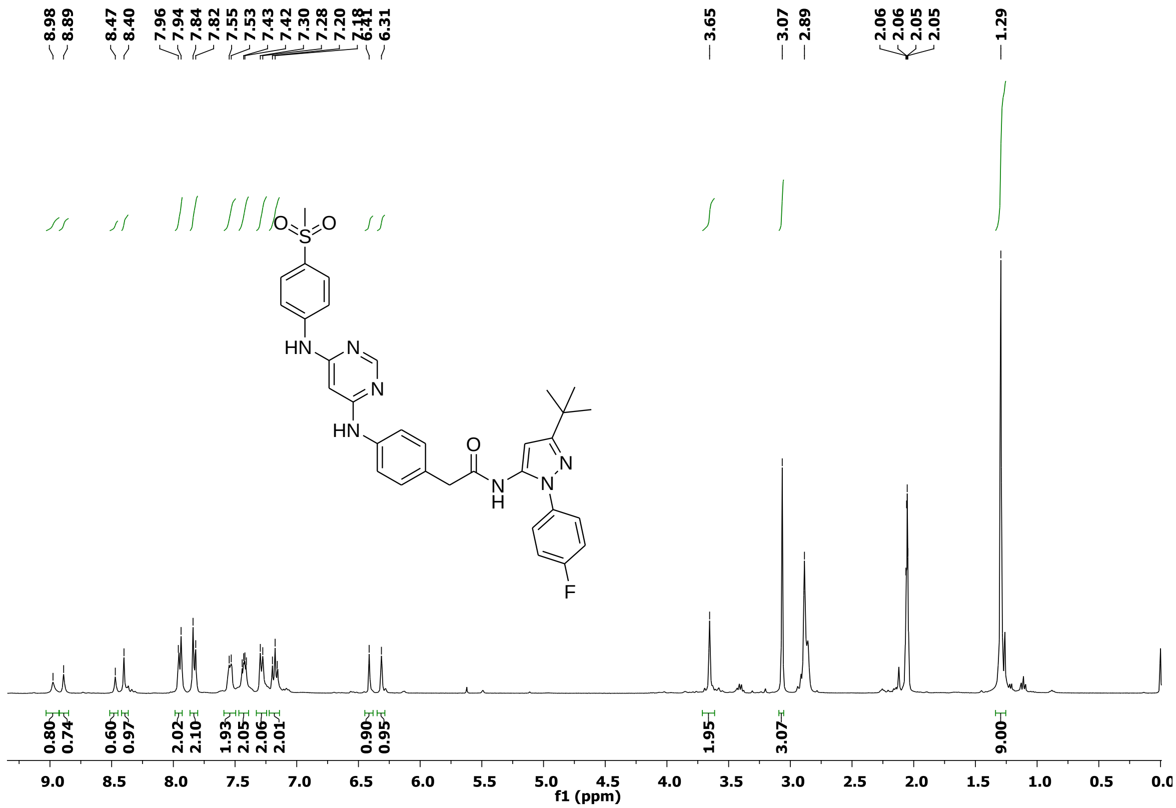

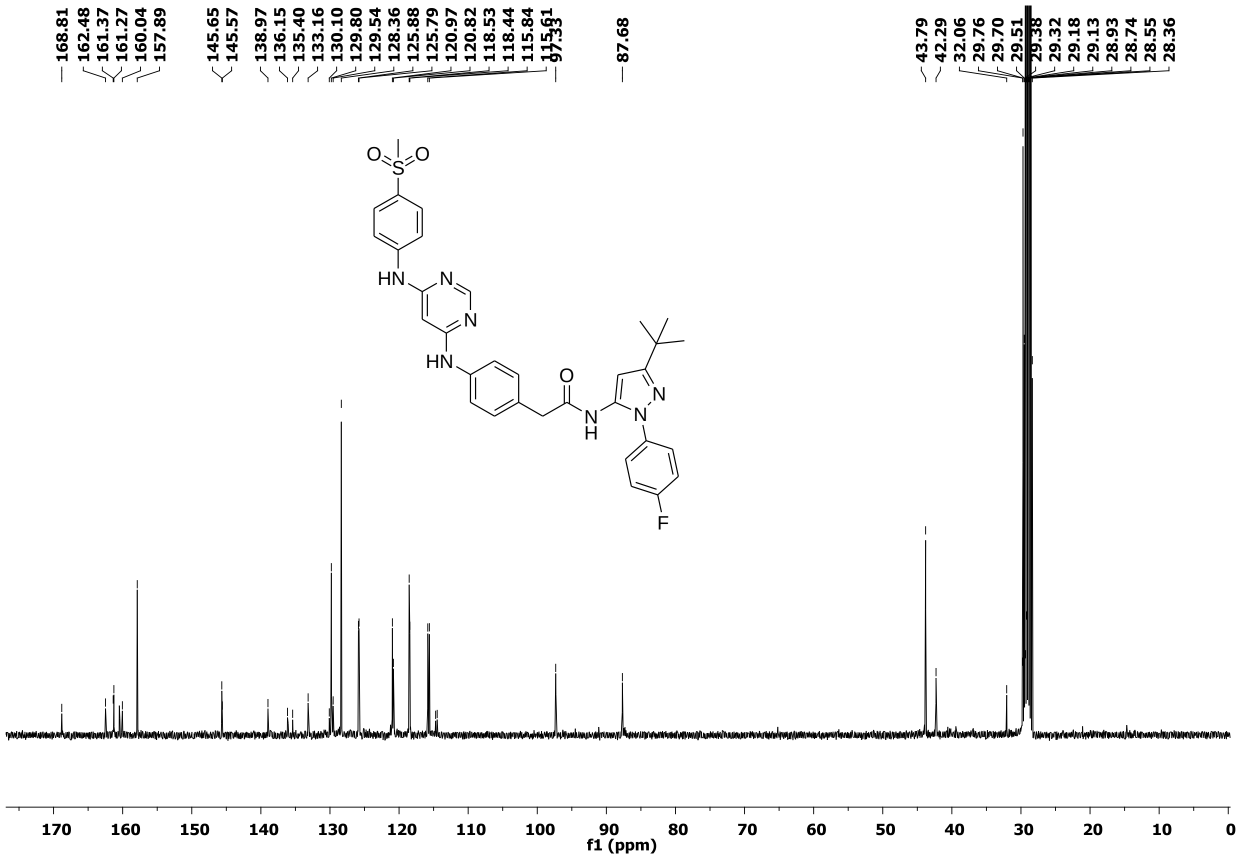
168.81
162.48
161.37
161.27
160.04
157.89
145.65
145.57
138.97
136.15
135.40
133.16
130.10
129.80
129.54
128.36
125.88
125.79
120.97
120.82
118.53
118.44
115.84
115.61
97.33
87.68
43.79
42.29
32.06
29.76
29.70
29.51
29.38
29.32
29.18
29.13
28.93
28.74
28.55
28.36
HN
N
N
HN
O
N
H
N
N
F
O
S
O
170
160
150
140
130
120
110
100
90
80
70
60
50
40
30
20
10
0
f1 (ppm)

S24. ^{1}H, ^{13}C spectrum of 2-(4-(6-(4-(methylsulfonyl)phenylamino)pyrimidin-4-ylamino)phenyl)-N-(3-tert-butyl-1-p-tolyl-1H-pyrazol-5-yl)acetamide (13h)

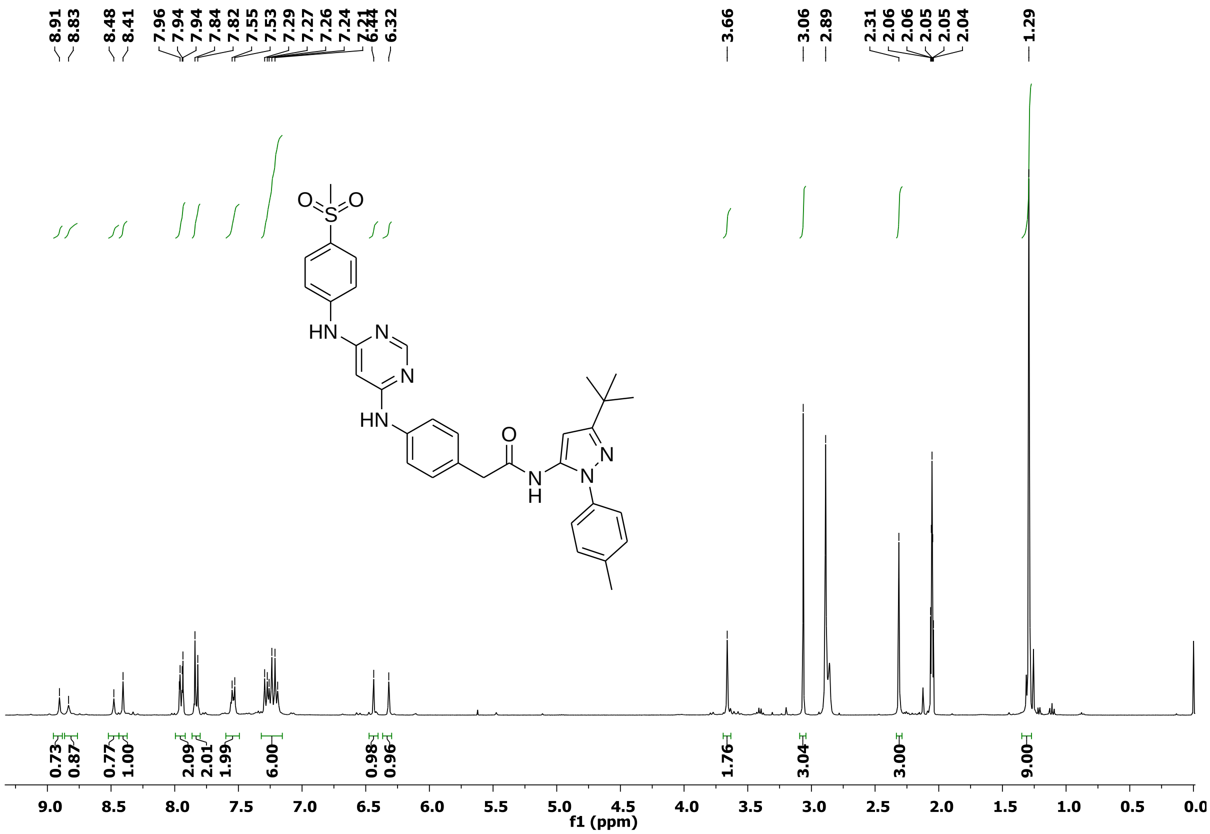

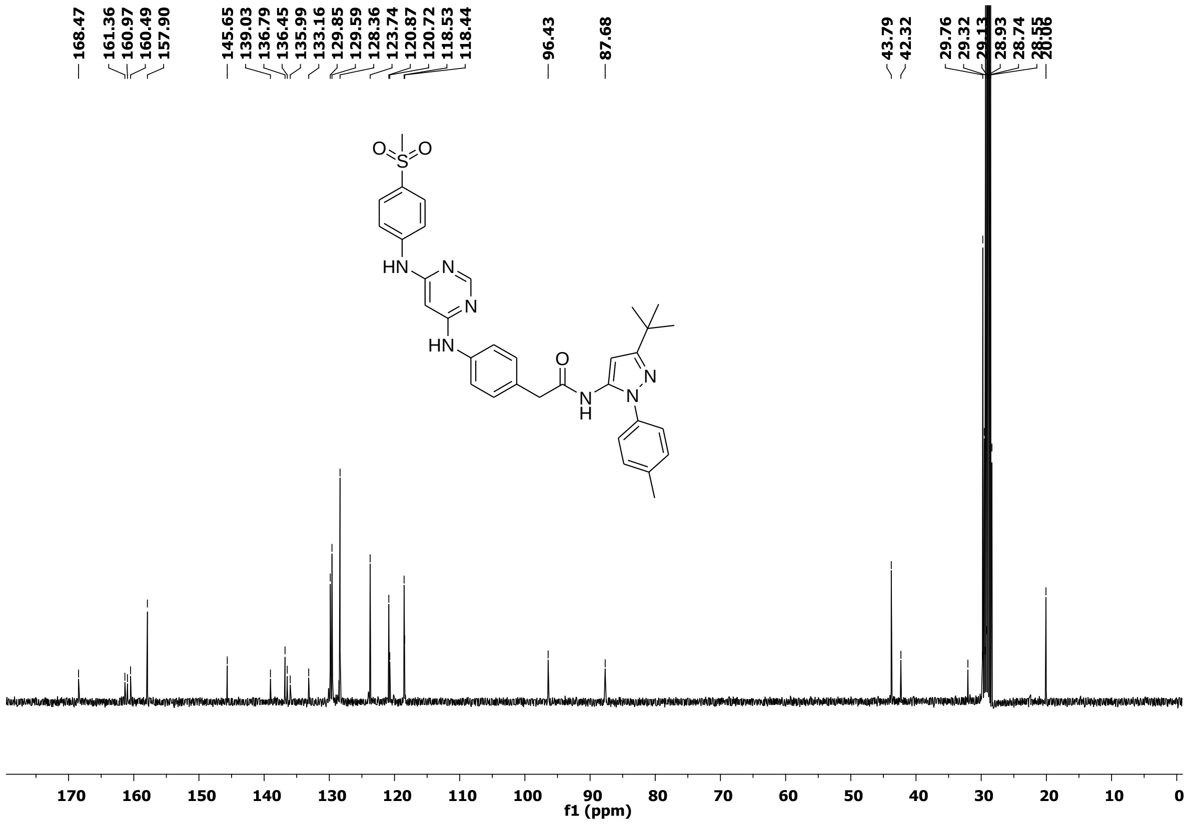
168.47
161.36
160.97
160.49
157.90
145.65
139.03
136.79
136.45
135.99
133.16
129.85
129.59
128.36
123.74
120.87
120.72
118.53
118.44
96.43
87.68
43.79
42.32
29.76
29.32
29.13
28.93
28.74
28.55
O
S
O
HN
N
N
HN
O
N
H
N
N
170
160
150
140
130
120
110
100
90
80
70
60
50
40
30
20
10
0
f1 (ppm)

S25. **^1H, ^{13}C spectrum of 2-(4-(6-morpholinopyrimidin-4-ylamino)phenyl)-N-(3-tert-butyl-1-methyl-1H-pyrazol-5-yl)acetamide (13i)**

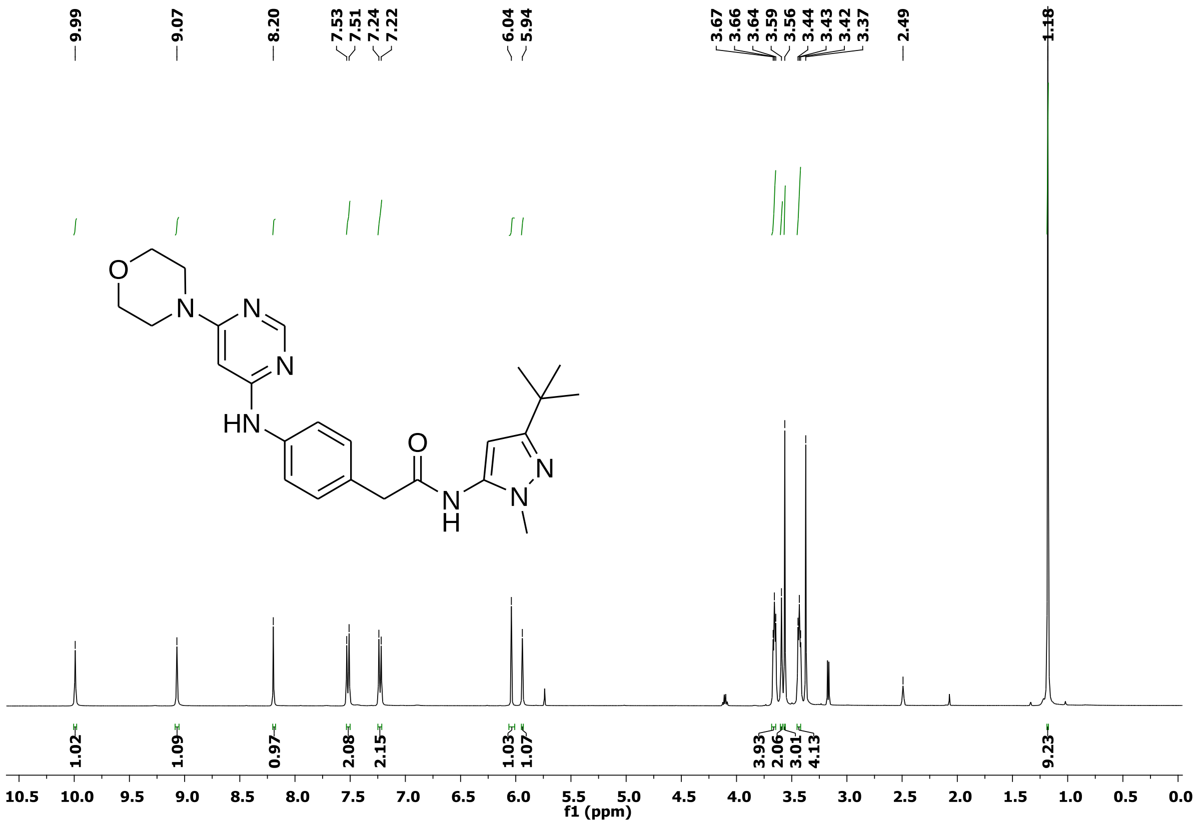

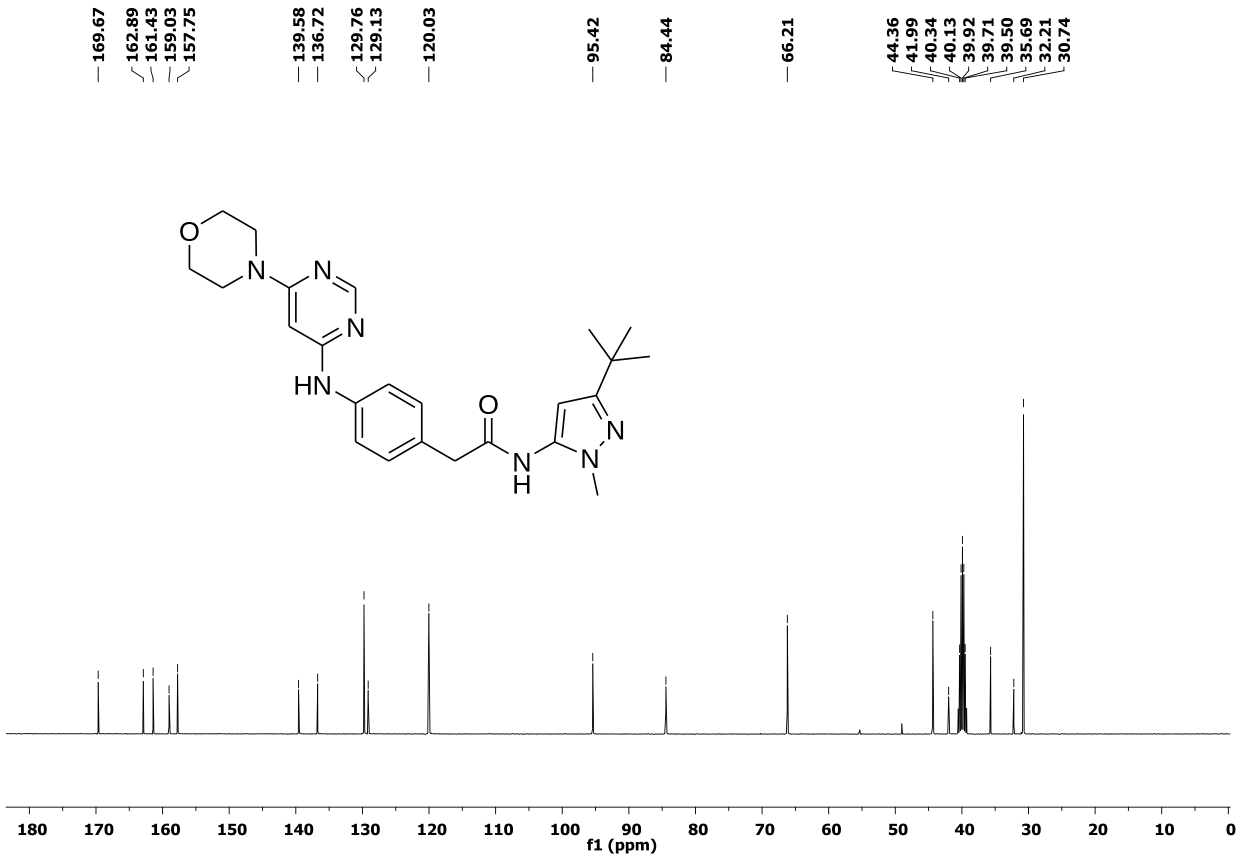
169.67
162.89
161.43
159.03
157.75
139.58
136.72
129.76
129.13
120.03
95.42
84.44
66.21
44.36
41.99
40.34
40.13
39.92
39.71
39.50
35.69
32.21
30.74
180
170
160
150
140
130
120
110
100
90
80
70
60
50
40
30
20
10
0
f1 (ppm)

S26. ^{1}H, ^{13}C spectrum of 2-(4-(6-(4-methylpiperazin-1-yl)pyrimidin-4-ylamino)phenyl)-N-(3-tert-butyl-1-(4-bromophenyl)-1H-pyrazol-5-yl)acetamide (13j)

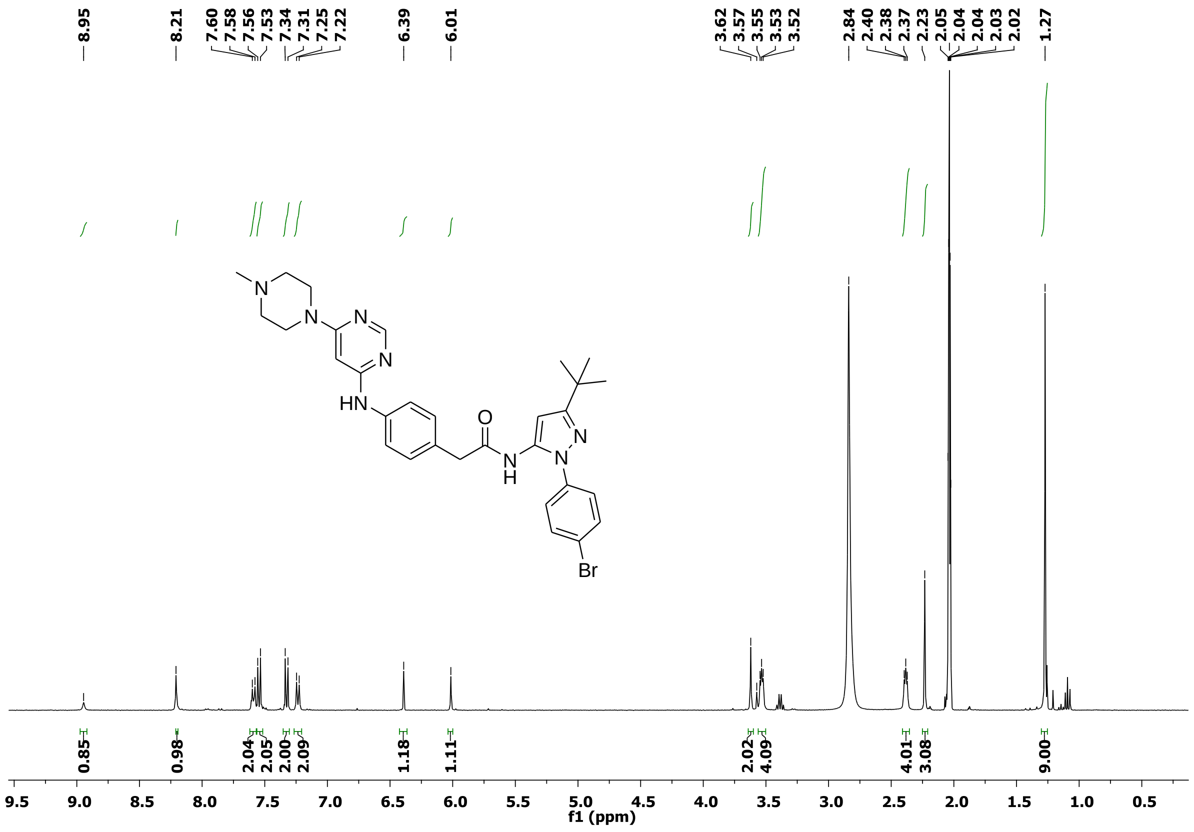

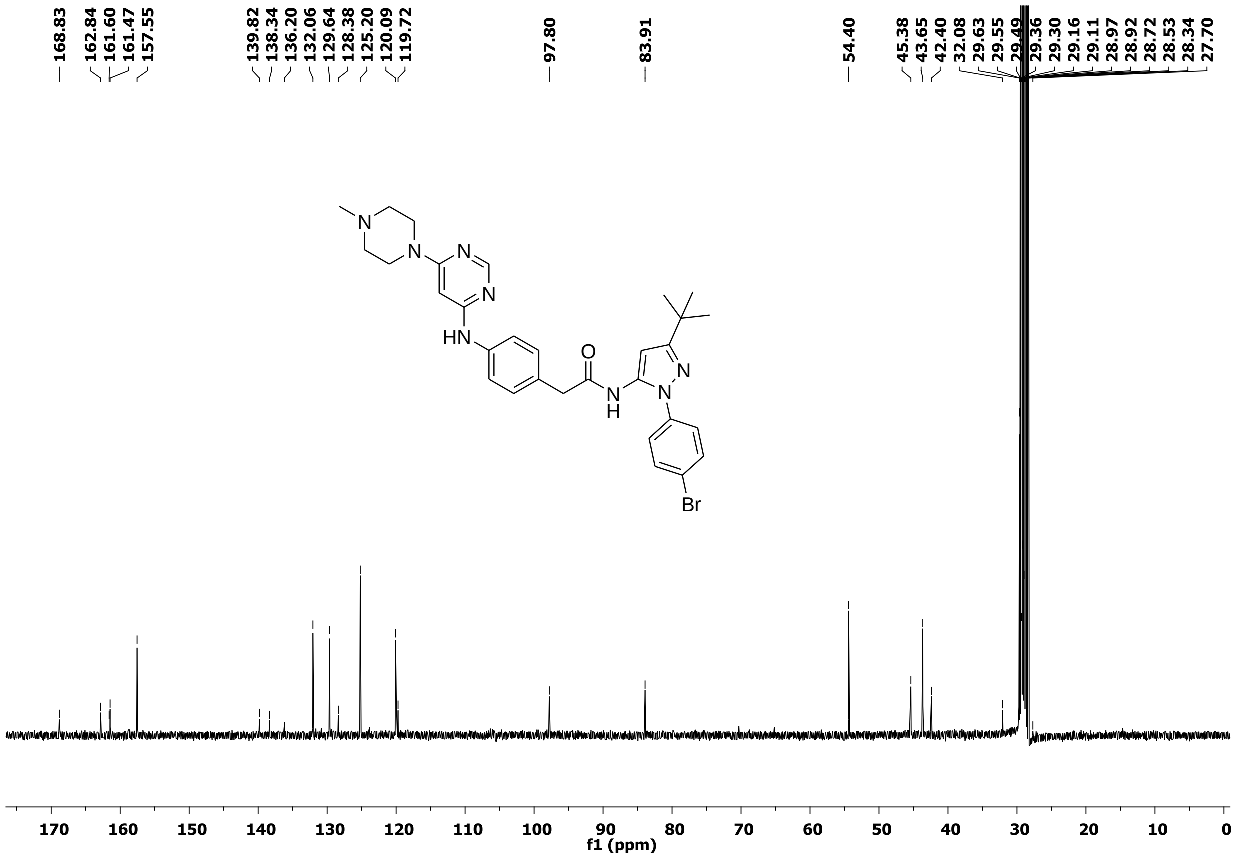
168.83
162.84
161.60
161.47
157.55
139.82
138.34
136.20
132.06
129.64
128.38
125.20
120.09
119.72
97.80
83.91
54.40
45.38
43.65
42.40
32.08
29.63
29.55
29.49
29.36
29.30
29.16
29.11
28.97
28.92
28.72
28.53
28.34
27.70
HN
N
N
N
N
O
N
H
N
N
Br
170 160 150 140 130 120 110 100 90 80 70 60 50 40 30 20 10 0
f1 (ppm)

S27. ^{1}H, ^{13}C spectrum of 2-(4-(6-(4-(methylsulfonyl)phenylamino)pyrimidin-4-ylamino)phenyl)-N-(5-tert-butylisoxazol-3-yl)acetamide (13k)

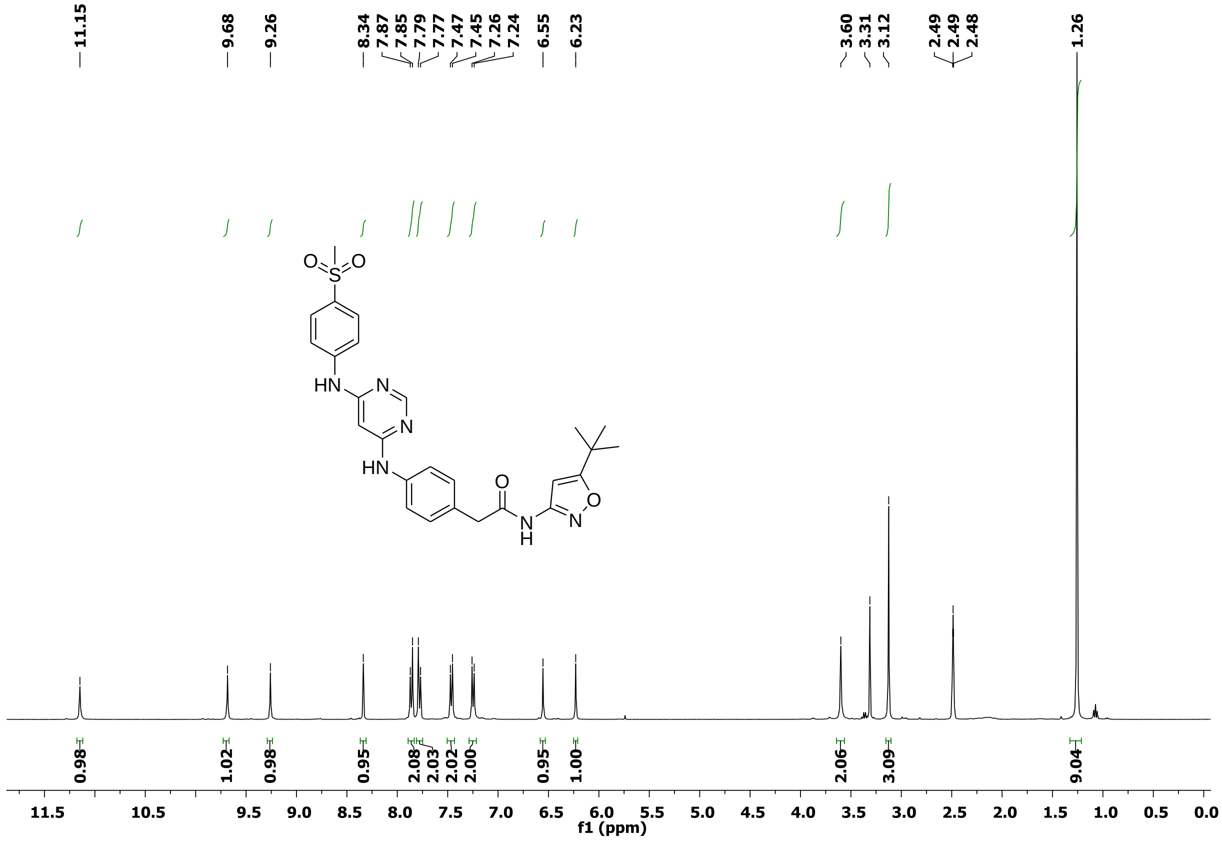

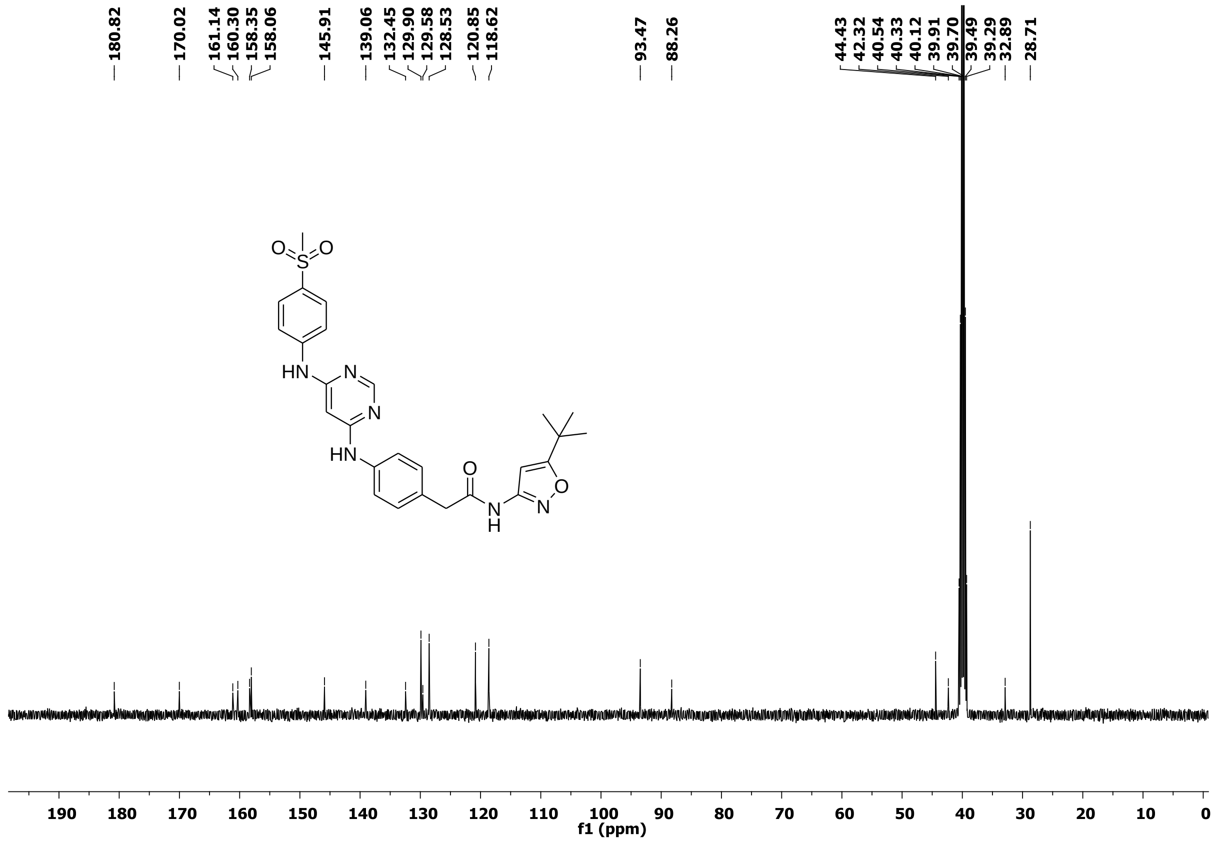
180.82
170.02
161.14
160.30
158.35
158.06
145.91
139.06
132.45
129.90
129.58
128.53
120.85
118.62
93.47
88.26
44.43
42.32
40.54
40.33
40.12
39.91
39.70
39.49
39.29
32.89
28.71
f1 (ppm)

S28. ^{1}H, ^{13}C spectrum of 2-(4-(6-(3-(trifluoromethyl)phenylamino)pyrimidin-4-yloxy)phenyl)-N-(5-tert-butylisoxazol-3-yl)acetamide (13l)

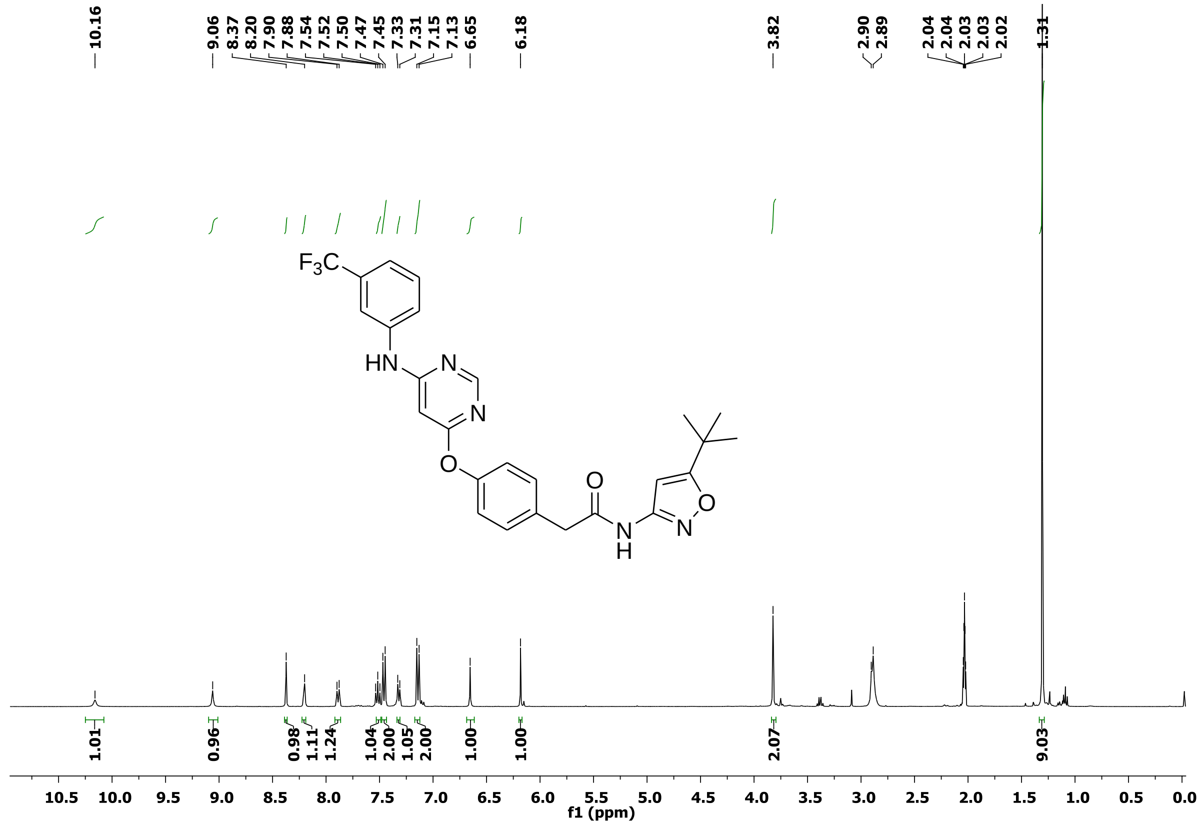

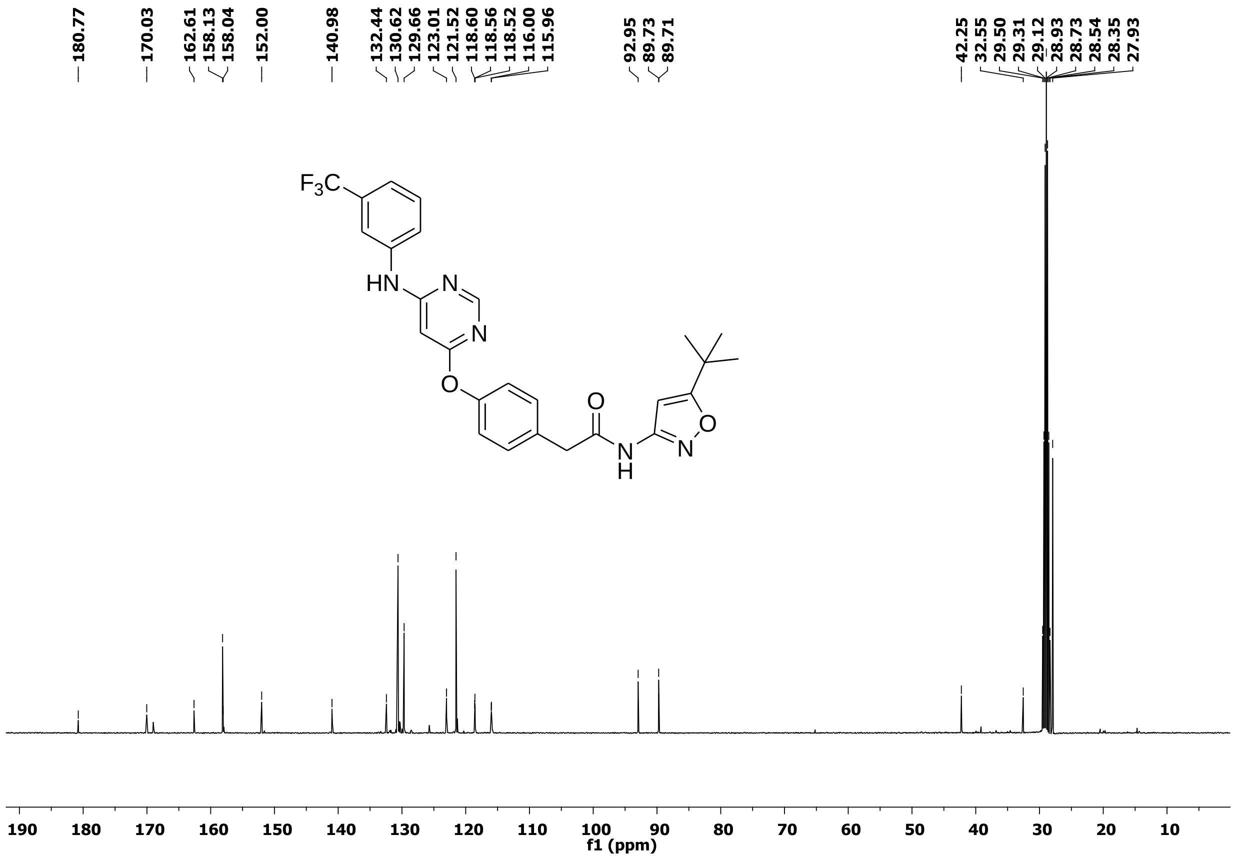

180.77
170.03
162.61
158.13
158.04
152.00
140.98
132.44
130.62
129.66
123.01
121.52
118.60
118.56
118.52
116.00
115.96
92.95
89.73
89.71
42.25
32.55
29.50
29.31
29.12
28.93
28.73
28.54
28.35
27.93
F3C
HN
N
N
O
O
N
H
N
O
190 180 170 160 150 140 130 120 110 100 90 80 70 60 50 40 30 20 10
f1 (ppm)

S29. ^{1}H, ^{13}C spectrum of 2-(4-(6-(3-(trifluoromethyl)phenylamino)pyrimidin-4-ylamino)phenyl)-N-(5-tert-butylisoxazol-3-yl)acetamide (13m)

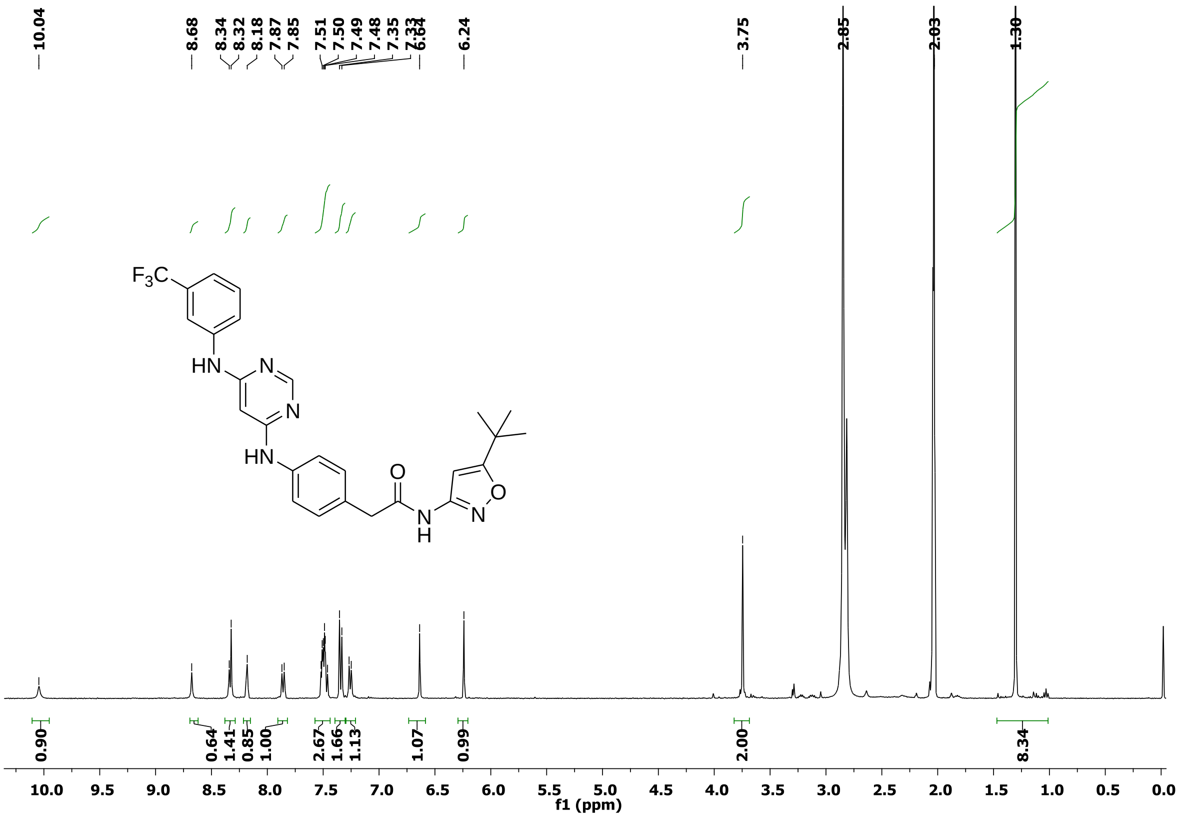

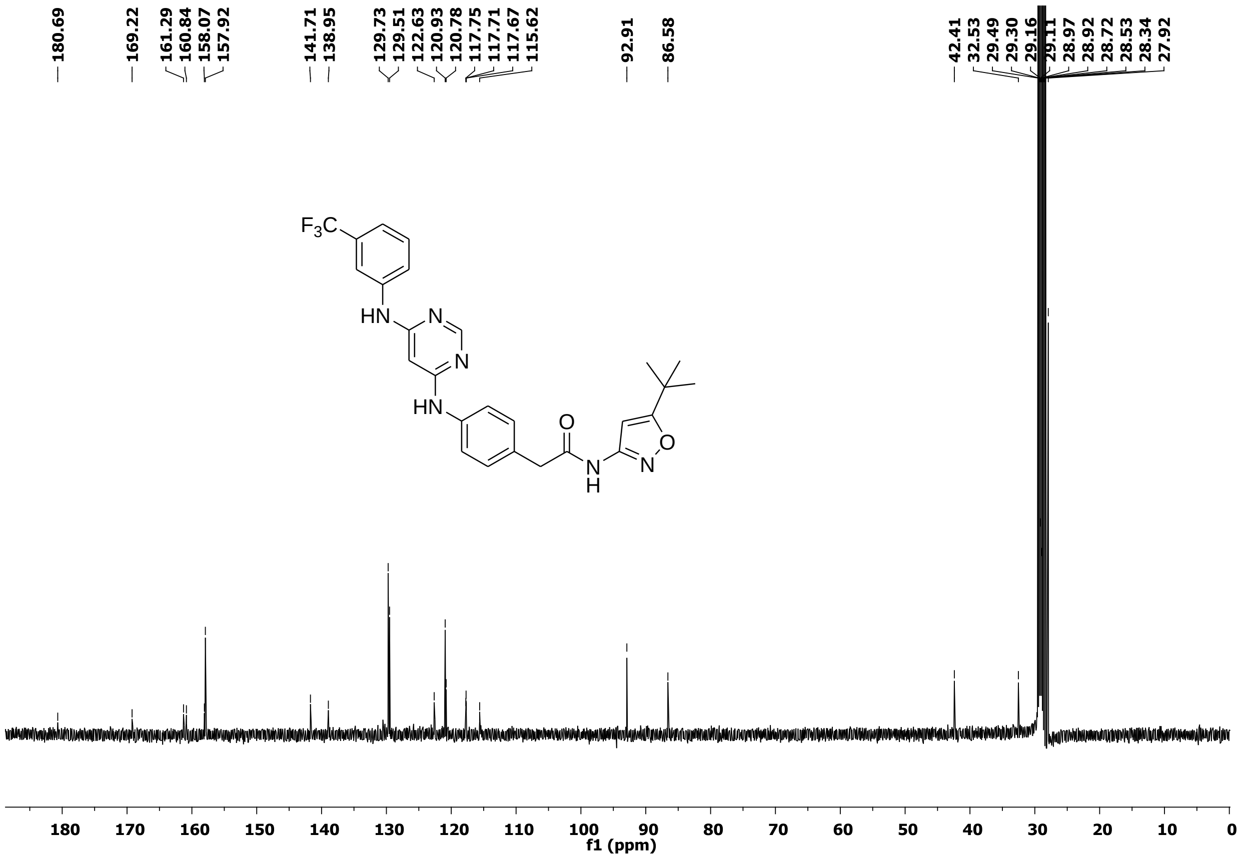
180.69
169.22
161.29
160.84
158.07
157.92
141.71
138.95
129.73
129.51
122.63
120.93
120.78
117.75
117.71
117.67
115.62
92.91
86.58
42.41
32.53
29.49
29.30
29.16
29.11
28.97
28.92
28.72
28.53
28.34
27.92
F3C
HN
N
N
HN
O
N
H
N
O
180
170
160
150
140
130
120
110
100
90
80
70
60
50
40
30
20
10
0
f1 (ppm)

S30. ^{1}H, ^{13}C spectrum of 2-(4-(6-(3-(pyrrolidin-1-yl)propylamino)pyrimidin-4-ylamino)phenyl)-N-(5-tert-butylisoxazol-3-yl)acetamide (13n)

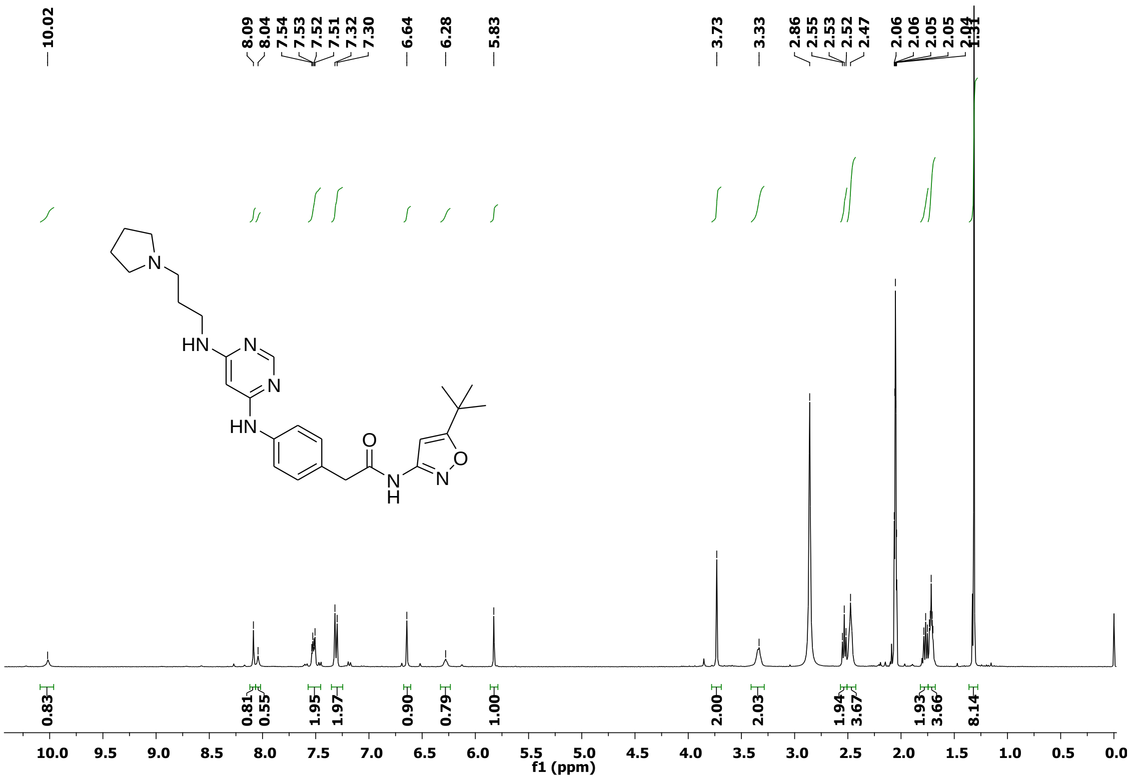

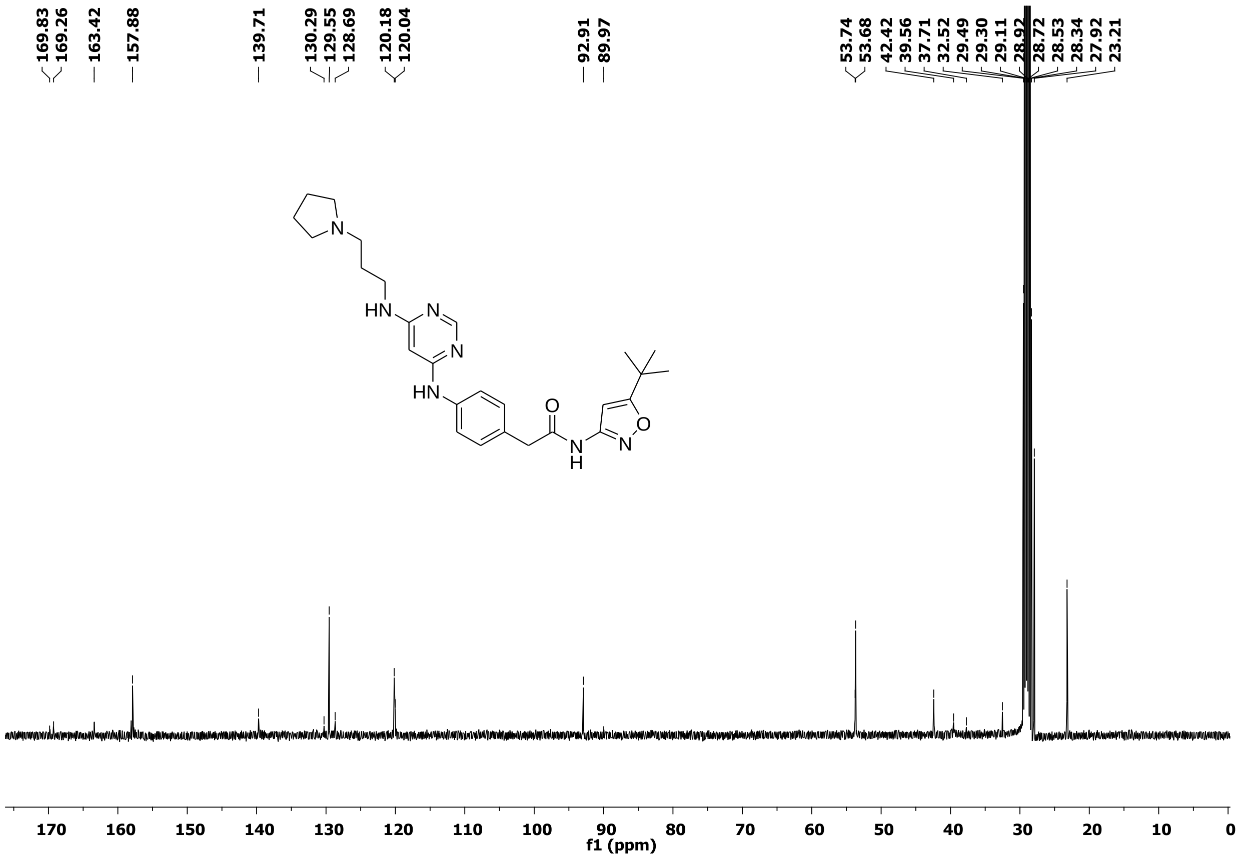
169.83
169.26
163.42
157.88
139.71
130.29
129.55
128.69
120.18
120.04
92.91
89.97
53.74
53.68
42.42
39.56
37.71
32.52
29.49
29.30
29.11
28.92
28.72
28.53
28.34
27.92
23.21
HN
N
N
HN
O
N
H
O
N
N
170
160
150
140
130
120
110
100
90
80
70
60
50
40
30
20
10
0
f1 (ppm)

S31. ^{1}H, ^{13}C spectrum of 2-(4-(6-(4-bromophenoxy)pyrimidin-4-ylamino)phenyl)-N-(5-tert-butylisoxazol-3-yl)acetamide (13o)

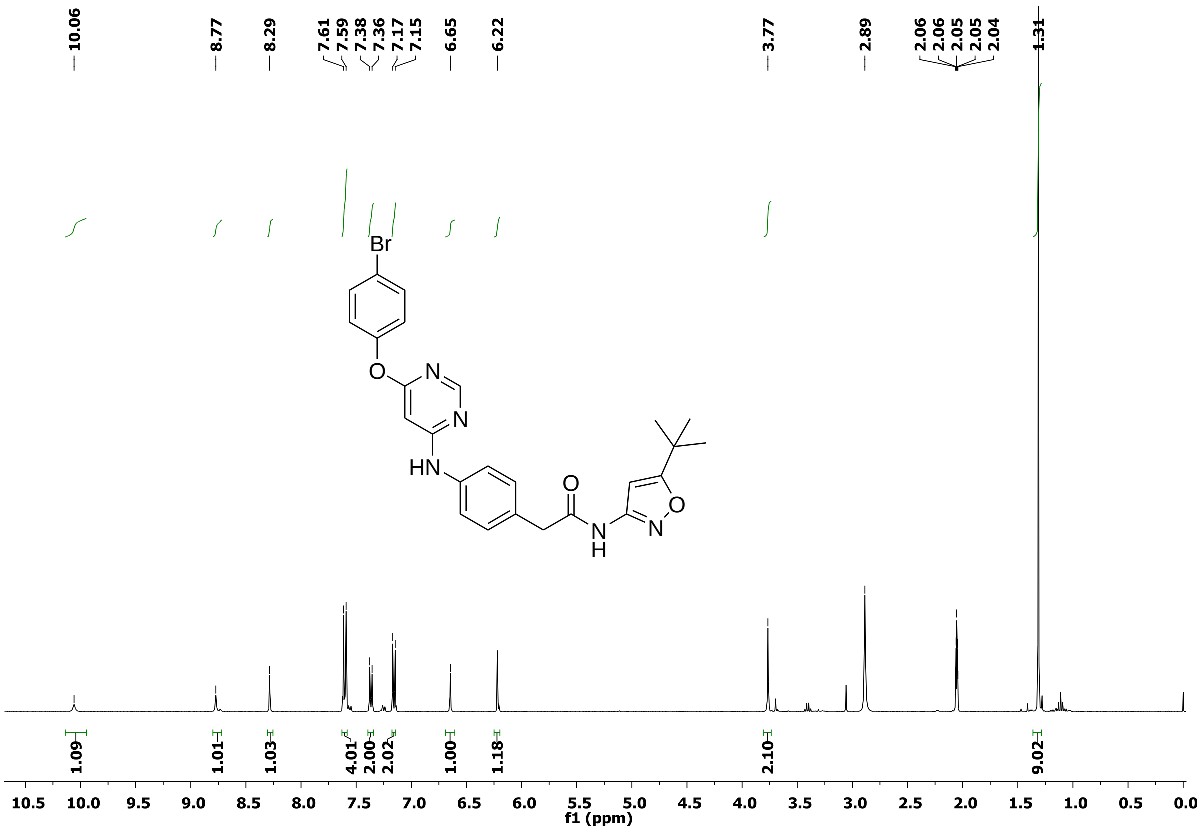

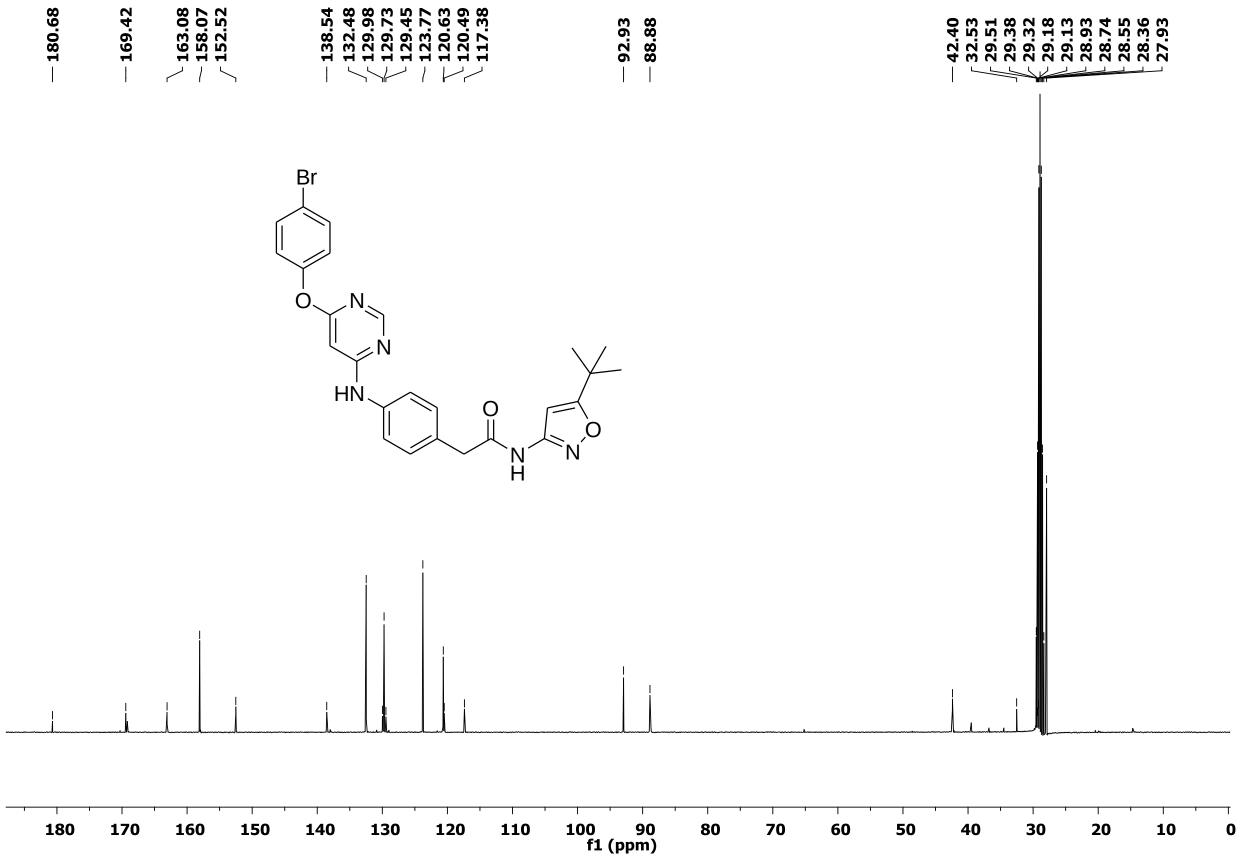

180.68
169.42
163.08
158.07
152.52
138.54
132.48
129.98
129.73
129.45
123.77
120.63
120.49
117.38
92.93
88.88
42.40
32.53
29.51
29.38
29.32
29.18
29.13
28.93
28.74
28.55
28.36
27.93
Br
O
N
N
HN
O
N
H
O
N
180
170
160
150
140
130
120
110
100
90
80
70
60
50
40
30
20
10
0
f1 (ppm)

S32. ^{1}H, ^{13}C spectrum of 2-(4-(6-morpholinopyrimidin-4-ylamino)phenyl)-N-(5-tert-butylisoxazol-3-yl)acetamide (13p)

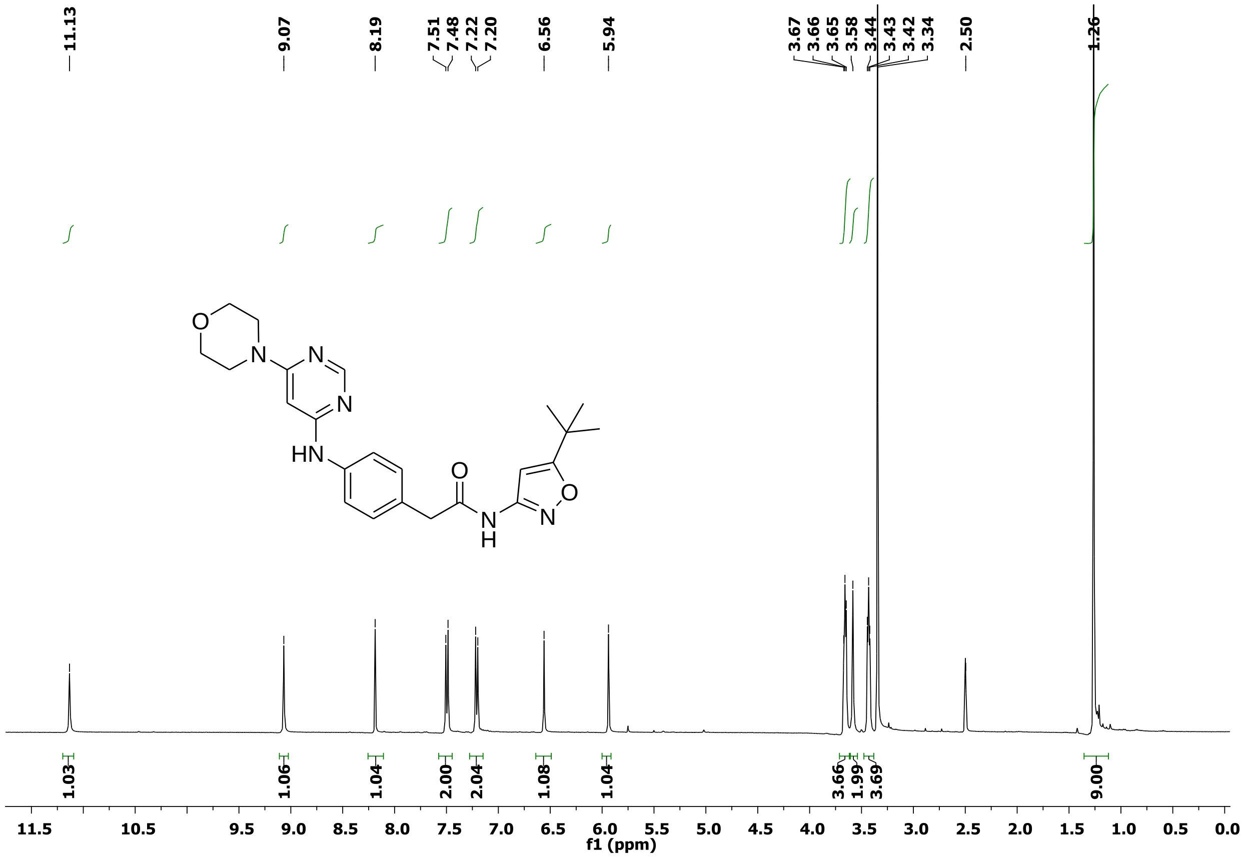

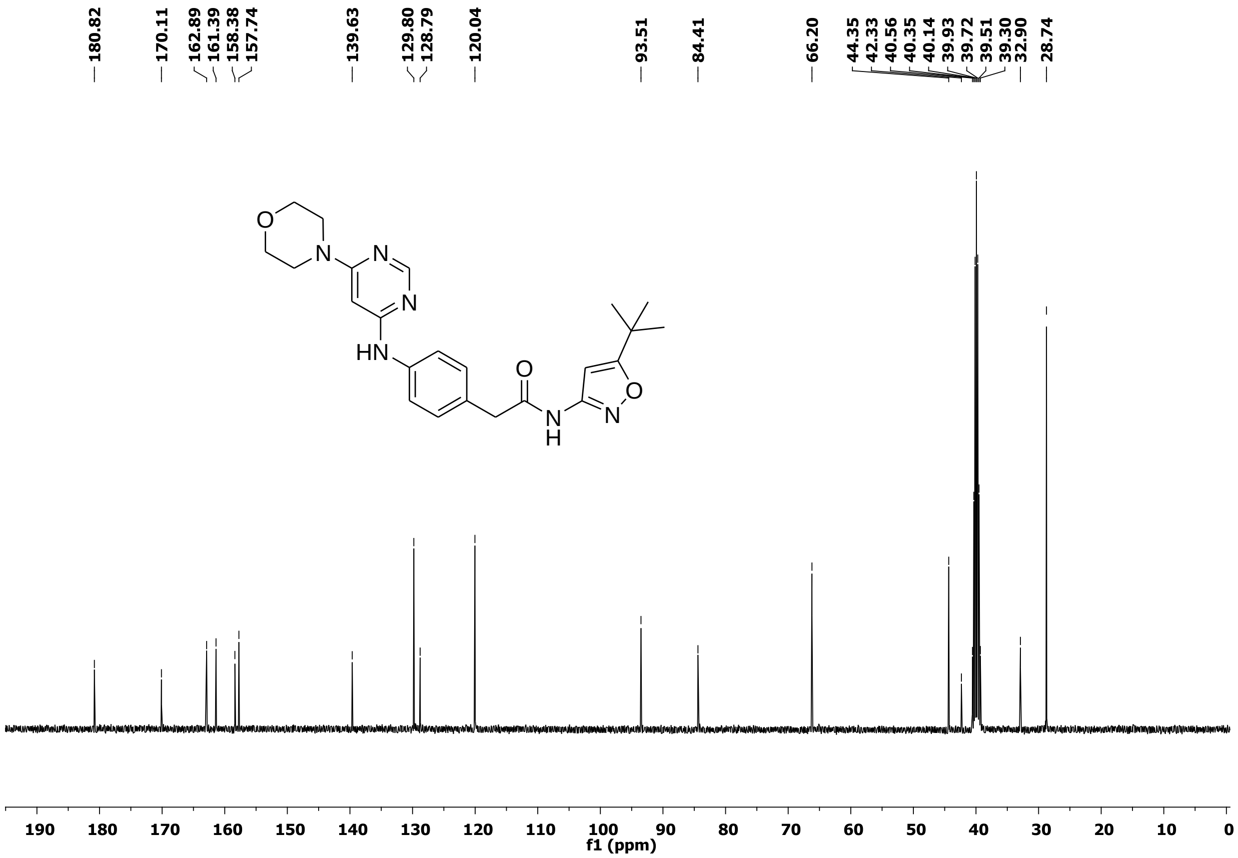
180.82
170.11
162.89
161.39
158.38
157.74
139.63
129.80
128.79
120.04
93.51
84.41
66.20
44.35
42.33
40.56
40.35
40.14
39.93
39.72
39.51
39.30
32.90
28.74
O
N
N
N
HN
O
N
H
N
O
190
180
170
160
150
140
130
120
110
100
90
80
70
60
50
40
30
20
10
0
f1 (ppm)

S33. ^{1}H, ^{13}C spectrum of 2-(4-(6-(dimethylamino)pyrimidin-4-yloxy)phenyl)-N-(5-tert-butylisoxazol -3-yl)acetamide (13q)

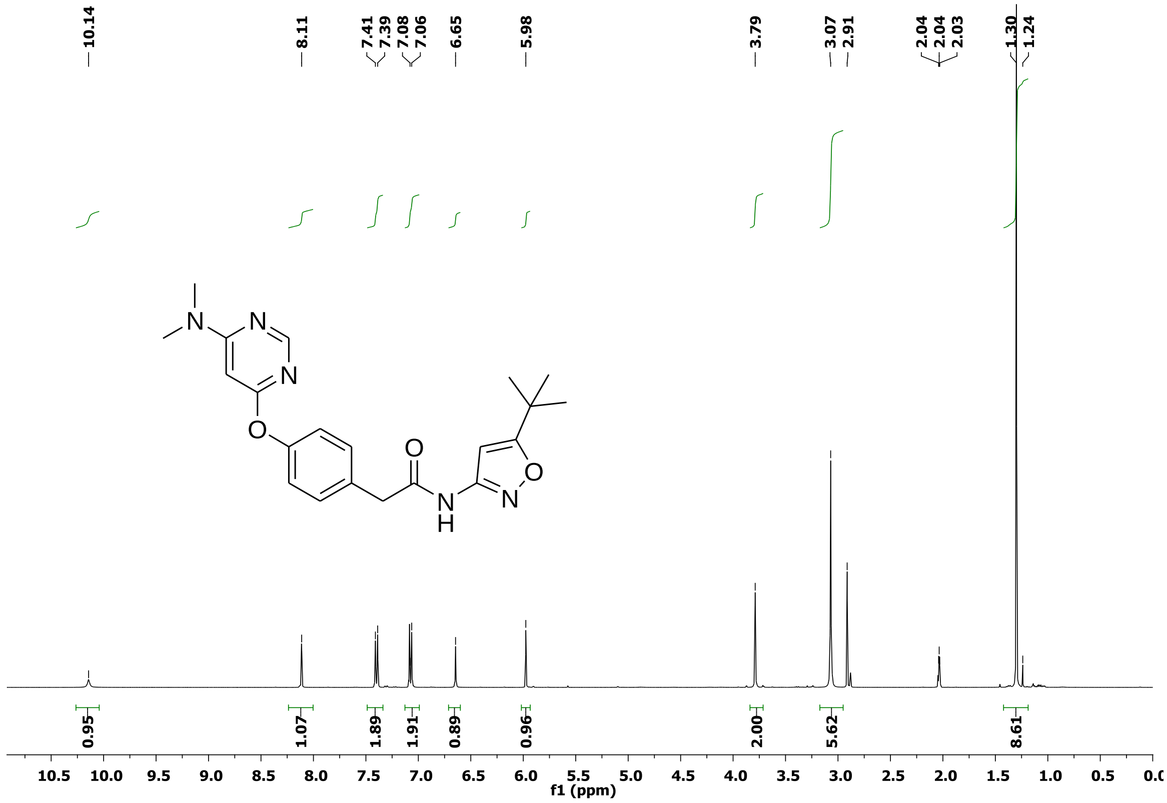

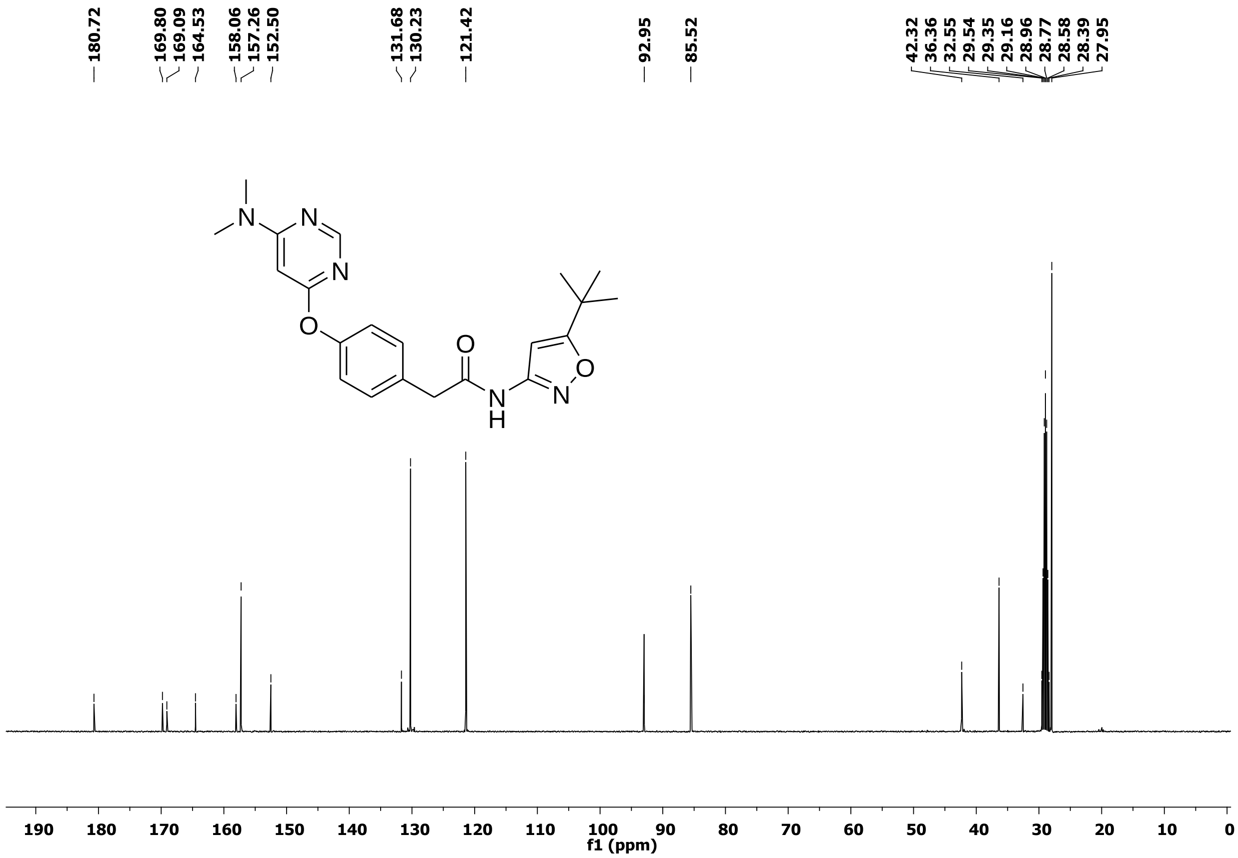

180.72
169.80
169.09
164.53
158.06
157.26
152.50
131.68
130.23
121.42
92.95
85.52
42.32
36.36
32.55
29.54
29.35
29.16
28.96
28.77
28.58
28.39
27.95
190 180 170 160 150 140 130 120 110 100 90 80 70 60 50 40 30 20 10 0
f1 (ppm)

S34. ^{1}H, ^{13}C spectrum of 2-(4-(6-(methylamino)pyrimidin-4-ylamino)phenyl)-N-(3-(trifluoromethyl) phenyl)acetamide (13r)

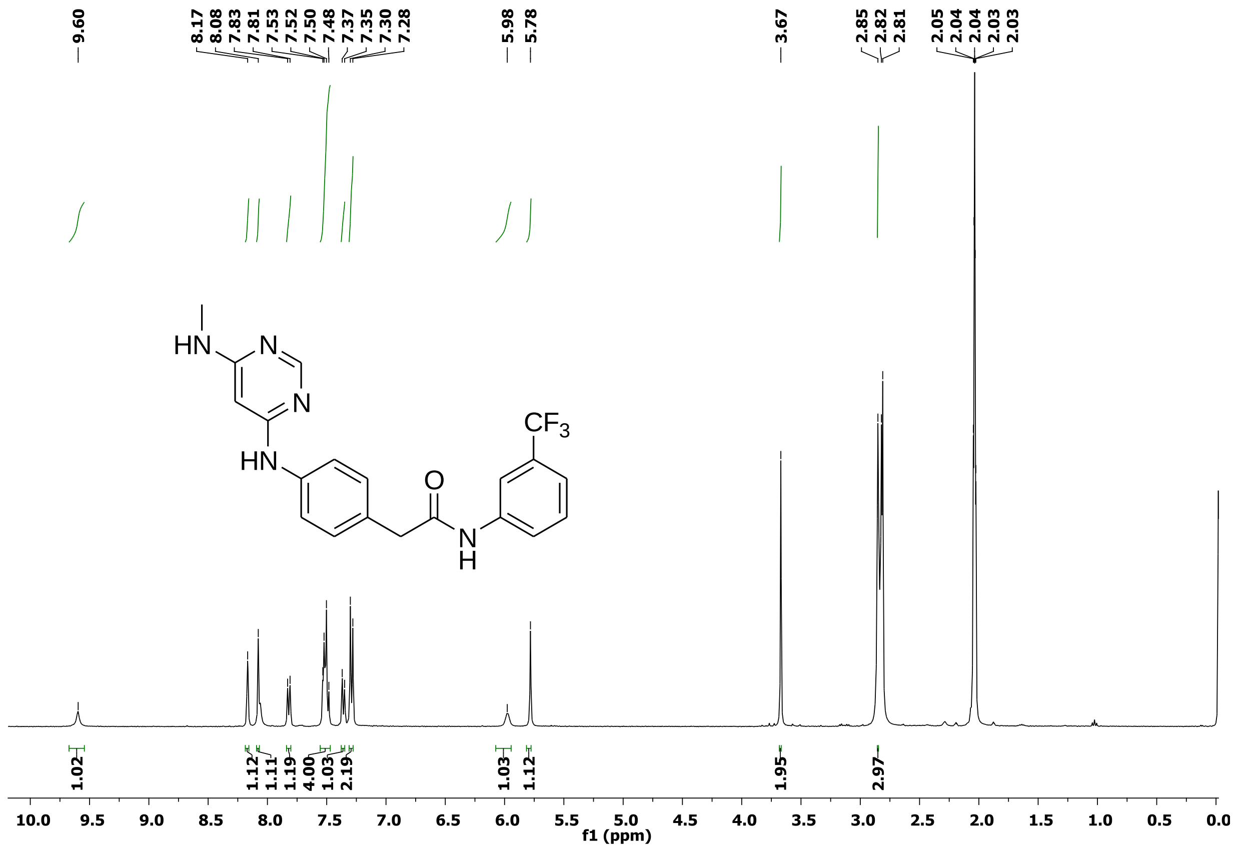

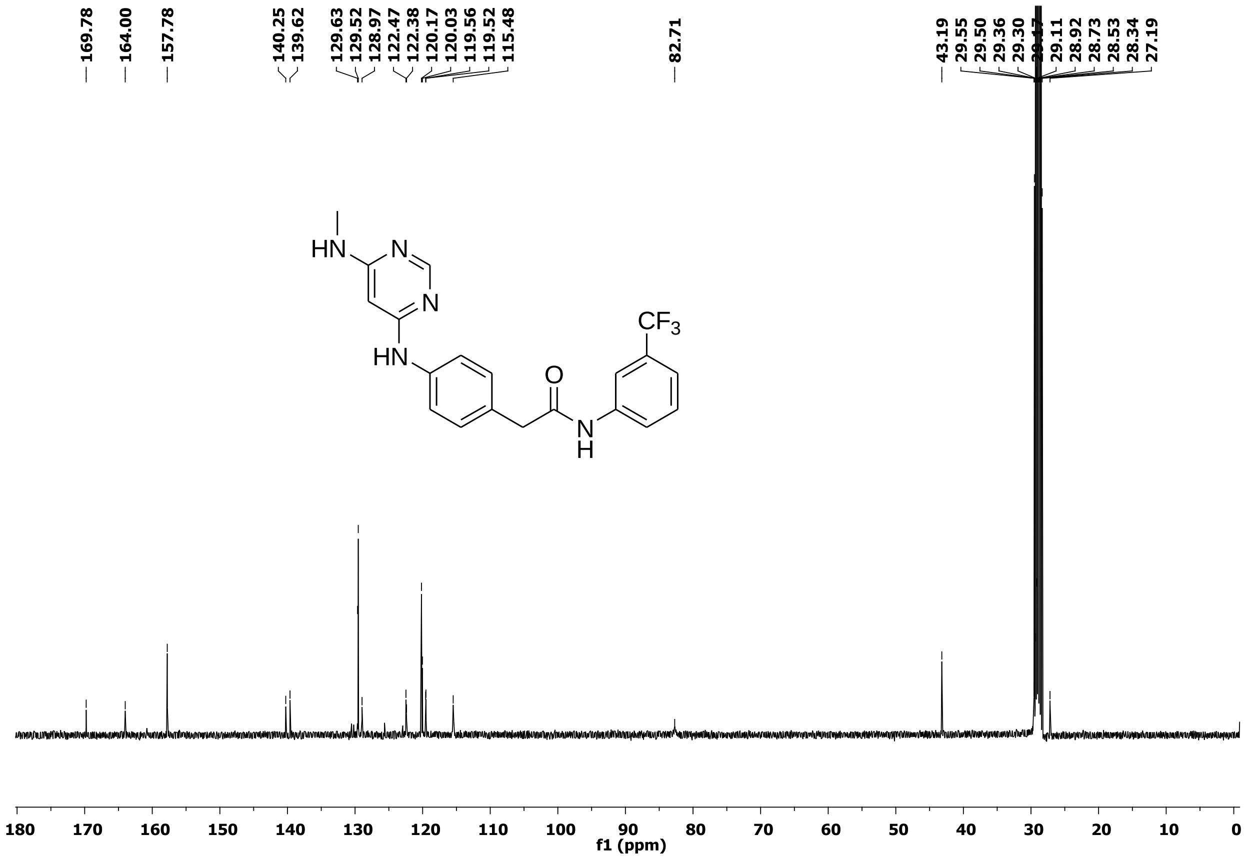

169.78
164.00
157.78
140.25
139.62
129.63
129.52
128.97
122.47
122.38
120.17
120.03
119.56
119.52
115.48
82.71
43.19
29.55
29.50
29.36
29.30
29.11
28.92
28.73
28.53
28.34
27.19
HN
N
N
HN
CF3
O
N
H
f1 (ppm)

S35. ^{1}H, ^{13}C spectrum of 2-(4-(6-(4-(methylsulfonyl)phenylamino)pyrimidin-4-ylamino)phenyl)-N-(3-(trifluoromethyl)phenyl)acetamide (13s)

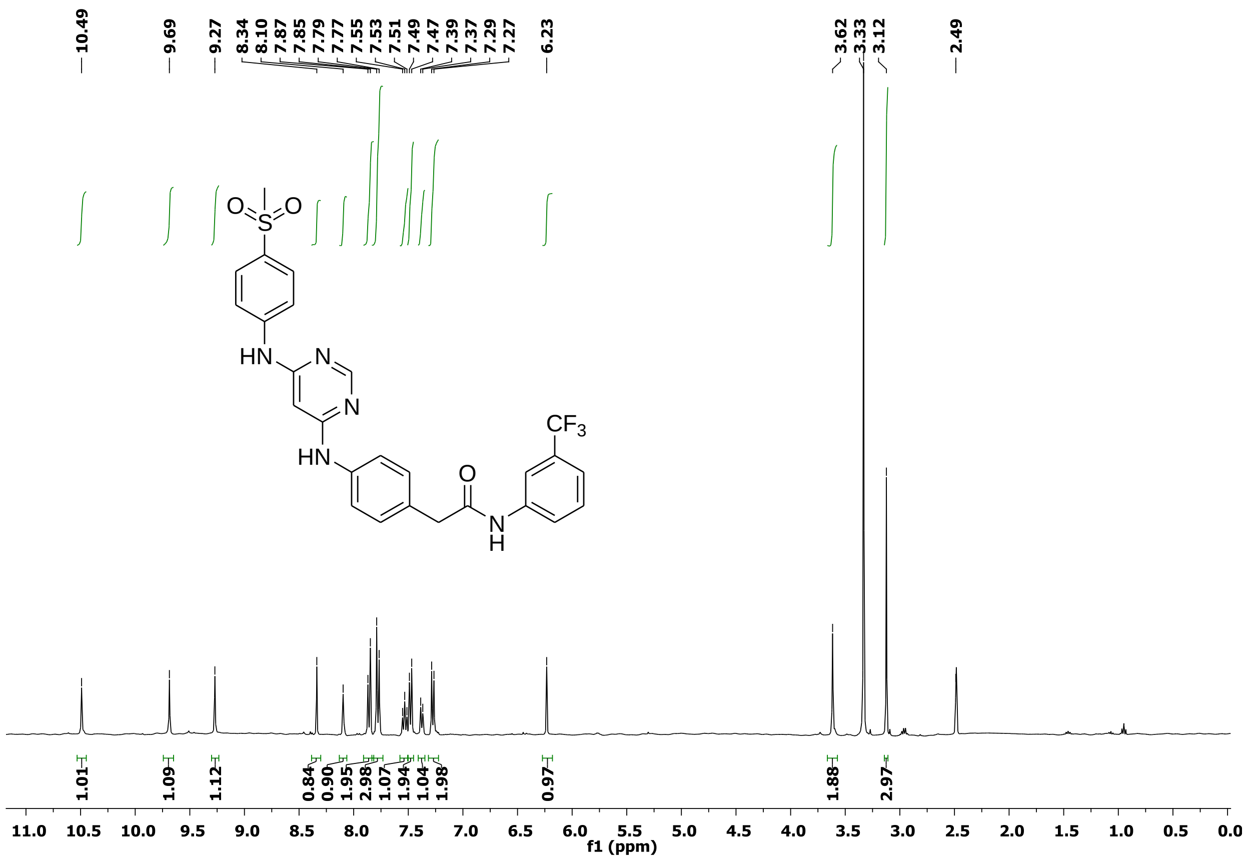

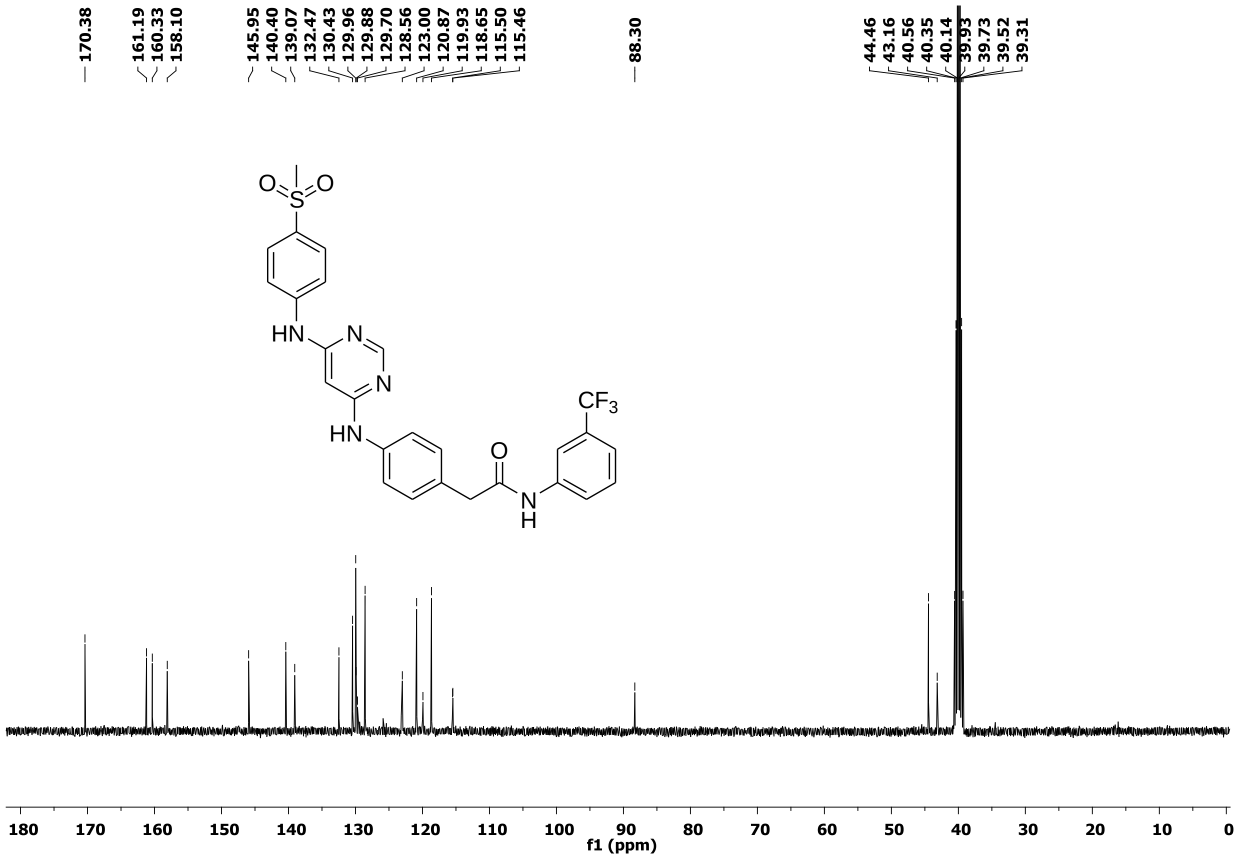

170.38
161.19
160.33
158.10
145.95
140.40
139.07
132.47
130.43
129.96
129.88
129.70
128.56
123.00
120.87
119.93
118.65
115.50
115.46
88.30
44.46
43.16
40.56
40.35
40.14
39.93
39.73
39.52
39.31
O
S
O
HN
N
N
HN
CF3
O
N
H
180
170
160
150
140
130
120
110
100
90
80
70
60
50
40
30
20
10
0
f1 (ppm)

S36. ^{1}H, ^{13}C spectrum of 2-(4-(6-(3-(pyrrolidin-1-yl)propylamino)pyrimidin-4-ylamino)phenyl)-N-(4-(trifluoromethoxy)phenyl)acetamide (13t)

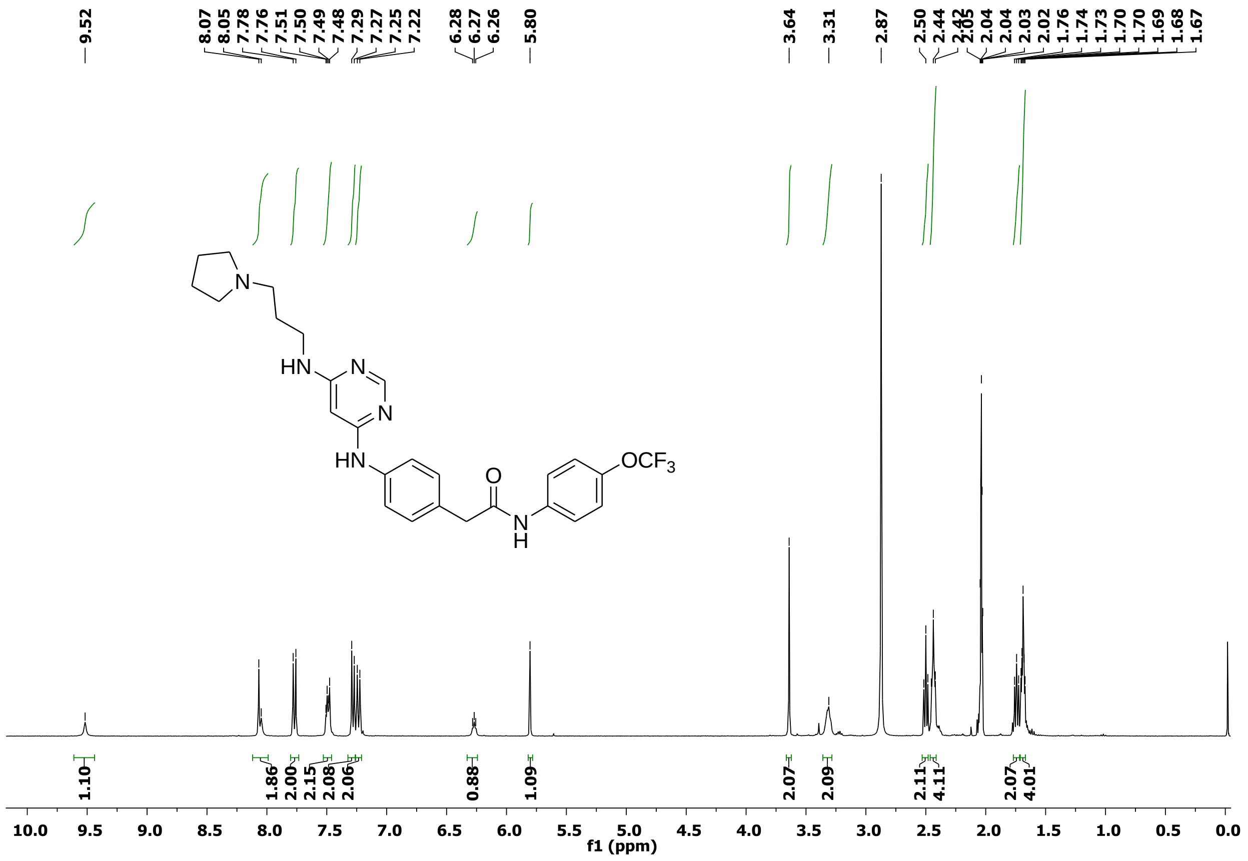

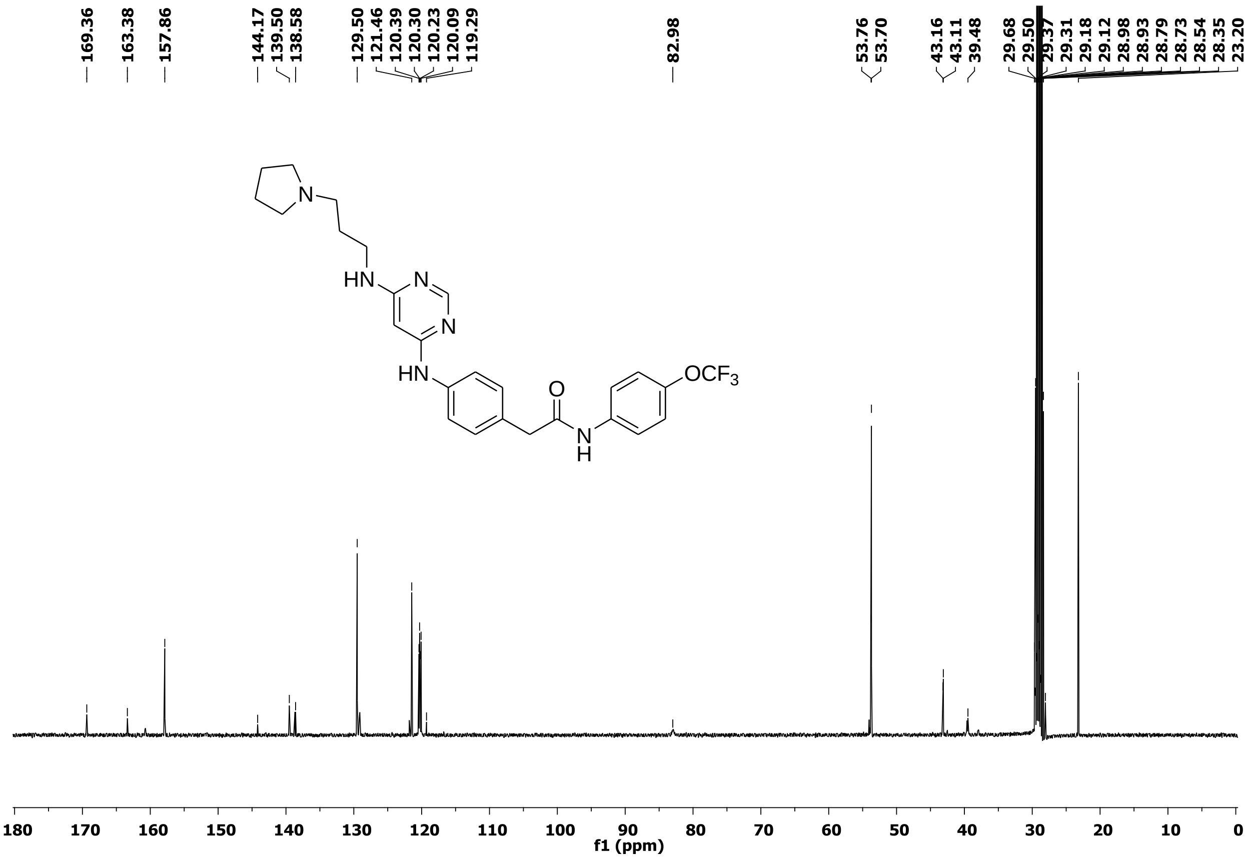

169.36
163.38
157.86
144.17
139.50
138.58
129.50
121.46
120.39
120.30
120.23
120.09
119.29
82.98
53.76
53.70
43.16
43.11
39.48
29.68
29.50
29.37
29.31
29.18
29.12
28.98
28.93
28.79
28.73
28.54
28.35
23.20
N
HN
N
N
HN
O
N
H
OCF3
180 170 160 150 140 130 120 110 100 90 80 70 60 50 40 30 20 10 0
f1 (ppm)

S37. ^{1}H, ^{13}C spectrum of 2-(4-(6-(2,3-dihydrobenzo[b][1,4]dioxin-5-ylamino)pyrimidin-4-ylamino)phenyl)-N-(4-(trifluoromethoxy)phenyl)acetamide (13u)

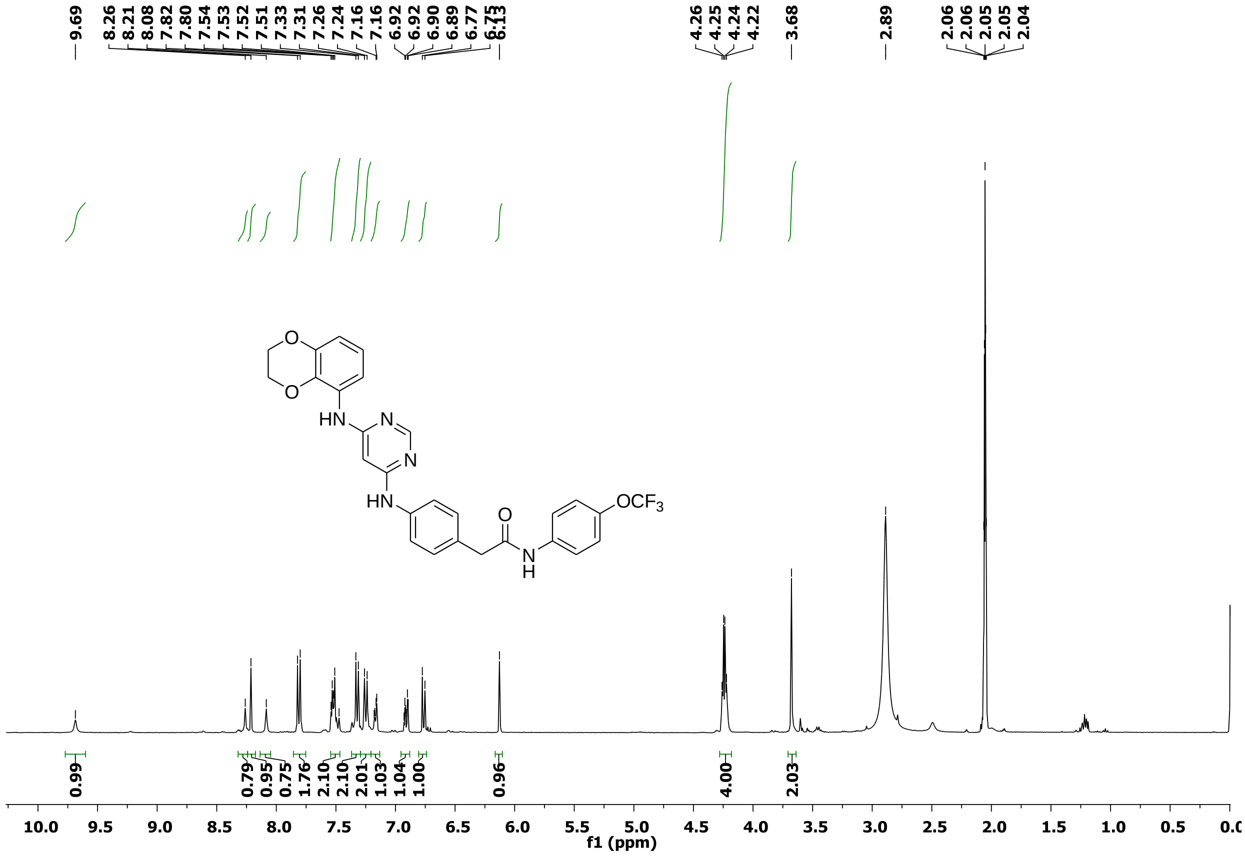

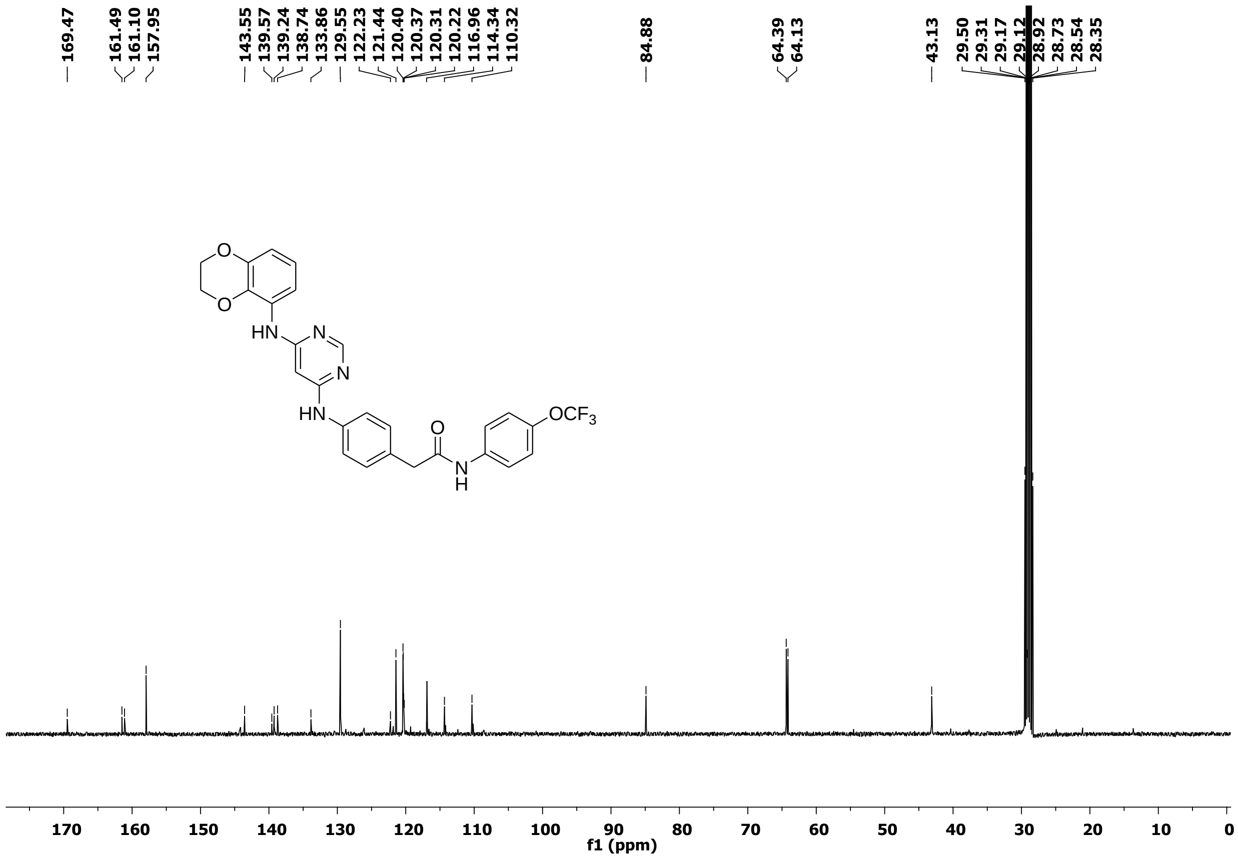
169.47
161.49
161.10
157.95
143.55
139.57
139.24
138.74
133.86
129.55
122.23
121.44
120.40
120.37
120.31
120.22
116.96
114.34
110.32
84.88
64.39
64.13
43.13
29.50
29.31
29.17
29.12
28.92
28.73
28.54
28.35
HN
N
N
HN
O
O
O
N
H
OCF3
170
160
150
140
130
120
110
100
90
80
70
60
50
40
30
20
10
0
f1 (ppm)

S38. ^{1}H, ^{13}C spectrum of 2-(4-(6-(methylamino)pyrimidin-4-ylamino)phenyl)-N-(4-(methylsulfonyl) phenyl)acetamide (13v)

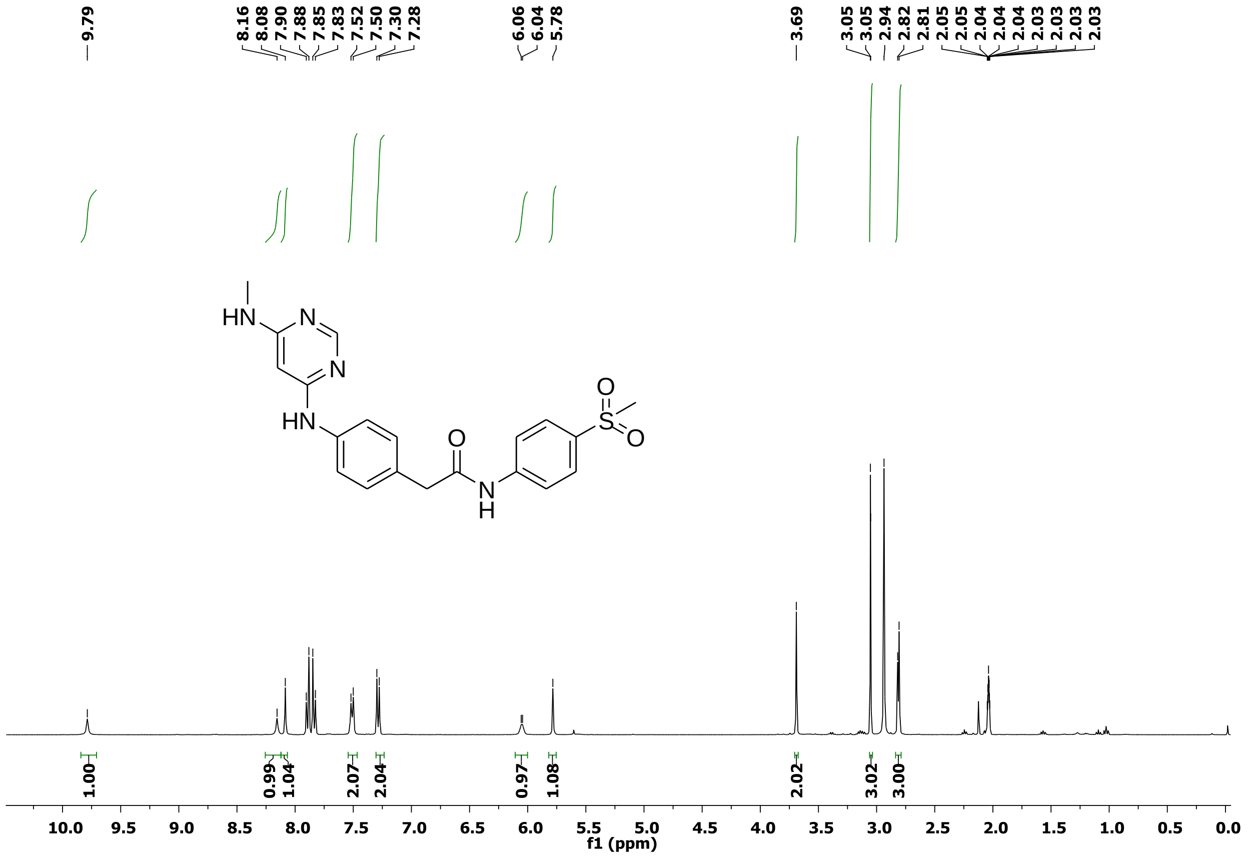

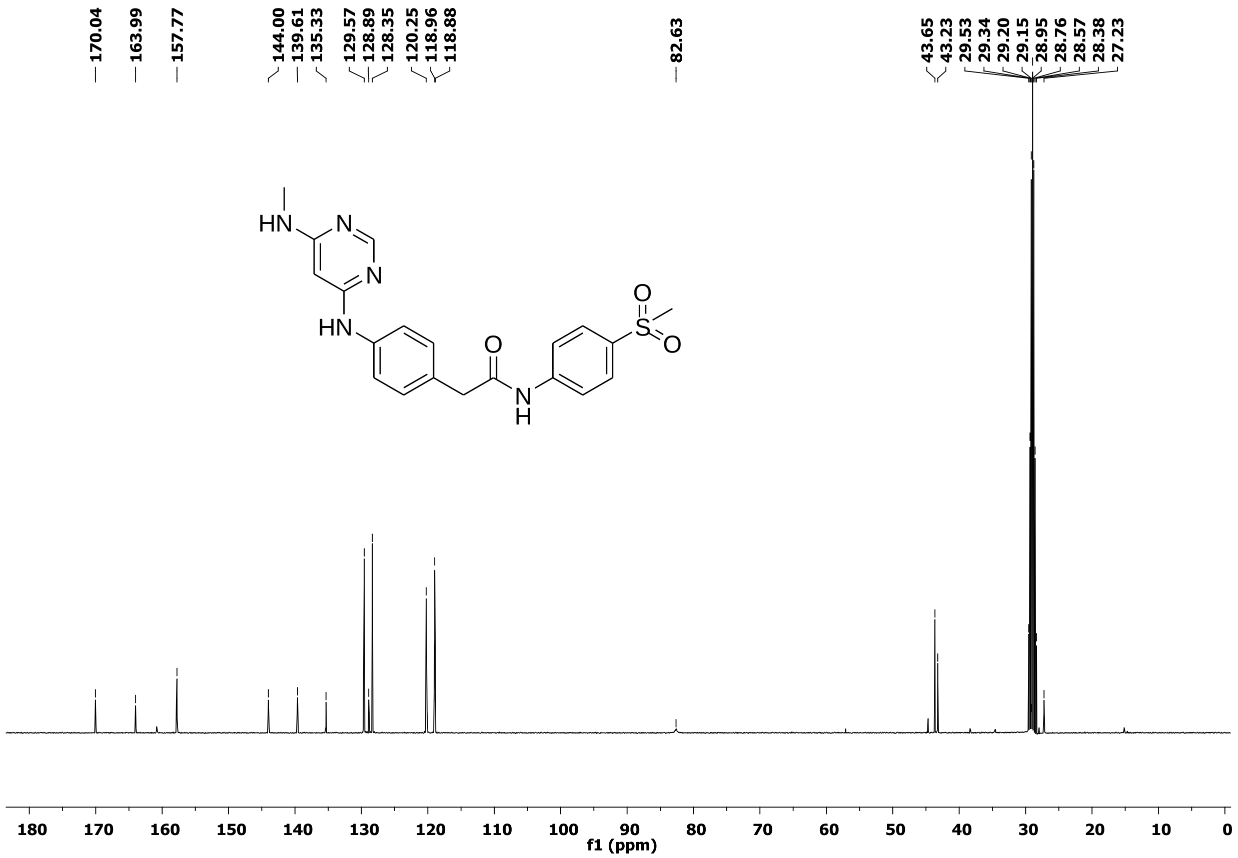

170.04
163.99
157.77
144.00
139.61
135.33
129.57
128.89
128.35
120.25
118.96
118.88
82.63
43.65
43.23
29.53
29.34
29.20
29.15
28.95
28.76
28.57
28.38
27.23
HN
N
N
HN
O
N
H
O
S
O
180 170 160 150 140 130 120 110 100 90 80 70 60 50 40 30 20 10 0
f1 (ppm)

S39. ^{1}H, ^{13}C spectrum of N-(4-fluorobenzyl)-2-(4-(6-(3-(pyrrolidin-1-yl)propylamino)pyrimidin-4-yloxy)phenyl)acetamide (13w)

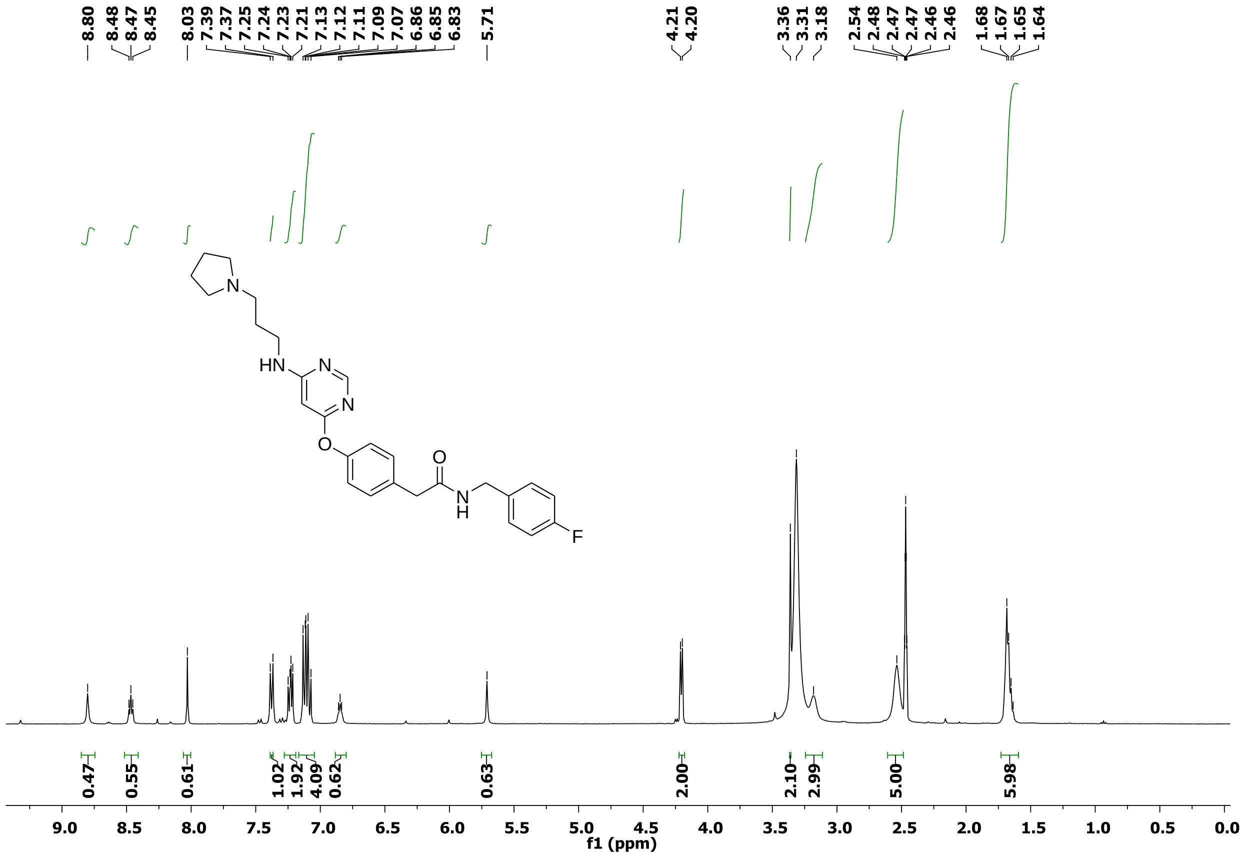

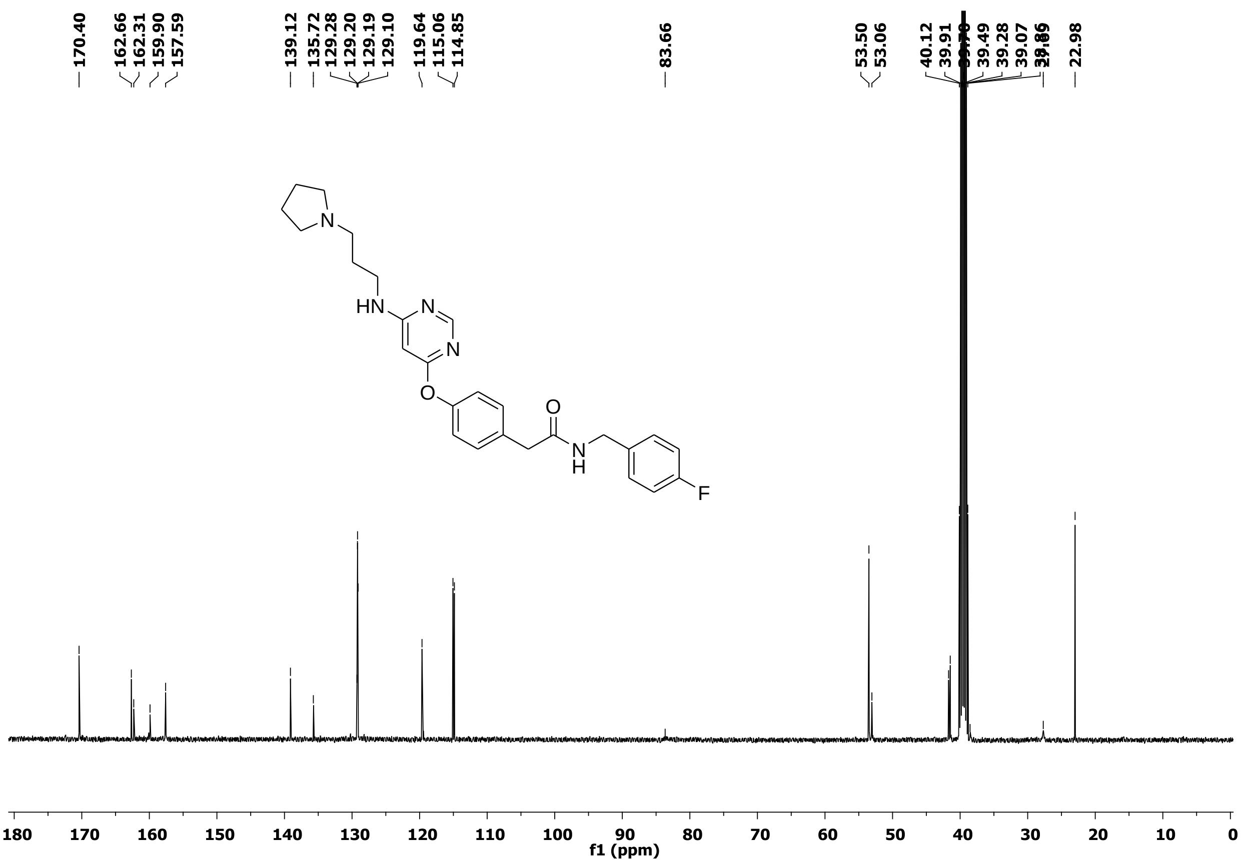

170.40
162.66
162.31
159.90
157.59
139.12
135.72
129.28
129.20
129.19
129.10
119.64
115.06
114.85
83.66
53.50
53.06
40.12
39.91
39.49
39.28
39.07
22.98
N
HN
N
N
O
O
N
H
F
180
170
160
150
140
130
120
110
100
90
80
70
60
50
40
30
20
10
0
f1 (ppm)

S40. ^{1}H, ^{13}C spectrum of 2-(4-(6-(methylamino)pyrimidin-4-yloxy)phenyl)-N-(4-tert-butylphenyl) Acetamide (13x)

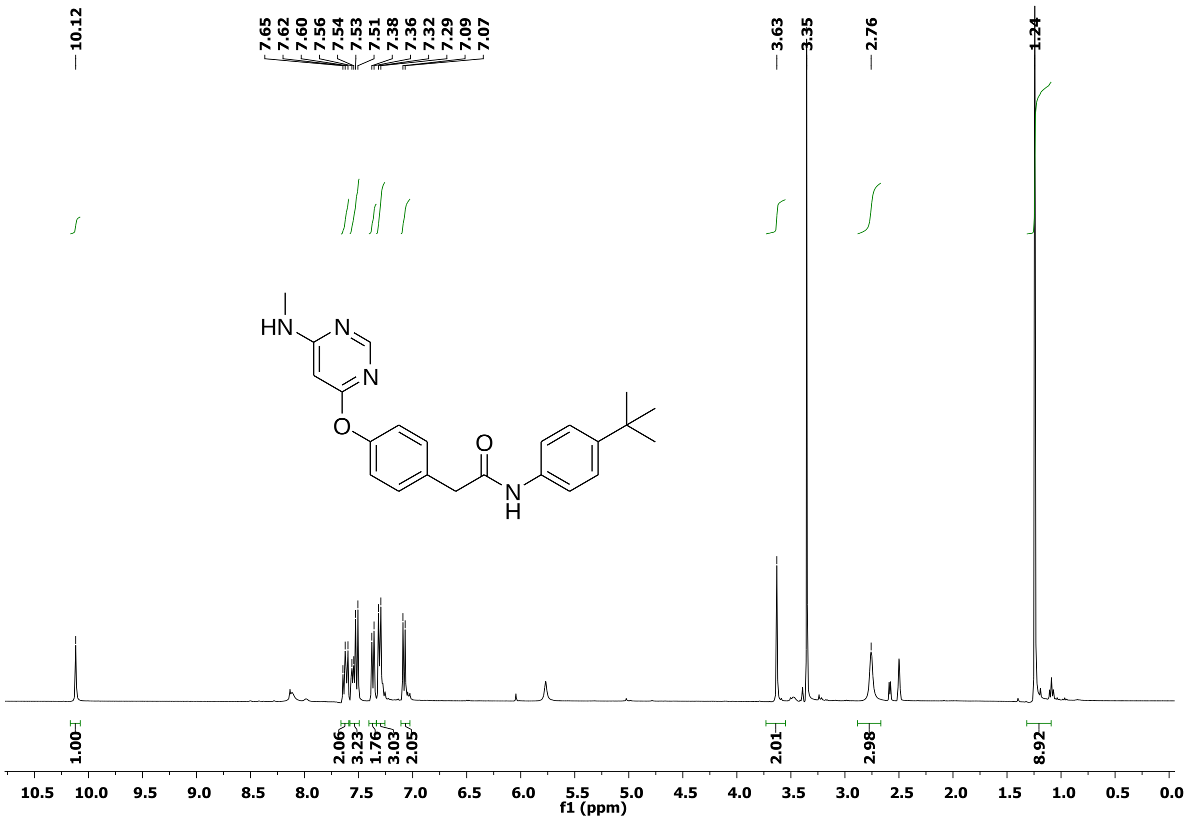

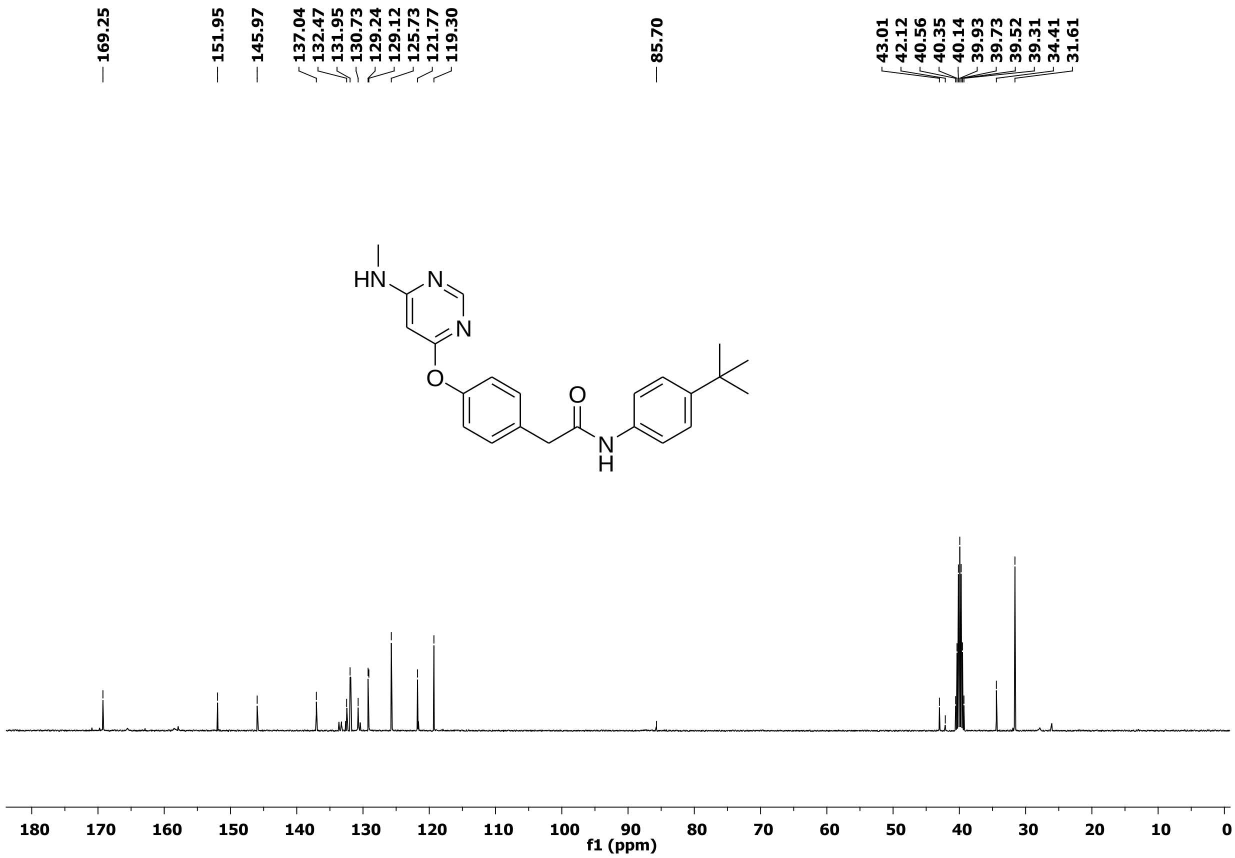
169.25
151.95
145.97
137.04
132.47
131.95
130.73
129.24
129.12
125.73
121.77
119.30
85.70
43.01
42.12
40.56
40.35
40.14
39.93
39.73
39.52
39.31
34.41
31.61
HN
N
N
O
O
N
H
180
170
160
150
140
130
120
110
100
90
80
70
60
50
40
30
20
10
0
f1 (ppm)

S41. ^{1}H, ^{13}C spectrum of 2-(4-(6-(3-(piperidin-1-yl)propylamino)pyrimidin-4-ylamino)phenyl)-N-(4-(trifluoromethoxy)phenyl)acetamide (13y)

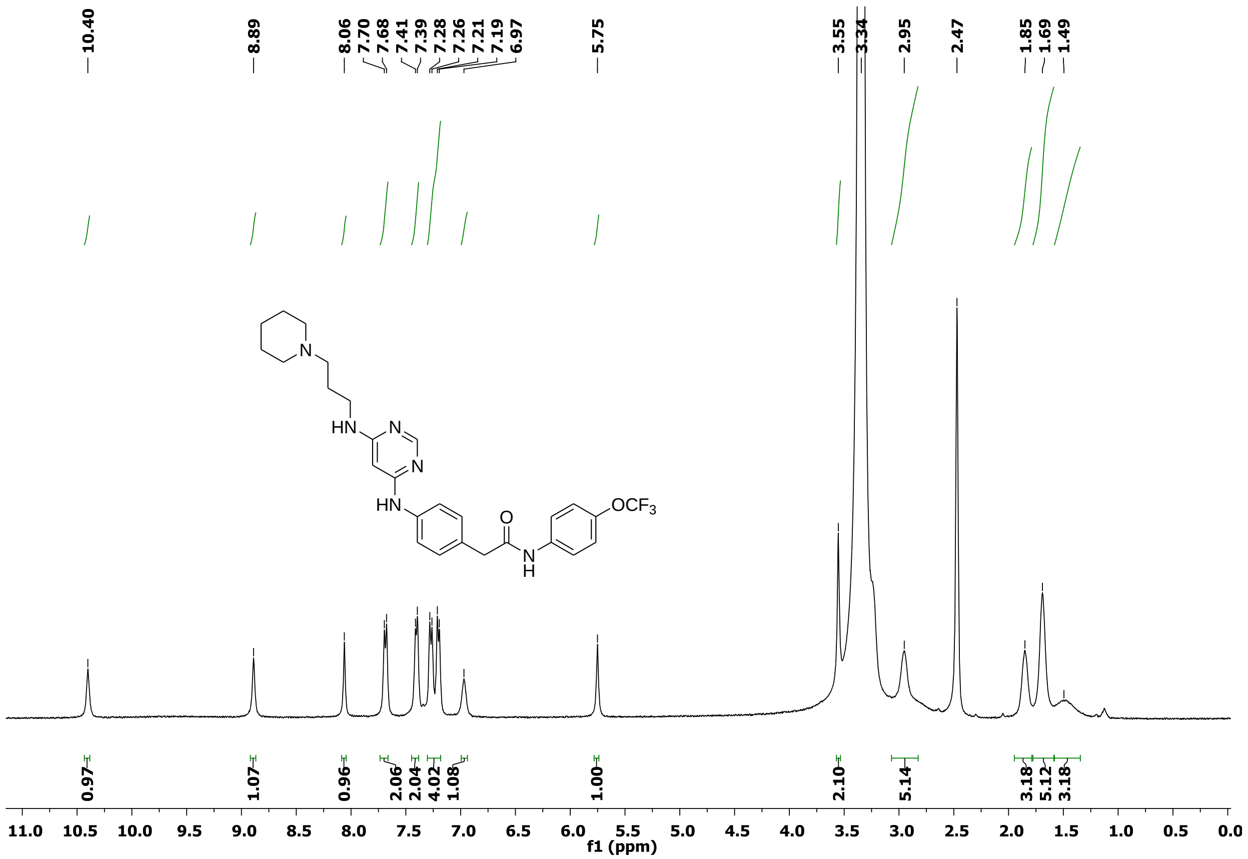

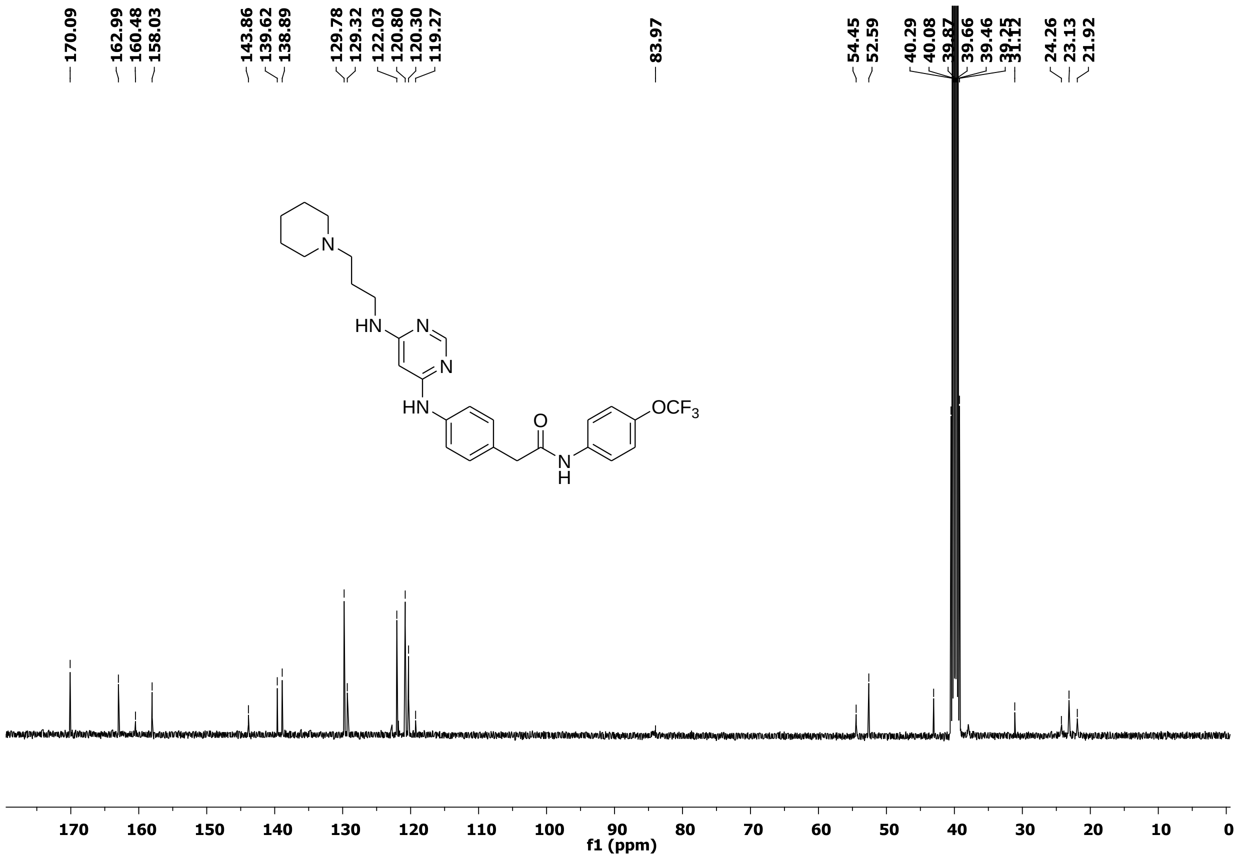
170.09
162.99
160.48
158.03
143.86
139.62
138.89
129.78
129.32
122.03
120.80
120.30
119.27
83.97
54.45
52.59
40.29
40.08
39.87
39.66
39.46
39.25
31.12
24.26
23.13
21.92
HN
N
N
HN
O
N
H
OCF3
170
160
150
140
130
120
110
100
90
80
70
60
50
40
30
20
10
0
f1 (ppm)

S42. ^{1}H, ^{13}C spectrum of 2-(4-(6-(methylamino)pyrimidin-4-ylamino)phenyl)-N-(4-(trifluoromethoxy)phenyl)acetamide (13z)

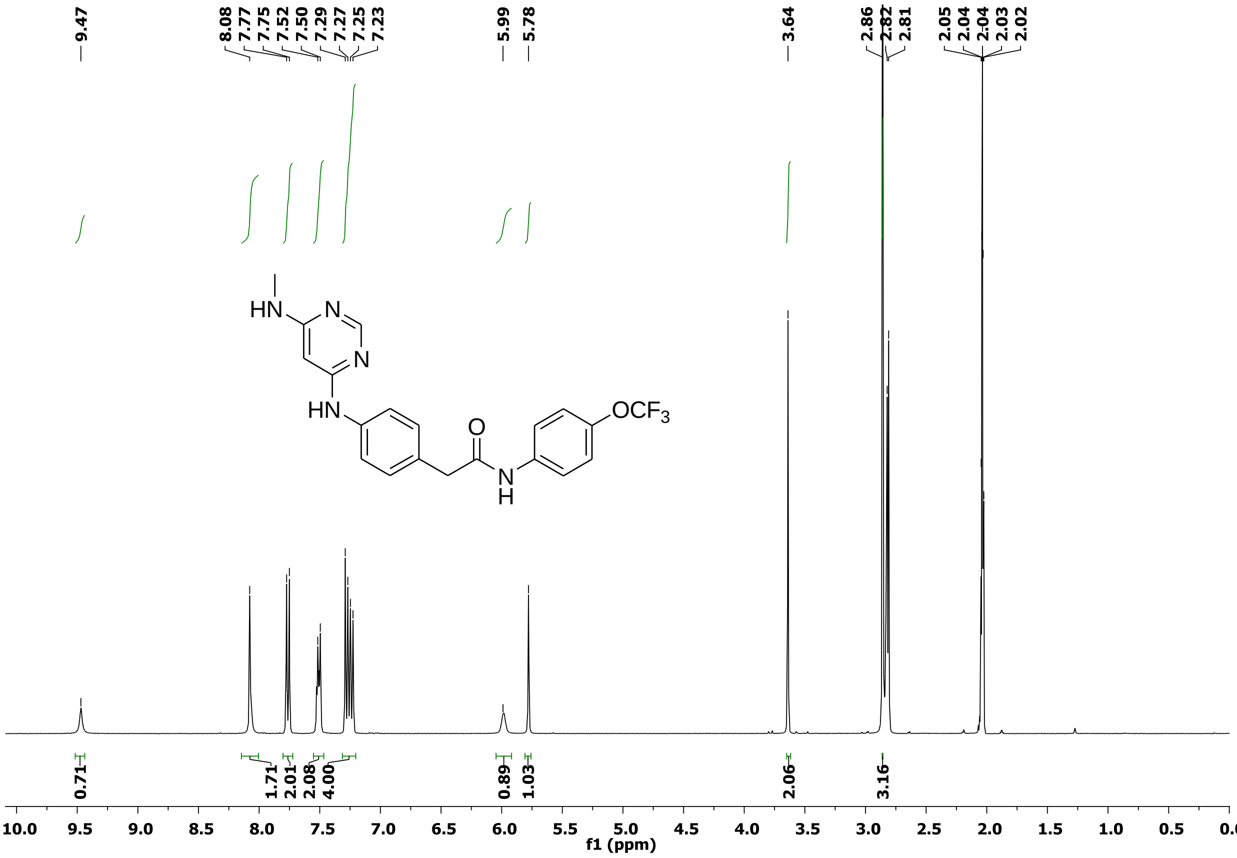

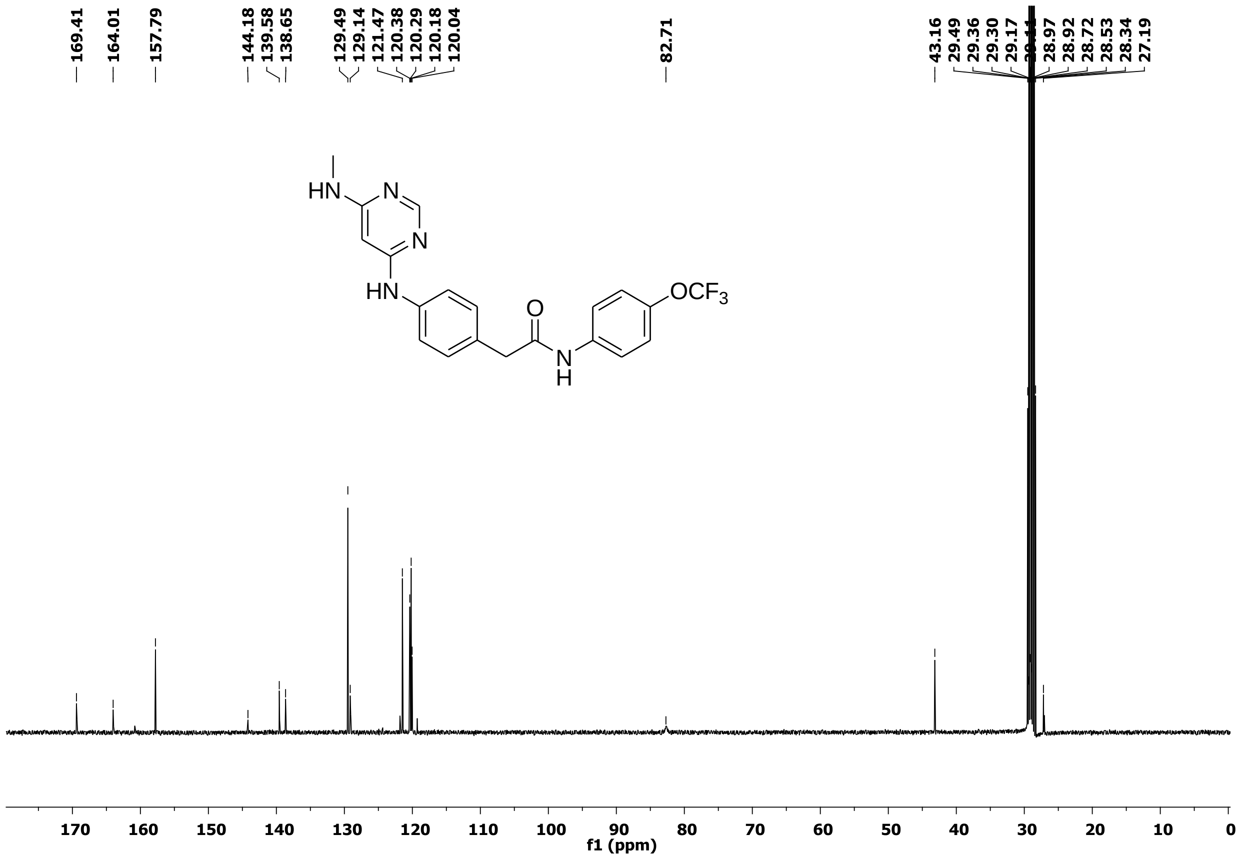
169.41
164.01
157.79
144.18
139.58
138.65
129.49
129.14
121.47
120.38
120.29
120.18
120.04
82.71
43.16
29.49
29.36
29.30
29.17
28.97
28.92
28.72
28.53
28.34
27.19
HN
N
N
HN
O
N
H
OCF3
170
160
150
140
130
120
110
100
90
80
70
60
50
40
30
20
10
0
f1 (ppm)

S43. ^{1}H, ^{13}C spectrum of 2-(4-(6-(4-(methylsulfonyl)phenylamino)pyrimidin-4-yloxy)phenyl)-N-(3-fluorophenyl)acetamide (13aa)

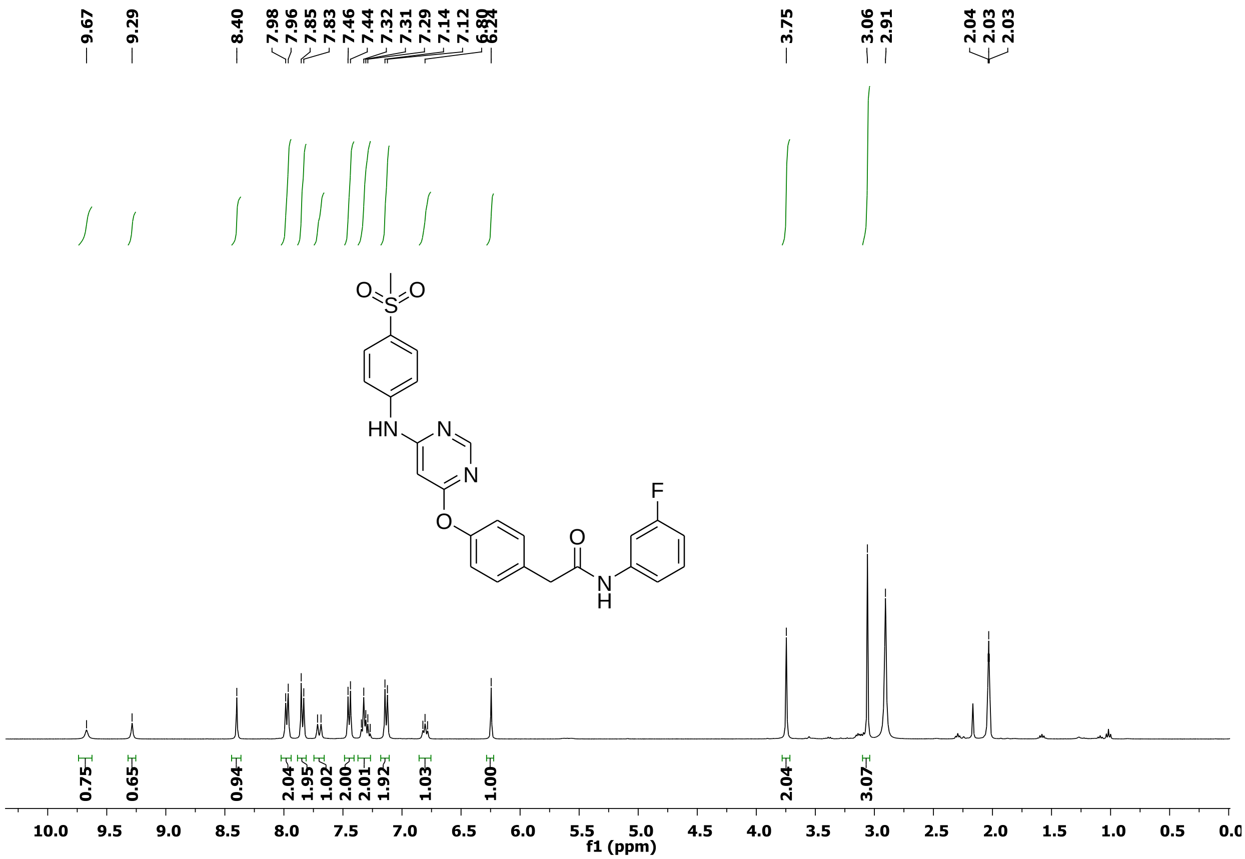

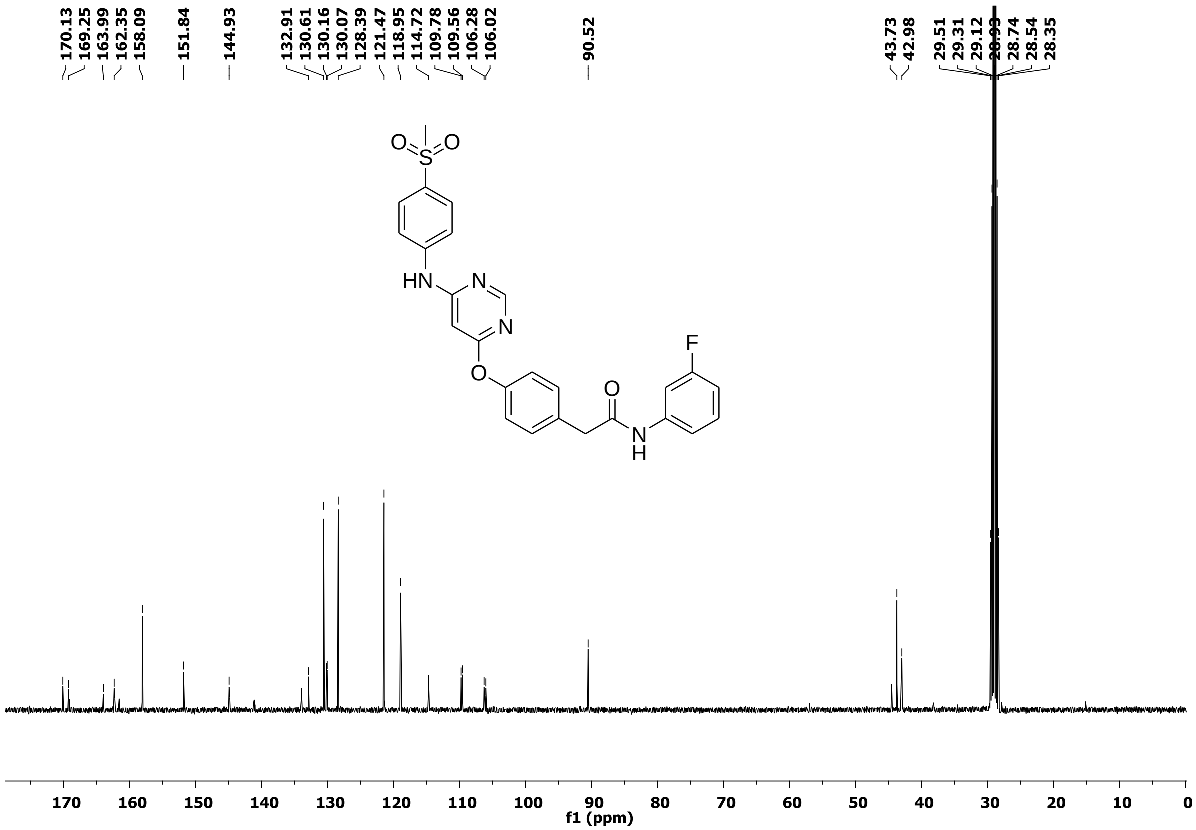
170.13
169.25
163.99
162.35
158.09
151.84
144.93
132.91
130.61
130.16
130.07
128.39
121.47
118.95
114.72
109.78
109.56
106.28
106.02
90.52
43.73
42.98
29.51
29.31
29.12
28.74
28.54
28.35
f1 (ppm)

S44. ^{1}H, ^{13}C spectrum of 2-(4-(6-(3-(pyrrolidin-1-yl)propylamino)pyrimidin-4-ylamino)phenyl)-N-(3-(trifluoromethyl)phenyl)acetamide (13ab)

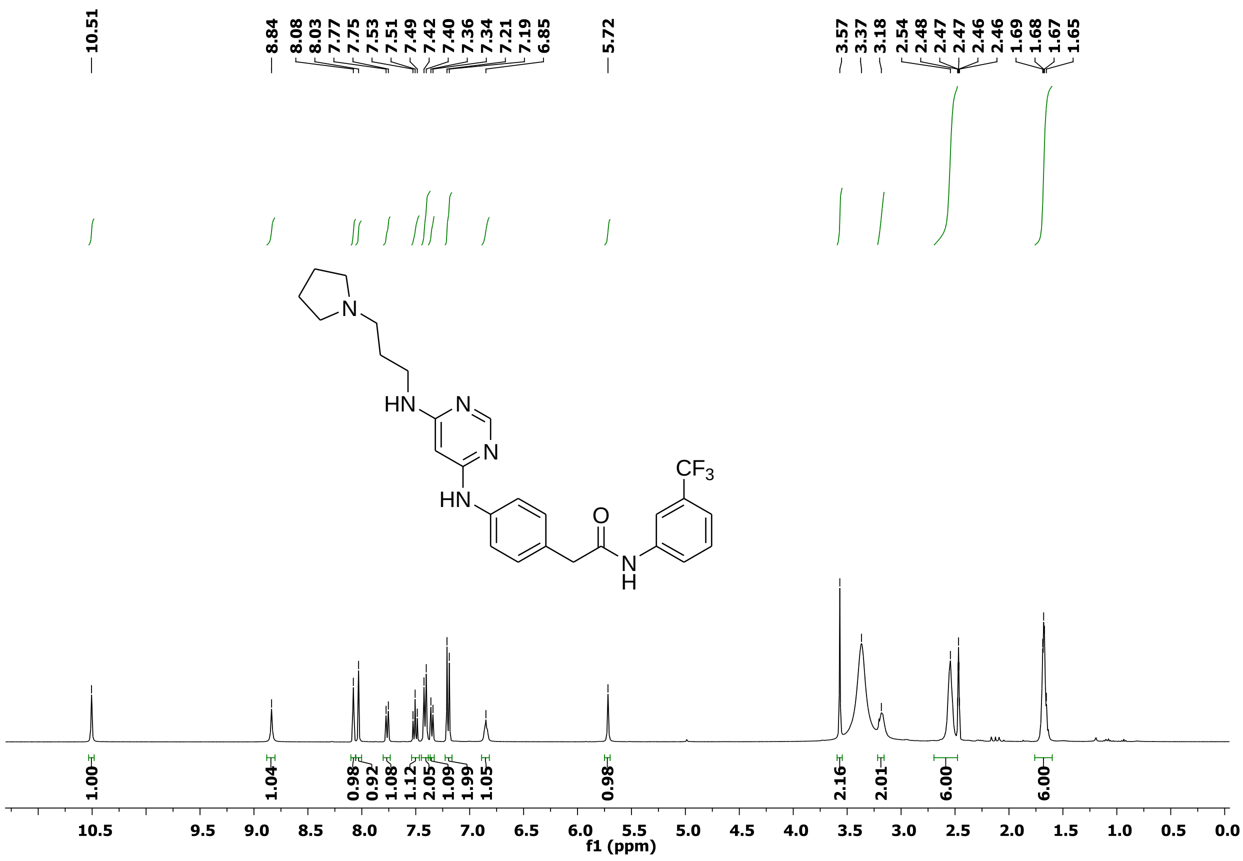

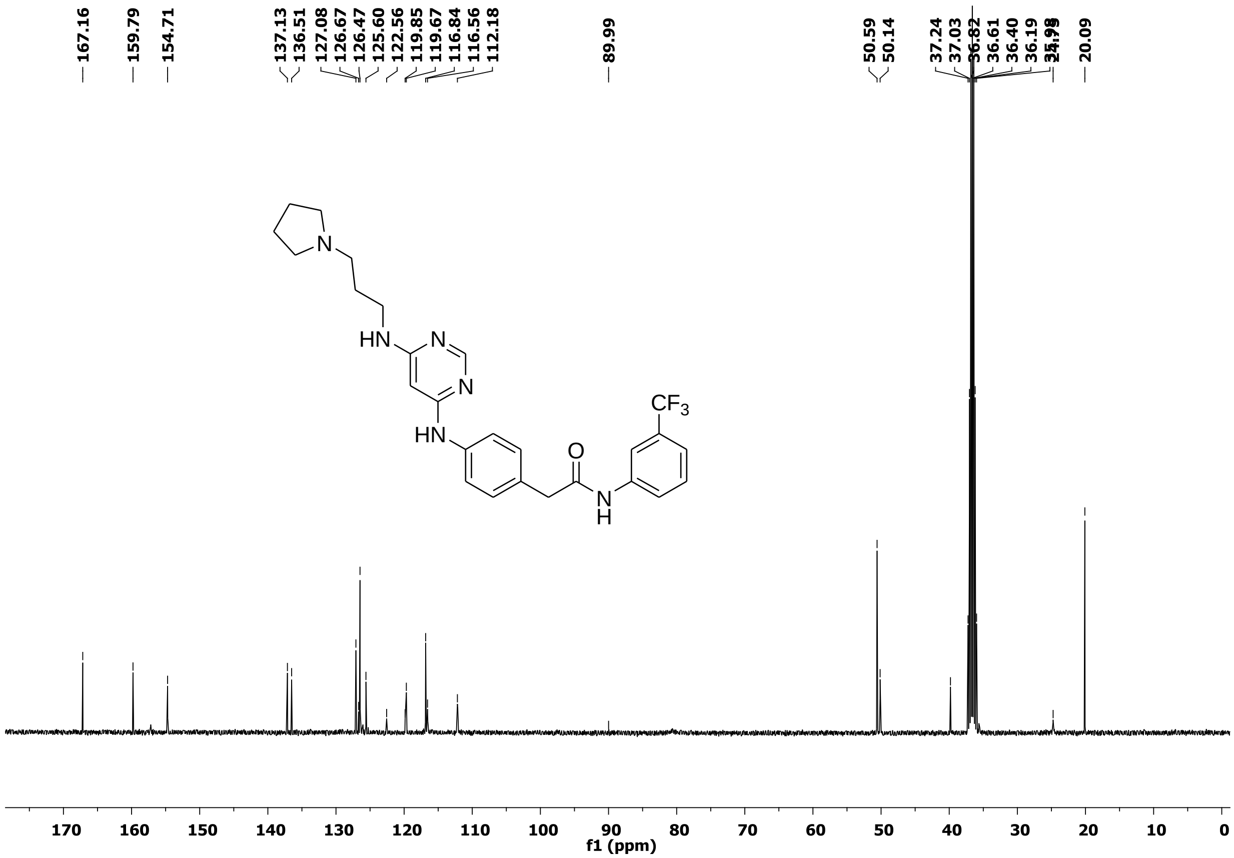
167.16
159.79
154.71
137.13
136.51
127.08
126.67
126.47
125.60
122.56
119.85
119.67
116.84
116.56
112.18
89.99
50.59
50.14
37.24
37.03
36.82
36.61
36.40
36.19
20.09
HN
N
N
HN
N
CF3
O
N
H
170
160
150
140
130
120
110
100
90
80
70
60
50
40
30
20
10
0
f1 (ppm)

S45. ^{1}H, ^{13}C spectrum of 2-(4-(6-(4-(methylsulfonyl)phenylamino)pyrimidin-4-ylamino)phenyl) -N-(3-fluorophenyl)acetamide (13ac)

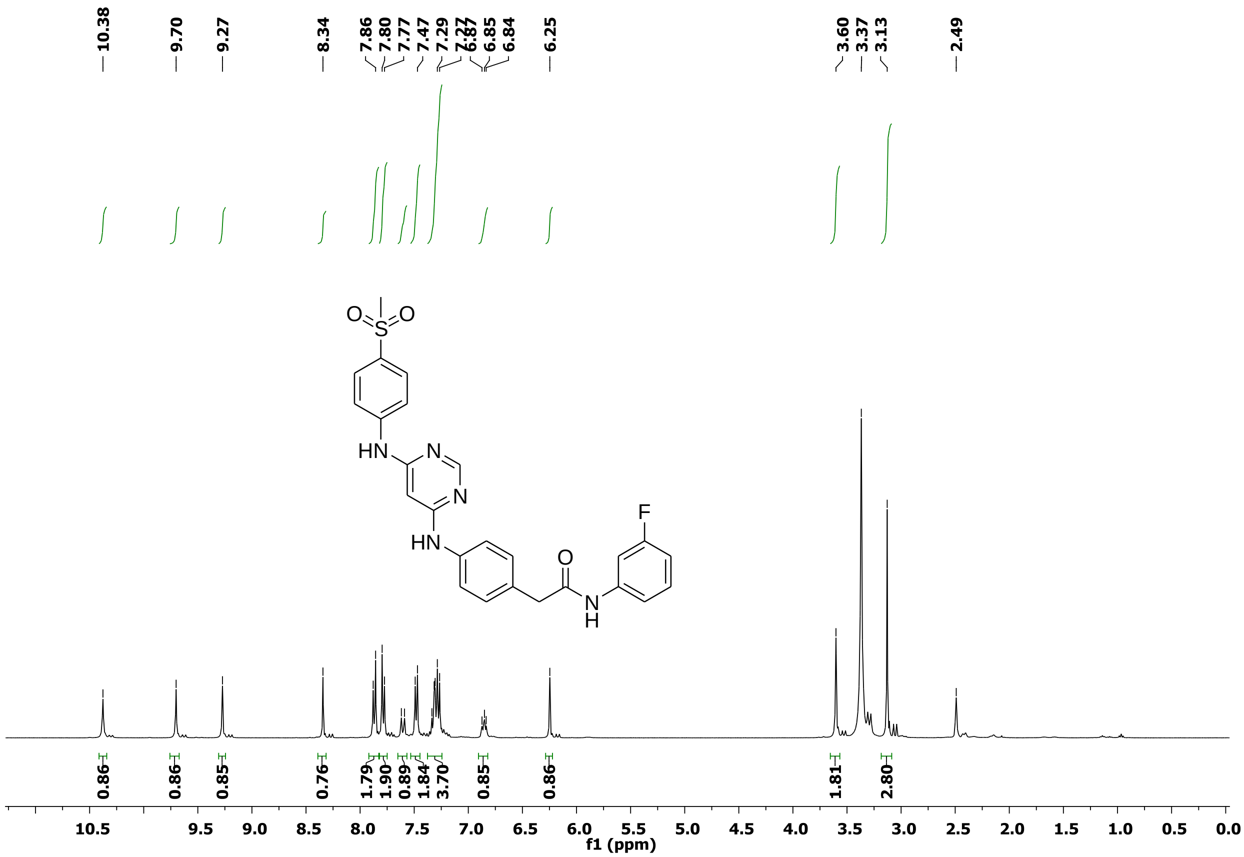

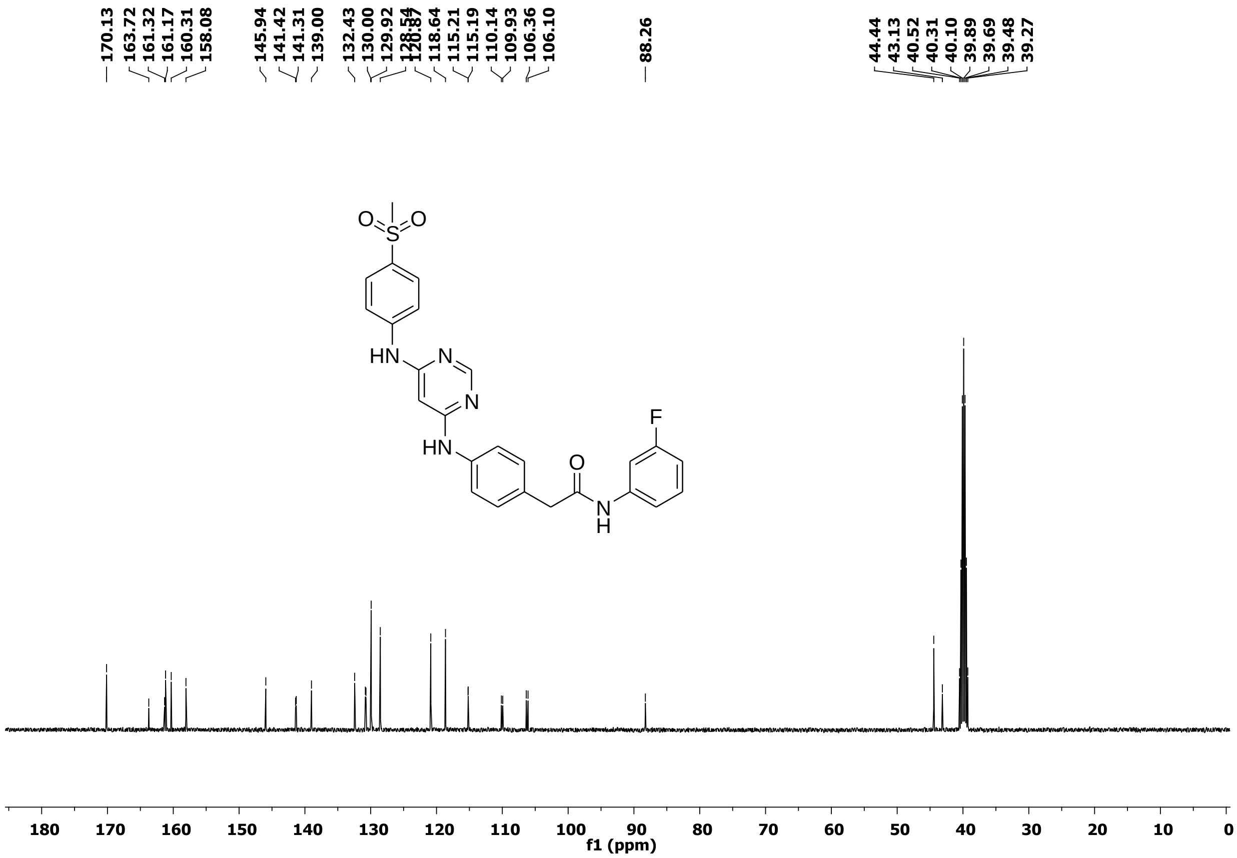
170.13
163.72
161.32
161.17
160.31
158.08
145.94
141.42
141.31
139.00
132.43
130.00
129.92
128.54
120.87
118.64
115.21
115.19
110.14
109.93
106.36
106.10
88.26
44.44
43.13
40.52
40.31
40.10
39.89
39.69
39.48
39.27
180 170 160 150 140 130 120 110 100 90 80 70 60 50 40 30 20 10 0
f1 (ppm)

S46. ^{1}H, ^{13}C spectrum of 2-(4-(6-(methylamino)pyrimidin-4-yloxy)phenyl)-N-(4-fluorophenyl)acetamide (13ad)

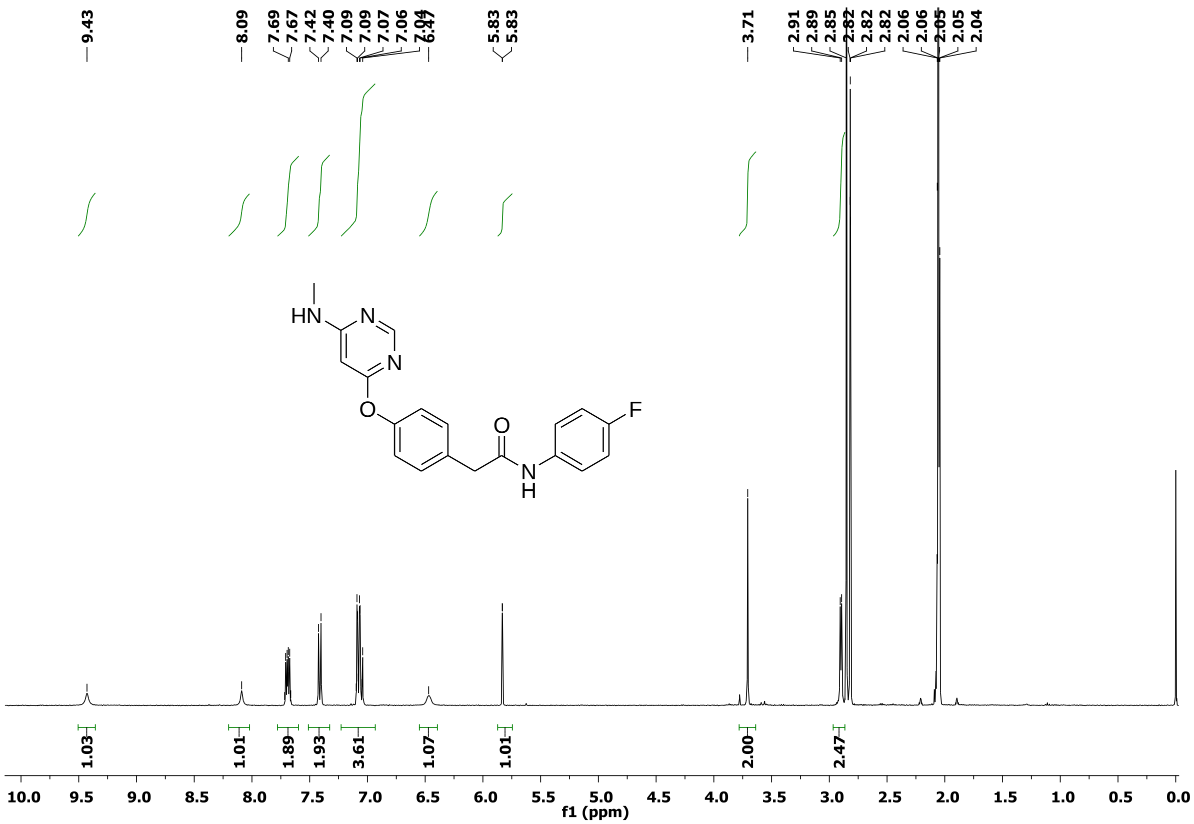

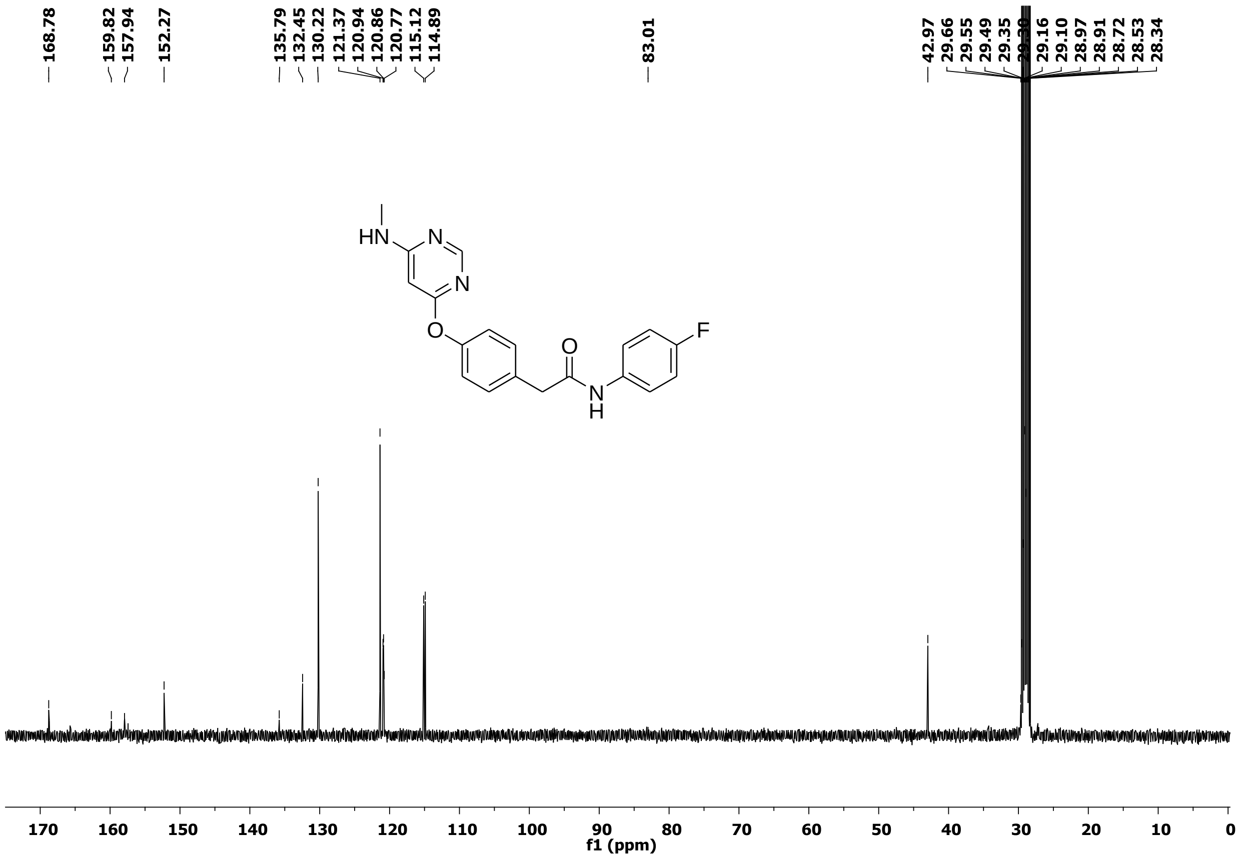
168.78
159.82
157.94
152.27
135.79
132.45
130.22
121.37
120.94
120.86
120.77
115.12
114.89
83.01
42.97
29.66
29.55
29.49
29.35
29.16
29.10
28.97
28.91
28.72
28.53
28.34
HN
N
N
O
O
N
H
F
170
160
150
140
130
120
110
100
90
80
70
60
50
40
30
20
10
0
f1 (ppm)

S47. ^{1}H, ^{13}C spectrum of 2-(4-(6-(4-(methylsulfonyl)phenylamino)pyrimidin-4-ylamino)phenyl)-N-cyclopropylacetamide (13ae)

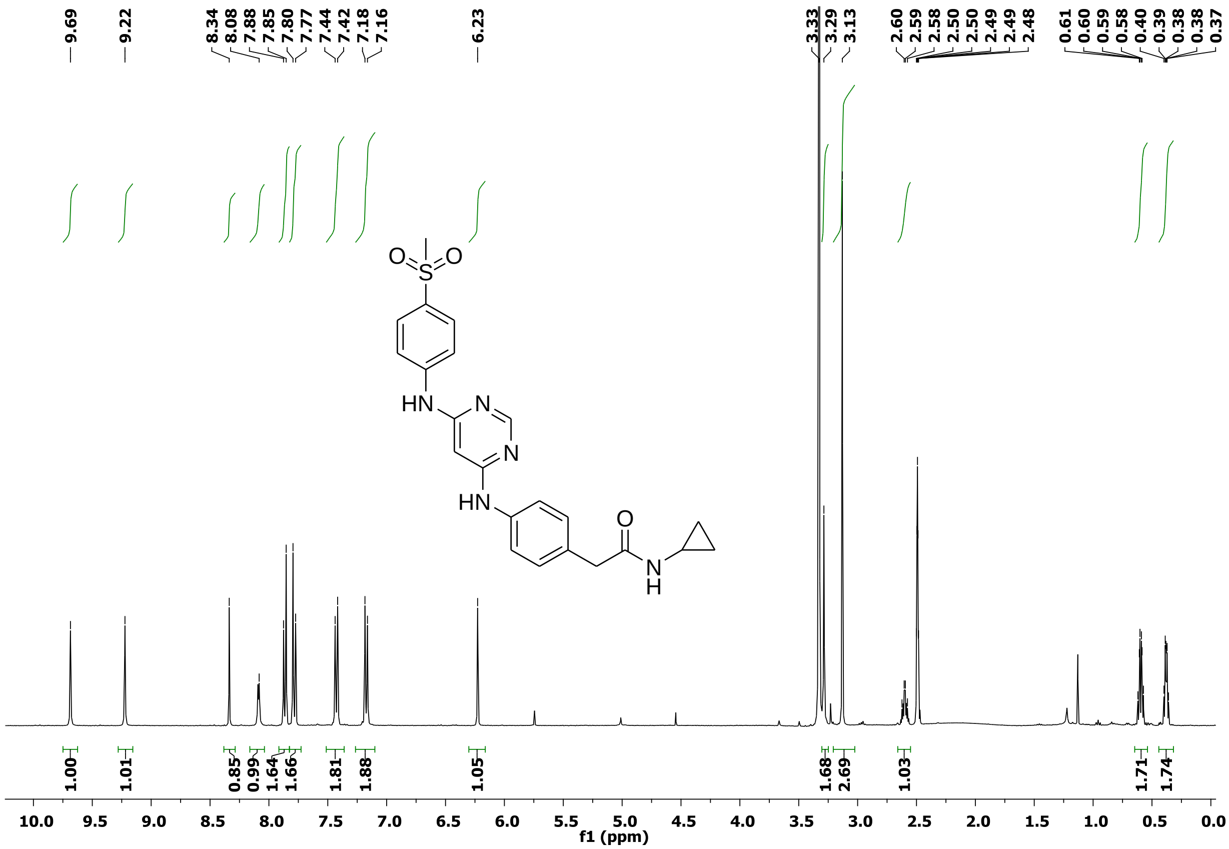

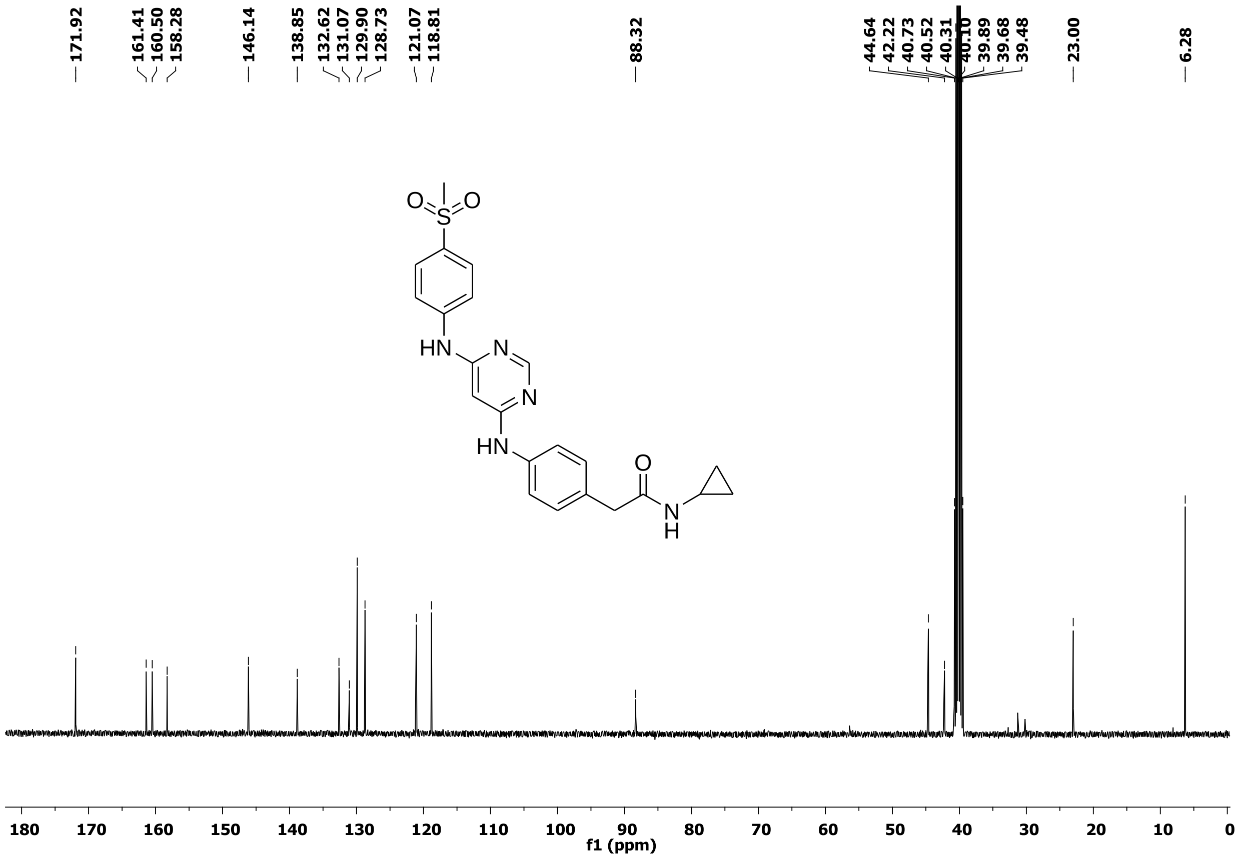
171.92
161.41
160.50
158.28
146.14
138.85
132.62
131.07
129.90
128.73
121.07
118.81
88.32
44.64
42.22
40.73
40.52
40.31
40.10
39.89
39.68
39.48
23.00
6.28
180 170 160 150 140 130 120 110 100 90 80 70 60 50 40 30 20 10 0
f1 (ppm)

S48. ^{1}H, ^{13}C spectrum of 2-(4-(6-(3-(trifluoromethyl)phenylamino)pyrimidin-4-ylamino)phenyl)-N-cyclopropylacetamide (13af)

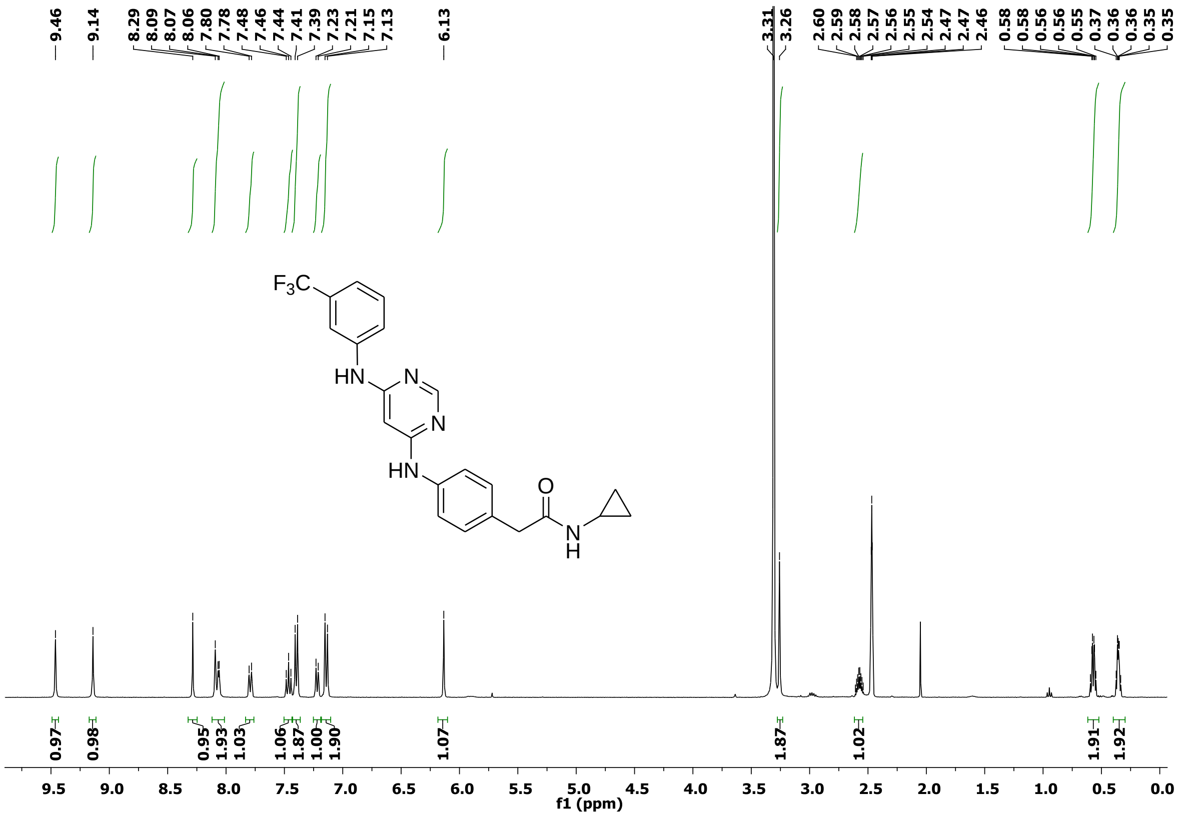

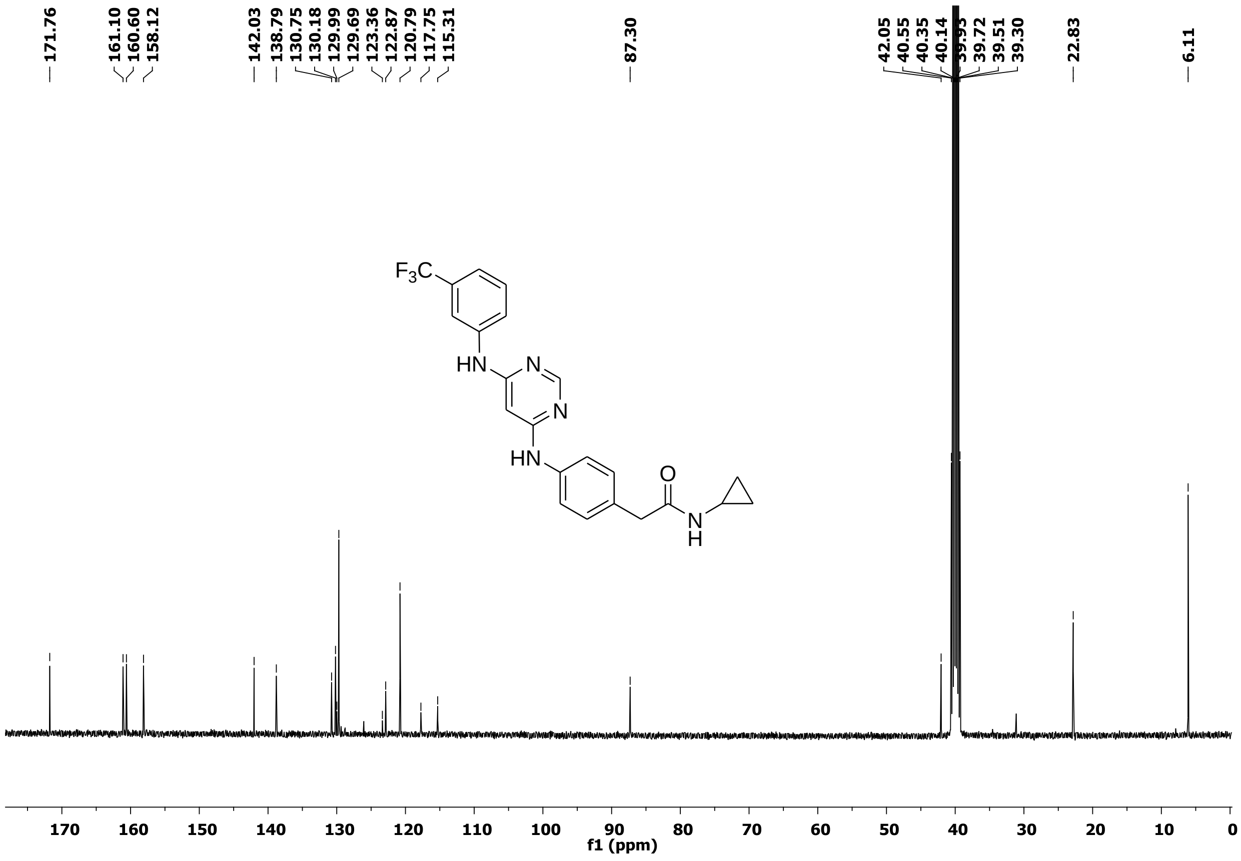

171.76
161.10
160.60
158.12
142.03
138.79
130.75
130.18
129.99
129.69
123.36
122.87
120.79
117.75
115.31
87.30
42.05
40.55
40.35
40.14
39.93
39.72
39.51
39.30
22.83
6.11
F3C
HN
N
N
HN
O
N
H
170
160
150
140
130
120
110
100
90
80
70
60
50
40
30
20
10
0
f1 (ppm)

S49. ^{1}H, ^{13}C spectrum of 2-(4-(6-(2,3-dihydrobenzo[b][1,4]dioxin-5-ylamino)pyrimidin-4-ylamino)phenyl)-N-cyclopropylacetamide (13ag)

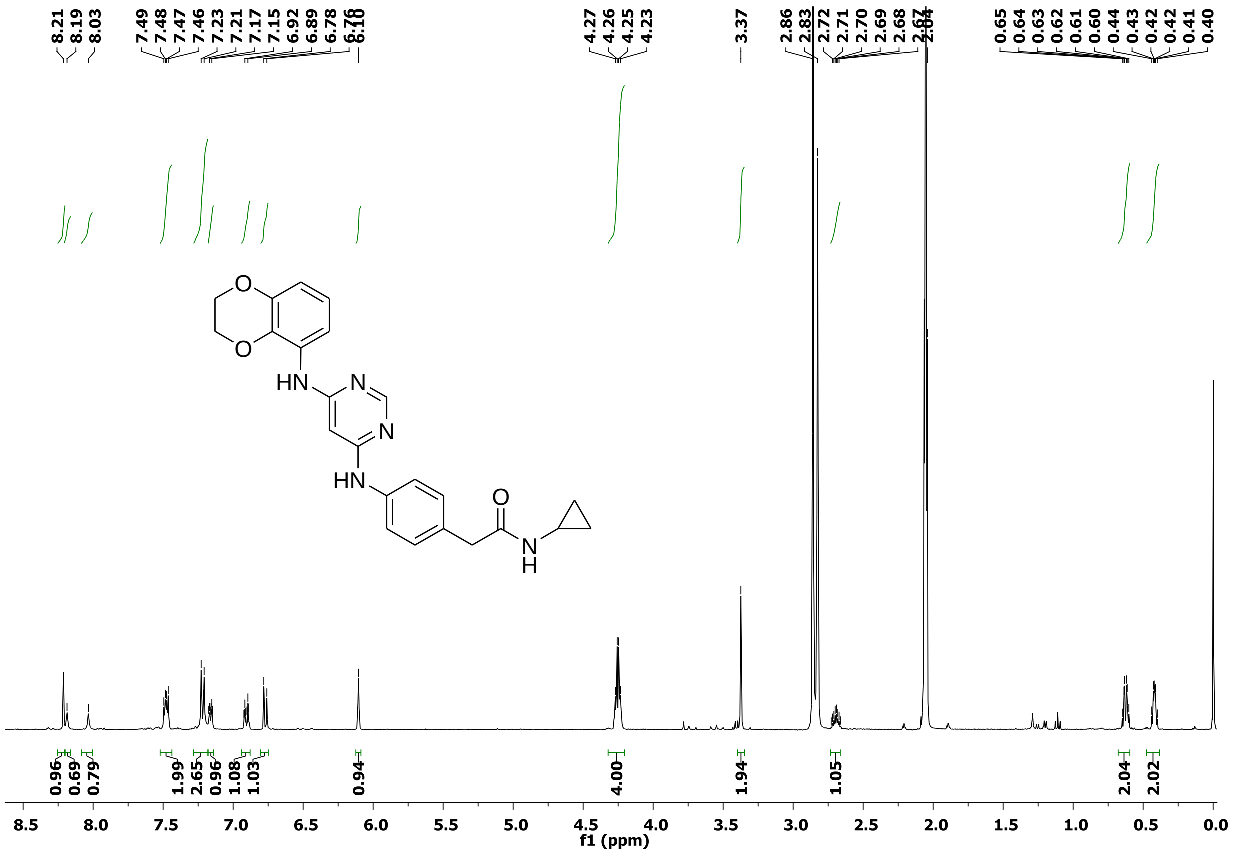

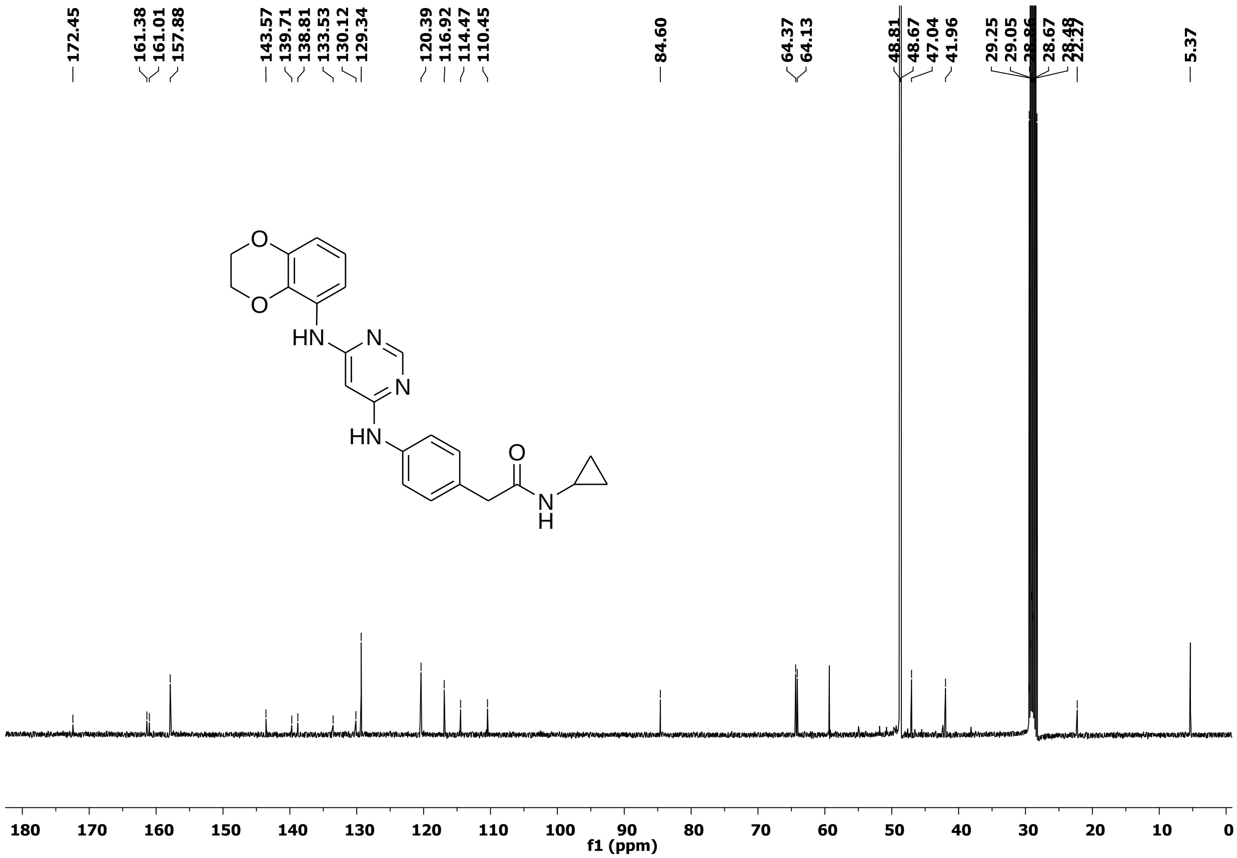
172.45
161.38
161.01
157.88
143.57
139.71
138.81
133.53
130.12
129.34
120.39
116.92
114.47
110.45
84.60
64.37
64.13
48.81
48.67
47.04
41.96
29.25
29.05
28.67
22.27
5.37
O
O
HN
N
N
HN
O
N
H
180
170
160
150
140
130
120
110
100
90
80
70
60
50
40
30
20
10
0
f1 (ppm)

S50. ^{1}H, ^{13}C spectrum of 2-(4-(6-(4-methylpiperazin-1-yl)pyrimidin-4-ylamino)phenyl)-N-Cyclopropylacetamide (13ah)

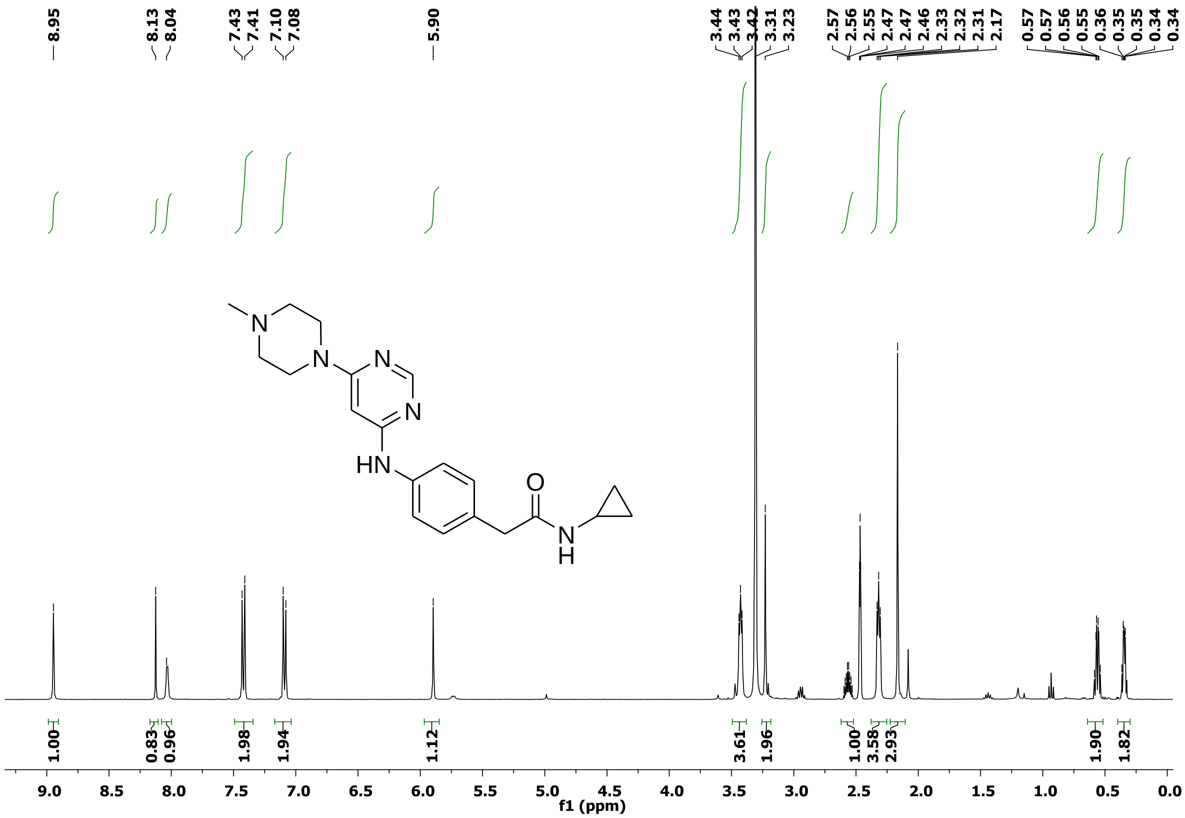

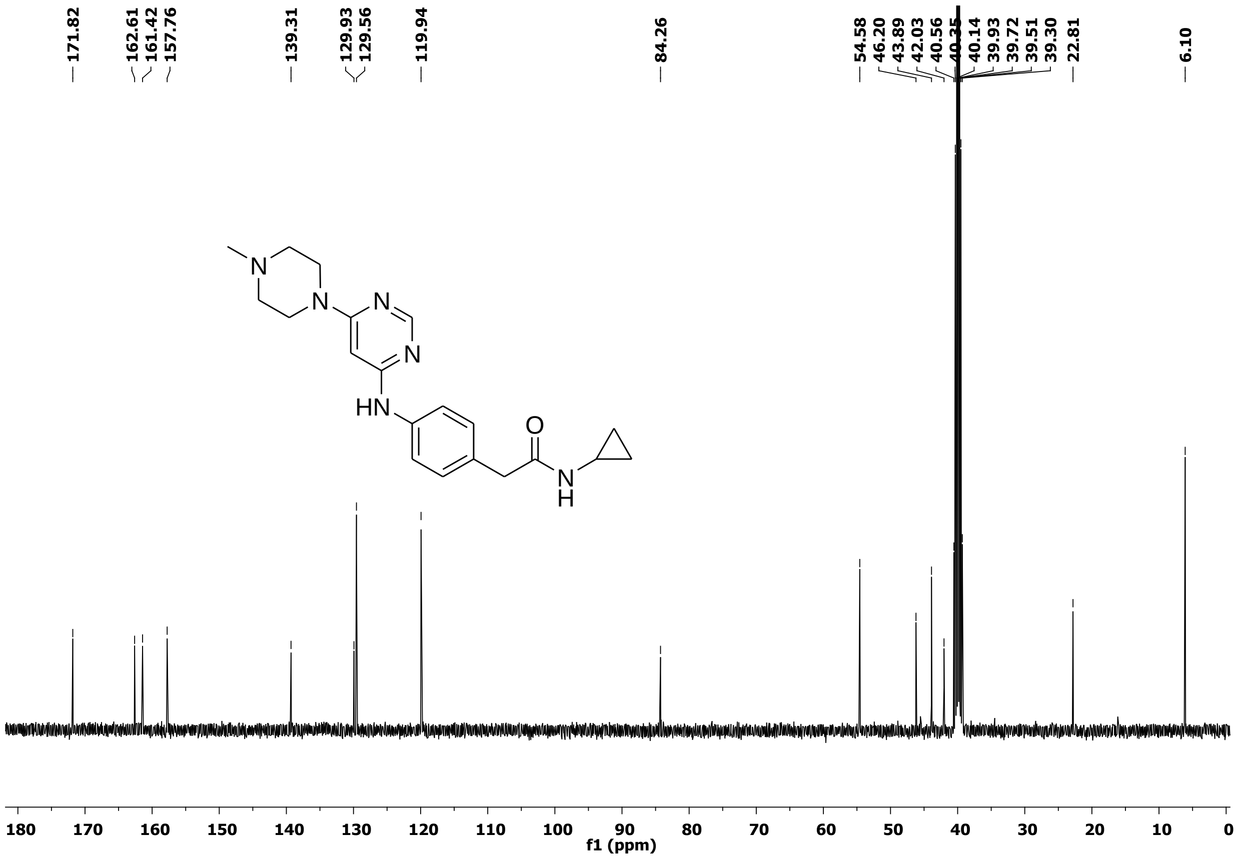

171.82
162.61
161.42
157.76
139.31
129.93
129.56
119.94
84.26
54.58
46.20
43.89
42.03
40.56
40.35
40.14
39.93
39.72
39.51
39.30
22.81
6.10
N
N
N
N
HN
O
N
H
180
170
160
150
140
130
120
110
100
90
80
70
60
50
40
30
20
10
0
f1 (ppm)

S51. ^{1}H, ^{13}C spectrum of 2-(4-(6-morpholinopyrimidin-4-ylamino)phenyl)-N-cyclopropylacetamide (13ai)

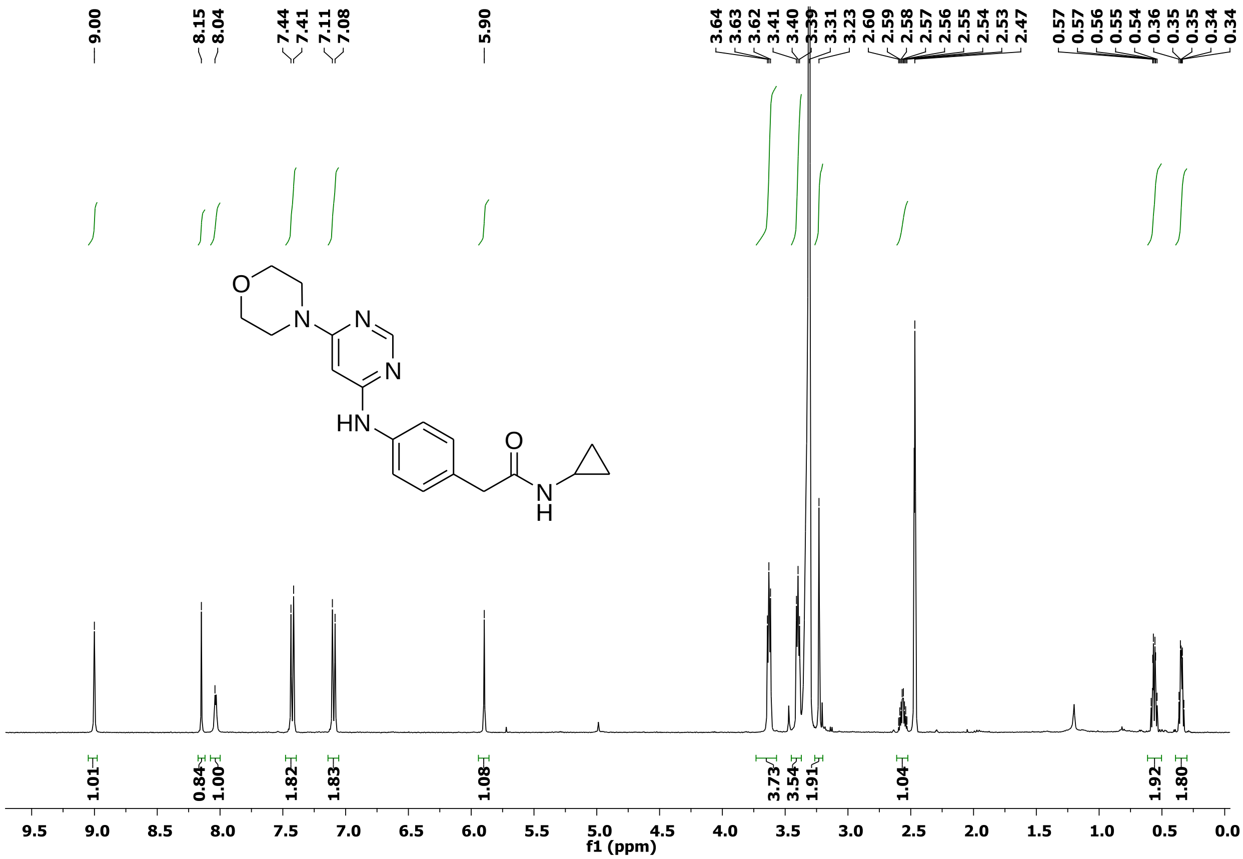

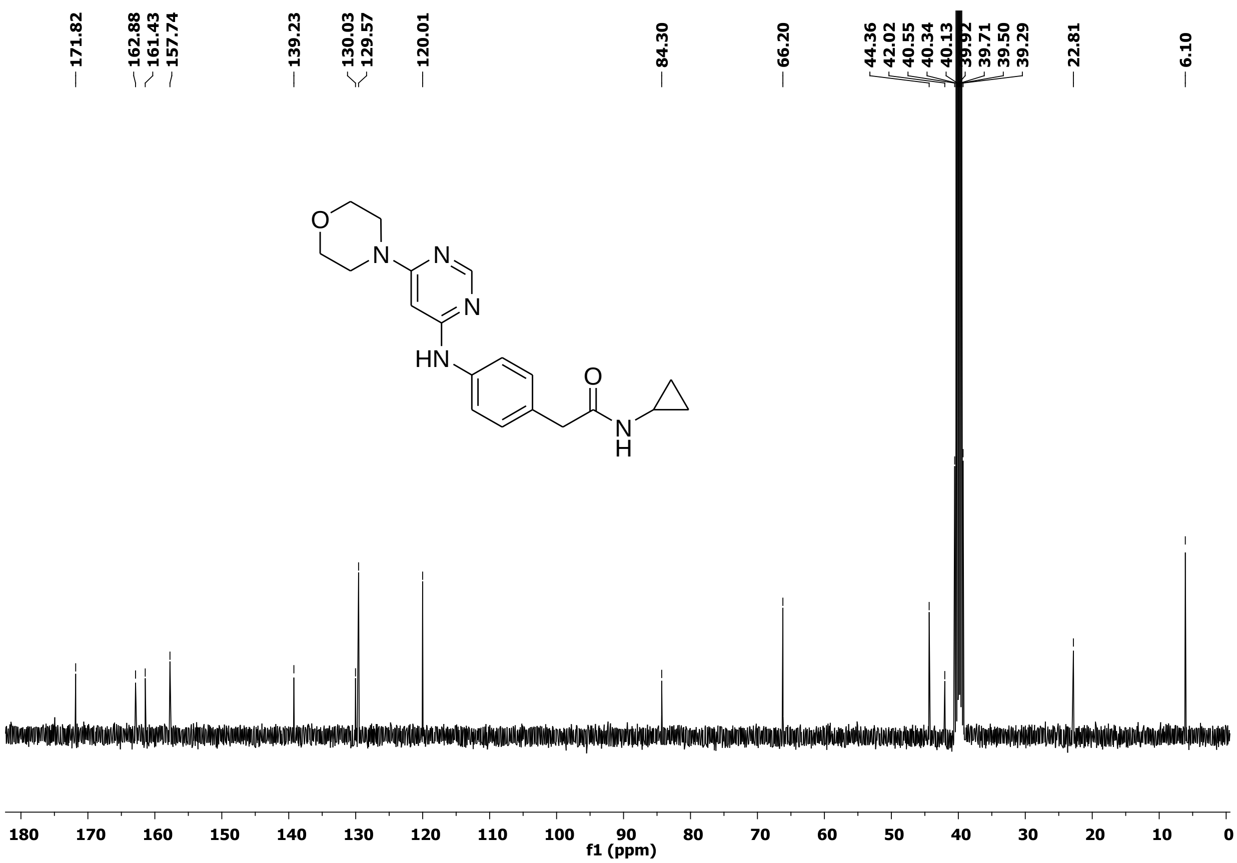
171.82
162.88
161.43
157.74
139.23
130.03
129.57
120.01
84.30
66.20
44.36
42.02
40.55
40.34
40.13
39.92
39.71
39.50
39.29
22.81
6.10
O
N
N
N
HN
O
N
H
180 170 160 150 140 130 120 110 100 90 80 70 60 50 40 30 20 10 0
f1 (ppm)

S52. ^{1}H, ^{13}C spectrum of 2-(4-(6-(butylamino)pyrimidin-4-yloxy)phenyl)-N-(4-tert-butylthiazol-2-yl)acetamide (13aj)

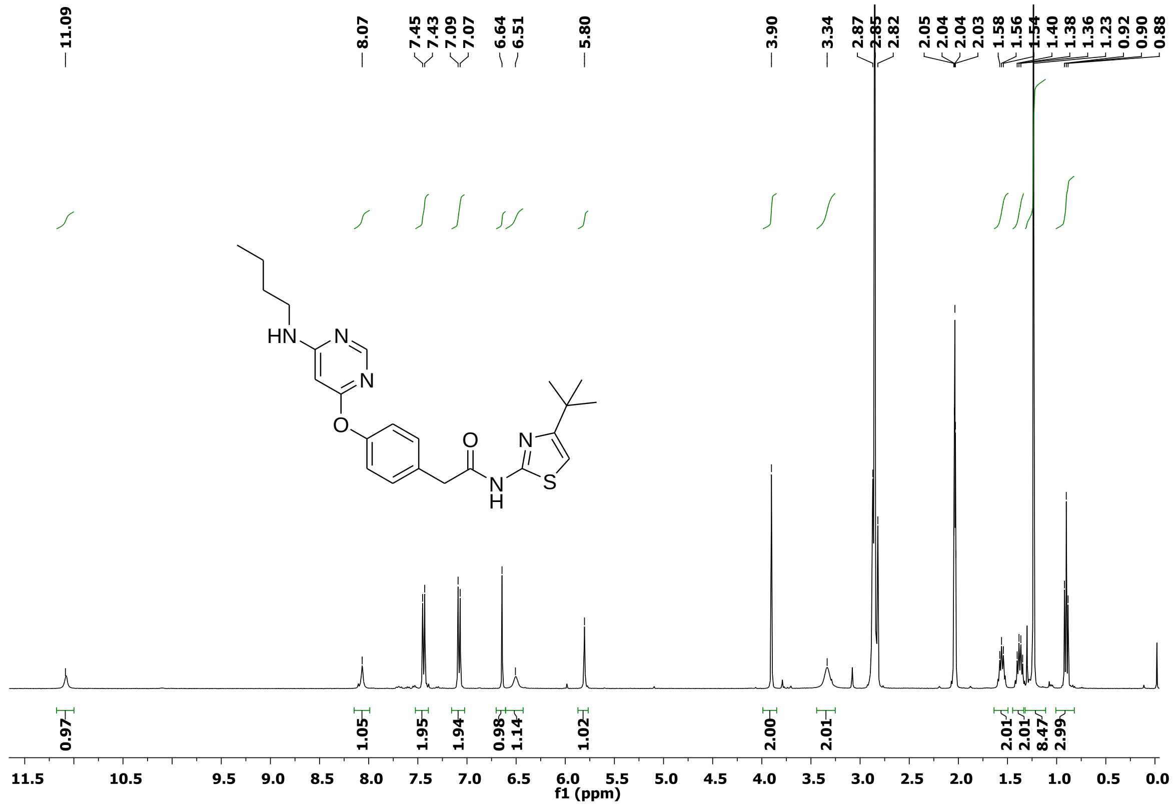

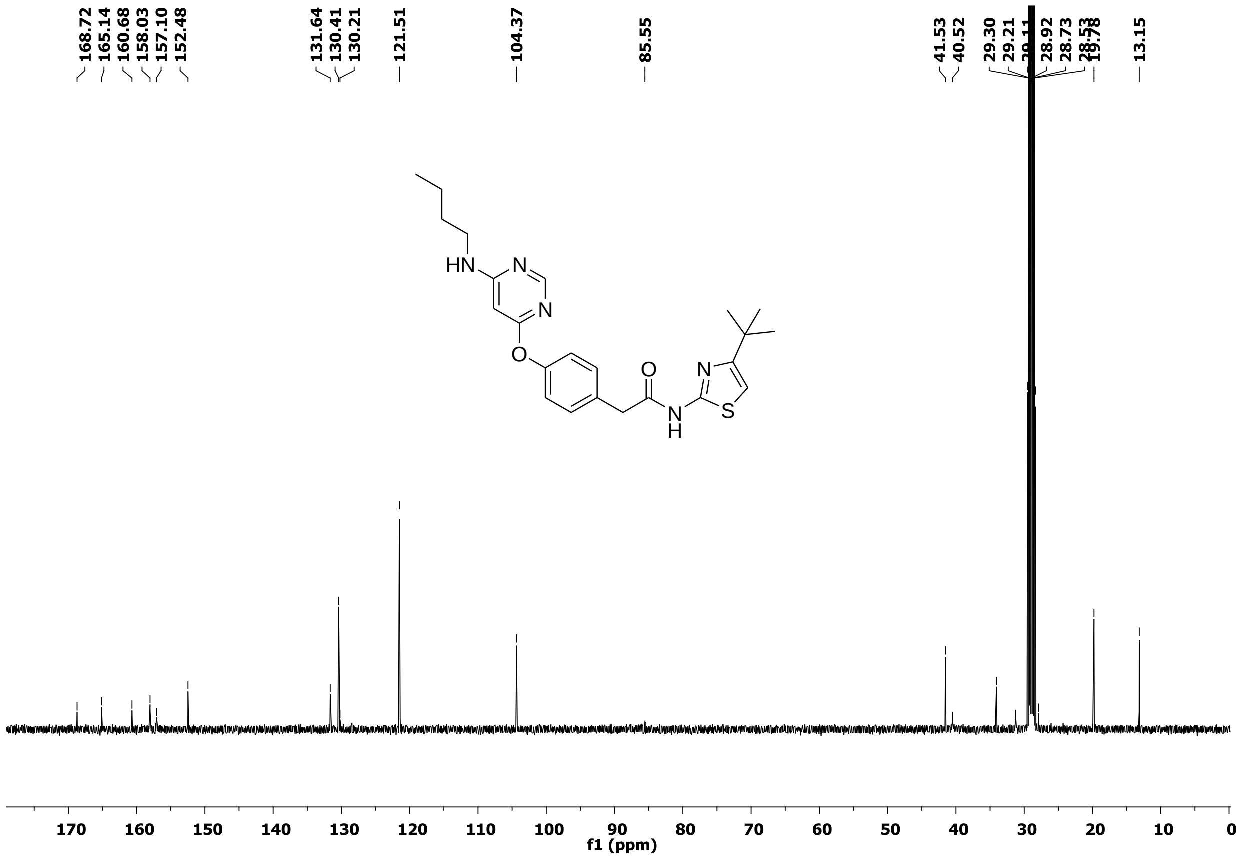
168.72
165.14
160.68
158.03
157.10
152.48
131.64
130.41
130.21
121.51
104.37
85.55
41.53
40.52
29.30
29.21
28.92
28.73
19.78
13.15
HN
N
N
O
O
N
S
N
H
170 160 150 140 130 120 110 100 90 80 70 60 50 40 30 20 10 0
f1 (ppm)

S53. ^{1}H, ^{13}C spectrum of N-(2,4-dimethoxybenzyl)-2-(4-(6-(1-benzylpiperidin-4-ylamino) pyrimidin-4-yloxy)phenyl)acetamide (13ak)

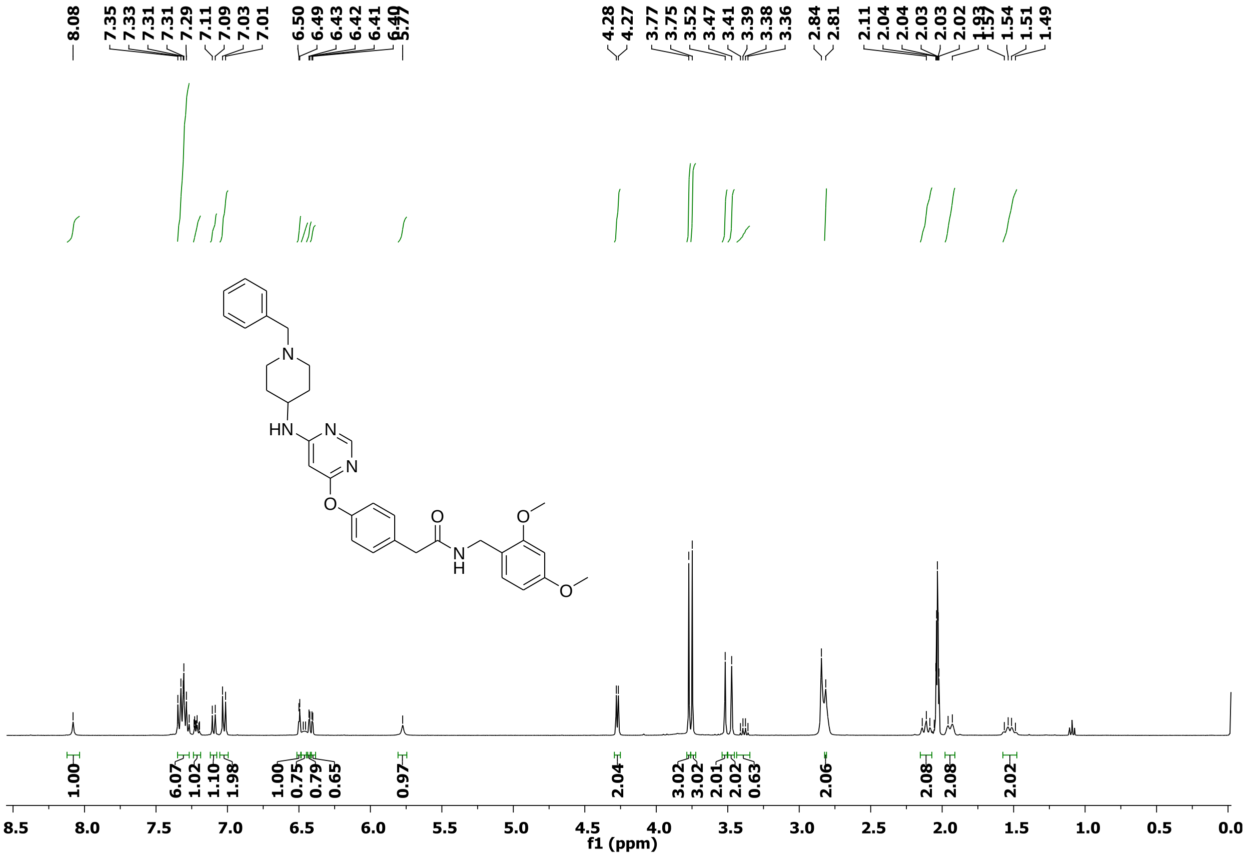

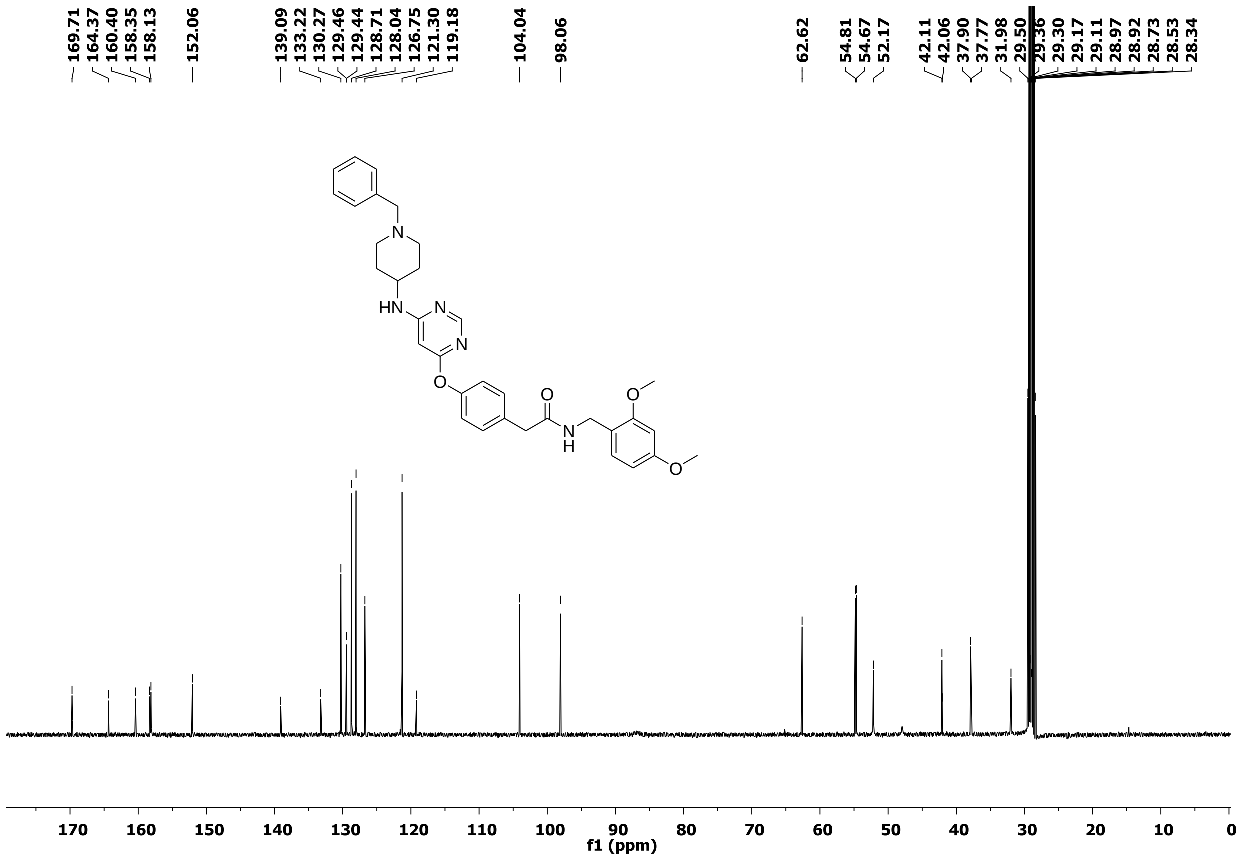
169.71
164.37
160.40
158.35
158.13
152.06
139.09
133.22
130.27
129.46
129.44
128.71
128.04
126.75
121.30
119.18
104.04
98.06
62.62
54.81
54.67
52.17
42.11
42.06
37.90
37.77
31.98
29.50
29.36
29.30
29.17
29.11
28.97
28.92
28.73
28.53
28.34
HN
N
N
N
O
O
N
H
O
O
f1 (ppm)

S54. ^{1}H, ^{13}C spectrum of 2-(4-(6-(4-(methylsulfonyl)phenylamino)pyridin-2-ylamino)phenyl)-N- (3-tert-butyl-1-methyl-1H-pyrazol-5-yl)acetamide (18)

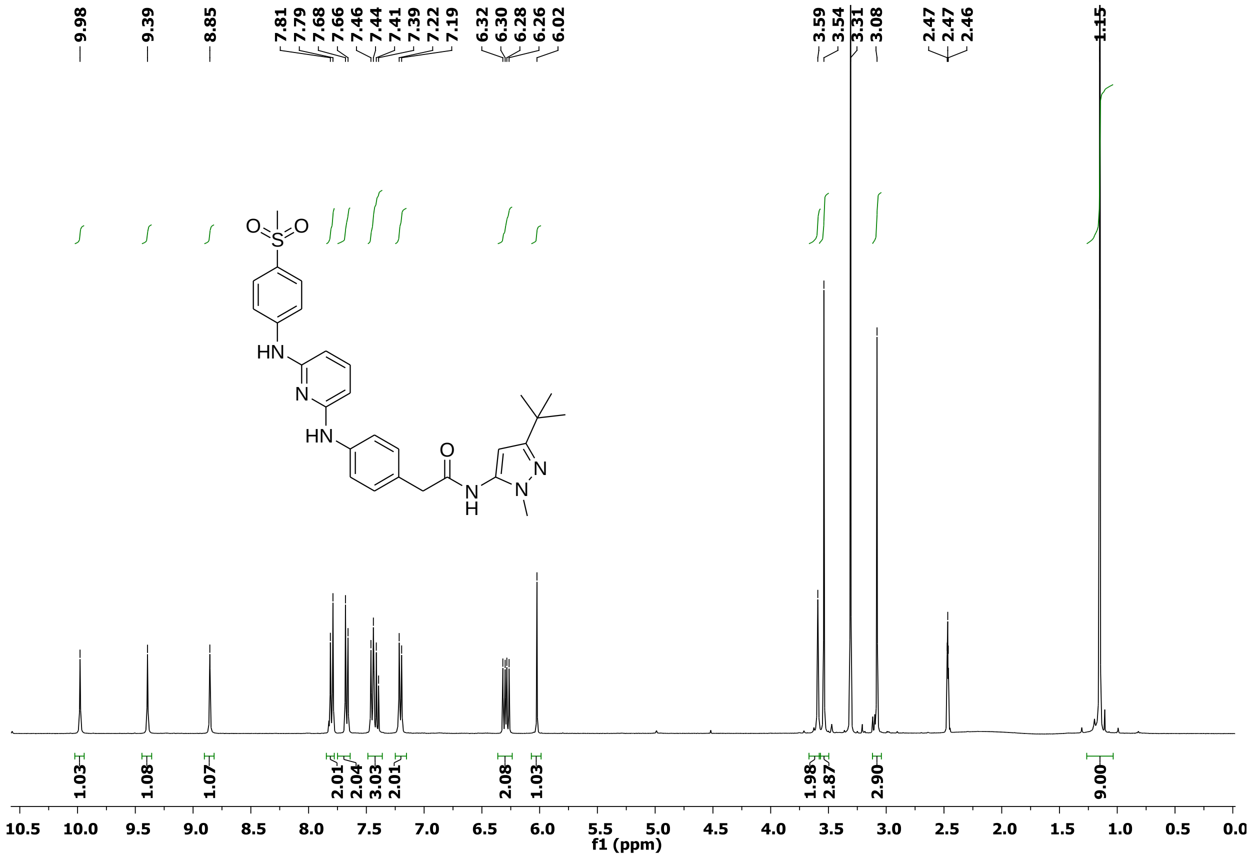

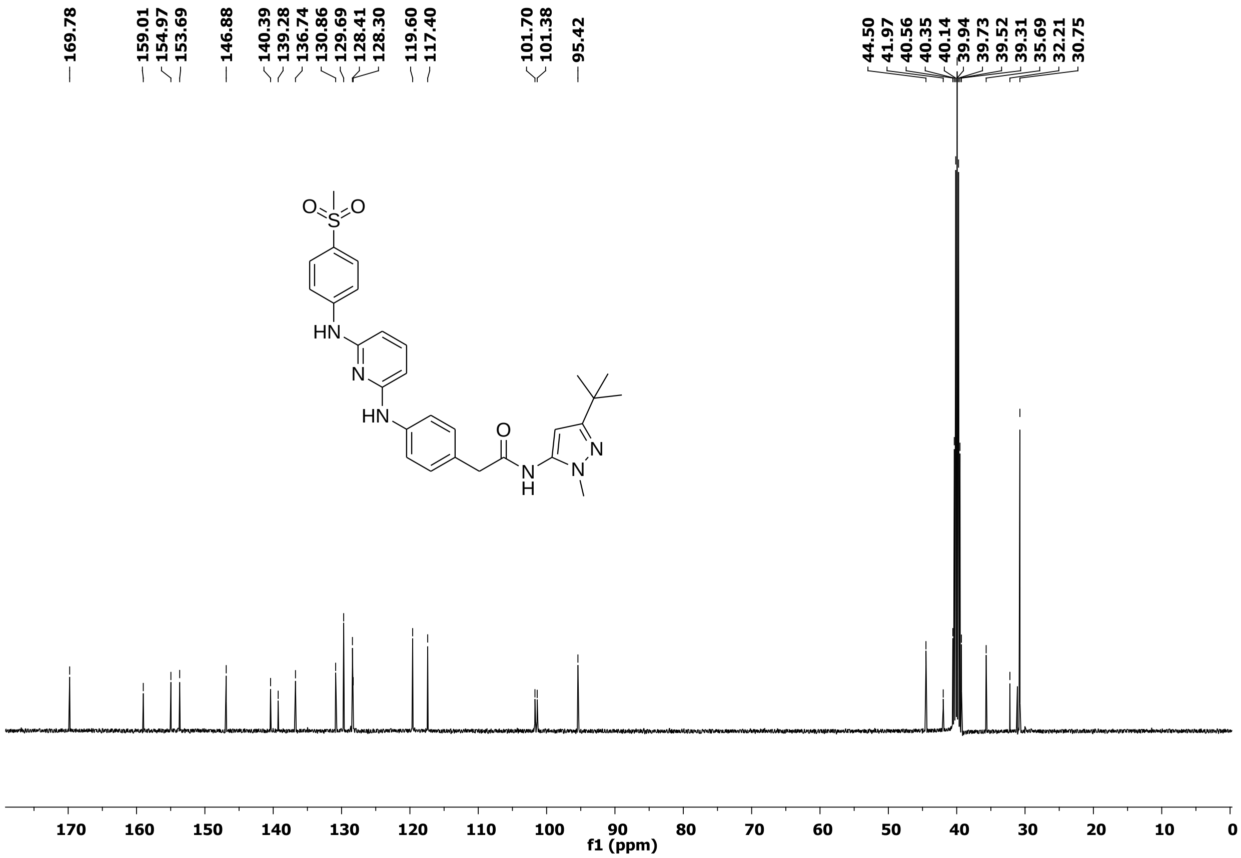
169.78
159.01
154.97
153.69
146.88
140.39
139.28
136.74
130.86
129.69
128.41
128.30
119.60
117.40
101.70
101.38
95.42
44.50
41.97
40.56
40.35
40.14
39.94
39.73
39.52
39.31
35.69
32.21
30.75
170
160
150
140
130
120
110
100
90
80
70
60
50
40
30
20
10
0
f1 (ppm)

S55. ^{1}H, ^{13}C spectrum of Synthesis of tert-butyl 4-(6-chloropyrimidin-4-ylamino)phenylcarbamate (20)

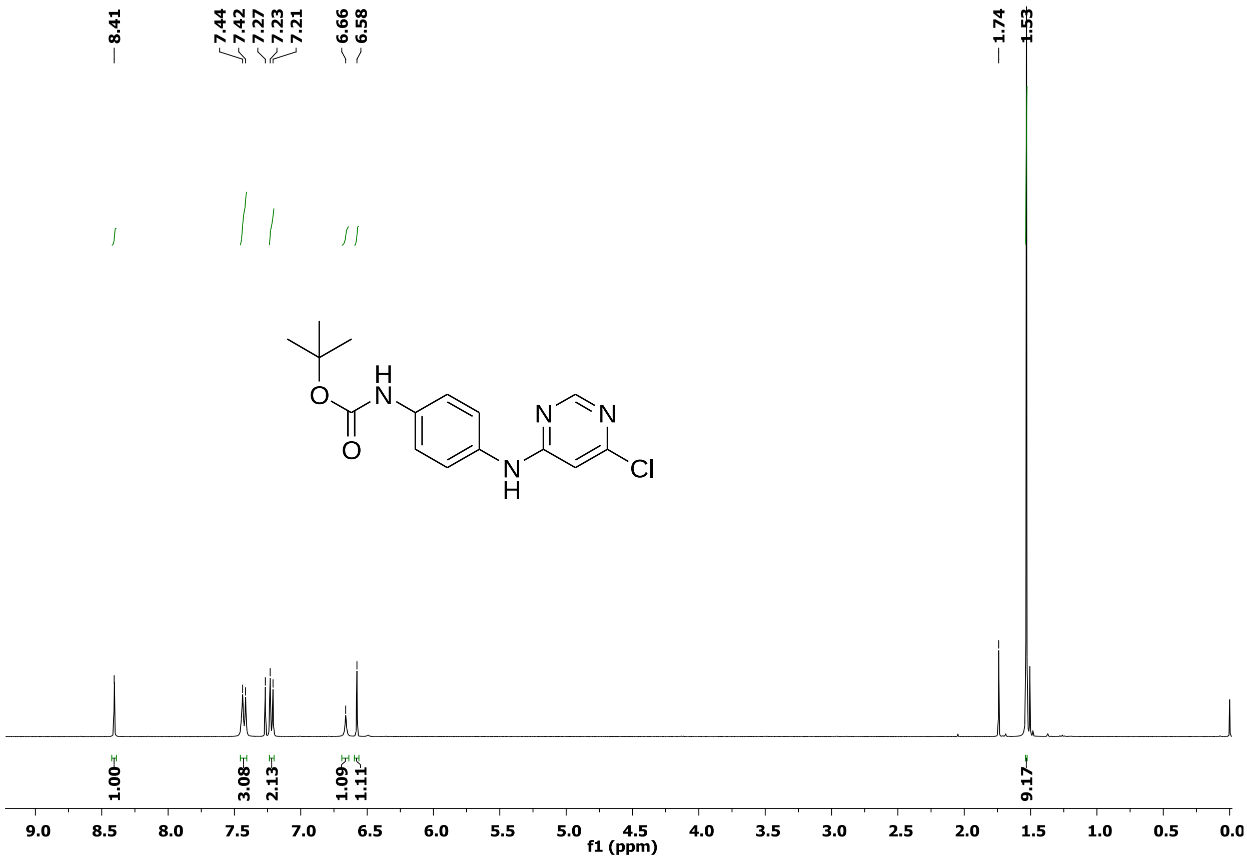

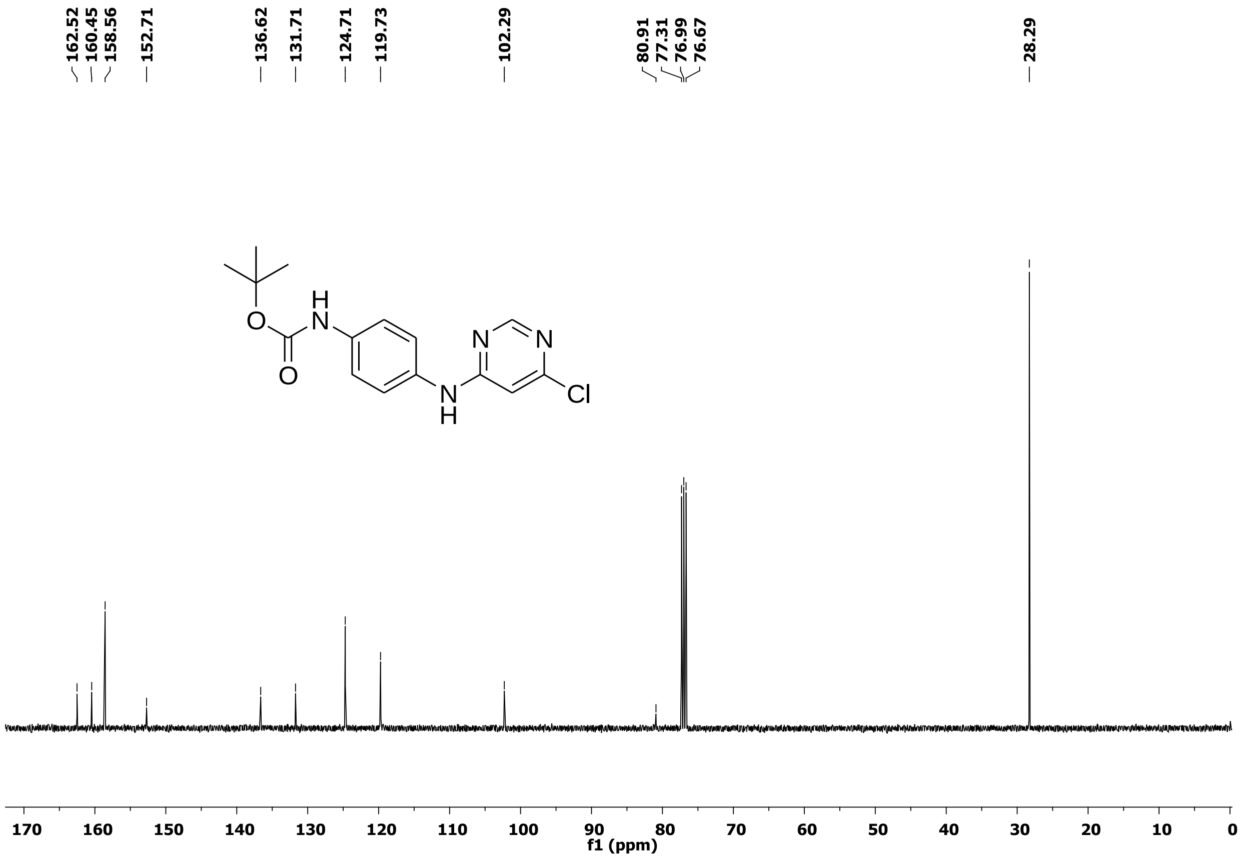
162.52
160.45
158.56
152.71
136.62
131.71
124.71
119.73
102.29
80.91
77.31
76.99
76.67
28.29
H
N
O
O
N
N
N
H
Cl
170 160 150 140 130 120 110 100 90 80 70 60 50 40 30 20 10 0
f1 (ppm)

S56. ^{1}H, ^{13}C spectrum of Synthesis of tert-butyl 4-(6-(4 (methylsulfonyl)phenylamino)pyrimidin-4-ylamino)phenylcarbamate (21)

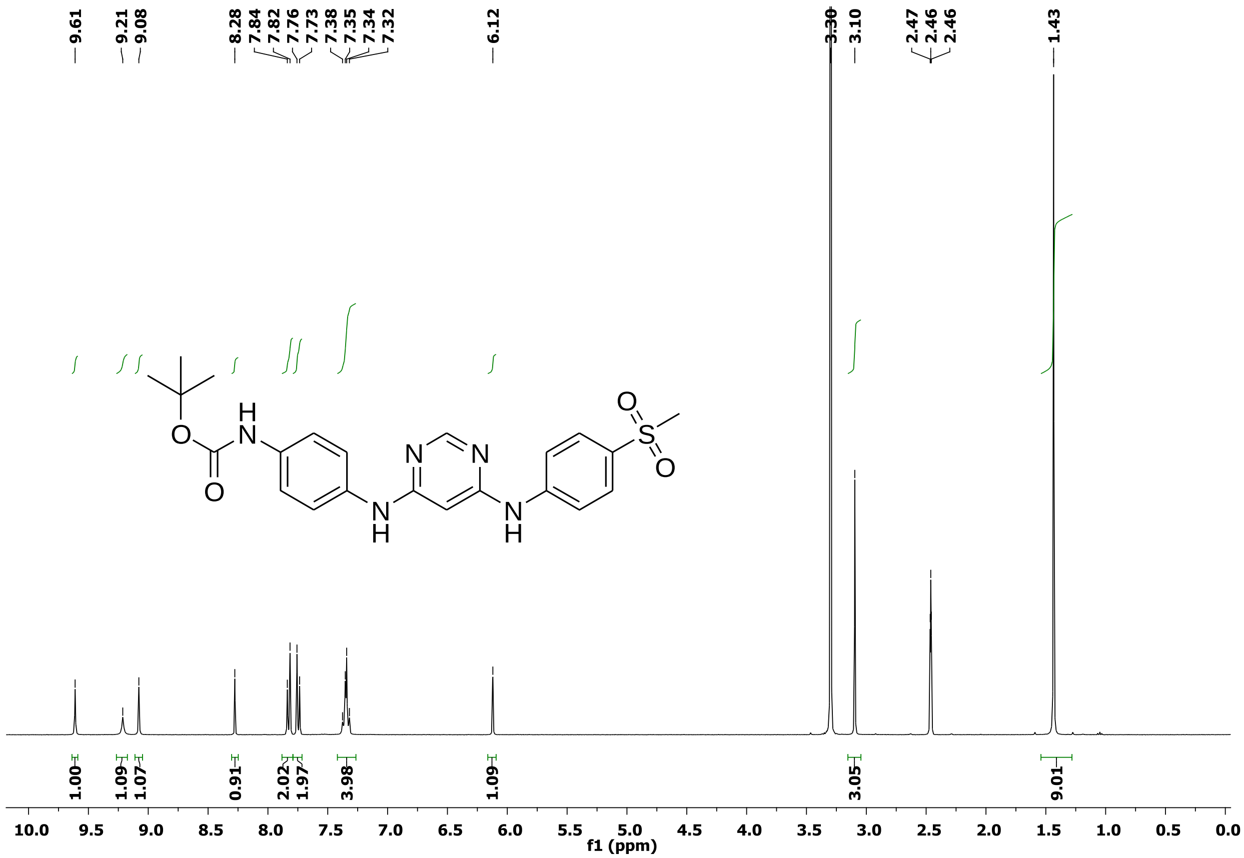

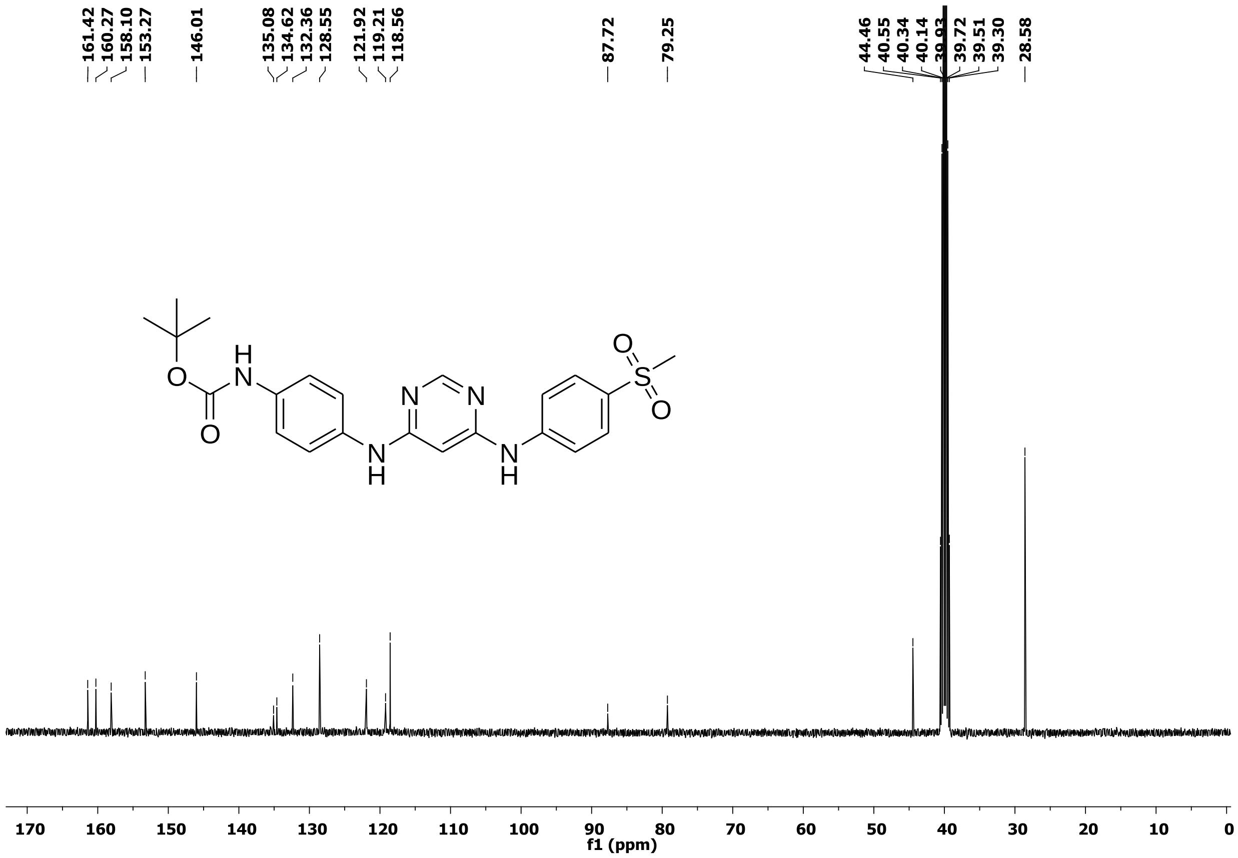
161.42
160.27
158.10
153.27
146.01
135.08
134.62
132.36
128.55
121.92
119.21
118.56
87.72
79.25
44.46
40.55
40.34
40.14
39.93
39.72
39.51
39.30
28.58
H
N
O
O
N
N
N
H
N
H
O
S
O
170
160
150
140
130
120
110
100
90
80
70
60
50
40
30
20
10
0
f1 (ppm)

S57. ^{1}H, ^{13}C spectrum of Synthesis of N4-(4-aminophenyl)-N6-(4-(methylsulfonyl)phenyl)pyrimidine-4,6-diamine (22)

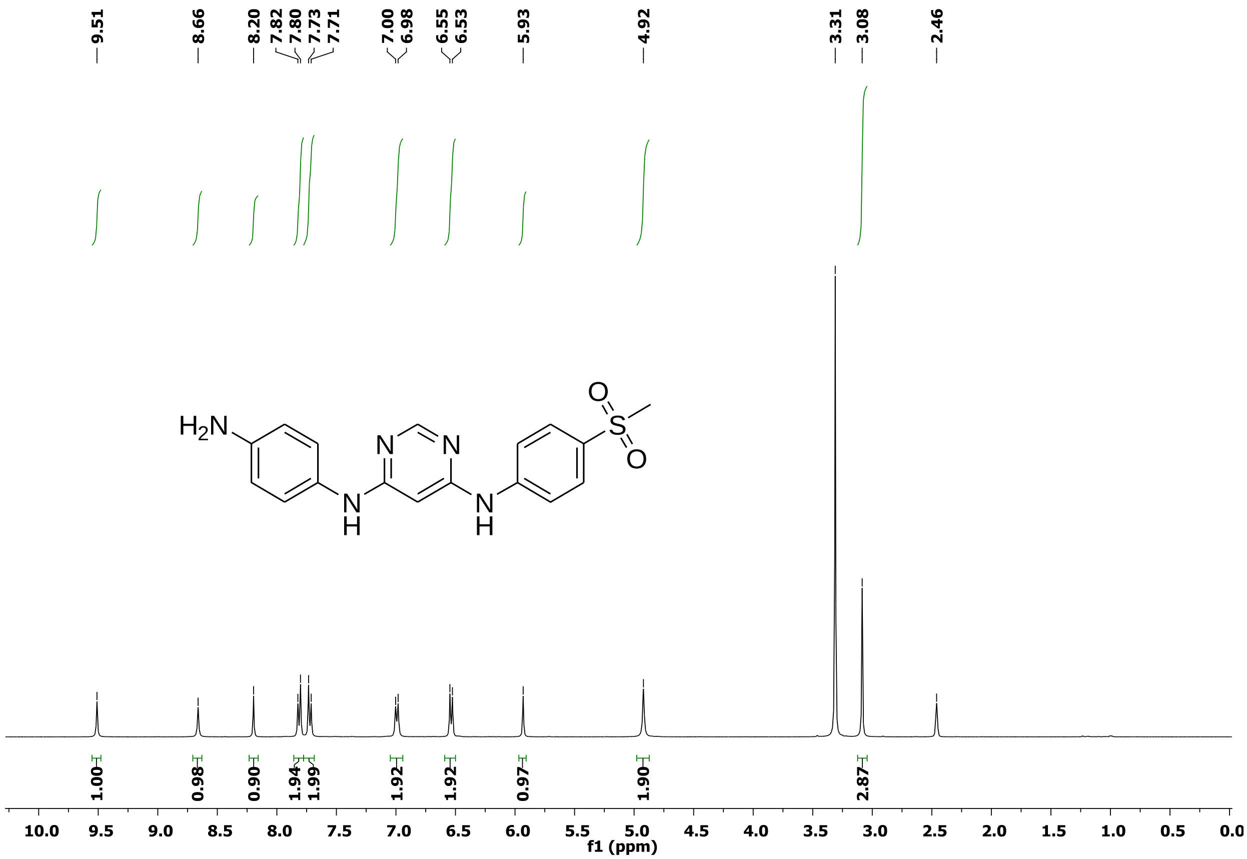

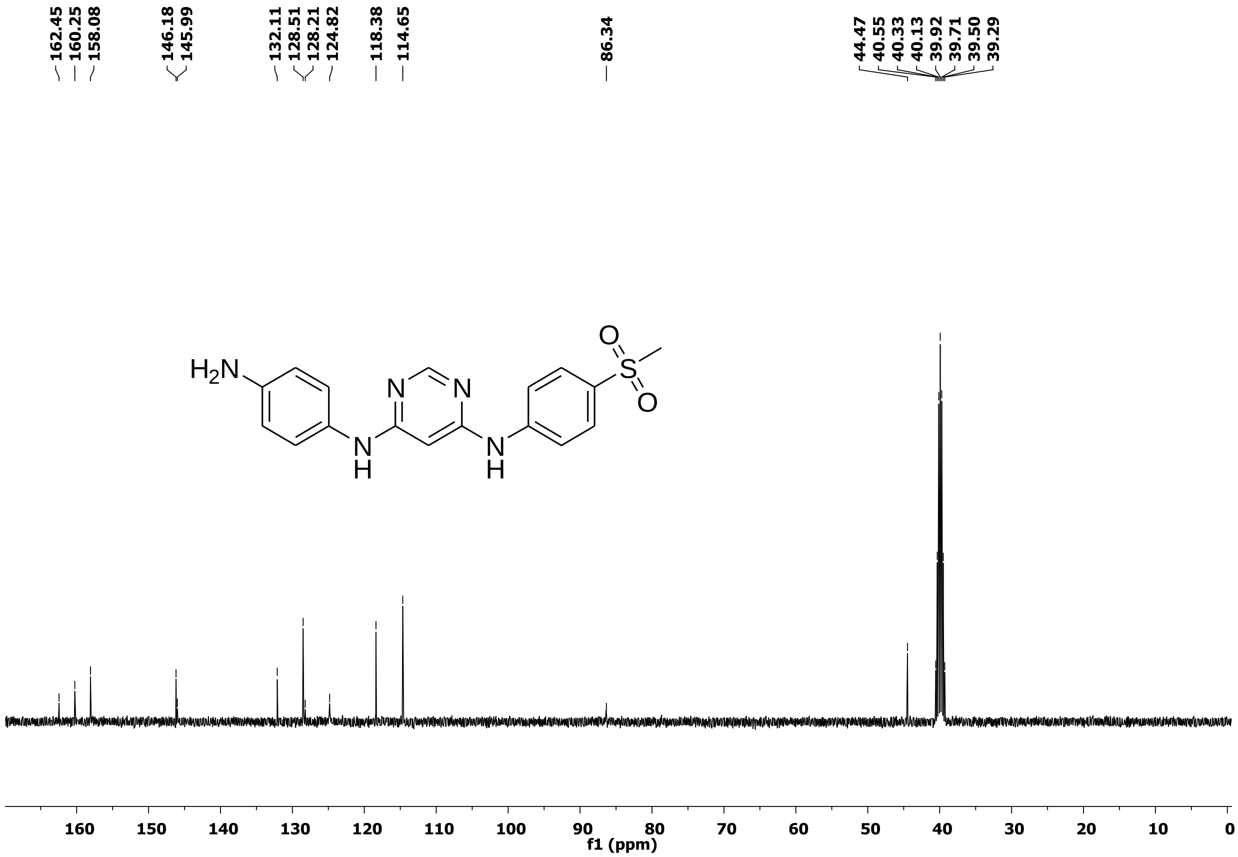
162.45
160.25
158.08
146.18
145.99
132.11
128.51
128.21
124.82
118.38
114.65
86.34
44.47
40.55
40.33
40.13
39.92
39.71
39.50
39.29
H_2N
N
N
N
H
N
H
S
O
O
160
150
140
130
120
110
100
90
80
70
60
50
40
30
20
10
0
f1 (ppm)

S58. ^{1}H, ^{13}C spectrum of 1-(4-(6-(4-(methylsulfonyl)phenylamino)pyrimidin-4-ylamino)phenyl)-3-(3-tert-butyl-1-methyl-1H-pyrazol-5-yl)urea (24a)

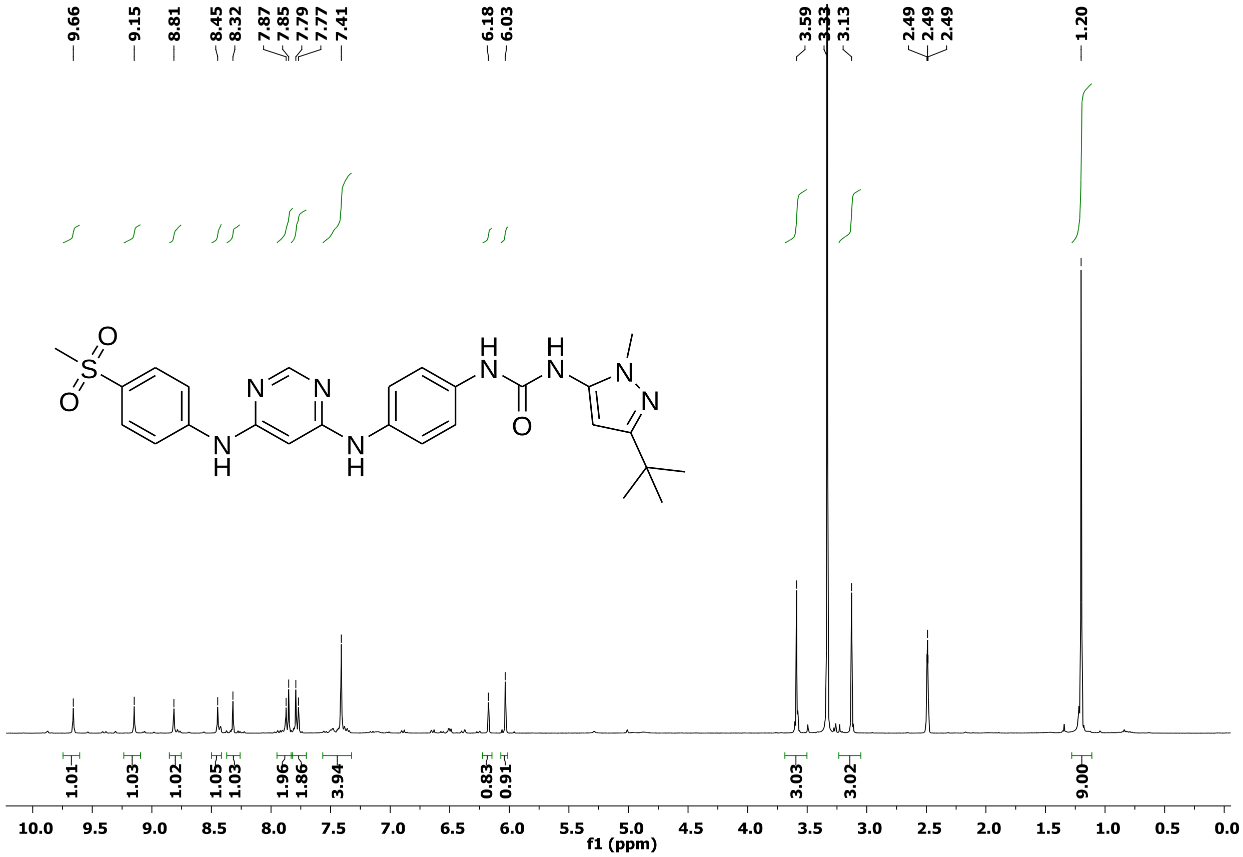

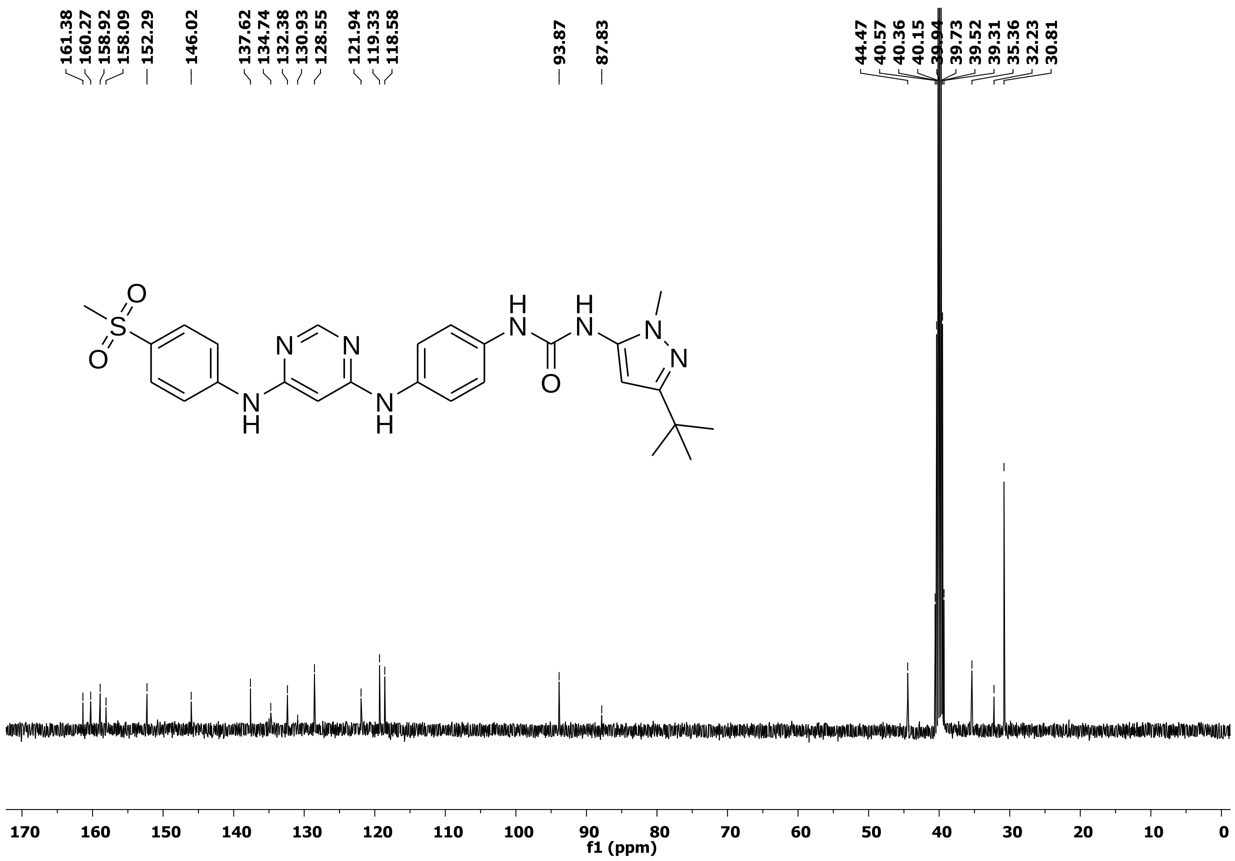
161.38
160.27
158.92
158.09
152.29
146.02
137.62
134.74
132.38
130.93
128.55
121.94
119.33
118.58
93.87
87.83
44.47
40.57
40.36
40.15
39.94
39.73
39.52
39.31
35.36
32.23
30.81
f1 (ppm)

S59. 1H, 13C spectrum of 1-(4-(6-(methylamino)pyrimidin-4-ylamino)phenyl)-3-(3-tert-butyl-1-methyl-1H-pyrazol-5-yl)urea (24b)

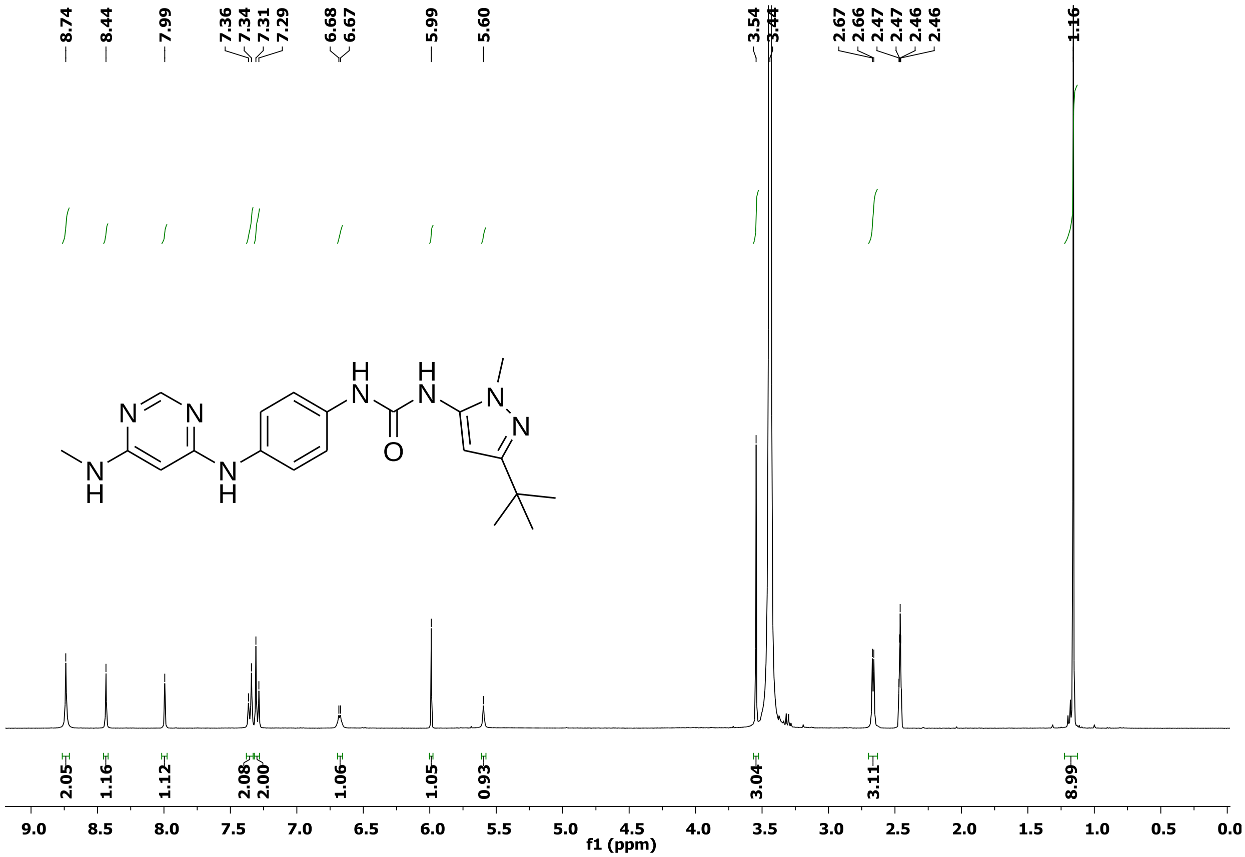

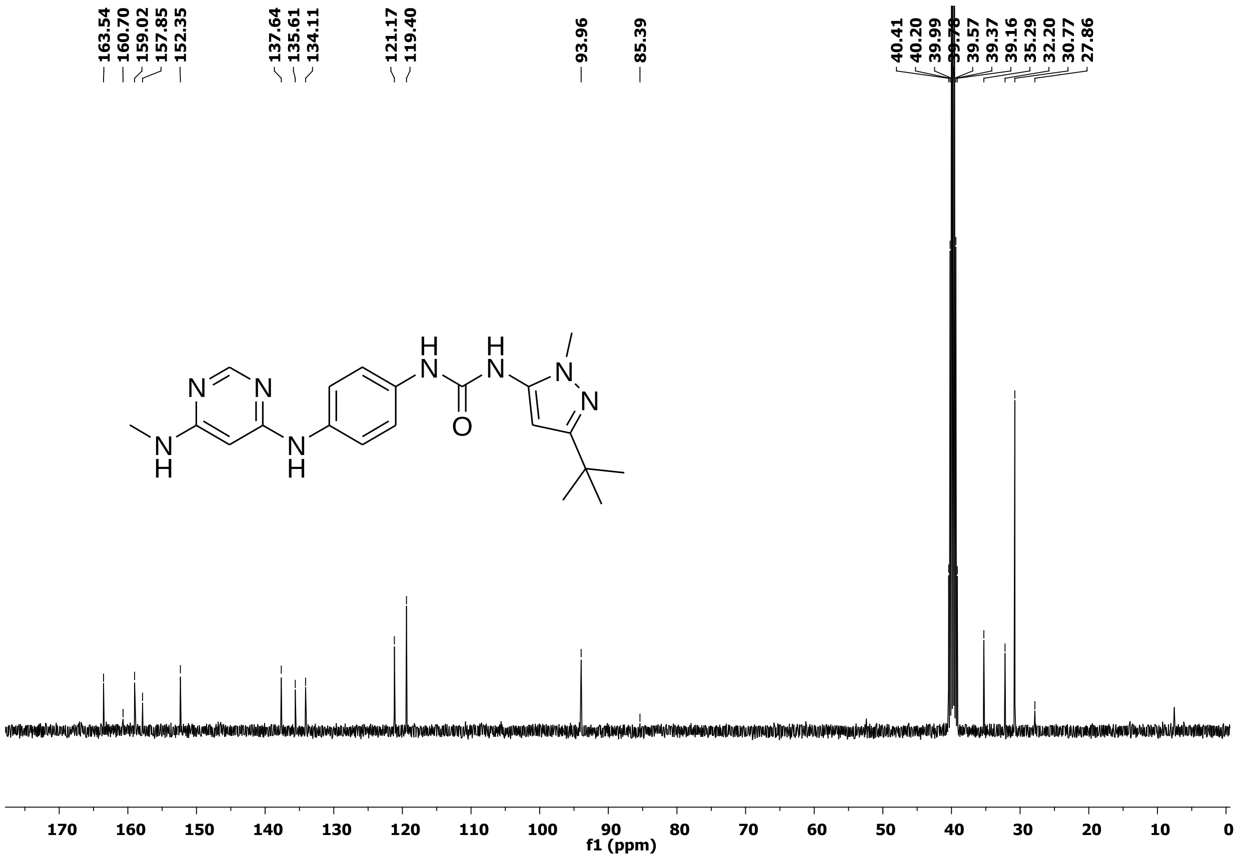

163.54
160.70
159.02
157.85
152.35
137.64
135.61
134.11
121.17
119.40
93.96
85.39
40.41
40.20
39.99
39.78
39.57
39.37
39.16
35.29
32.20
30.77
27.86
170 160 150 140 130 120 110 100 90 80 70 60 50 40 30 20 10 0
f1 (ppm)

S60. ^{1}H, ^{13}C spectrum of 1-(4-(6-(4-(methylsulfonyl)phenylamino)pyrimidin-4-ylamino)phenyl)-3-(5-tert-butylisoxazol-3-yl)urea (24c)

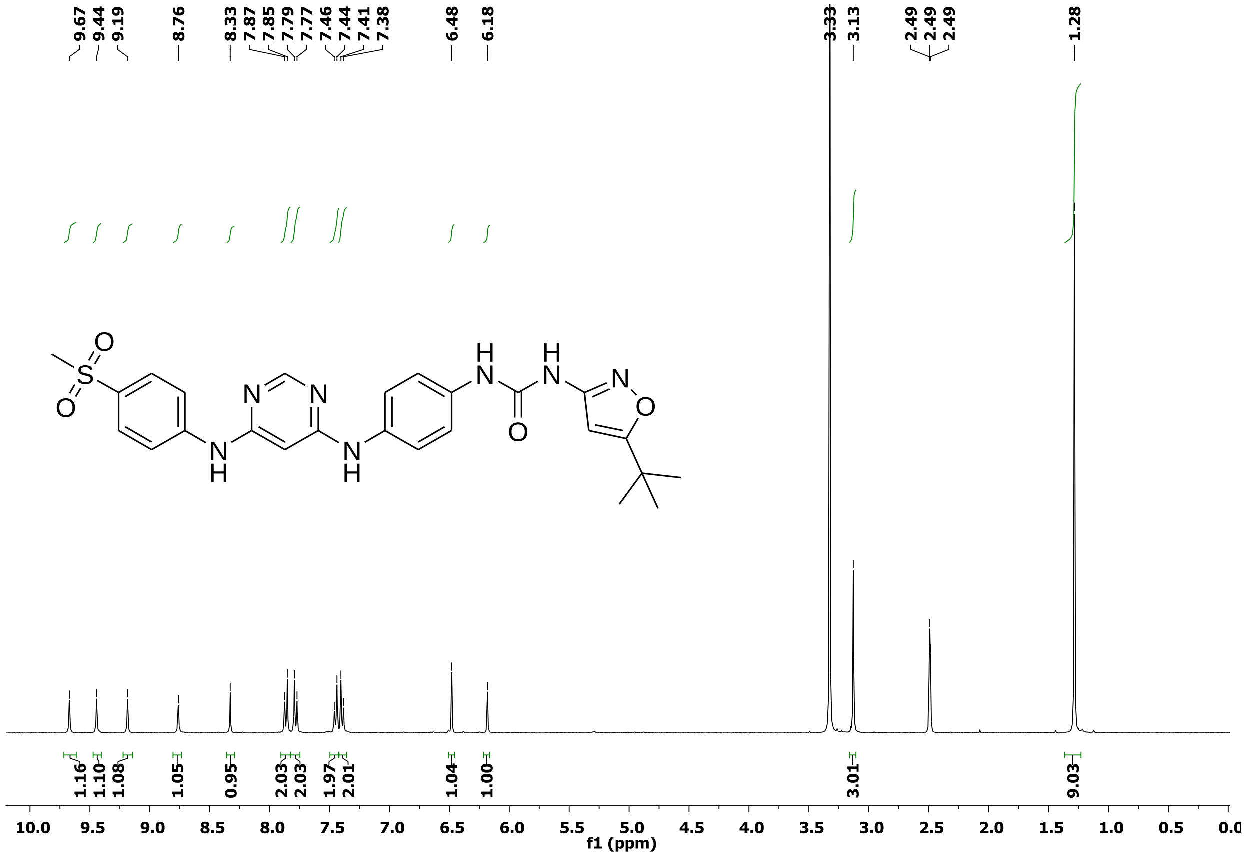

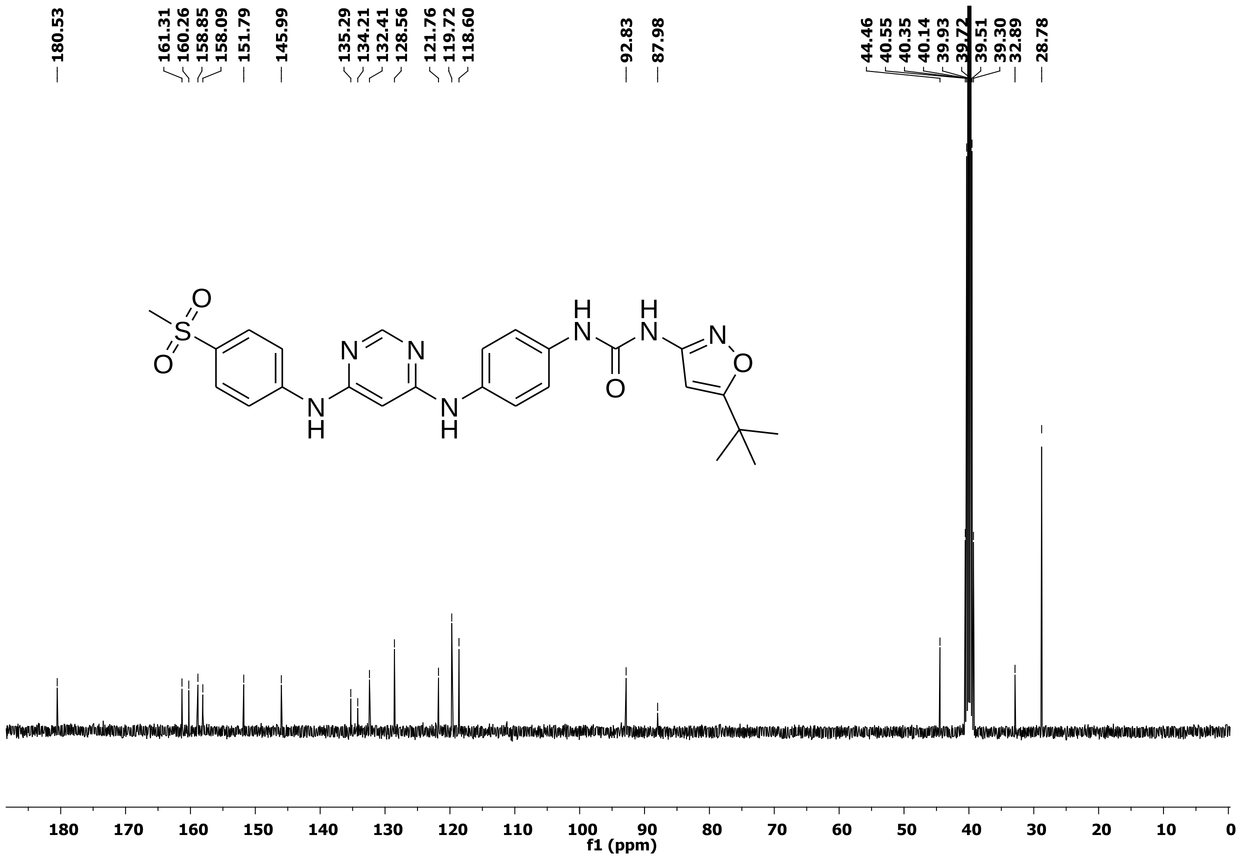
180.53
161.31
160.26
158.85
158.09
151.79
145.99
135.29
134.21
132.41
128.56
121.76
119.72
118.60
92.83
87.98
44.46
40.55
40.35
40.14
39.93
39.72
39.51
39.30
32.89
28.78
180 170 160 150 140 130 120 110 100 90 80 70 60 50 40 30 20 10 0
f1 (ppm)

S61. References cited in supporting information

1 Frett, B. *et al.* Identification of pyrazine-based TrkA inhibitors: design, synthesis, evaluation, and computational modeling studies. *MedChemComm* 5, 1507-1514 (2014).

2 Frett, B., Moccia, M., Carlomagno, F., Santoro, M. & Li, H. Y. Identification of two novel RET kinase inhibitors through MCR-based drug discovery: design, synthesis and evaluation. *Eur. J. Med. Chem.* 86, 714-723 (2014).

3 Trott, O. & Olson, A. J. AutoDock Vina: improving the speed and accuracy of docking with a new scoring function, efficient optimization and multithreading. *J. Comput. Chem.* 31, 455-461 (2010).

4 Smith, C. C. *et al.* Validation of ITD mutations in FLT3 as a therapeutic target in human acute myeloid leukaemia. *Nature* 485, 260–263 (2012).
